# Characterization of Triacylglycerol Estolide Isomers Using High-Resolution Tandem Mass Spectrometry with Nanoelectrospray Ionization

**DOI:** 10.3390/biom13030475

**Published:** 2023-03-03

**Authors:** Lukáš Cudlman, Aleš Machara, Vladimír Vrkoslav, Miroslav Polášek, Zuzana Bosáková, Stephen J. Blanksby, Josef Cvačka

**Affiliations:** 1 Institute of Organic Chemistry and Biochemistry of the Czech Academy of Sciences, Flemingovo Náměstí 542/2, 166 00 Prague 6, Czech Republic; 2 Department of Analytical Chemistry, Faculty of Science, Charles University, Hlavova 2030/8, 128 43 Prague 2, Czech Republic; 3 J. Heyrovský Institute of Physical Chemistry of the Czech Academy of Sciences, Dolejškova 2155/3, 182 23 Prague 8, Czech Republic; 4 School of Chemistry and Physics and the Central Analytical Research Facility, Queensland University of Technology, Brisbane, QLD 4000, Australia

**Keywords:** triacylglycerol estolides, glycerol *sn*-regioisomers, estolide-branching isomers, collision-induced dissociation, higher-energy collisional dissociation, ozone-induced dissociation, high-resolution mass spectrometry

## Abstract

Triacylglycerol estolides (TG-EST) are biologically active lipids extensively studied for their anti-inflammatory and anti-diabetic properties. In this work, eight standards of TG-EST were synthesized and systematically investigated by nanoelectrospray tandem mass spectrometry. Mass spectra of synthetic TG-EST were studied with the purpose of enabling the unambiguous identification of these lipids in biological samples. TG-EST glycerol *sn*-regioisomers and isomers with the fatty acid ester of hydroxy fatty acid (FAHFA) subunit branched in the ω-, α-, or 10-position were used. Ammonium, lithium, and sodium adducts of TG-EST formed by nanoelectrospray ionization were subjected to collision-induced dissociation (CID) and higher-energy collisional dissociation (HCD). Product ion spectra allowed for identification of fatty acid (FA) and FAHFA subunits originally linked to the glycerol backbone and distinguished the α-branching site of the FAHFA from other estolide-branching isomers. The ω- and 10-branching sites were determined by combining CID with ozone-induced dissociation (OzID). Lithium adducts provided the most informative product ions, enabling characterization of FA, hydroxy fatty acid (HFA), and FAHFA subunits. Glycerol *sn*-regioisomers were distinguished based on the relative abundance of product ions and unambiguously identified using CID/OzID of lithium and sodium adducts.

## 1. Introduction

Triacylglycerol estolides (TG-ESTs) are a minor class of lipids consisting of a glycerol backbone to which a combination of fatty acids (FA) and estolides is esterified (also reported as fatty acid esters of hydroxy fatty acid, FAHFA, and (*O*-acyl)-hydroxy fatty acid [1,2,3]) as a structural subunit [4]. TG-ESTs are biologically active molecules known from mammals [5,6,7,8,9,10,11], plants [12,13,14,15,16,17,18,19,20,21,22,23,24,25,26,27,28,29,30,31,32,33,34,35,36,37,38], and fungi [29,33,39,40,41]. In mammalian TG-ESTs, a hydroxy fatty acid (HFA) is usually esterified with a non-hydroxy FA (“capped” estolide), while in plants and fungi, another HFA may be attached (“uncapped” estolide). TG-ESTs serve as a reservoir of biologically active FAHFAs that exhibit various biological activities. FAHFAs are released from TG-ESTs by the action of lipases which may exhibit hydrolytic specificity regarding the estolide-branching site. A subgroup of FAHFAs with specific biological functions can thus be mobilized [7]. Understanding TG-EST and FAHFA metabolism can help to establish new strategies for treating obesity, diabetes, and other diseases [6,42].

The structural variability of TG-EST stems from numerous FA and HFA that can be combined. There are many TG-EST positional isomers, including glycerol *sn*-regioisomers that differ by the arrangement of acyl chains on the glycerol backbone and estolide-branching regioisomers with different positions of the estolide ester bond within the FAHFA subunit [6,7]. The structural variability of TG-EST is further extended by stereoisomers existing due to the chirality of the estolide-branching carbon [43]. The estolide regioisomers branched in the ω-position (terminal carbon atom of the HFA chain) are found in some plants [12,14,19,20,21,24,31]. The FAHFA subunit is, however, more often branched on an “inner” carbon of the HFA chain; such TG-ESTs exist in plants, fungi, and mammals [6,7,8,9,11,15,16,17,18,22,25,26,27,28,29,30,32,33,34,35,36,39,40,41]. To our knowledge, TG-ESTs with the FAHFA branched in the α-position (carbon number 2 of HFA) have not been reported yet. Their structural unit, α-FAHFA, was recently detected in vernix caseosa, a biofilm that covers the skin of newborn babies [3]. Additionally, the α-FAHFA is a major motif in estolides attached to sugar backbones as in lipid A within *Escherichia coli* [44]. Due to the metabolic connections between FAHFAs and TG-ESTs [6,7,42], the existence of TG-ESTs with α-branched FAHFA subunits is likely.

After hydrolysis and derivatization, TG-EST can be analyzed by gas chromatography [12,13,14,15,16,18,19,24,39,40]. High-performance liquid chromatography (HPLC) is preferred nowadays because it separates intact TG-EST molecules, preserving key structural information on ester bond linkages. Reversed-phase HPLC makes it possible to separate TG-EST isomers according to the number of carbon atoms, carbon–carbon double bonds, and the estolide-branching site in the FAHFA subunit [7]. HPLC coupled with mass spectrometry (HPLC/MS) offers excellent sensitivity and selectivity for TG-EST in biological samples [6,7,8,9,10,11,25]. Electrospray ionization (ESI) of this lipid class typically generates ammonium [M + NH_4_]^+^, sodium [M + Na]^+^, or lithium [M + Li]^+^ molecular adducts depending on conditions, with subsequent ion activation by collision-induced dissociation (CID) or higher-energy collisional dissociation (HCD) generating structurally informative product ions [6,7,8,9,10,25,26,27,28,35,37]. These spectra usually show product ions identifying the FA(s) linked to the glycerol backbone and the composition of the FAHFA subunit(s) [6,7,25,26,27]. Lithium adducts provide somewhat more informative spectra, with product ion peaks useful for deducing the sub-structure of FAHFA subunits and, in some cases, differentiating between glycerol *sn*-regioisomers [26,28]. The structure of lipid ions can also be probed by alternative ion activation methods, such as ozone-induced dissociation (OzID) [45,46]. OzID makes it possible to determine the carbon–carbon double bond position within the acyl chain [47,48], the position of estolide-branching [49], and the glycerol *sn*-position of the acyl chain [48,50,51]. Although some information on TG-EST fragmentation can be found in the literature [6,7,25,26,27], a systematic study of the fragmentation behavior of these lipids is not available. In the absence of these data, identifying TG-EST isomers in biological samples is challenging, and leads to a greater reliance on structural inferences from HPLC retention data [6,7,8,9,11].

In this study, we synthesized eight TG-EST standards to study the mass spectra of estolide-branching regioisomers and glycerol *sn*-regioisomers. Ammonium, lithium, and sodium adducts generated by nanoelectrospray ionization were fragmented using CID and HCD. Structurally significant ions were identified, and some unimolecular dissociation mechanisms are suggested and further investigated by multistage mass spectrometry, including with OzID.

## 2. Materials and Methods

### 2.1. Solvents and Additives

Acetonitrile (LC/MS grade) and propan-2-ol (LC/MS grade) were purchased from Biosolve BV (Valkenswaard, The Netherlands). Methyl *t*-butyl ether (>99.8%), methanol (99.9%), ammonium formate (>99.0%), lithium formate monohydrate (98%), and sodium formate (>99.0%,) were obtained from Sigma-Aldrich (St. Luis, MO, USA). Chloroform (99.8%) stabilized by ~1% ethanol from Penta (Chrudim, Czech Republic) was purified by distillation.

### 2.2. TG-EST Standards

**Figure 1:**
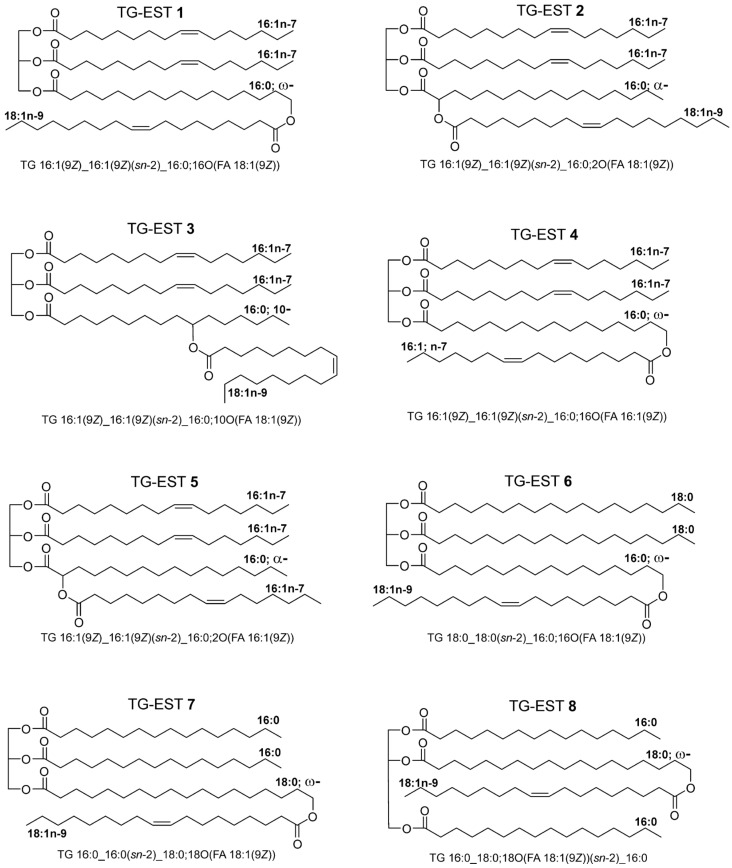
Studied structures of TG-EST **1**–**8**. Lipid nomenclature according to Liebisch et al. [52].

The structures of eight TG-EST standards investigated in this work are shown in Figure 1.

The standards each had one FAHFA subunit in either the *sn*-1/3 (Standards TG-EST **1**–**7**) or the *sn*-2 position (Standard TG-EST **8**). The FAHFA subunits contained the estolide ester bond in positions-2 (α; Standards TG-EST **2**, and **5**), -10 (Standard TG-EST **3**), or at the HFA chain terminus (ω; Standards TG-EST **1**, **4**, **6**, **7**, and **8**). The standards were synthesized by Steglich esterification mediated by *N,N*′-dicyclohexylcarbodiimide (DCC) [53]. 10-Hydroxyhexadecanoate, the key compound for preparation of TG-EST **3**, was prepared according to our previous work [54]. Details regarding the synthesis, together with spectral data of intermediates and final products, are provided in Supplementary Materials (Schemes S1–S8 and Figures S1–S75).

For CID and HCD mass spectrometry experiments, the TG-EST standards were dissolved in a mixture of chloroform: propan-2-ol: acetonitrile: aqueous salt solution (0.5:0.05:0.80:0.10, by vol.) at a concentration of 10 µg mL^−1^. The aqueous solutions of the salts (ammonium, lithium, or sodium formate) were prepared at a concentration of 1.0 mmol L^−1^.

For OzID experiments, standards of TG-EST were dissolved in a mixture of chloroform: methyl *t*-butyl ether: methanol (0.05:0.5:0.5, by vol.) at a concentration of 100 µg mL^−1^. The TG-EST sample was further diluted 1:1 (*v*/*v*) in methanolic salt solution (Li^+^ or Na^+^; 2.5 mmol L^−1^).

### 2.3. Mass Spectrometry

For CID and HCD workflows, nanoESI-MS^n^ experiments were performed on the Orbitrap Fusion Lumos Tribrid mass spectrometer (Thermo Fisher Scientific, San Jose, CA, USA) equipped with a robotic chip-based nanoESI apparatus TriVersa NanoMate (Advion, Inc., Ithaca, NY, USA). Data were recorded and interpreted manually using Xcalibur 4.1.50 software (Thermo). The TriVersa NanoMate, controlled by Chipsoft 8.3.1 software, was operated with an ionization spray voltage of 1800 V and nitrogen delivery gas at 0.5 psi. TG-EST solutions were loaded into 96-well plates, and the sample aliquots (5–15 μL) were aspirated and infused into the mass spectrometer. The Orbitrap mass analyzer was operated at a resolution of 120,000 FWHM, and *m*/*z* values were acquired with an accuracy of less than 3.0 ppm.

[M + NH_4_]^+^, [M + Li]^+^, and [M + Na]^+^ ions were fragmented in MS^2^ and MS^3^ using CID and HCD. Helium and nitrogen served as collision gases for CID and HCD, respectively. Values of normalized collision energies (NCEs) are shown in spectra and are a unitless parameter (as NCE automatically compensates for the mass dependence on collision energy). The precursor isolation window was 1.0–1.5 mass units. The mass spectra were recorded in the *m*/*z* range of 200−1200 for full MS and MS^2^, and a *m*/*z* range of 150–900 for MS^3^ was used. The energy-resolved dissociation curves (Supplementary Materials, Figures S76–S87) were calculated from spectra acquired by introducing incremental changes to NCE values. The relative ion abundance values were averaged from 20–40 scans.

For MS^3^ CID/OzID and MS^4^ CID/CID/OzID sequential workflows, nanoESI-MS^n^ experiments were performed on the LTQ Orbitrap Elite mass spectrometer (Thermo) modified for OzID experiments [55]. An ozone generator (Titan30 UHC; Absolute Ozone, Edmonton, AB, Canada) was used for the external production of ozone (ca. 17% in oxygen), which was introduced into the ion-trap region of the instrument. The TriVersa NanoMate was used in the same way as in the case of CID and HCD with identical parameters (ionization spray voltage, nitrogen delivery, resolution, precursor window, and accuracy). Lithiated estolide-branching isomers and lithiated/sodiated glycerol *sn*-regioisomers were activated by CID (NCE 40%). Depending on the sequential workflow, the re-isolated MS^2^ or MS^3^ CID product ions reacted with ozone for 1.0 s at NCE of 0–1%.

## 3. Results

### 3.1. Mass Spectra of ω-, 10-, and α-Estolide-Branching Regioisomers

The FAHFA subunit of TG-EST consists of an HFA esterified (via its hydroxy group) to a second FA. The position of the hydroxy group can range from second (α-)carbon (adjacent to the carboxylic acid moiety) to the final (ω-)carbon on the chain terminus. We investigated ω-, 10-, and α-estolide-branching regioisomers to establish whether their mass spectra provided information characteristic of the estolide ester bond position. When subjected to positive ion electrospray ionization from solutions containing ammonium, lithium, and sodium cations, TG-EST produced abundant [M + NH_4_]^+^, [M + Li]^+^, and [M + Na]^+^ adducts, respectively.

#### 3.1.1. Fragmentation of Ammonium Adducts

Ammonium formate and acetate are relatively volatile salts that are widely used in ESI and HPLC/MS. Ammonium adducts have been broadly adopted for the analysis neutral lipids, e.g., TGs [56,57], and they have also been utilized in the structural analysis of TG-ESTs [6,7,8,9,10,11,25,37]. Activation of these adducts typically leads to the neutral loss of ammonia, yielding product ions arising from proton transfer to the neutral lipid.

**Figure 2:**
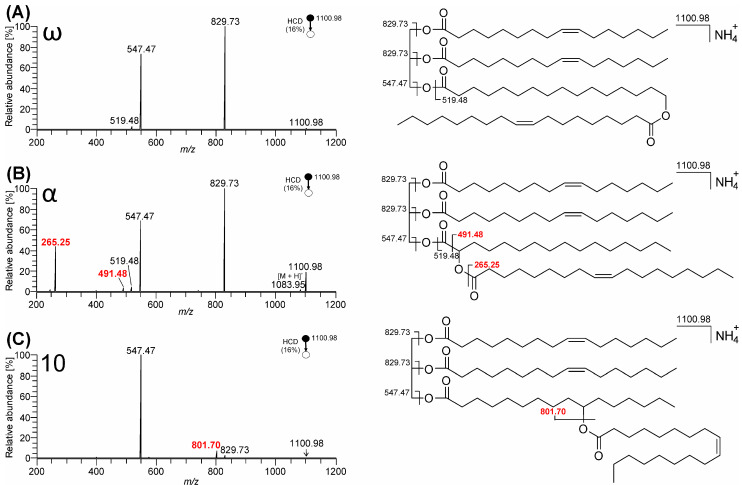
MS^2^ HCD spectra of TG-EST [M + NH_4_]^+^ (NCE 16%): (**A**) TG-EST **1**—ω-isomer; (**B**) TG-EST **2**—α-isomer; (**C**) TG-EST **3**—10-isomer. Diagnostic fragment ions for the various positional isomers are highlighted in red.

MS^2^ HCD spectra of TG-EST ammonium adducts yielded fragments consistent with the neutral loss of ammonia combined with FA and FAHFA. As illustrated in Figure 2, upon activation, ammonium adducts TG-EST **1**–**3** (*m*/*z* 1100.98; C_69_H_130_O_8_N^+^) were determined to eliminate ammonia and palmitoleic acid (*m*/*z* 829.73; C_53_H_97_O_6_^+^) and ammonia and the FAHFA subunit, i.e., ester of oleic and hydroxy palmitic acid (*m*/*z* 547.47; C_35_H_63_O_4_^+^). The elimination of oleic acid bound in the FAHFA subunit proceeded only for TG-EST **3** (*m*/*z* 801.70; C_51_H_93_O_6_^+^). In this isomer (10-), eliminations of FA linked to glycerol and bound in the FAHFA moiety were similarly efficient. Neutral loss of FAs from FAHFA moiety was documented previously for TG-EST with estolide-branching site on “inner” carbons of HFA chain [6,7].

In addition to neutral loss peaks, FAHFA acylium ion (*m*/*z* 519.48; C_34_H_63_O_3_^+^) was present at low abundance in TG EST **1** and TG-EST **2**. In the α-isomer (TG-EST **2**), *m*/*z* 491.48 (C_33_H_63_O_2_^+^) was probably formed from [M + H]^+^ (*m*/*z* 1083.95; C_69_H_127_O_8_^+^) by a cleavage of C1-C2 bond in HFA weakened by the esterified α-OH group. Oleic acid acylium ion (*m*/*z* 265.25; C_18_H_33_O^+^) likely originated from this ion after proton rearrangement, analogously to forming FA acylium ions from TGs [58] (p. 109). The abundant acylium ion made it possible to identify FA in the FAHFA subunit. As the acylium ions were formed only from the TG-EST **2** (Figure 2B) and TG-EST **5** (Figure S88B), they can be considered diagnostic ions for the α-estolide-branching regioisomers. Mass spectra MS^2^ HCD of ammonium adducts of TG-EST **4**–**8** are presented in Supplementary Materials (Figure S88).

The ion trap CID spectra contained fragments identical to HCD spectra, but the low mass cut-off in the ion trap prevented detection of product ions of *m*/*z* < 350 (Figure S89). Therefore, HCD was more useful for TG-EST structure elucidation than CID. Energy-resolved dissociation curves of TG-EST ammonium adducts helped us to determine the optimal value of NCE for MS^2^ experiments. As shown in Figures S76–S79, the optimum NCEs in CID were almost the same for TG-EST **1**–**3** (NCE 30–35). In HCD, the optimum NCE values ensuring the detection of all diagnostic product ions were dependent on the TG-EST structure. While TG-EST **1** required NCE values above 20, TG-EST **2** fragmented best at NCE around 15, and TG-EST **3** needed an NCE value set to approximately 10. This observation is consistent with differences in the energetics of competing dissociation pathways across the isomeric structures.

MS^3^ offered additional insight into the TG-EST structure. CID spectra of the first generator product ions at *m*/*z* 829.73 (Figure S90A–C) differed significantly for the investigated isomers. Under these conditions, all isomers eliminated the second palmitoleic acid (*m*/*z* 575.50; C_37_H_67_O_4_^+^) from the glycerol backbone and the oleic acid from the FAHFA subunit (*m*/*z* 547.47; C_35_H_63_O_4_^+^). Unlike the other isomers, the ω-isomer (TG-EST **1**) provided a strong FAHFA acylium ion signal (*m*/*z* 519.48; C_34_H_63_O_3_^+^) along with its dehydration product ions (*m*/*z* 501.47; *m*/*z* 483.46). The spectrum of α-isomer (TG-EST **2**) was characterized by the oleic acid acylium ion (*m*/*z* 265.25; C_18_H_33_O^+^) and neutral loss of palmitic acid ketene (*m*/*z* 593.51; C_37_H_69_O_5_^+^). MS^3^ of *m*/*z* 829.73 thus made it possible to distinguish the individual isomers. CID spectra of *m*/*z* 547.47 (Figure S90D–F) showed less significant differences in peak intensities, making these fragmentation patterns less likely to be useful for discriminating between isomers in a complex biological mixture. In theory, further information about the estolide structure could also be obtained by re-isolation and further dissociation of FAHFA acylium ion at *m*/*z* 519.48, but in practice, its abundance was too low to get measurable diagnostic product ion signals.

The MS^2^ HCD spectra of TG-EST ammonium adducts made it possible to characterize FAHFA subunit and FAs linked to glycerol. FA bound in the FAHFA subunit was detectable as an acylium ion in α-isomer and as a neutral loss in the 10-isomer. Therefore, the spectra allowed us to distinguish estolide-branching isomers and characterize all four FA chains in TG-EST, except for ω-isomers, for which the spectra did not provide information about individual chains in the FAHFA moiety (see spectra of TG-EST **4** and TG-EST **6** in Figure S88A,C, respectively). Determining the estolide-branching position in MS^2^ was thus possible for α- and 10-isomers. MS^3^ allowed us to obtain additional structural information; characteristic fragments indicating α- and ω-isomers were identified.

#### 3.1.2. Fragmentation of Lithium Adducts

**Figure 3:**
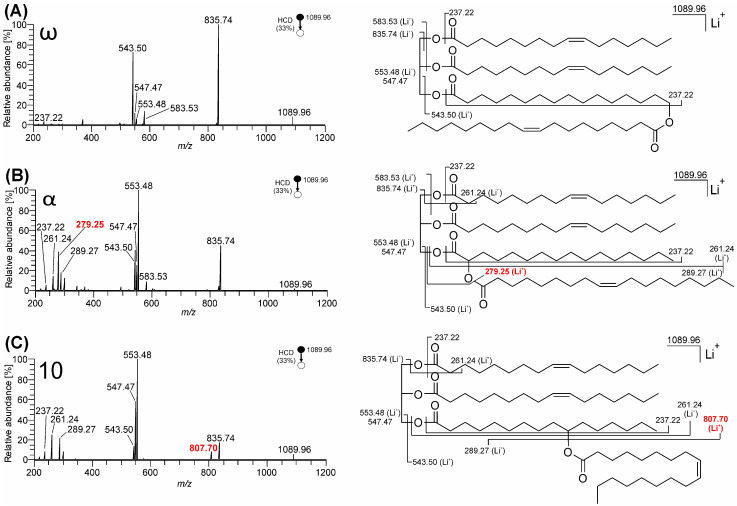
MS^2^ HCD spectra of TG-EST [M + Li]^+^ (NCE 33%): (**A**) TG-EST **1**—ω-isomer; (**B**) TG-EST **2**—α-isomer; (**C**) TG-EST **3**—10-isomer. Diagnostic fragment ions for the various positional isomers are highlighted in red.

Under CID and HCD conditions, lithium adducts of neutral lipids most often provide more structural information than the corresponding sodium or ammonium adducts due to the high oxygen affinity of the lithium [59,60,61]. Figure 3 shows MS^2^ HCD spectra of lithiated TG-EST **1**–**3** (*m*/*z* 1089.96; C_69_H_126_O_8_Li^+^). Some of the fragmentation pathways observed in these spectra resemble those observed for the ammonium adducts. Lithiated TG-EST eliminated palmitoleic acids linked to the glycerol backbone (*m*/*z* 835.74; C_53_H_96_O_6_Li^+^) and the FAHFA moiety both as the neutral acid (*m*/*z* 553.48; C_35_H_62_O_4_Li^+^) and its lithium salt (*m*/*z* 547.47; C_35_H_63_O_4_^+^). Eliminating oleic acid from the FAHFA subunit (*m*/*z* 807.70; C_51_H_92_O_6_Li^+^) occurred only from the 10-isomer (TG-EST **3**), with an efficiency comparable to the neutral loss of glycerol-linked FA.

**Figure 4:**
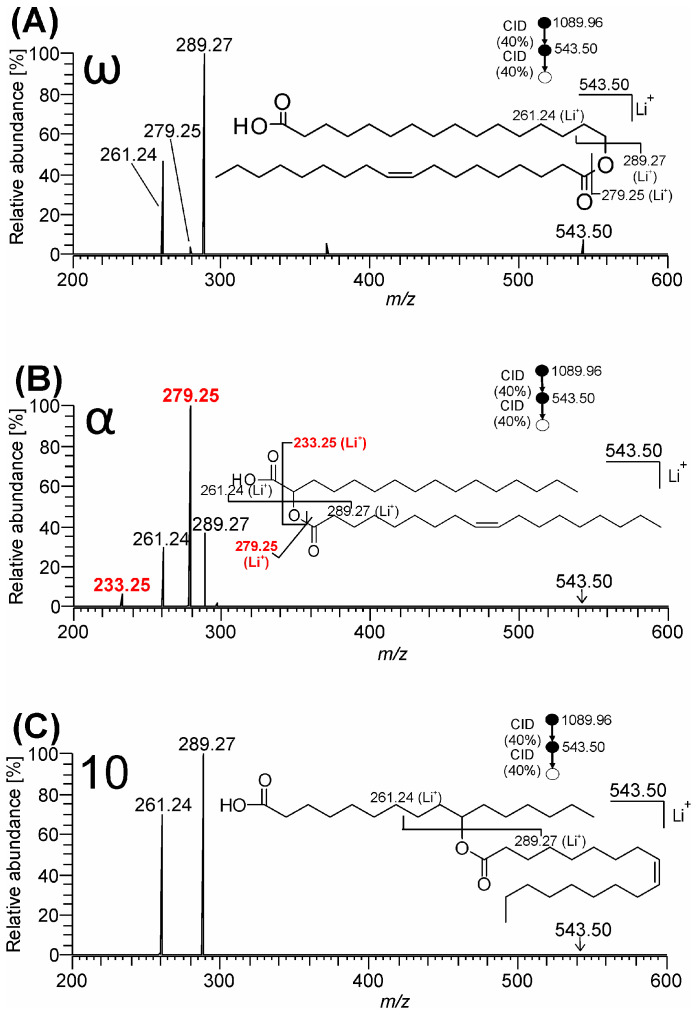
MS^3^ CID/CID spectra of TG-EST [M + Li]^+^. The product ion at *m*/*z* 543.50 arising from [M + Li]^+^ was further fragmented: (**A**) TG-EST **1**—ω-isomer; (**B**) TG-EST **2**—α-isomer; (**C**) TG-EST **3**—10-isomer. Diagnostic fragment ions for the various positional isomers are highlighted in red.

HCD of lithium adducts (Figure 3) also opened dissociation channels that were not observed for ammonium adducts. The FAHFA moiety provided an abundant lithiated fragment *m*/*z* 543.50 (C_34_H_64_O_4_Li^+^), which offered the possibility to characterize FAHFA subunit in the subsequent MS^3^ step (see below). Lithium adducts of TG-EST **1** (ω-) and **2** (α-) eliminated both glycerol-linked FAs (*m*/*z* 583.53; C_37_H_68_O_4_Li^+^) while this pathway was almost absent in the TG-EST **3** (10-isomer). These product ions likely arise following the elimination of the first palmitoleic acid by charge-remote fragmentation, as previously suggested for TGs [59] (see in Supplementary Materials, Figure S91). The acylium ion of palmitoleic acid originally bound to glycerol (*m*/*z* 237.22; C_16_H_29_O^+^) was detected as a small peak in all three TG-EST spectra. For α- and 10-isomers, lithiated palmitoleic acid (*m*/*z* 261.24; C_16_H_30_O_2_Li^+^) and lithiated oleic acid (*m*/*z* 289.27; C_18_H_34_O_2_Li^+^) ions were formed. The ion *m*/*z* 279.25 (C_16_H_32_O_3_Li^+^), corresponding to the lithium adduct of the HFA, was observed in the spectrum of α-isomer TG-EST **2** (Figure 3B) and TG-EST **5** (Figure S92B). This product ion was rationalized as resulting from the neutral loss of the ketene of oleic acid from *m*/*z* 543.50 that is the lithium adduct of FAHFA subunit. This proposal was confirmed by the explicit MS^3^ of *m*/*z* 543.50 shown in Figure 4B. This product ion was determined to be diagnostic of α-estolide-branching regioisomers as evidenced by the wider test-set of HCD spectra of TG-EST **4**–**8** lithium adducts that are presented in Figure S92.

The ion-trap CID mass spectra of lithium adduct ions yielded fragments identical to those of HCD, with the exception of the absence of low mass ions (Figure S93). The energy-resolved dissociation curves of lithiated TG-EST (Figures S80–S83) displayed an optimal NCE for MS^2^ HCD experiments of 33.

The MS^3^ experiments with lithiated FAHFA (*m*/*z* 543.50) were performed with the preceding ion trap-CID step providing a more abundant precursor ion than HCD. The MS^3^ (CID/CID) spectra of ω-, α-, and 10-isomers (TG-EST **1**–**3**) are shown in Figure 4.

All the spectra provided lithiated oleic acid (*m*/*z* 289.27; C_18_H_34_O_2_Li^+^) and lithiated FAHFA after the elimination of neutral oleic acid (*m*/*z* 261.24; C_16_H_30_O_2_Li^+^) with the latter ion corresponding to the lithium adduct of dehydrated HFA. The α-isomer MS^2^ spectrum (Figure 4B) showed abundant *m*/*z* 279.25 (C_16_H_32_O_3_Li^+^), the same ion already observed in MS^2^. Since the ion was negligibly small in ω- and absent in 10-isomers, it could be considered a diagnostic ion for α-estolide-branching regioisomers that carries information about HFA component of the FAHFA unit. This peak was accompanied by low abundant product ion at *m*/*z* 233.25 (C_15_H_30_OLi^+^), differing from it by the mass of CO_2_.

Compared to ammonium adducts, the CID and HCD mass spectra of the lithium adducts allowed a deeper insight into the structure of TG-EST. Abundant lithiated FAHFA species made it possible to determine FA and HFA in the FAHFA moiety for all estolide-branching regioisomers. As in the case of ammonium adducts, clear differentiation of the α-isomers from the others was possible. In summary, the MS^2^ HCD and MS^3^ CID/CID spectra of lithiated FAHFA made it possible to characterize the FAHFA subunit, i.e., to identify FA, HFA, and the α-estolide-branching site.

OzID has previously been proven to be a useful ion activation method for the structural analysis of lipids and in particular for the resolution of lipid regioisomers [47,48,49,50,51,62]. This technique harnesses ozonolysis reactions of mass-selected ions within the mass spectrometer to identify the location of carbon–carbon double bonds that are either present in the native lipid structure or are formed during preceding ion activation events. As ozonolysis is selective for carbon–carbon double bonds, only highly-specific fragmentations are induced that can provide for unambiguous structure elucidation [45,46]. We employed ozonolysis in a sequential CID/CID/OzID workflow to differentiate estolide-branching site of isomers. The MS^4^ spectra of lithiated TG-EST **1**–**3** are shown in Figure S94. Elimination of FA from FAHFA moiety yields dehydration products [49,63]. In the case of lithiated TG-EST **3** (10-isomer), the CID product ion *m*/*z* 261.24 is a mixture of lithiated 9- and 10-Hexadecenoic acids. Their ozonolysis in the final OzID step provided aldehyde ions *m*/*z* 179.12 (C_9_H_16_O_3_Li^+^) and *m*/*z* 193.14 (C_10_H_18_O_3_Li^+^) which unambiguously identified *n*-7 and *n*-6 carbon–carbon double bond positions and thus also the estolide-branching site (Figure S94C). The product ion *m*/*z* 263.22 (C_15_H_28_O_3_Li^+^) diagnostic for ω-estolide-branching site was observed in the spectrum of TG-EST **1** (Figure S94A). OzID did not provide any diagnostic fragment for the α-estolide-branching site in TG-EST **2** (Figure S94B).

#### 3.1.3. Fragmentation of Sodium Adducts

Sodium adducts are readily formed from neutral lipids [64], including TG-EST [25,36,41], and provide informative fragmentation spectra.

**Figure 5:**
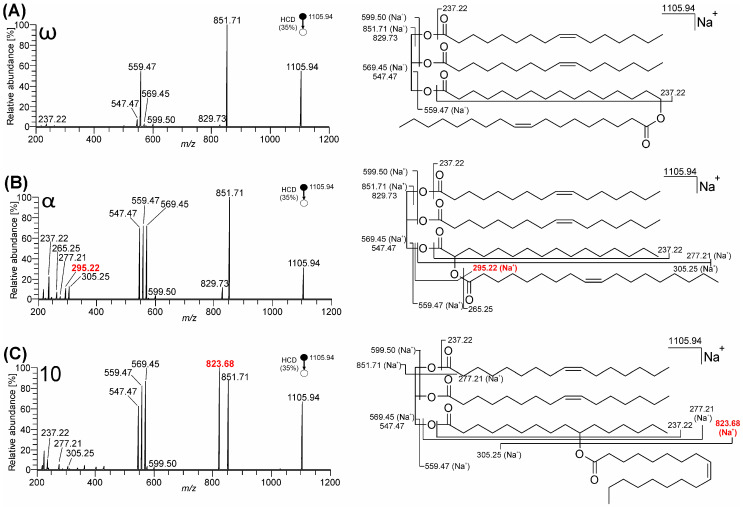
MS^2^ HCD spectra of TG-EST [M + Na]^+^ (NCE 35%): (**A**) TG-EST **1**—ω-isomer; (**B**) TG-EST **2**—α-isomer; (**C**) TG-EST **3**—ω-isomer. Diagnostic fragment ions for the various positional isomers are highlighted in red.

The MS^2^ HCD spectra of sodiated TG-EST **1**–**3** (*m*/*z* 1105.94; C_69_H_126_O_8_Na^+^) closely resembled spectra of lithium adducts (Figure 5). All estolide-branching regioisomers easily eliminated glycerol-linked palmitoleic acid (*m*/*z* 851.71; C_53_H_96_O_6_Na^+^) and readily formed sodiated FAHFA (*m*/*z* 559.47; C_34_H_64_O_4_Na^+^). Elimination of palmitoleic acid sodium salt (*m*/*z* 829.73; C_53_H_97_O_6_^+^) and combined loss of both glycerol-linked palmitoleic acids (*m*/*z* 599.50; C_37_H_68_O_4_Na^+^) yielded minor signals. While the neutral loss of FAHFA moiety as neutral acid (*m*/*z* 569.45; C_35_H_62_O_4_Na^+^) or sodium salt (*m*/*z* 547.47; C_35_H_63_O_4_^+^) proceeded inefficiently in ω- (TG-EST **1**), it was a favored process in α- (TG-EST **2**) and 10- (TG-EST **3**) isomers. Neutral loss of oleic acid from the FAHFA subunit occurred only from the 10-isomer (TG-EST **3**), yielding abundant fragment *m*/*z* 823.68 (C_51_H_92_O_6_Na^+^). In the low mass range region, acylium ions of glycerol-linked palmitoleic acid (*m*/*z* 237.22; C_16_H_29_O^+^) and HFA-linked oleic acid (*m*/*z* 265.25; C_18_H_33_O^+^) were detected, alongside with sodium adducts of these FAs (*m*/*z* 277.21; C_16_H_30_O_2_Na^+^ and *m*/*z* 305.25; C_18_H_34_O_2_Na^+^, respectively). The fragment *m*/*z* 295.22 (C_16_H_32_O_3_Na^+^) in the spectrum of the α-isomer (TG-EST **2**) corresponded to neutral loss of ketene of oleic acid from *m*/*z* 559.47 (FAHFA subunit sodium adduct). The HCD spectra of TG-EST **4**–**8** sodium adducts are presented in Supplementary Materials (Figure S95).

The HCD spectra of sodium adducts provided approximately the same structural information as lithium adducts. The ion trap CID of TG-EST sodium adducts showed fragments identical to HCD, with some ions missing due to the low mass cut-off in the ion trap (Figure S96). Further fragmentation of sodiated ions by collisional activation turned out to be more challenging than for lithium with great competition for dissociation to the bare cation upon CID and thus leading to a lower abundance of lipid-related product ions.

### 3.2. Mass Spectra of Glycerol sn-Regioisomers

Since the neutral loss of FAs from the *sn*-1 and *sn*-3 positions of TGs are equally favored and more competitive than losses from *sn*-2, the relative abundance of the resulting diacylglycerol-like product ions can be used to infer the regiochemical assignment [59,65].

**Figure 6:**
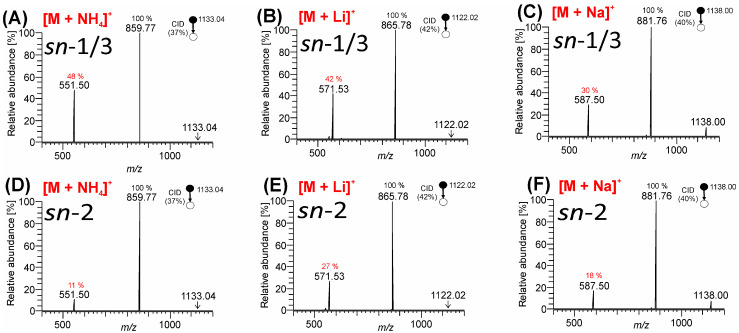
MS^2^ CID spectra of TG-EST glycerol *sn*-regioisomers. Variable molecular adducts of TG-EST **7** (*sn*-1/3) were fragmented: (**A**) [M + NH_4_]^+^; (**B**) [M + Li]^+^; and (**C**) [M + Na]^+^. Variable adducts of TG-EST **8** (*sn*-2) were fragmented: (**D**) [M + NH_4_]^+^; (**E**) [M + Li]^+^; and (**F**) [M + Na]^+^. Values of relative abundance ratios of fragments were described.

Here, we compared MS^2^ CID and HCD spectra of TG-EST **7** and TG-EST **8** differing by the relative position of FAHFA moiety on the glycerol backbone. As follows from the energy-resolved dissociation curves for [M + NH_4_]^+^, [M + Li]^+^, and [M + Na]^+^ (Figures S79, S83 and S87, respectively), CID provided a relatively stable ratio of fragment peak intensities across a wide range of NCE values (typically NCE 30–35). This was not the case for HCD, where a small change in NCE caused significant changes in product ion abundance. Therefore, CID was used to investigate the relative intensity ratios of peaks corresponding to the neutral loss of palmitic acid and FAHFA units from their positions on glycerol (Figure 6). All the spectra showed a greater peak intensity corresponding to the elimination of palmitic acid (*m*/*z* 859.77, C_55_H_103_O_6_^+^ for [M + NH_4_]^+^; *m*/*z* 865.78, C_55_H_102_O_6_Li^+^ for [M + Li]^+^; and *m*/*z* 881.76, C_55_H_102_O_6_Na^+^ for [M + Na]^+^), consistent with the presence of two palmitic acid radyls in each isomer. In contrast, the relative abundance of the product ions corresponding to the neutral loss of FAHFA (*m*/*z* 551.50, C_35_H_67_O_4_^+^, for [M + NH_4_]^+^) or lithiated/sodiated FAHFA (*m*/*z* 571.53, C_36_H_68_O_4_Li^+^ for [M + Li]^+^; and *m*/*z* 587.50, C_36_H_68_O_4_Na^+^ for [M + Na]^+^), while variable across the adducts, was consistently higher where the FAHFA was esterified at *sn*-1/3 (compared to FAHFA linked to *sn*-2). The effect was most pronounced in the case of ammonium adducts (Figure 6A,D). Therefore, the relative abundance of these diagnostic product ions consistently reflected the FAHFA position on the glycerol backbone.

Relying on product ion abundances can lead to ambiguous regiochemical assignments particularly where standard reference materials are not available or in the case of complex samples that may comprise isomeric mixtures. Therefore, we searched for product ions specific to the backbone regiochemistry of TG-EST subunit on glycerol.

**Figure 7:**
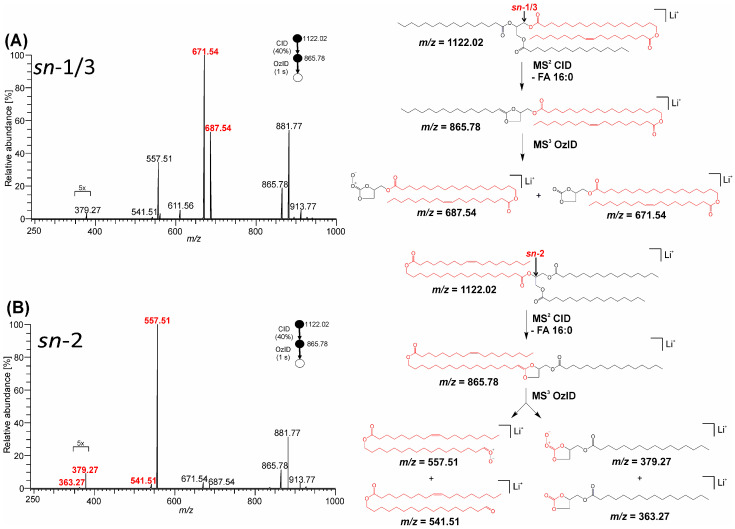
MS^3^ CID/OzID spectra of TG-EST [M + Li]^+^. The CID product ion at *m*/*z* 865.78 corresponding to neutral loss of palmitic acid was re-isolated and allowed to react with ozone: (**A**) TG-EST **7**—*sn*-1/3-isomer; (**B**) TG-EST **8**—*sn*-2-isomer. Diagnostic product ions for the various positional isomers are highlighted in red. Selected product ion abundances were magnified, as indicated.

Previous investigations have shown that CID of lithium and sodium adduct ions of TGs [50] lead to a five-membered 1,3-dioxolane ring and neutral the loss of one FA. The adjacent FA remains tethered to the glycerol by a newly formed carbon–carbon double bond that can react with ozone and reveal the adjacency of the two FA on the backbone. Analogous MS^3^ CID/OzID workflow was used for TG-EST **7** and TG-EST **8.** Activation of lithium and sodium adducts in CID resulted in a neutral loss of the palmitic acid originally linked to the glycerol backbone, yielding product ions at *m*/*z* 865.78 (C_55_H_102_O_6_Li^+^) and *m*/*z* 881.76 (C_55_H_102_O_6_Na^+^), respectively. When exposed to ozone, the lithiated product ion provided MS^3^ (CID/OzID) spectra shown in Figure 7. For both *sn*-isomers, an ozonide at *m*/*z* 913.77 (C_55_H_102_O_9_Li^+^) and its oxygen elimination product (*m*/*z* 881.77; C_55_H_102_O_7_Li^+^) were formed. Oxidative cleavage across the nascent carbon–carbon double bond led to Criegee, aldehyde and carbonate ester product ions indicative of the neutral loss of the FA and/or FAHFA moiety adjacent to the palmitic acid and thus a fragmentation pattern suitable for distinguishing the *sn*-regioisomers (Figure 7). The TG-EST **7**, with the FAHFA moiety in the *sn*-1/3 position, provided a pair of Criegee and carbonate ester fragments at *m*/*z* 687.54 (C_40_H_72_O_8_Li^+^) and *m*/*z* 671.54 (C_40_H_72_O_7_Li^+^), respectively. On the contrary, lithiated TG-EST **8** with the FAHFA moiety in the *sn*-2 decomposed to two pairs of diagnostic ions, (i) Criegee fragment *m*/*z* 557.51 (C_35_H_66_O_4_Li^+^) and aldehyde ion *m*/*z* 541.51 (C_35_H_66_O_3_Li^+^), and (ii) Criegee fragment *m*/*z* 379.27 (C_20_H_36_O_6_Li^+^) accompanied by carbonate ester *m*/*z* 363.27 (C_20_H_36_O_5_Li^+^).

**Figure 8:**
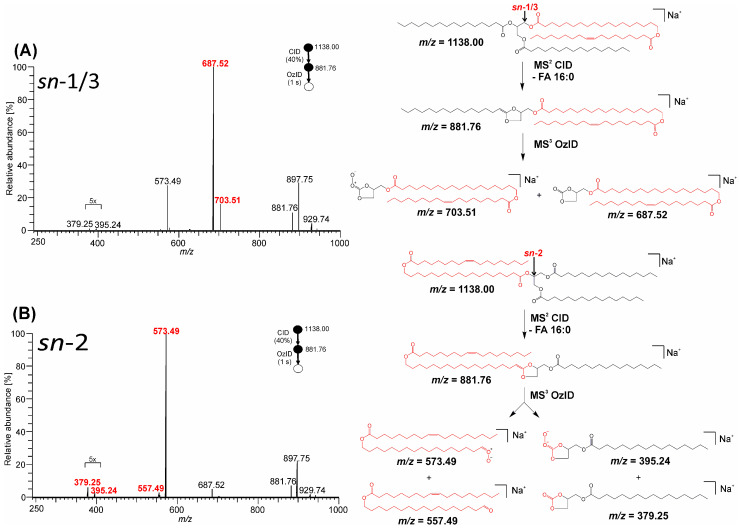
MS^3^ CID/OzID spectra of TG-EST [M + Na]^+^. The CID product ion at *m*/*z* 881.76 corresponding to neutral loss of palmitic acid was re-isolated and allowed to react with ozone: (**A**) TG-EST **7**—*sn*-1/3-isomer; (**B**) TG-EST **8**—*sn*-2-isomer. Diagnostic product ions for the various positional isomers are highlighted in red. Selected product ion abundances were magnified, as indicated.

An analogous fragmentation was observed for sodiated TG-EST isomers (Figure 8).

CID/OzID mass spectra of both sodiated and lithiated adduct ions showed the presence of low abundant product ions that were inconsistent with either the putative lipid regiochemistry or the exclusivity of the dissociation mechanisms indicated in Figure 7 and Figure 8, for example, the presence of a small amount of *m*/*z* 671.54 in Figure 7B and *m*/*z* 687.52 in Figure 8B. These ions point to either a small amount of the alternate regioisomer, perhaps arising from transacylation during synthesis, or the participation of an alternative, but poorly competitive, dissociation pathway. Future work to combine multi-stage ion activation with chromatographic resolution of isomers would be a suitable means to address this ambiguity.

## 4. Discussion

High-resolution tandem mass spectrometry of ammonium, lithium, and sodium adducts provided important pieces of information on the structure of TG-EST. By selecting the ion type and activation method, it was possible to distinguish TG-EST isomeric species and characterize their structural features. Lithium was determined to be the most useful cationization agent. Lithiated species provided more informative CID and HCD spectra than sodium and ammonium adducts. In the MS^2^, ions permitting the identification of FA, HFA, and FAHFA subunits were present, and some of the lithiated fragments allowed us to characterize branching of the FAHFA moiety. In the MS^3^, all isomers showed loss of FA from lithiated FAHFA ions; abundant ions corresponding to a loss of ketene of FA were additionally present in the α-isomers. The MS^4^ with OzID as the last fragmentation step allowed an even more detailed characterization of the estolide-branching site in FAHFA. In this experiment, ozonolysis was used to determine the position of carbon–carbon double bonds formed after elimination of FA from lithiated FAHFA. A similar level of structural information was obtained with sodiated TG-EST. Sodiated species were, however, difficult to fragment in subsequent MS steps because sodium tended to dissociate as bare cation during collisions. Despite their easy formation, ammonium adducts proved less useful as they did not provide FAHFA fragments that could be activated further. All the adducts allowed us to distinguish between *sn*-1/3 and *sn*-2 TG-EST isomers. The relative abundance of CID fragments corresponding to neutral loss of FAHFA and FA (ammonium adducts) or lithiated/sodiated FAHFA and neutral loss of FA was significantly higher for *sn*-1/3 isomers. The effect was pronounced the most in the case of ammonium adducts. The glycerol *sn*-regioisomers were also distinguished by CID/OzID of lithium or sodium adducts. Specific peaks in the spectra made it possible to identify the regioisomers unambiguously.

The fragmentation pathways described in this work are the basis for developing new methods for analyzing TG-EST in biological samples using HPLC/MS. A high-resolution mass spectrometer was used to understand the fragmentation pathways. High-resolution instruments are also recommended for analyzing biological samples; however, reliable identification of TG-EST can also be achieved using devices with lower resolving power. CID in QqQ or QTOF-type instruments can be expected to provide similar spectra to HCD discussed in this work. We worked with a concentration of standards of 10 µmol L^−1^. Spectra of similar quality can, however, be obtained at significantly lower concentrations (Figure S97) that are biologically relevant (concentration of TG-EST in the HPLC/MS samples are typically in µmol L^−1^ range). To comprehensively analyze TG-ESTs in biological matrices, it will be necessary to combine mass spectrometry with efficient chromatography. The HPLC separation of isomeric lipids is a great challenge, especially when it comes to very complex lipid matrices, such as vernix caseosa.

## Data Availability

The data presented in this study are available on request from the corresponding author.

## Supplementary Materials

The following supporting information can be downloaded at: https://www.mdpi.com/article/10.3390/biom13030475/s1; Schemes S1–S8: Preparation of TG-EST **1**–**8**; Figures S1–S75: ^1^H and ^13^C NMR spectra of prepared TG-EST **1**–**8** and their intermediates; Figures S76–S79: Energy-resolved dissociation curves for [M + NH_4_]^+^ of TG-EST **1**–**8** obtained by CID and HCD; Figures S80–S83: Energy-resolved dissociation curves for [M + Li]^+^ of TG-EST **1**–**8** obtained by CID and HCD; Figures S84–S87: Energy-resolved dissociation curves for [M + Na]^+^ of TG-EST **1**–**8** obtained by CID and HCD; Figure S88: MS^2^ HCD spectra of TG-EST [M + NH_4_]^+^ (NCE 16%) of TG-EST **4**–**8**; Figure S89: MS^2^ CID spectra of TG-EST [M + NH_4_]^+^ (NCE 30%) of TG-EST **1**–**6**; Figure S90: MS^3^ CID/CID spectra of TG-EST [M + NH_4_]^+^ of TG-EST **1**–**3**; Figure S91: Reaction mechanism for fragmentation of lithiated TG-EST **1** resulting in *m*/*z* 583.53; Figure S92: MS^2^ HCD spectra of TG-EST [M + Li]^+^ (NCE 33%) of TG-EST **4**–**8**; Figure S93: MS^2^ CID spectra of TG-EST [M + Li]^+^ (NCE 40%) of TG-EST **1**–**6**; Figure S94: MS^4^ CID/CID/OzID spectra of TG-EST [M + Li]^+^ of TG-EST **1**–**3**; Figure S95: MS^2^ HCD spectra of TG-EST [M + Na]^+^ (NCE 35%) of TG-EST **4**–**8**; Figure S96: MS^2^ CID spectra of TG-EST [M + Na]^+^ (NCE 40%) of TG-EST **1**–**6**; Figure S97: Mass spectra of TG-EST **2** at lower concentrations.
