# Supplementary material for: Characterization of Triacylglycerol Estolide Isomers Using High-Resolution Tandem Mass Spectrometry with Nanoelectrospray Ionization
Source: Biomolecules. 2023 Mar 3;13(3):475. doi: 10.3390/biom13030475 (PMC10046810; doi:10.3390/biom13030475)
Supplement: Supplementary file 1 [file biomolecules-13-00475-s001.zip › biomolecules-2223017_PROOF_Supplementary Material.pdf]

SUPPLEMENTARY MATERIAL

Characterization of Triacylglycerol Estolide Isomers Using High-Resolution Tandem Mass Spectrometry with Nanoelectrospray Ionization

Lukáš Cudlman [1,2], Aleš Machara [1], Vladimír Vrkoslav [1], Miroslav Polášek [3], Zuzana Bosáková [2], Stephen J. Blanksby [4], Josef Cvačka [1,2,*]

[1]Institute of Organic Chemistry and Biochemistry of the Czech Academy of Sciences, Flemingovo náměstí 542/2, 166 00 Prague 6, Czech Republic

[2]Department of Analytical Chemistry, Faculty of Science, Charles University, Hlavova 2030/8, 128 43 Prague 2, Czech Republic

[3]J. Heyrovský Institute of Physical Chemistry of the Czech Academy of Sciences, Dolejškova 2155/3, 182 23 Prague 8, Czech Republic

[4]School of Chemistry and Physics and the Central Analytical Research Facility, Queensland University of Technology, Brisbane, QLD 4000, Australia

*Correspondence to Josef Cvačka: josef.cvacka@uochb.cas.cz; +420-220-183-303

List of contents

1 Experimental procedures for the preparation of compounds ... 2
1.1 Preparation of TG-EST 1 ... 3
1.2 Preparation of TG-EST 2 ... 6
1.3 Preparation of TG-EST 3 ... 9
1.4 Preparation of TG-EST 4 ... 13
1.5 Preparation of TG-EST 5 ... 16
1.6 Preparation of TG-EST 6 ... 18
1.7 Preparation of TG-EST 7 ... 20
1.8 Preparation of TG-EST 8 ... 24
1.9 Copies of ^{1}H-NMR and ^{13}C-NMR spectra ... 26
2 Energy-resolved dissociation curves for TG-EST 1–8 ... 64
2.1 Ammonium adducts ... 64
2.2 Lithium adducts ... 67

2.3 Sodium adducts 70
3 Fragmentation of ammonium adducts 73
3.1 MS^2 HCD spectra of TG-EST 4–8 73
3.2 MS^2 CID spectra of TG-EST 1–6 74
3.3 MS^3 CID/CID spectra of TG-EST 1–3 75
4 Fragmentation of lithium adducts 76
4.1 Rationalization of *m/z* 583.53 (MS^2 HCD) 76
4.2 MS^2 HCD spectra of TG-EST 4–8 77
4.3 MS^2 CID spectra of TG-EST 1–6 78
4.4 MS^4 CID/CID/OzID spectra of TG-EST 1–3 79
5 Fragmentation of sodium adducts 80
5.1 MS^2 HCD spectra of TG-EST 4–8 80
5.2 MS^2 CID spectra of TG-EST 1–6 81
6 Mass spectra of TG-EST 2 at lower concentrations 82

1 Experimental procedures for the preparation of compounds

Unless otherwise noted, all reactions were carried out under argon in oven-dried glassware. Solvents were distilled from drying agents as indicated and transferred under argon: THF (Na/benzophenone), toluene (Na/benzophenone), and DCM (CaH_2). Chromatography was performed using Fluka silica gel 60 (0.040 – 0.063 mm) or Merck silica gel 60. Spots were detected both by UV light and a solution of $Ce(SO_4)_2 \cdot 4H_2O$ (1%) and $H_3P(Mo_3O_{10})_4$ (2%) in 10% sulfuric acid. All starting materials were used as purchased (Sigma Aldrich, TCI Chemicals) unless otherwise indicated. Steglich esterification mediated by DCC was done according to the standard procedure.

The NMR spectra were measured on a 400 MHz Bruker AVANCE III spectrometer (^1H at 400 MHz, and ^{13}C at 101 MHz. Chemical shifts are provided in δ-scale; coupling constants J are given in Hz.

High-resolution mass spectra were recorded using LTQ Orbitrap XL mass spectrometer (Thermo Fisher Scientific) at a resolution of 100,000 FWHM. The instrument was equipped with an electrospray ion source and controlled by Xcalibur software 4.1.50 (Thermo).

1.1 Preparation of TG-EST 1

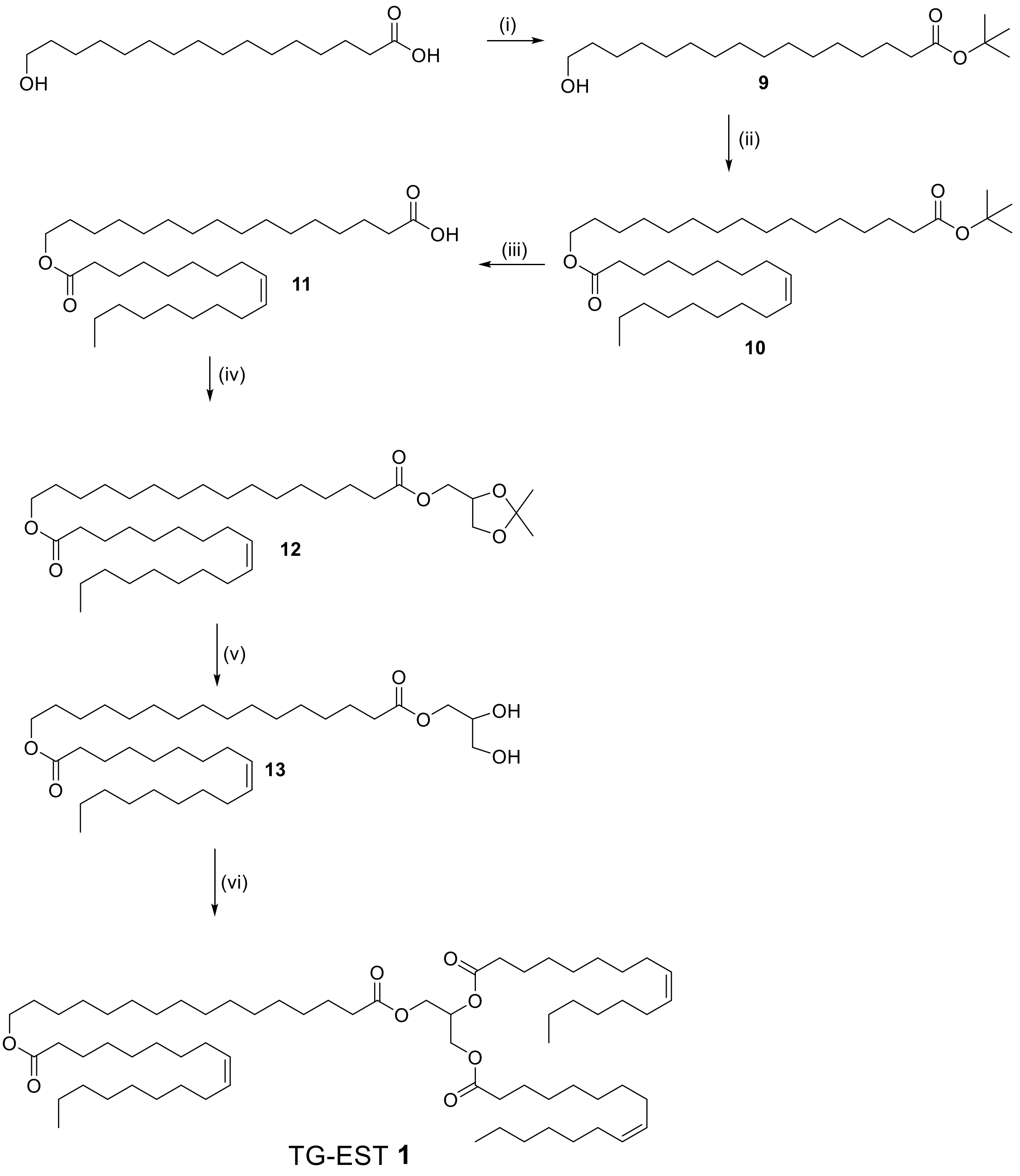

Scheme S1. Preparation of TG-EST **1**. *Reaction and conditions*: (i) *N*,*N*-dimethylformamide di-*tert*-butylacetal, 110 °C, toluene; (ii) DCC, oleic acid, DMAP, DCM; (iii) TFA, DCM; (iv) DCC, 2,3-*O*-isopropylidene glycerol, DMAP, DCM; (v) Amberlyst 15 H-form, methanol; (vi) DCC, (*Z*)-hexadec-9-enoic acid, DMAP, DCM.

Tert-butyl 16-hydroxyhexadecanoate (**9**)

16-Hydroxyhexadecanoic acid (0.1 g; 0.37 mmol) was suspended in toluene (3 mL) and *N*,*N*-dimethylformamide di-*tert*-butylacetal (0.15 g; 0.73 mmol) was added. The reaction mixture was stirred at 110 °C in a sealed ampoule overnight. Then the mixture was partitioned between water and EtOAc. After the organic layer was washed with brine and the solvent was evaporated, the crude product was purified by silica gel chromatography (eluent EtOAc:cyclohexane/1:1) affording 0.05 g (41%) of **9** as colorless oil.

^{1}H NMR (400 MHz, Chloroform-*d*) δ 3.67 (t, *J* = 6.6 Hz, 2H), 2.22 (m, 2H), 1.53-1.62 (m, 12H), 1.47 (s, 9H), 1.25-1.37 (m, 14H).

^{13}C NMR (100 MHz, Chloroform-*d*) δ 173.5, 80.0, 63.2, 35.7, 32.9, 29.75, 29.74, 29.72, 29.70, 29.59, 29.55, 29.42, 29.21, 28.2, 25.9, 25.2.

Tert-butyl 16-{[(9Z)-octadec-9-enoyl]oxy}hexadecanoate (**10**)

N,*N*'-Dicyclohexylcarbodiimide (DCC, 0.06 g; 0.29 mmol) was added to a stirred solution of *tert*-butyl 16-hydroxyhexadecanoate (0.08 g; 0.243 mmol), oleic acid (0.08 g; 0.29 mmol) and DMAP (0.003 g; 0.024 mmol) in DCM (3 mL). After stirring at room temperature for 16 h the reaction mixture was evaporated and the residual oil was purified by column chromatography (silica gel, eluent EtOAc:cyclohexane/1:8) to give 0.13 g (90%) of desired intermediate **10**.

^{1}H NMR (400 MHz, Chloroform-*d*) δ 5.58 – 5.15 (m, 2H), 4.08 (t, *J* = 6.7 Hz,2H), 2.31 (t, *J* = 7.6 Hz, 2H), 2.22 (t, *J* = 7.6 Hz, 2H), 2.03 (m, 4H), 1.70 – 1.59 (m, 6H), 1.47 (s, 9H), 1.39 – 1.21 (m, 40H), 0.90 (t, *J* = 6.5 Hz, 3H).

^{13}C NMR (100 MHz, Chloroform-*d*) δ 173.9, 173.3, 129.9, 129.7, 79.8, 64.4, 35.6, 34.4, 31.9, 29.8, 29.69, 29.67, 29.65, 29.61, 29.59, 29.53, 29.49, 29.32, 29.27, 29.18, 29.14, 29.11, 28.7, 27.2, 27.1, 25.9, 25.1, 25.0, 22.7, 14.1.

16-{[(9Z)-Octadec-9-enoyl]oxy}hexadecanoic acid (**11**)

To a solution of *tert*-butyl ester **10** (0.13 g; 0.22 mmol) in DCM (2 mL) placed in an ice bath was added dropwise neat trifluoroacetic acid (1 mL) and the reaction mixture was stirred at room temperature for 3 h. Then was the mixture evaporated on rotovap, the residue was twice evaporated with toluene and purified by column chromatography (silica gel, eluent EtOAc:cyclohexane/1:4) to give 0.10 g (84%) of acid **11**.

^{1}H NMR (400 MHz, Chloroform-*d*) δ 5.48 – 4.98 (m, 2H), 4.08 (t, *J* = 6.8 Hz, 2H), 2.37 (t, *J* = 7.5 Hz, 2H), 2.31 (t, *J* = 7.5 Hz, 2H), 2.16 – 1.85 (m, 4H), 1.69-1.62 (m, 4H), 1.43 – 1.18 (m, 40H), 1.06 – 0.82 (m, 3H).

(2,2-Dimethyl-1,3-dioxolan-4-yl)methyl 16-{[(9Z)-octadec-9-enoyl]oxy}hexadecanoate (**12**)

N,*N*'-Dicyclohexylcarbodiimide (DCC, 0.05 g; 0.22 mmol) was added to a stirred solution of 2,3-*O*-isopropylidene glycerol (0.04 g; 0.28 mmol), acid **11** (0.10 g; 0.18 mmol) and DMAP

(0.003 g; 0.022 mmol) in DCM (4 mL). After stirring at room temperature for 16 h the reaction mixture was evaporated and the residual oil was purified by column chromatography (silica gel, eluent EtOAc:cyclohexane/1:6) to give 0.075 g (62%) of desired intermediate **12**.

HR-ESI-MS calculated for $C_{40}H_{74}O_6Na$ $[M + Na]^+$ *m/z* 673.5383, found *m/z* 673.5368.

^1H NMR (400 MHz, Chloroform-*d*) δ 5.51 – 5.22 (m, 2H), 4.33 (qd, *J* = 6.1, 4.6 Hz, 1H), 4.18 (dd, *J* = 11.5, 4.7 Hz, 1H), 4.15 – 4.04 (m, 4H), 3.76 (dd, *J* = 8.4, 6.2 Hz, 1H), 2.36 (t, *J* = 7.6 Hz, 2H), 2.30 (t, *J* = 7.6 Hz, 2H), 2.03 (m, 4H), 1.63 (m, 6H), 1.45 (s, 3H), 1.39 (s, 3H), 1.37 – 1.20 (m, 40H), 0.90 (m, 3H).

^{13}C NMR (100 MHz, Chloroform-*d*) δ 173.9, 173.6, 129.9, 129.7, 109.8, 73.7, 66.4, 64.5, 64.4, 34.4, 34.1, 31.9, 29.8, 29.70, 29.67, 29.65, 29.61, 29.60, 29.55, 29.53, 29.47, 29.33, 29.27, 29.27, 29.18, 29.14, 29.12, 28.67, 26.69, 25.95, 25.41, 25.0, 24.9, 22.7, 14.1.

2,3-Dihydroxypropyl 16-{[(9Z)-octadec-9-enoyl]oxy}hexadecanoate (**13**)

Acetonide **12** (0.05 g; 0.07 mmol) and Amberlyst 15 H-form (0.05 g) were stirred at methanol (2 mL) overnight. Then catalyst was filtered off, the filtrate was evaporated, and the residue was purified by column chromatography (eluent EtOAc:cyclohexane/1:1) to give 0.016 g (34%) of 1-acylglycerol **13**.

HR-ESI-MS calculated for $C_{37}H_{70}O_6Na$ $[M + Na]^+$ *m/z* 633.5070, found *m/z* 633.5058.

^1H NMR (400 MHz, Chloroform-*d*) δ 5.59 – 5.19 (m, 2H), 4.31 – 4.12 (m, 2H), 4.08 (t, *J* = 6.8 Hz, 2H), 3.96 (tt, *J* = 6.0, 4.3 Hz, 1H), 3.72 (dd, *J* = 11.5, 4.0 Hz, 1H), 3.62 (dd, *J* = 11.5, 5.8 Hz, 1H), 2.37 (t, *J* = 7.6 Hz, 2H), 2.31 (t, *J* = 7.5 Hz, 2H), 2.03 (m, 4H), 1.75 – 1.47 (m, 6H), 1.42 – 1.21 (m, 40H), 0.90 (m, 3H).

^{13}C NMR (100 MHz, Chloroform-*d*) δ 174.4, 174.0, 130.0, 129.7, 70.3, 65.2, 64.4, 63.3, 34.4, 34.2, 31.9, 29.8, 29.70, 29.65, 29.63, 29.58, 29.53, 29.45, 29.33, 29.26, 29.25, 29.18, 29.15, 29.12, 28.7, 27.23, 27.18, 25.9, 25.0, 24.9, 22.7, 14.1.

rac-1,2-Di-((9Z)-hexadec-9-enoyl)-3-{16-{[(9Z)-octadec-9-enoyl]oxy}hexadecanoyl}glycerol (TG-EST **1**)

N,*N*’-Dicyclohexylcarbodiimide (DCC, 0.022 g; 0.10 mmol) was added to a stirred solution of 1-acyl glycerol **13** (0.016 g; 0.026 mmol), (*Z*)-hexadec-9-enoic acid (0.026 g; 0.10 mmol) and DMAP (0.002 g; 0.01 mmol) in DCM (3 mL). After stirring at room temperature for 16 h the reaction mixture was evaporated and the residual oil was purified by column chromatography (silica gel, eluent EtOAc:cyclohexane/1:12) to give 0.025 g (88%) of desired product TG-EST **1**.

HR-ESI-MS calculated for $C_{69}H_{126}O_8Na$ $[M + Na]^+$ *m/z* 1105.9350, found *m/z* 1105.9332.

^1H NMR (400 MHz, Chloroform-*d*) δ 5.48 – 5.32 (m, 6H), 5.29 (ddd, *J* = 5.9, 4.3, 1.6 Hz, 1H), 4.32 (dd, *J* = 11.9, 4.3 Hz, 2H), 4.17 (dd, *J* = 11.9, 6.0 Hz, 2H), 4.07 (t, *J* = 6.7 Hz, 2H), 2.47 – 2.22 (m, 6H), 2.03 (m, 10H), 1.78 – 1.50 (m, 10H), 1.42 – 1.22 (m, 80H), 1.10 – 0.77 (m, 9H).

^{13}C NMR (100 MHz, Chloroform-*d*) δ 174.0, 173.30, 173.28, 172.8, 130.03, 130.01, 129.99, 129.76, 129.72, 129.69, 68.9, 64.4, 62.1, 34.4, 34.2, 34.1, 34.0, 31.92, 31.79, 29.78, 29.74, 29.73, 29.71, 29.69, 29.66, 29.62, 29.57, 29.54, 29.51, 29.33, 29.30, 29.21, 29.19, 29.15, 29.12, 29.10, 29.06, 29.00, 28.7, 27.23, 27.18, 25.9, 25.0, 24.9, 24.87, 24.85, 22.70, 22.67, 14.1.

1.2 Preparation of TG-EST 2

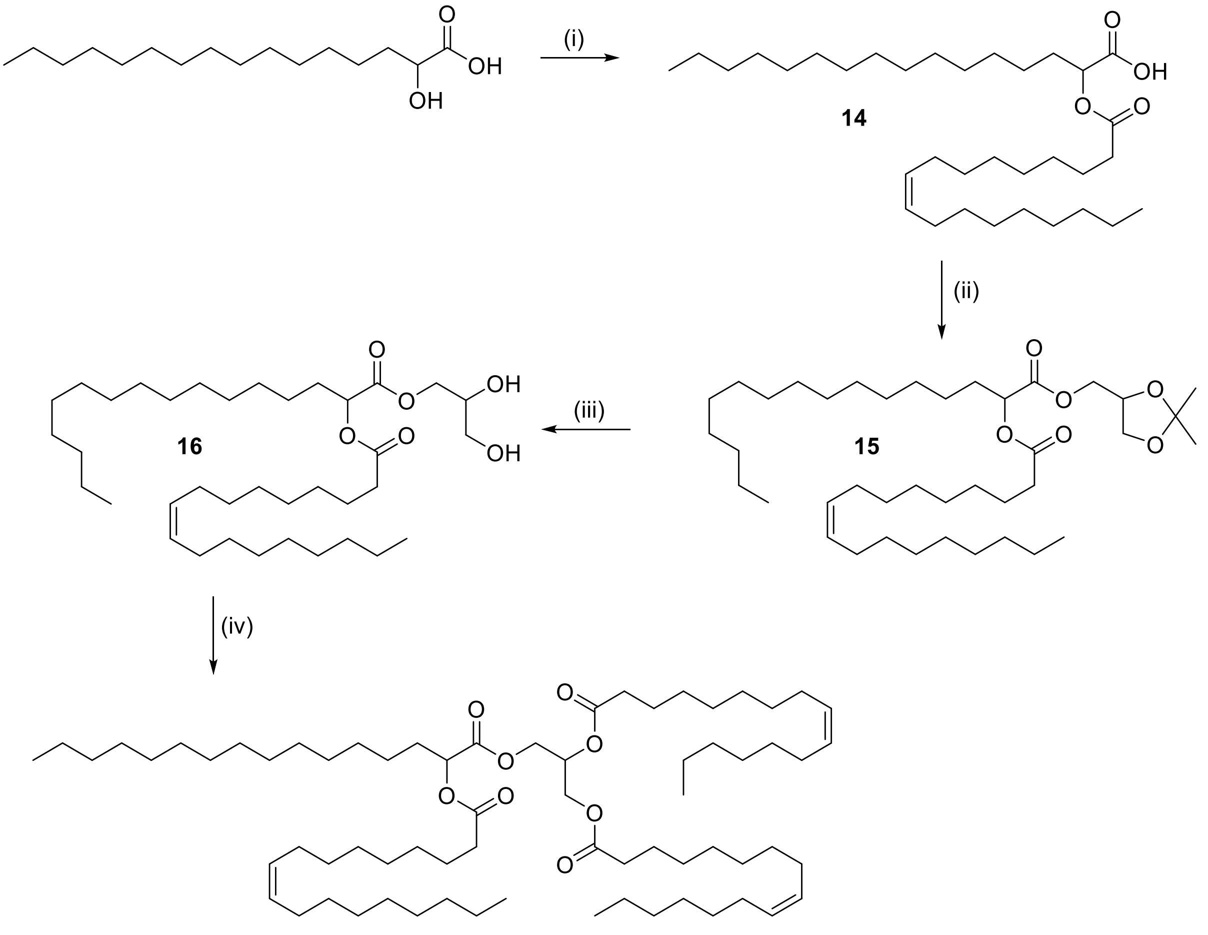

TG-EST **2**

Scheme S2. Preparation of TG-EST **2**. *Reaction and conditions*: (i) oleoyl chloride, pyridine, DCM; (ii) DCC, 2,3-*O*-isopropylidene glycerol, DMAP, DCM; (iii) Amberlyst 15 H-form, methanol, DCM; (iv) DCC, (*Z*)-hexadec-9-enoic acid, DMAP, DCM.

2-{[(9Z)-Octadec-9-enoyl]oxy}hexadecanoic acid (**14**)

A mixture of oleic acid (0.26 g; 0.9 mmol) and thionyl chloride (1 mL) in toluene (3 mL) was stirred at 100 °C for one hour in a sealed ampoule. Then was the mixture evaporated under reduced pressure and the residue was twice evaporated with toluene. Thus formed (Z)-octadec-9-enoyl chloride was diluted with DCM (3 mL) and immediately was added dropwise to a stirred solution of 2-hydroxyhexadecanoic acid (0.25 g; 0.9 mmol) and pyridine (1 mL) in DCM

(5 mL) at 0 °C. After stirring at room temperature for 16 hours, the mixture was diluted with DCM (15 mL), washed with 10% aqueous HCl (20 mL), water (10 mL) and brine (20 mL). The solvent was evaporated in vacuo and the oily residue was purified by column chromatography (silica gel, eluent EtOAc:cyclohexane/1:4) to give 0.32 g (66%) of 2-(oleoyl)hexadecanoic acid **14**.

HR-ESI-MS calculated for $C_{34}H_{63}O_4$ $[M – H]^-$ *m/z* 535.4726, found *m/z* 535.4730.

^{1}H NMR (400 MHz, Chloroform-*d*) δ 5.42 – 5.28 (m, 2H), 5.04 (dd, *J* = 7.1, 5.6 Hz, 1H), 2.42 (m, 2H), 2.03 (m, 4H), 1.95 – 1.84 (m, 2H), 1.67 (m, 2H), 1.50 – 1.41 (m, 2H), 1.39 – 1.21 (m, 40H), 0.98 – 0.82 (m, 6H).

^{13}C NMR (100 MHz, Chloroform-*d*) δ 174.9, 173.4, 130.0, 129.7, 71.6, 33.9, 31.9, 30.9, 29.78, 29.70, 29.67, 29.63, 29.54, 29.37, 29.34, 29.16, 29.11, 29.04, 27.23, 27.18, 25.1, 24.8, 22.7, 14.1.

(2,2-Dimethyl-1,3-dioxolan-4-yl)methyl 2-{[(9Z)-octadec-9-enoyl]oxy}hexadecanoate (**15**)

N,N'-Dicyclohexylcarbodiimide (DCC, 0.15 g; 0.71 mmol) was added to a stirred solution of 2,3-*O*-isopropylidene glycerol (0.12 g; 0.9 mmol), acid **14** (0.32 g; 0.60 mmol) and DMAP (0.009 g; 0.07 mmol) in DCM (5 mL). After stirring at room temperature for 16 h the reaction mixture was evaporated and the residual oil was purified by column chromatography (silica gel, eluent EtOAc:cyclohexane/1:6) to give 0.30 g (76%) of desired intermediate **15**.

HR-ESI-MS calculated for $C_{40}H_{74}O_6Na$ $[M + Na]^+$ *m/z* 673.5377, found *m/z* 673.5382.

^{1}H NMR (400 MHz, Chloroform-*d*) δ 5.46 – 5.24 (m, 2H), 5.08 – 4.94 (m, 1H), 4.46 – 4.27 (m, 1H), 4.28 – 4.20 (m, 2H), 4.08 (ddd, *J* = 8.5, 6.4, 0.9 Hz, 1H), 3.77 (ddd, *J* = 10.3, 8.5, 5.9 Hz, 1H), 2.60 – 2.24 (m, 2H), 2.03 (m, 4H), 1.95 – 1.78 (m, 2H), 1.67 (t, *J* = 7.3 Hz, 4H), 1.45 (s, 3H), 1.38 (s, 3H), 1.42 – 1.20 (m, 38H), 1.02 – 0.85 (m, 6H).

^{13}C NMR (100 MHz, Chloroform-*d*) δ 173.4, 170.4, 130.0, 129.7, 109.8, 73.4, 72.1, 66.3, 65.1, 33.9, 31.9, 31.1, 29.78, 29.71, 29.67, 29.63, 29.54, 29.39, 29.38, 29.33, 29.18, 29.13, 29.08, 27.23, 27.19, 26.71, 26.67, 25.4, 25.3, 25.2, 24.8, 22.7, 14.1.

2,3-Dihydroxypropyl 2-{[(9Z)-octadec-9-enoyl]oxy}hexadecanoate (**16**)

Acetonide **15** (0.20 g; 0.31 mmol) and Amberlyst 15 H-form (0.05 g) were stirred in a mixture of methanol (5 mL) and DCM (1 mL) for 3 days. Then was catalyst filtered off, the filtrate was evaporated and the residue was purified by column chromatography (eluent EtOAc:cyclohexane/1:1) to give 0.15 g (79%) of 1-acylglycerol **16**.

HR-ESI-MS calculated for $C_{37}H_{70}O_6Na$ $[M + Na]^+$ *m/z* 633.5070, found *m/z* 633.5061.

^{1}H NMR (400 MHz, Chloroform-*d*) δ 5.44 – 5.32 (m, 2H), 4.93 (t, *J* = 6.5 Hz, 1H), 4.58 – 4.16 (m, 2H), 4.05 – 3.88 (m, 1H), 3.79 – 3.68 (m, 1H), 3.63 (dd, *J* = 11.5, 5.5 Hz, 1H), 2.41 (td, *J* = 7.4, 1.3 Hz, 2H), 2.03 (m, 4H), 1.86 (m, 2H), 1.66 (m, 4H), 1.54 – 1.17 (m, 40H), 1.03 – 0.81 (m, 6H).

^{13}C NMR (100 MHz, Chloroform-*d*) δ 174.0, 170.9, 130.0, 129.7, 72.5, 69.9, 65.9, 63.1, 33.9, 31.9, 31.0, 29.8, 29.71, 29.67, 29.62, 29.54, 29.37, 29.33, 29.16, 29.12, 29.05, 27.23, 27.18, 25.2, 24.8, 22.7, 14.1.

rac-1,2-Di-((9Z)-hexadec-9-enoyl)-3-{2-{[(9Z)-octadec-9-enoyl]oxy}hexadecanoyl}glycerol (TG-EST **2**)

N,N'-Dicyclohexylcarbodiimide (DCC, 0.2 g; 0.98 mmol) was added to a stirred solution of 1-acyl glycerol **16** (0.15 g; 0.24 mmol), (*Z*)-hexadec-9-enoic acid (0.25 g; 0.98 mmol) and DMAP (0.012 g; 0.09 mmol) in DCM (5 mL). After stirring at room temperature for 16 h the reaction mixture was evaporated and the residual oil was purified by column chromatography (silica gel, eluent EtOAc:cyclohexane/1:10) to give 0.15 g (56%) of desired product TG-EST **2**.

HR-ESI-MS calculated for $C_{69}H_{126}O_8Na$ $[M + Na]^+$ *m/z* 1105.9350, found *m/z* 1105.9336.

^{1}H NMR (400 MHz, Chloroform-*d*) δ 5.43 – 5.33 (m, 6H), 5.32 – 5.23 (m, 1H), 4.98 (dt, *J* = 7.7, 6.4 Hz, 1H), 4.53 – 4.26 (m, 2H), 4.23 (m, 1H), 4.20 – 4.07 (m, 1H), 2.48 – 2.36 (m, 2H), 2.38 – 2.23 (m, 4H), 2.03 (m, 10H), 1.83 (m, 2H), 1.73 – 1.53 (m, 10H), 1.43 – 1.21 (m, 74H), 0.97 – 0.79 (m, 12H).

^{13}C NMR (100 MHz, Chloroform-*d*) δ 173.2, 172.7, 170.1, 169.6, 130.05, 130.01, 129.72, 129.69, 129.68, 71.9, 68.6, 62.8, 61.9, 34.1, 34.01, 33.92, 31.94, 31.92, 31.80, 29.78, 29.74, 29.72, 29.69, 29.66, 29.54, 29.38, 29.34, 29.23, 29.20, 29.15, 29.13, 29.11, 29.09, 29.00, 27.24, 27.19, 27.15, 24.8, 22.7, 22.7, 14.1.

1.3 Preparation of TG-EST 3

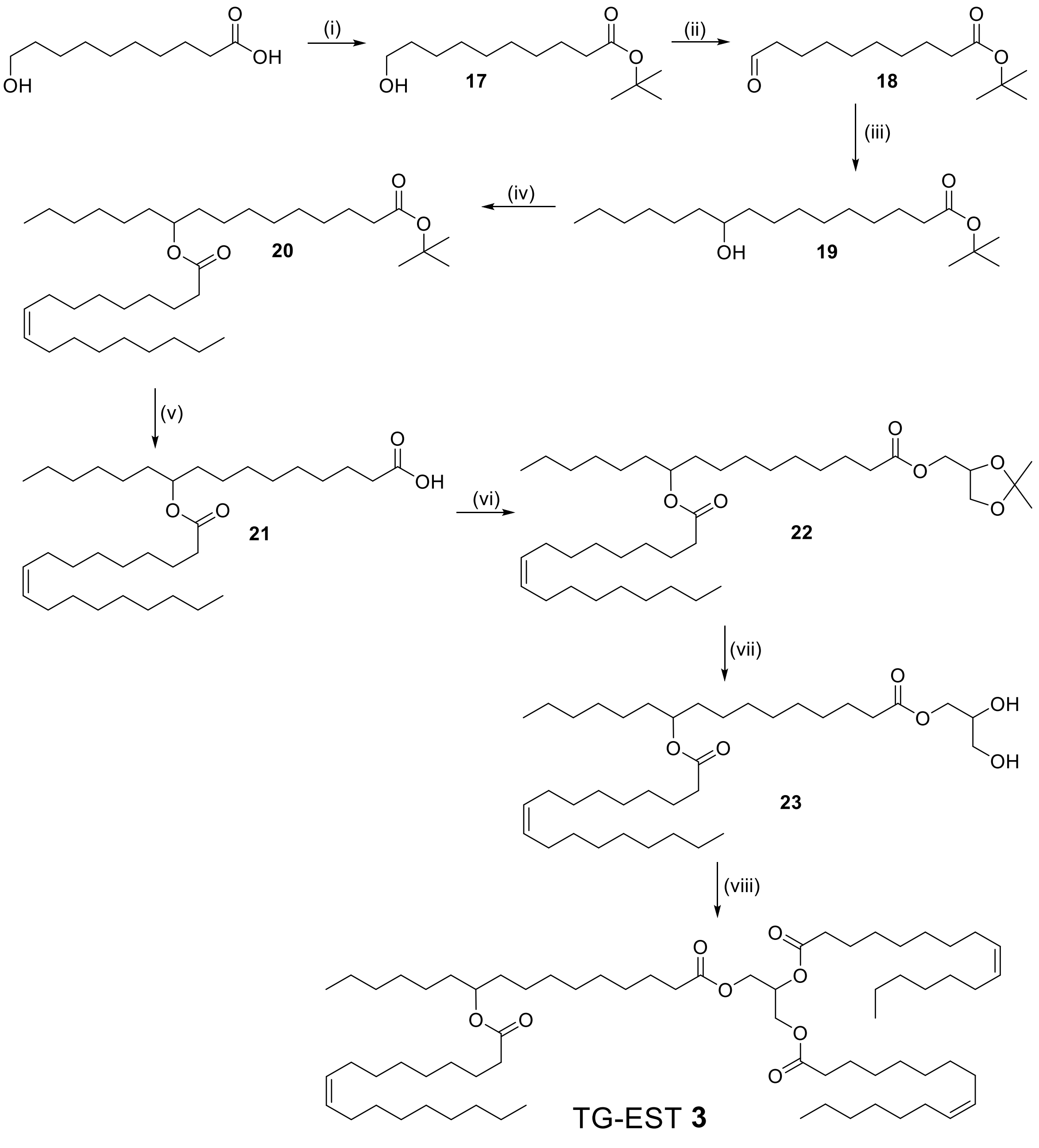

Scheme S3. Preparation of TG-EST **3**. *Reaction and conditions:* (i) *N*,*N*-dimethylformamide di-*tert*-butylacetal; (ii) PCC, DCM; (iii) hexylmagnesium bromide, THF; (iv) DCC, oleic acid, DMAP, DCM; (v) TFA, DCM; (vi) DCC, 2,3-*O*-isopropylidene glycerol, DMAP, DCM; (vii) Amberlyst 15 H-form, methanol, DCM; (viii) DCC, (*Z*)-hexadec-9-enoic acid, DMAP, DCM.

Tert-butyl 10-hydroxydecanoate (**17**)

10-Hydroxydecanoic acid (0.5 g; 2.65 mmol) was suspended in toluene (3 mL) and *N*,*N*-dimethylformamide di-*tert*-butylacetal (1.08 g; 5.31 mmol) was added. The reaction mixture was stirred at 110 °C in a sealed ampoule overnight. Then the mixture was partitioned between

water and EtOAc. After the organic layer was washed with brine and the solvent was evaporated, the crude product was purified by silica gel chromatography (eluent EtOAc:cyclohexane/1:2) affording 0.22 g (34%) of **17** as colorless oil.

HR-ESI-MS calculated for $C_{14}H_{28}O_3Na$ $[M + Na]^+$ *m/z* 267.1936, found *m/z* 267.1927.

^{1}H NMR (400 MHz, Chloroform-*d*) δ 3.66 (t, *J* = 6.6 Hz, 2H), 2.22 (t, *J* = 7.5 Hz, 2H), 1.65 – 1.54 (m, 4H), 1.47 (s, 9H), 1.32 (m, 10H).

^{13}C NMR (100 MHz, Chloroform-*d*) δ 173.4, 79.9, 63.1, 35.6, 32.8, 29.40, 29.34, 29.21, 29.06, 25.7, 25.1.

Tert-butyl 10-oxodecanoate (**18**)

Solid pyridinium chlorochromate (PCC, 0.39 g; 1.80 mmol) was added to a stirred suspension of *tert*-butyl 10-hydroxydecanoate **17** (0.22 g; 0.9 mmol) and Celite (0.50 g) in DCM (10 mL). After stirring at room temperature for 8 h the reaction mixture was evaporated and the solid material was loaded on a column of silica gel. Column chromatography (eluent EtOAc:cyclohexane/1:2) afforded 0.14 g (63%) of desired aldehyde **18**.

HR-ESI-MS calculated for $C_{14}H_{26}O_3Na$ $[M + Na]^+$ *m/z* 265.1780, found *m/z* 265.1773.

^{1}H NMR (400 MHz, Chloroform-*d*) δ 9.78 (s, 1H), 2.44 (td, *J* = 7.3, 1.9 Hz, 2H), 2.22 (t, *J* = 7.5 Hz, 2H), 1.82 – 1.52 (m, 4H), 1.46 (s, 9H), 1.41 – 1.05 (m, 10H).

^{13}C NMR (100 MHz, Chloroform-*d*) δ 202.9, 173.3, 79.9, 43.9, 35.6, 29.18, 29.10, 29.09, 29.00, 28.1, 25.1, 22.1.

Tert-butyl 10-hydroxyhexadecanoate (**19**)

Hexylmagnesium bromide (0.35 ml; 0.70 mmol, 2 M in diethyl ether) was added dropwise to a solution of *tert*-butyl 10-oxodecanoate **18** (0.14 g; 0.57 mmol) in THF (3 mL) at -78 °C. The reaction mixture was then removed from a cooling bath and was stirred at room temperature for 3 h. The reaction was quenched with addition of water (2 mL) and saturated solution of ammonium chloride (1 mL). Then the mixture was partitioned between water and EtOAc. After the organic layer was washed with brine and the solvent was evaporated, the crude product was purified by silica gel chromatography (eluent EtOAc:cyclohexane/1:4) affording 0.10 g (54%) of alcohol **19** as colorless oil.

HR-ESI-MS calculated for $C_{20}H_{40}O_3Na$ $[M + Na]^+$ *m/z* 351.2875, found *m/z* 351.2865.

^{1}H NMR (400 MHz, Chloroform-*d*) δ 3.60 (m, 2H), 2.22 (t, *J* = 7.5 Hz, 1H), 1.67 – 1.55 (m, 2H), 1.47 (m, 15H), 1.32 (m, 16H), 1.09 – 0.78 (m, 3H).

^{13}C NMR (100 MHz, Chloroform-*d*) δ 173.3, 79.9, 72.0, 37.5, 37.5, 35.6, 31.8, 29.6, 29.44, 29.39, 29.25, 29.07, 28.1, 25.6, 25.1, 22.6, 14.1.

Tert-butyl 10-{[(9Z)-octadec-9-enoyl]oxy}hexadecanoate (**20**)

N,*N*'-Dicyclohexylcarbodiimide (DCC, 0.11 g; 0.53 mmol) was added to a stirred solution of *tert*-butyl 10-hydroxyhexadecanoate **19** (0.15 g; 0.45 mmol), oleic acid (0.16 g; 0.53 mmol) and DMAP (0.006 g; 0.049 mmol) in DCM (5 mL). After stirring overnight at room temperature the reaction mixture was evaporated and the residual oil was purified by column chromatography (silica gel, eluent EtOAc:cyclohexane/1:10) to give 0.24 g (90%) of desired intermediate **20**.

HR-ESI-MS calculated for $C_{38}H_{72}O_4Na$ $[M + Na]^+$ *m/z* 615.5328, found *m/z* 615.5319.

^1H NMR (400 MHz, Chloroform-*d*) δ 5.46 – 5.31 (m, 2H), 4.88 (p, *J* = 6.3 Hz, 1H), 2.30 (t, *J* = 7.5 Hz, 2H), 2.22 (t, *J* = 7.5 Hz, 2H), 2.03 (m, 4H), 1.79 – 1.49 (m, 10H), 1.47 (s, 9H), 1.31 (m, 36H), 1.03 – 0.86 (m, 6H).

^{13}C NMR (100 MHz, Chloroform-*d*) δ 173.7, 173.3, 129.9, 129.8, 79.9, 74.1, 35.6, 34.7, 34.2, 31.9, 31.8, 29.78, 29.72, 29.54, 29.50, 29.38, 29.33, 29.25, 29.22, 29.18, 29.16, 29.08, 28.1, 27.23, 27.19, 25.32, 25.29, 25.18, 25.1, 22.7, 22.6, 14.13, 14.08.

10-{[(9Z)-Octadec-9-enoyl]oxy}hexadecanoic acid (**21**)

To a solution of *tert*-butyl ester **20** (0.22 g; 0.37 mmol) in DCM (4 mL) placed in an ice bath was added dropwise neat trifluoroacetic acid (2 mL) and the reaction mixture was stirred at room temperature for 3 h. Then the mixture was evaporated on rotovap, the residue was twice evaporated with toluene and purified by column chromatography (silica gel, gradient from EtOAc:cyclohexane/1:4 to EtOAc:cyclohexane/1:1) to give 0.16 g (80%) of acid **21**.

HR-ESI-MS calculated for $C_{34}H_{64}O_4Na$ $[M + Na]^+$ *m/z* 559.4702, found *m/z* 559.4695.

^1H NMR (400 MHz, Chloroform-*d*) δ 5.36 (m, 2H), 4.89 (m, 1H), 2.36 (t, *J* = 7.5 Hz, 2H), 2.30 (t, *J* = 7.4 Hz, 2H), 2.03 (m, 4H), 1.64 (m, 4H), 1.52 (m, 4H), 1.31 (m, 38H), 0.89 (m, 6H).

^{13}C NMR (100 MHz, Chloroform-*d*) δ 179.7, 173.8, 129.9, 129.7, 74.0, 34.7, 34.2, 34.15, 34.0, 31.92, 31.77, 29.78, 29.71, 29.54, 29.46, 29.33, 29.31, 29.21, 29.17, 29.15, 29.03, 27.23, 27.18, 25.3, 25.2, 24.6, 22.7, 22.6, 14.12, 14.08.

(2,2-Dimethyl-1,3-dioxolan-4-yl)methyl 10-{[(9Z)-octadec-9-enoyl]oxy}hexadecanoate (**22**)

N,*N*'-Dicyclohexylcarbodiimide (DCC, 0.11 g; 0.53 mmol) was added to a stirred solution of 2,3-*O*-isopropylidene glycerol (0.08 g; 0.62 mmol), acid **21** (0.24 g; 0.45 mmol) and DMAP (0.006 g; 0.049 mmol) in DCM (5 mL). After stirring at room temperature for 16 h the reaction mixture was evaporated and the residual oil was purified by column chromatography (silica gel, eluent EtOAc:cyclohexane/1:4) to give 0.25 g (52%) of desired intermediate **22**.

HR-ESI-MS calculated for $C_{40}H_{74}O_6Na$ $[M + Na]^+$ *m/z* 673.5383, found *m/z* 673.5375.

^1H NMR (400 MHz, Chloroform-*d*) δ 5.48 – 5.27 (m, 2H), 4.88 (m, 1H), 4.34 (m, 1H), 4.19 (dd, *J* = 11.5, 4.7 Hz, 1H), 4.16 – 4.05 (m, 2H), 3.76 (dd, *J* = 8.4, 6.2 Hz, 1H), 2.36 (t, *J* = 7.6

Hz, 2H), 2.30 (t, *J* = 7.5 Hz, 2H), 2.09 – 1.94 (m, 4H), 1.63 (m, 4H), 1.57 (m, 6H), 1.46 (s, 3H), 1.40 (s, 3H), 1.31 (m, 38H), 0.90 (m, 6H).

^{13}C NMR (100 MHz, Chloroform-*d*) δ 173.7, 173.6, 129.9, 129.8, 109.8, 74.0, 73.7, 66.4, 64.5, 34.7, 34.2, 34.1, 31.9, 31.7, 29.78, 29.72, 29.54, 29.49, 29.36, 29.33, 29.22, 29.20, 29.18, 29.16, 29.10, 27.23, 27.19, 26.7, 25.4, 25.3, 25.29, 25.2, 24.9, 22.7, 22.6, 14.13, 14.09.

2,3-Dihydroxypropyl 10-{[(9Z)-octadec-9-enoyl]oxy}hexadecanoate (**23**)

Acetonide **22** (0.15 g; 0.14 mmol) and Amberlyst 15 H-form (0.10 g) were stirred in a mixture of methanol (5 mL) and DCM (1 mL) for 3 days. Then was catalyst filtered off, the filtrate was evaporated and the residue was purified by column chromatography (eluent EtOAc:cyclohexane/1:1) to give 0.10 g (70%) of 1-acylglycerol **23**.

HR-ESI-MS calculated for $C_{37}H_{70}O_6Na$ [M + Na]$^+$ *m/z* 633.5070, found *m/z* 633.5062.

^{1}H NMR (400 MHz, Chloroform-*d*) δ 5.45 – 5.20 (m, 2H), 4.87 (p, *J* = 6.3 Hz, 1H), 4.42 – 4.05 (m, 2H), 3.94 (m, 1H), 3.70 (dd, *J* = 11.5, 3.9 Hz, 1H), 3.60 (dd, *J* = 11.5, 5.9 Hz, 1H), 2.35 (t, *J* = 7.5 Hz, 2H), 2.29 (t, *J* = 7.5 Hz, 2H), 2.02 (m, 4H), 1.86 – 1.56 (m, 4H), 1.51 (m, 4H), 1.47 – 1.18 (m, 40H), 0.89 (m, 6H).

^{13}C NMR (100 MHz, Chloroform-*d*) δ 174.3, 173.9, 129.9, 129.7, 74.1, 70.2, 65.1, 63.4, 34.7, 34.2, 34.1, 31.9, 31.7, 29.76, 29.70, 29.52, 29.39, 29.32, 29.29, 29.20, 29.16, 29.14, 29.12, 29.03, 27.21, 27.17, 25.3, 25.2, 24.9, 22.7, 22.56, 14.12, 14.07.

rac-1,2-Di-((9Z)-hexadec-9-enoyl)-3-{10-{[(9Z)-octadec-9-enoyl]oxy}hexadecanoyl}glycerol (TG-EST **3**)

N,*N*'-Dicyclohexylcarbodiimide (DCC, 0.14 g; 0.65 mmol) was added to a stirred solution of 1-acyl glycerol **23** (0.10 g; 0.16 mmol), (*Z*)-hexadec-9-enoic acid (0.16 g; 0.65 mmol) and DMAP (0.008 g; 0.065 mmol) in DCM (5 mL). After stirring at room temperature overnight the reaction mixture was evaporated and the residual oil was purified by column chromatography (silica gel, eluent EtOAc:cyclohexane/1:10) to give 0.19 g (63%) of desired product TG-EST **3**.

HR-ESI-MS calculated for $C_{69}H_{126}O_8Na$ [M + Na]$^+$ *m/z* 1105.9350, found *m/z* 1105.9335.

^{1}H NMR (400 MHz, Chloroform-*d*) δ 5.42 – 5.31 (m, 6H), 5.31 – 5.19 (m, 1H), 4.88 (m, 1H), 4.31 (dd, *J* = 11.8, 4.2 Hz, 2H), 4.16 (dd, *J* = 11.9, 6.0 Hz, 2H), 2.53 – 2.12 (m, 10H), 2.02 (d, *J* = 6.2 Hz, 12H), 1.88 – 1.57 (m, 10H), 1.51 (m, 4H), 1.40 – 1.19 (m, 66H), 0.89 (m, 12H).

^{13}C NMR (100 MHz, Chloroform-*d*) δ 173.6, 173.23, 173.22, 172.8, 130.00, 129.99, 129.96, 129.73, 129.70, 129.67, 74.0, 68.8, 62.1, 34.7, 34.2, 34.17, 34.03, 34.01, 31.9, 31.79, 31.77, 29.77, 29.74, 29.71, 29.53, 29.51, 29.37, 29.33, 29.21, 29.19, 29.17, 29.15, 29.12, 29.11, 29.09, 29.05, 28.99, 27.22, 27.17, 25.33, 25.29, 25.17, 24.88, 24.84, 24.83, 22.69, 22.66, 22.59, 14.11, 14.07.

1.4 Preparation of TG-EST 4

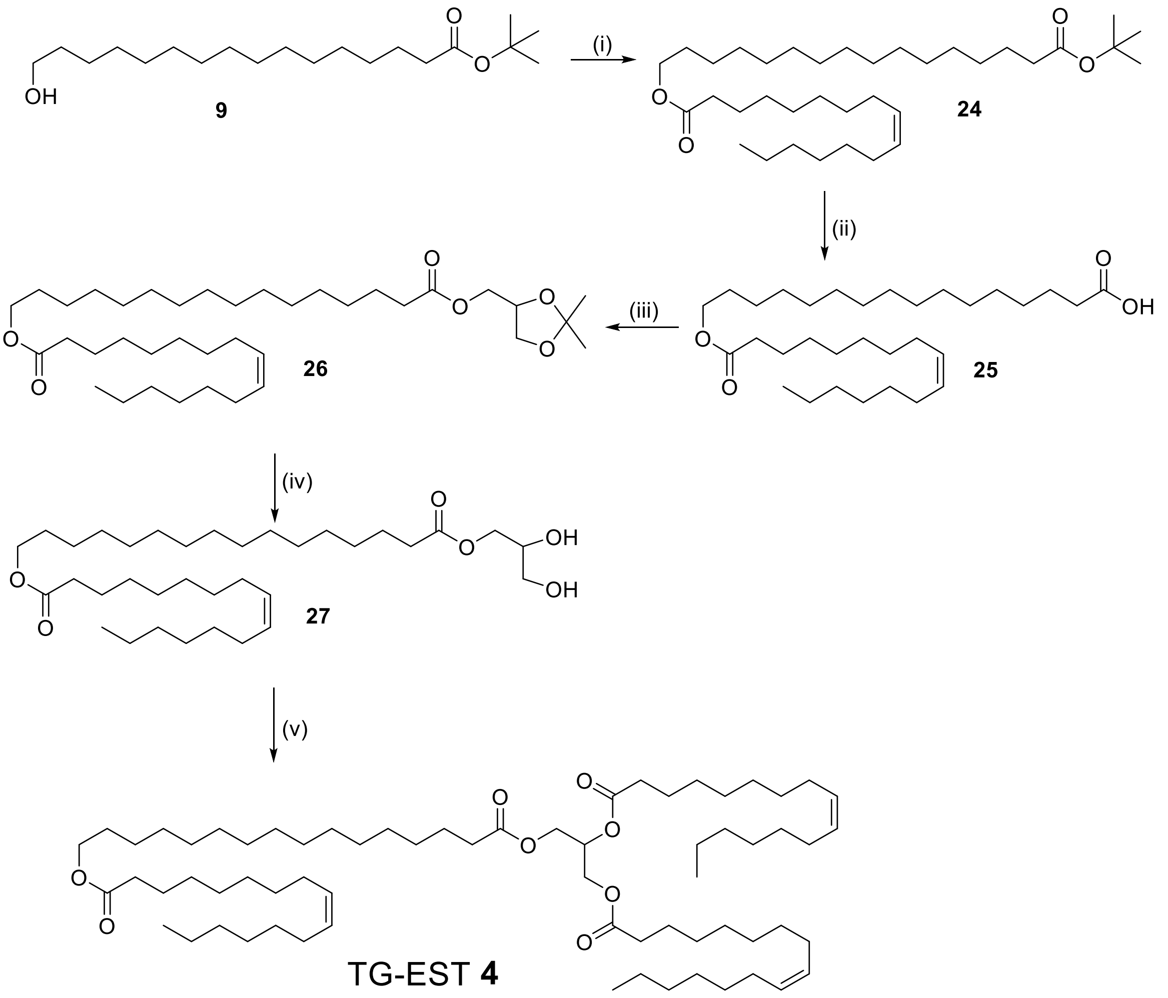

Scheme S4. Preparation of TG-EST **4**. *Reaction and conditions:* (i) DCC, (*Z*)-hexadec-9-enoic acid, DMAP, DCM; (ii) TFA, DCM; (iii) DCC, 2,3-*O*-isopropylidene glycerol, DMAP, DCM; (iv) Amberlyst 15 H-form, 2-methoxyethanol; (v) DCC, (*Z*)-hexadec-9-enoic acid, DMAP, DCM.

Tert-butyl 16-{[(9Z)-hexadec-9-enoyl]oxy}hexadecanoate (**24**)

N,*N*'-Dicyclohexylcarbodiimide (DCC, 0.11 g; 0.51 mmol) was added to a stirred solution of *tert*-butyl 16-hydroxyhexadecanoate **9** (0.14 g; 0.43 mmol), (*Z*)-hexadec-9-enoic acid (0.13 g; 0.51 mmol) and DMAP (0.006 g; 0.051 mmol) in DCM (4 mL). After stirring at room temperature for 16 h the reaction mixture was evaporated and the residual oil was purified by column chromatography (silica gel, eluent EtOAc:cyclohexane/1:10) to give 0.22 g (92%) of desired intermediate **24**.

HR-ESI-MS calculated for $C_{36}H_{68}O_4Na$ $[M + Na]^+$ *m/z* 587.5015, found *m/z* 587.5011.

^{1}H NMR (400 MHz, Chloroform-*d*) δ 5.47 – 5.28 (m, 2H), 4.08 (t, *J* = 6.8 Hz, 2H), 2.31 (t, *J* = 7.5 Hz, 2H), 2.22 (t, *J* = 7.5 Hz, 2H), 2.12 – 1.92 (m, 4H), 1.61 (m, 6H), 1.47 (s, 9H), 1.39 – 1.22 (m, 40H), 0.90 (m, 3H).

^{13}C NMR (100 MHz, Chloroform-*d*) δ 174.0, 173.4, 129.9, 129.8, 79.8, 64.4, 35.6, 34.4, 31.8, 29.75, 29.70, 29.68, 29.66, 29.62, 29.60, 29.55, 29.50, 29.32, 29.28, 29.18, 29.15, 29.12, 29.00, 28.7, 28.1, 27.23, 27.17, 25.9, 25.1, 25.0, 22.7, 14.1.

16-{[(9Z)-Hexadec-9-enoyl]oxy}hexadecanoic acid (**25**)

To a solution of *tert*-butyl ester **24** (0.21 g; 0.37 mmol) in DCM (4 mL) placed in an ice bath was added dropwise neat trifluoroacetic acid (2 mL) and the reaction mixture was stirred at room temperature for 3 h. Then the mixture was evaporated on rotovap, the residue was twice evaporated with toluene and purified by column chromatography (silica gel, eluent EtOAc:cyclohexane/1:4) to give 0.18 g (95%) of acid **24**.

HR-ESI-MS calculated for $C_{32}H_{59}O_4$ $[M – H]^–$ *m/z* 507.4413, found *m/z* 507.4412.

^{1}H NMR (400 MHz, Chloroform-*d*) δ 5.54 – 5.12 (m, 2H), 4.08 (t, *J* = 6.7 Hz, 2H), 2.37 (t, *J* = 7.5 Hz, 2H), 2.31 (t, *J* = 7.6 Hz, 2H), 2.03 (m, 4H), 1.64 (m, 6H), 1.31 (m, 40H), 0.90 (m, 3H).

^{13}C NMR (100 MHz, Chloroform-*d*) δ 178.7, 174.0, 129.9, 129.8, 64.4, 34.4, 33.8, 31.8, 29.8, 29.7, 29.6, 29.59, 29.54, 29.44, 29.27, 29.25, 29.18, 29.15, 29.12, 29.07, 29.00, 28.7, 27.2, 27.2, 25.9, 25.0, 24.7, 22.7, 14.1.

(2,2-Dimethyl-1,3-dioxolan-4-yl)methyl 16-{[(9Z)-hexadec-9-enoyl]oxy}hexadecanoate (**26**)

N,*N*'-Dicyclohexylcarbodiimide (DCC, 0.08 g; 0.40 mmol) was added to a stirred solution of 2,3-*O*-isopropylidene glycerol (0.066 g; 0.50 mmol), acid **25** (0.17 g; 0.33 mmol) and DMAP (0.005 g; 0.04 mmol) in DCM (4 mL). After stirring at room temperature for 16 h the reaction mixture was evaporated and the residual oil was purified by column chromatography (silica gel, eluent EtOAc:cyclohexane/1:6) to give 0.15 g (73%) of desired intermediate **26**.

HR-ESI-MS calculated for $C_{38}H_{70}O_6Na$ $[M + Na]^+$ *m/z* 645.5070, found *m/z* 645.5058.

^{1}H NMR (400 MHz, Chloroform-*d*) δ 5.58 – 5.10 (m, 2H), 4.34 (qd, *J* = 6.2, 4.7 Hz, 1H), 4.19 (dd, *J* = 11.5, 4.7 Hz, 1H), 4.15 – 4.01 (m, 4H), 3.76 (dd, *J* = 8.4, 6.2 Hz, 1H), 2.36 (t, *J* = 7.6 Hz, 2H), 2.31 (t, *J* = 7.5 Hz, 2H), 2.03 (m, 4H), 1.64 (m, 6H), 1.46 (s, 3H), 1.40 (s, 3H), 1.37 – 1.22 (m, 36H), 0.91 (m, 3H).

^{13}C NMR (100 MHz, Chloroform-*d*) δ 174.0, 173.6, 129.9, 129.8, 109.8, 73.7, 66.4, 64.5, 64.4, 34.4, 34.1, 31.8, 29.74, 29.70, 29.66, 29.61, 29.61, 29.55, 29.47, 29.27, 29.18, 29.14, 29.12, 29.00, 28.7, 27.23, 27.17, 26.7, 25.9, 25.4, 25.0, 24.9, 22.7, 14.1.

2,3-Dihydroxypropyl 16-{[(9Z)-hexadec-9-enoyl]oxy}hexadecanoate (**27**)

Acetonide **26** (0.15 g; 0.24 mmol) and Amberlyst 15 H-form (0.1 g) were stirred at 2-methoxyethanol (2 mL) for 6 h. Then was catalyst filtered off, the filtrate was evaporated and

the residue was purified by column chromatography (eluent EtOAc:cyclohexane/1:1) to give 0.09 g (64%) of 1-acylglycerol **27**.

HR-ESI-MS calculated for $C_{35}H_{66}O_6Na$ $[M + Na]^+$ *m/z* 605.4757, found *m/z* 605.4758.

^{1}H NMR (400 MHz, Chloroform-*d*) δ 5.44 – 5.25 (m, 2H), 4.20 (qd, *J* = 11.6, 5.4 Hz, 2H), 4.07 (t, *J* = 6.7 Hz, 2H), 3.95 (tt, *J* = 6.0, 4.3 Hz, 1H), 3.72 (dd, *J* = 11.4, 4.0 Hz, 1H), 3.62 (dd, *J* = 11.4, 5.8 Hz, 1H), 2.37 (t, *J* = 7.6 Hz, 2H), 2.31 (t, *J* = 7.5 Hz, 2H), 2.03 (m, 4H), 1.77 – 1.47 (m, 6H), 1.42 – 1.21 (m, 36H), 1.00 – 0.82 (m, 3H).

^{13}C NMR (100 MHz, Chloroform-*d*) δ 174.3, 174.1, 129.9, 129.8, 70.3, 65.2, 64.4, 63.3, 34.4, 34.2, 31.8, 29.74, 29.70, 29.65, 29.63, 29.58, 29.53, 29.45, 29.26, 29.25, 29.18, 29.14, 29.13, 29.11, 29.00, 28.7, 27.2, 27.1, 25.9, 25.0, 24.9, 22.7, 14.1.

rac-1,2-Di-((9Z)-hexadec-9-enoyl)-3-{16-{[(9Z)-hexadec-9-enoyl]oxy}hexadecanoyl}glycerol (TG-EST **4**)

N,*N*'-Dicyclohexylcarbodiimide (DCC, 0.13 g; 0.62 mmol) was added to a stirred solution of 1-acyl glycerol **27** (0.09 g; 0.15 mmol), (*Z*)-hexadec-9-enoic acid (0.16 g; 0.62 mmol) and DMAP (0.008 g; 0.061 mmol) in DCM (5 mL). After stirring at room temperature for 16 h the reaction mixture was evaporated and the residual oil was purified by column chromatography (silica gel, eluent EtOAc:cyclohexane/1:12) to give 0.15 g (91%) of desired product TG-EST **4**.

HR-ESI-MS calculated for $C_{67}H_{122}O_8Na$ $[M + Na]^+$ *m/z* 1077.9037, found *m/z* 1077.9029.

^{1}H NMR (400 MHz, Chloroform-*d*) δ 5.44 – 5.32 (m, 6H), 5.29 (ddd, *J* = 6.0, 4.3, 1.7 Hz, 1H), 4.32 (dd, *J* = 11.9, 4.3 Hz, 2H), 4.17 (dd, *J* = 11.9, 6.0 Hz, 2H), 4.07 (t, *J* = 6.8 Hz, 2H), 2.45 – 2.27 (m, 8H), 2.03 (m, 10H), 1.76 – 1.57 (m, 12H), 1.41 – 1.19 (m, 70H), 0.97 – 0.84 (m, 9H).

^{13}C NMR (100 MHz, Chloroform-*d*) δ 174.0, 173.3, 173.2, 172.8, 130.03, 130.02, 129.9, 129.76, 129.72, 129.69, 68.9, 64.4, 62.1, 34.4, 34.21, 34.06, 34.04, 31.8, 29.74, 29.73, 29.70, 29.66, 29.62, 29.57, 29.51, 29.30, 29.21, 29.18, 29.15, 29.12, 29.10, 29.06, 29.00, 28.7, 27.24, 27.18, 25.9, 25.0, 24.90, 24.88, 24.85, 22.7, 14.1.

1.5 Preparation of TG-EST 5

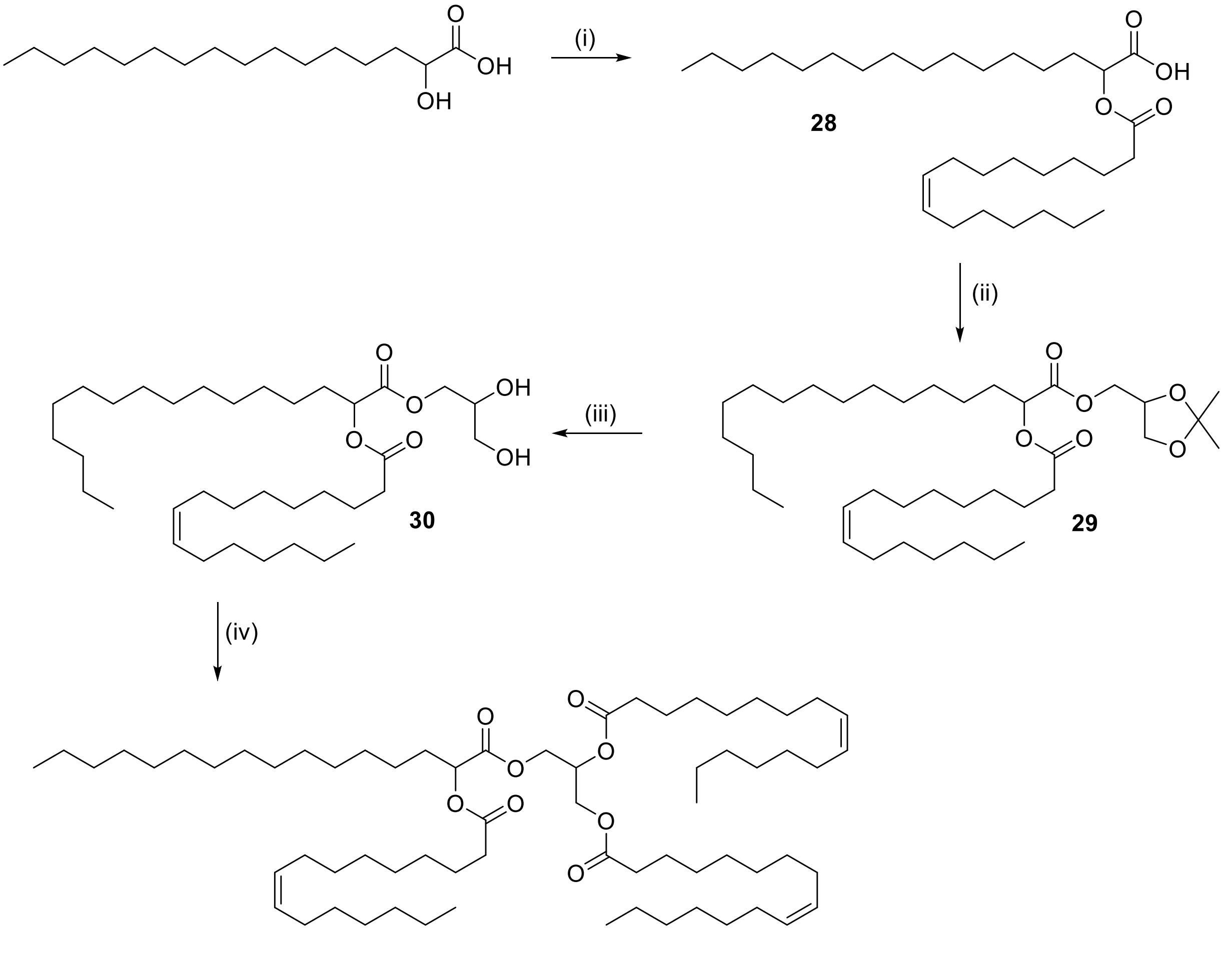

TG-EST **5**

Scheme S5. Preparation of compound TG-EST **5**. *Reaction and conditions:* (i) (*Z*)-hexadec-9-enoyl chloride, pyridine, DCM; (ii) DCC, 2,3-*O*-isopropylidene glycerol, DMAP, DCM; (iii) Amberlyst 15 H-form, methanol, DCM; (iv) DCC, (*Z*)-hexadec-9-enoic acid, DMAP, DCM.

2-{[(9Z)-Hexadec-9-enoyl]oxy}hexadecanoic acid (**28**)

A mixture of (*Z*)-hexadec-9-enoic acid (0.25 g; 1.0 mmol) and thionyl chloride (1 mL) in toluene (5 mL) was stirred at 100 °C for one hour in a sealed ampoule. Then was the mixture evaporated under reduced pressure and the residue was twice evaporated with toluene. Thus formed (Z)-hexadec-9-enoyl chloride was diluted with DCM (3 mL) and immediately was added dropwise to a stirred solution of 2-hydroxyhexadecanoic acid (0.27 g; 1.0 mmol) and pyridine (1 mL) in DCM (5 mL) at 0 °C. After stirring at room temperature for 16 hours, the mixture was diluted with DCM (15 mL), washed with 10% aqueous HCl (20 mL), water (10 mL) and brine (20 mL). The solvent was evaporated in vacuo and the oily residue was purified by column chromatography (silica gel, eluent EtOAc:cyclohexane/1:4) to give 0.37 g (72%) of 2-(acyl)hexadecanoic acid **28**.

HR-ESI-MS calculated for $C_{32}H_{59}O_4$ [M – H]$^-$ *m/z* 507.4419, found *m/z* 507.4417.

^{1}H NMR (400 MHz, Chloroform-*d*) δ 5.41 – 5.33 (m, 2H), 5.05 (dd, *J* = 7.0, 5.7 Hz, 1H), 2.42 (m, 2H), 2.10 – 1.97 (m, 4H), 1.89 (m, 2H), 1.68 (m, 2H), 1.55 – 1.19 (m, 40H), 0.96 – 0.86 (m, 6H).

^{13}C NMR (100 MHz, Chloroform-*d*) δ 174.7, 173.4, 130.00, 129.7, 71.5, 33.9, 31.9, 31.8, 30.9, 29.75, 29.70, 29.67, 29.63, 29.54, 29.37, 29.16, 29.11, 29.04, 29.00, 27.24, 27.17, 25.1, 24.8, 22.7, 22.7, 14.14, 14.12.

(2,2-Dimethyl-1,3-dioxolan-4-yl)methyl 2-{[(9Z)-hexadec-9-enoyl]oxy}hexadecanoate (**29**)

N,*N*'-Dicyclohexylcarbodiimide (DCC, 0.17 g; 0.82 mmol) was added to a stirred solution of 2,3-*O*-isopropylidene glycerol (0.14 g; 1.0 mmol), acid **28** (0.35 g; 0.67 mmol) and DMAP (0.01 g; 0.08 mmol) in DCM (5 mL). After stirring at room temperature for 16 h the reaction mixture was evaporated and the residual oil was purified by column chromatography (silica gel, eluent EtOAc:cyclohexane/1:6) to give 0.40 g (93%) of desired intermediate **29**.

HR-ESI-MS calculated for $C_{38}H_{70}O_6Na$ [M + Na]$^+$ *m/z* 645.5070, found *m/z* 645.5062.

^{1}H NMR (400 MHz, Chloroform-*d*) δ 5.45 – 5.28 (m, 2H), 5.00 (td, *J* = 6.4, 1.7 Hz, 1H), 4.38 – 4.28 (m, 1H), 4.21 (m, 2H), 4.09 (m, 1H), 3.77 (m, 1H), 3.27 – 3.14 (m, 1H), 2.50 – 2.34 (m, 2H), 2.03 (m, 4H), 1.99 – 1.88 (m, 2H), 1.88 – 1.80 (m, 2H), 1.80 – 1.70 (m, 2H), 1.61– 1.58 (m, 6H), 1.45 (s, 3H), 1.38 (s, 3H), 1.42 – 1.19 (m, 36H), 1.00 – 0.85 (m, 6H).

^{13}C NMR (100 MHz, Chloroform-*d*) δ 173.4, 170.4, 130.0, 129.7, 109.84, 109.78, 73.37, 73.31, 72.13, 72.08, 66.32, 66.29, 65.1, 64.9, 55.8, 34.9, 33.9, 31.94, 31.79, 31.10, 29.74, 29.71, 29.67, 29.63, 29.54, 29.37, 29.18, 29.14, 29.12, 29.07, 29.00, 27.23, 27.18, 26.71, 26.68, 25.46, 25.37, 25.33, 25.16, 24.83, 24.81, 24.7, 22.70, 22.67, 14.13, 14.12.

2,3-Dihydroxypropyl 2-{[(9Z)-hexadec-9-enoyl]oxy}hexadecanoate (**30**)

Acetonide **29** (0.17 g; 0.27 mmol) and Amberlyst 15 H-form (0.05 g) were stirred in a mixture of methanol (5 mL) and DCM (2 mL) for 3 days. Then was catalyst filtered off, the filtrate was evaporated and the residue was purified by column chromatography (eluent EtOAc:cyclohexane/1:1) to give 0.07 g (44%) of 1-acylglycerol **30**.

HR-ESI-MS calculated for $C_{35}H_{66}O_6Na$ [M + Na]$^+$ *m/z* 605.4757, found *m/z* 605.4749.

^{1}H NMR (400 MHz, Chloroform-*d*) δ 5.46 – 5.20 (m, 2H), 4.93 (t, *J* = 6.5 Hz, 1H), 4.57 – 4.09 (m, 2H), 4.10 – 3.86 (m, 1H), 3.82 – 3.68 (m, 1H), 3.63 (m, 1H), 2.41 (td, *J* = 7.4, 1.3 Hz, 2H), 2.03 (m, 4H), 1.86 (m, 2H), 1.66 (m, 6H), 1.44 – 1.16 (m, 36H), 1.00 – 0.81 (m, 6H).

^{13}C NMR (100 MHz, Chloroform-*d*) δ 173.9, 170.9, 130.0, 129.7, 72.58, 72.56, 69.90, 65.93, 65.74, 63.08, 63.05, 33.9, 31.94, 31.79, 31.0, 29.74, 29.70, 29.67, 29.62, 29.53, 29.37, 29.15, 29.11, 29.05, 29.00, 27.24, 27.17, 25.2, 24.8, 14.14, 14.12.

rac-1,2-Di-((9Z)-hexadec-9-enoyl)-3-{2-{[(9Z)-hexadec-9-enoyl]oxy}hexadecanoyl}glycerol (TG-EST **5**)

N,*N*'-Dicyclohexylcarbodiimide (DCC, 0.095 g; 0.44 mmol) was added to a stirred solution of 1-acyl glycerol **30** (0.065 g; 0.11 mmol), (*Z*)-hexadec-9-enoic acid (0.11 g; 0.44 mmol) and DMAP (0.006 g; 0.04 mmol) in DCM (5 mL). After stirring at room temperature for 16 h the reaction mixture was evaporated and the residual oil was purified by column chromatography (silica gel, eluent EtOAc:cyclohexane/1:10) to give 0.11 g (94%) of desired product TG-EST **5**.

HR-ESI-MS calculated for $C_{67}H_{122}O_8Na$ $[M + Na]^+$ *m/z* 1077.9037, found *m/z* 1077.9024.

^{1}H NMR (400 MHz, Chloroform-*d*) δ 5.48 – 5.32 (m, 6H), 5.29 (td, *J* = 4.3, 1.6 Hz, 1H), 4.98 (dt, *J* = 7.8, 6.4 Hz, 1H), 4.48 – 4.26 (m, 3H), 4.23 (dd, *J* = 11.9, 5.9 Hz, 1H), 4.15 (ddd, *J* = 11.9, 5.9, 3.4 Hz, 1H), 2.43 – 2.36 (m, 2H), 2.37 – 2.25 (m, 4H), 2.12 – 1.90 (m, 12H), 1.88 – 1.77 (m, 2H), 1.74 – 1.53 (m, 12H), 1.42 – 1.23 (m, 66H), 0.96 – 0.86 (m, 12H).

^{13}C NMR (100 MHz, Chloroform-*d*) δ 173.33, 173.19, 172.8, 170.1, 130.06, 130.01, 129.74, 129.72, 129.70, 71.9, 68.6, 34.13, 34.01, 33.9, 31.94, 31.80, 29.74, 29.72, 29.68, 29.66, 29.59, 29.43, 29.38, 29.23, 29.19, 29.16, 29.13, 29.11, 29.09, 29.00, 27.24, 27.19, 24.8, 22.71, 22.67, 14.1.

1.6 Preparation of TG-EST 6

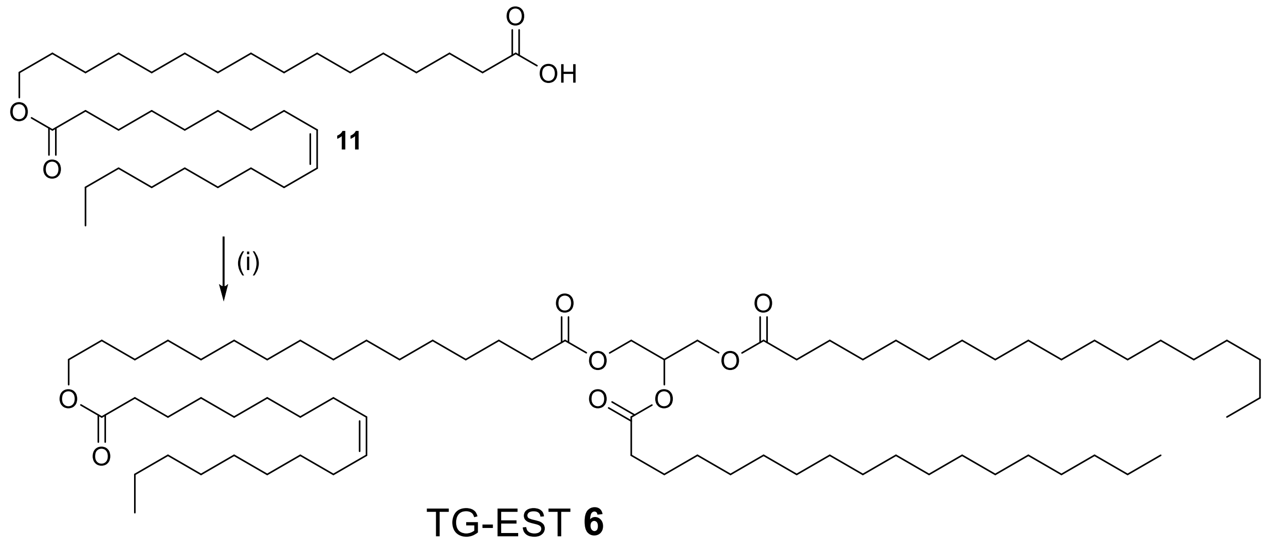

Scheme S6. Preparation of compound TG-EST **6**. *Reaction and conditions:* (i) DCC, *rac*-1,2-distearoylglycerol, DMAP, DCM.

rac-1,2-Dioctadecanoyl-3-{16-{[(9Z)-octadec-9-enoyl]oxy}hexadecanoyl}glycerol
(TG-EST **6**)

N,*N*'-Dicyclohexylcarbodiimide (DCC, 0.04 g; 0.20 mmol) was added to a stirred solution of intermediate **11** (0.10 g; 0.186 mmol), *rac*-1,2-distearoylglycerol (0.13 g; 0.20 mmol) and DMAP (0.002 g; 0.016 mmol) in DCM (3 mL). After stirring at room temperature for 16 h the reaction mixture was evaporated and the residual oil was purified by column chromatography (silica gel, eluent EtOAc:cyclohexane/1:9) to give 0.15 g (70%) of desired product TG-EST **6**.

^{1}H NMR (400 MHz, Chloroform-*d*) δ 5.49 – 5.34 (m, 2H), 5.29 (m, 1H), 4.32 (dd, *J* = 11.9, 4.2 Hz, 2H), 4.17 (dd, *J* = 11.9, 6.0 Hz, 2H), 4.07 (t, *J* = 6.8 Hz, 2H), 2.40 – 2.18 (m, 8H), 2.03 (m, 4H), 1.75 – 1.57 (m, 16H), 1.30 (m, 94H), 1.05 – 0.81 (m, 9H).

^{13}C NMR (100 MHz, Chloroform-*d*) δ 174.0, 173.3, 172.9, 130.0, 129.8, 68.8, 64.4, 62.1, 55.8, 34.9, 34.4, 34.2, 34.1, 31.9, 31.9, 29.8, 29.7, 29.7, 29.68, 29.63, 29.57, 29.54, 29.52, 29.50, 29.38, 29.33, 29.30, 29.19, 29.15, 29.13, 29.10, 28.7, 27.2, 27.1, 25.9, 25.5, 25.0, 24.9, 24.8, 24.7, 22.7, 14.1.

1.7 Preparation of TG-EST 7

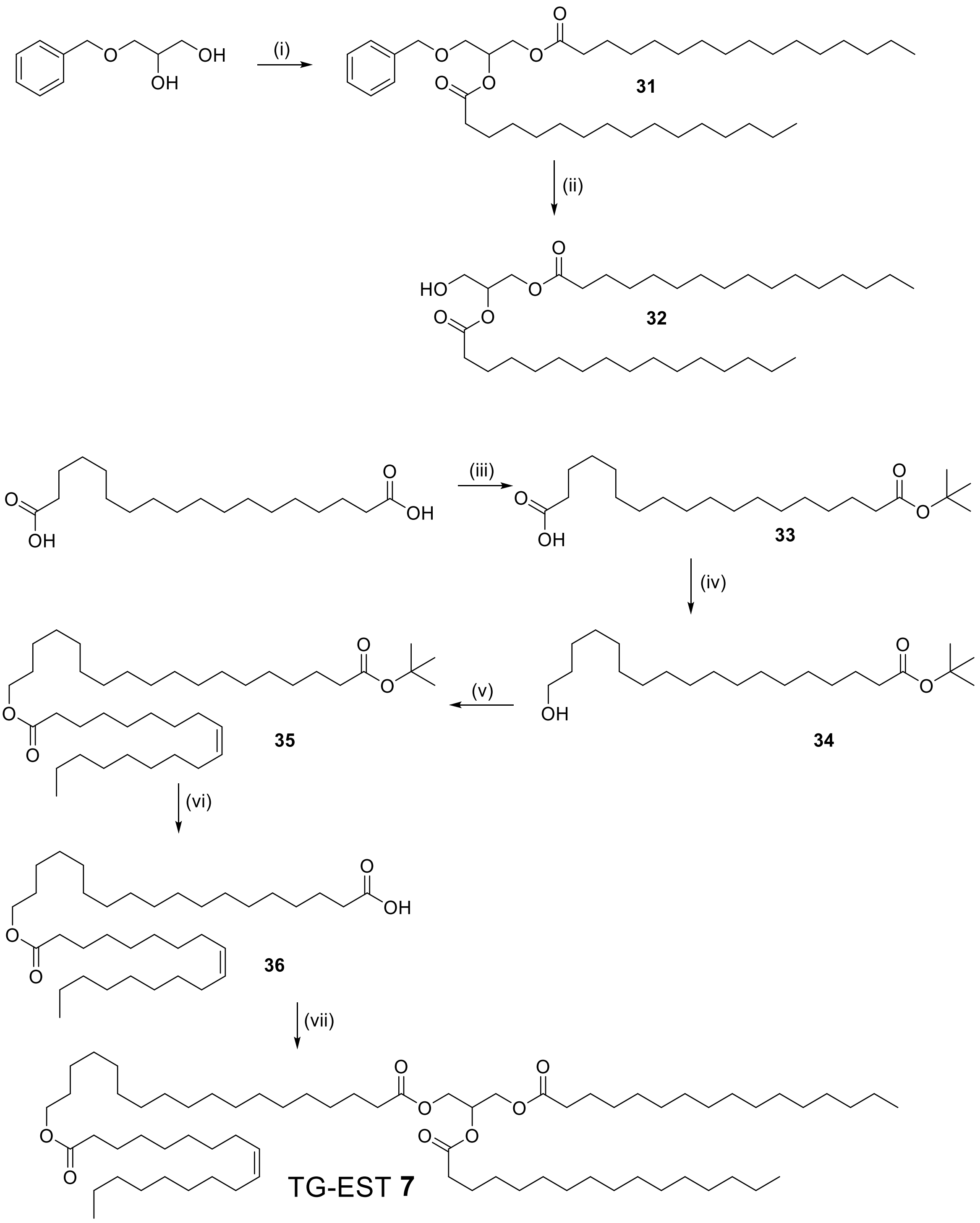

Scheme S7. Preparation of TG-EST **7**. *Reaction and conditions:* (i) DCC, hexadecanoic acid, DMAP, DCM; (ii) H_2, Pd/C, DCM, EtOH; (iii) *N*,*N*-dimethylformamide di-*tert*-butylacetal, 110 °C, toluene; (iv) $BH_3.Me_2S$; (v) DCC, oleic acid, DMAP, DCM; (vi) TFA, DCM; (vii) DCC, compound **32**, DMAP, DCM.

rac-1-Benzyl-2,3-dihexadecanoylglycerol (**31**)

N,*N*'-Dicyclohexylcarbodiimide (DCC, 0.90 g; 4.4 mmol) was added to a stirred solution of *rac*-3-benzyloxy-1,2-propanediol (0.20 g; 1.1 mmol), hexadecanoic acid (1.12 g; 4.4 mmol) and DMAP (0.014 g; 0.11 mmol) in DCM (10 mL). After stirring at room temperature for 16 h the reaction mixture was evaporated and the residual oil was purified by column chromatography (silica gel, eluent EtOAc:cyclohexane/1:8) to give 0.62 g (85%) of desired intermediate **31**.

HR-ESI-MS calculated for $C_{42}H_{74}O_5Na$ $[M + Na]^+$ *m/z* 681.5434, found *m/z* 681.5428.

^{1}H NMR (400 MHz, Chloroform-*d*) δ 7.48 – 7.29 (m, 5H), 5.26 (ddd, *J* = 6.5, 3.3, 1.4 Hz, 1H), 4.72 – 4.44 (m, 2H), 4.37 (dd, *J* = 11.9, 3.8 Hz,1H), 4.21 (dd, *J* = 11.9, 6.4 Hz, 1H), 3.61 (dd, *J* = 5.1, 1.2 Hz, 2H), 2.34 (t, *J* = 7.5 Hz, 2H), 2.30 (t, *J* = 7.5 Hz, 2H), 1.64 (m, 6H), 1.28 (d, *J* = 1.6 Hz, 52H), 0.98 – 0.79 (m, 6H).

^{13}C NMR (100 MHz, Chloroform-*d*) δ 173.4, 173.1, 169.6, 137.7, 128.4, 127.8, 127.6, 73.3, 70.0, 68.3, 62.7, 35.3, 34.4, 34.1, 31.9, 29.72, 29.68, 29.65, 29.58, 29.51, 29.41, 29.38, 29.31, 29.21, 29.14, 29.11, 28.9, 24.9, 24.8, 24.3, 22.7, 14.1.

rac-1,2-dihexadecanoylglycerol (**32**)

Benzylether **31** (0.62 g, 0.94 mmol) and Pd/C catalyst (0.06 g, 20 wt%) were suspended in a mixture of ethanol (15 mL) and DCM (8 mL). The flask was first purged with argon and then with hydrogen gas. To the flask was attached balloon filled with hydrogen and the mixture was intensively stirred for 4 h. The catalyst was filtered off and the filtrate was evaporated to dryness under reduced pressure. Column chromatography (silica gel, eluent EtOAc:cyclohexane/1:4) afforded 0.47 g (88%) of *rac*-1,2-palmitoylglycerol **32**.

HR-ESI-MS calculated for $C_{35}H_{68}O_5Na$ $[M + Na]^+$ *m/z* 591.4966, found *m/z* 591.4965.

^{1}H NMR (400 MHz, Chloroform-*d*) δ 5.25 – 4.93 (m, 1H), 4.34 (dd, *J* = 12.0, 4.5 Hz, 1H), 4.26 (dd, *J* = 12.0, 5.7 Hz, 1H), 3.86 – 3.71 (m, 2H), 2.53 – 2.17 (m, 4H), 1.65 (m, 6H), 1.28 (m, 52H), 0.90 (m, 3H).

^{13}C NMR (100 MHz, Chloroform-*d*) δ 173.8, 173.4, 72.1, 61.9, 61.6, 34.3, 34.1, 31.9, 29.71, 29.67, 29.63, 29.49, 29.38, 29.28, 29.14, 29.11, 25.0, 24.9, 22.7, 14.1.

18-Tert-butoxy-18-oxo-octadecanedioic acid (**33**)

Octadecanedioic acid (0.5 g; 1.59 mmol) was suspended in toluene (5 mL) and *N*,*N*-dimethylformamide di-*tert*-butylacetal (0.64 g; 3.18 mmol) was added. The reaction mixture was stirred at 60 °C in a sealed ampoule overnight. Then the mixture was allowed to cool down, the precipitated starting material was filtered off, the filtrate was evaporated and the residual oil was purified by silica gel chromatography (eluent EtOAc:cyclohexane/1:1) affording 0.16 g (27%) of mono-*tert*-butyl ester **33** as colorless oil.

^{1}H NMR (400 MHz, Chloroform-*d*) δ 2.37 (t, *J* = 7.5 Hz, 2H), 2.22 (t, *J* = 7.5 Hz, 2H), 1.75 – 1.53 (m, 4H), 1.47 (s, 9H), 1.38 – 1.21 (m, 24H).

^{13}C NMR (100 MHz, Chloroform-*d*) δ 178.3, 173.4, 79.9, 35.6, 33.8, 29.64, 29.62, 29.60, 29.57, 29.48, 29.42, 29.31, 29.23, 29.11, 29.06, 28.1, 25.1, 24.7.

Tert-butyl 18-hydroxyoctadecanoate (**34**)

Borane-dimethylsulfid complex (neat, 0.032 g; 0.42 mmol) was added to a solution of mono-*tert*-butyl ester **33** (0.13 g; 0.35 mmol) in THF (5 mL) at room temperature. The reaction mixture was stirred overnight and quenched with water at 0 °C. The mixture was extracted with EtOAc (3x5 mL). The combined organic layers were washed with brine and concentrated under reduced pressure. Column chromatography (silica gel, eluent EtOAc:cyclohexane/1:2) afforded 0.1 g (80%) of *tert*-butyl 18-hydroxyoctadecanoate **34** as a white solid.

HR-ESI-MS calculated for $C_{22}H_{44}O_3Na$ $[M + Na]^+$ *m/z* 379.3188, found *m/z* 379.3180.

^{1}H NMR (400 MHz, Chloroform-*d*) δ 3.66 (t, *J* = 6.6 Hz, 2H), 2.32 – 2.12 (m, 2H), 1.73 – 1.53 (m, 6H), 1.47 (s, 9H), 1.41 – 1.22 (m, 24H).

^{13}C NMR (100 MHz, Chloroform-*d*) δ 173.4, 79.9, 63.1, 35.6, 32.8, 29.67, 29.65, 29.62, 29.60, 29.49, 29.44, 29.32, 29.11, 28.1, 25.7, 25.1.

Tert-butyl 18-{[(9Z)-octadec-9-enoyl]oxy}octadecanoate (**35**)

N,*N*'-Dicyclohexylcarbodiimide (DCC, 0.17 g; 0.84 mmol) was added to a stirred solution of *tert*-butyl 18-hydroxyoctadecanoate **34** (0.20 g; 0.56 mmol), oleic acid (0.24 g; 0.84 mmol) and DMAP (0.007 g; 0.056 mmol) in DCM (5 mL). After stirring at room temperature for 16 h the reaction mixture was evaporated and the residual oil was purified by column chromatography (silica gel, eluent EtOAc:cyclohexane/1:8) to give 0.31 g (89%) of desired intermediate **35**.

HR-ESI-MS calculated for $C_{40}H_{76}O_4Na$ $[M + Na]^+$ *m/z* 643.5641, found *m/z* 643.5634.

^{1}H NMR (400 MHz, Chloroform-*d*) δ 5.50 – 5.14 (m, 2H), 4.08 (t, *J* = 6.7 Hz, 2H), 2.31 (t, *J* = 7.5 Hz, 2H), 2.22 (t, *J* = 7.5 Hz, 2H), 2.12 – 1.93 (m, 2H), 1.80 – 1.57 (m, 6H), 1.47 (s, 9H), 1.41 – 1.22 (m, 48H), 1.03 – 0.79 (m, 3H).

^{13}C NMR (100 MHz, Chloroform-*d*) δ 174.0, 173.4, 130.0, 129.7, 79.9, 64.4, 35.6, 34.4, 31.9, 29.78, 29.70, 29.67, 29.63, 29.60, 29.55, 29.54, 29.50, 29.33, 29.28, 29.19, 29.15, 29.12, 28.7, 28.1, 27.2, 27.1, 25.9, 25.1, 25.0, 22.7, 14.1.

18-{[(9Z)-Octadec-9-enoyl]oxy}octadecanoic acid (**36**)

To a solution of *tert*-butyl ester **35** (0.3 g; 0.48 mmol) in DCM (5 mL) placed in an ice bath was added dropwise neat trifluoroacetic acid (2 mL) and the reaction mixture was stirred at room temperature for 3 h. Then was the mixture evaporated on rotovap, the residue was twice evaporated with toluene and purified by column chromatography (silica gel, eluent EtOAc:cyclohexane/1:4) to give 0.25 g (92%) of acid **36**.

HR-ESI-MS calculated for $C_{36}H_{68}O_4Na$ $[M + Na]^+$ *m/z* 587.5015, found *m/z* 587.5010.

^{1}H NMR (400 MHz, Chloroform-*d*) δ 5.45 – 5.25 (m, 2H), 4.08 (t, *J* = 6.7 Hz, 2H), 2.37 (t, *J* = 7.5 Hz, 2H), 2.31 (t, *J* = 7.6 Hz, 2H), 2.03 (m, 4H), 1.64 (m, 6H), 1.46 – 1.23 (m, 48H), 0.90 (m, 3H).

^{13}C NMR (100 MHz, Chloroform-*d*) δ 174.0, 130.0, 129.7, 64.4, 34.4, 33.7, 31.9, 29.78, 29.71, 29.68, 29.65, 29.59, 29.54, 29.44, 29.34, 29.27, 29.25, 29.19, 29.15, 29.12, 29.07, 28.7, 27.3, 27.2, 25.9, 25.0, 24.7, 22.7, 14.1.

rac-1,2-Dihexadecanoyl-3-{18-{[(9Z)-octadec-9-enoyl]oxy}octadecanoyl}glycerol
(TG-EST **7**)

N,*N*'-Dicyclohexylcarbodiimide (DCC, 0.06 g; 0.29 mmol) was added to a stirred solution of intermediate **36** (0.15 g; 0.26 mmol), *rac*-1,2-dipalmitoylglycerol (0.16 g; 0.29 mmol) and DMAP (0.004 g; 0.029 mmol) in DCM (5 mL). After stirring at room temperature for 16 h the reaction mixture was evaporated. The residual oil was purified by column chromatography (silica gel, eluent EtOAc:cyclohexane/1:10) and subsequently it was subjected to the second column chromatography (eluent toluene) to give 0.08 g (27%) of desired product TG-EST **7**.

HR-ESI-MS calculated for $C_{71}H_{134}O_8Na$ $[M + Na]^+$ *m/z* 1137.9976, found *m/z* 1137.9961.

^{1}H NMR (400 MHz, Chloroform-*d*) δ 5.49 – 5.30 (m, 2H), 5.29 (ddd, *J* = 6.0, 4.2, 1.7 Hz, 1H), 4.31 (dd, *J* = 11.9, 4.3 Hz, 2H), 4.16 (dd, *J* = 11.9, 6.0 Hz, 2H), 4.07 (t, *J* = 6.7 Hz, 2H), 2.40 – 2.23 (m, 8H), 2.03 (m, 4H), 1.63 (m, 12H), 1.30 (m, 94H), 0.90 (m, 3H).

^{13}C NMR (100 MHz, Chloroform-*d*) δ 173.9, 173.3, 172.9, 129.9, 129.8, 68.8, 64.4, 62.1, 34.4, 34.2, 34.1, 31.94, 31.92, 29.78, 29.72, 29.70, 29.68, 29.66, 29.64, 29.62, 29.57, 29.54, 29.52, 29.50, 29.38, 29.33, 29.31, 29.29, 29.19, 29.15, 29.12, 29.09, 28.7, 27.2, 27.1, 25.9, 25.0, 24.9, 24.8, 22.7, 14.1.

1.8 Preparation of TG-EST 8

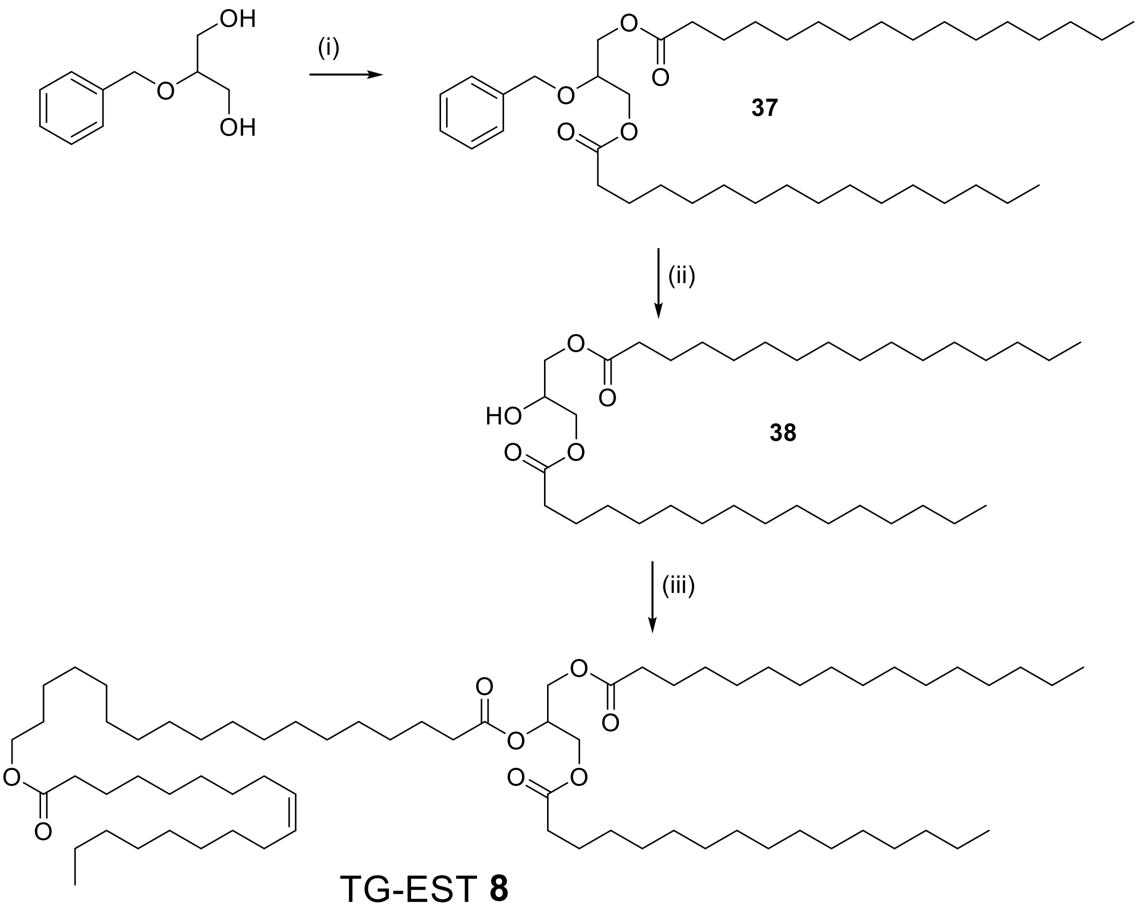

Scheme S8. Preparation of TG-EST **8**. *Reaction and conditions:* (i) DCC, hexadecanoic acid, DMAP, DCM; (ii) H_2, Pd/C, DCM, EtOH; (iii) DCC, compound **36**, DMAP, DCM.

2-Benzyl-1,3-dihexadecanoylglycerol (**37**)

N,*N*'-Dicyclohexylcarbodiimide (DCC, 0.90 g; 4.4 mmol) was added to a stirred solution of 2-benzyloxy-1,3-propanediol (0.20 g; 1.1 mmol), hexadecanoic acid (1.12 g; 4.4 mmol) and DMAP (0.014 g; 0.11 mmol) in DCM (10 mL). After stirring at room temperature for 16 h the reaction mixture was evaporated and the residual oil was purified by column chromatography (silica gel, eluent EtOAc:cyclohexane/1:8) to give 0.51 g (70%) of desired intermediate **37**.

HR-ESI-MS calculated for $C_{42}H_{74}O_5Na$ $[M + Na]^+$ *m/z* 681.5434, found *m/z* 681.5420.

^{1}H NMR (400 MHz, Chloroform-*d*) δ 7.37 (m, 5H), 4.68 (s, 2H), 4.27 (dd, *J* = 11.7, 4.7 Hz, 2H), 4.18 (dd, *J* = 11.7, 5.5 Hz, 2H), 3.84 (p, *J* = 5.2 Hz, 1H), 2.33 (t, *J* = 7.6 Hz, 4H), 1.84 – 1.53 (m, 6H), 1.28 (m, 52H), 0.90 (m, 3H).

^{13}C NMR (100 MHz, Chloroform-*d*) δ 173.6, 169.6, 137.8, 128.4, 127.8, 72.1, 62.9, 35.3, 34.2, 31.9, 29.70, 29.66, 29.58, 29.48, 29.38, 29.29, 29.16, 28.89, 24.91, 24.24, 22.7, 14.1.

1,3-Dihexadecanoylglycerol (**38**)

Benzylether **37** (0.50 g, 0.76 mmol) and Pd/C catalyst (0.05 g, 20 wt%) were suspended in a mixture of ethanol (15 mL) and DCM (8 mL). The flask was first purged with argon and then with hydrogen gas. To the flask was attached balloon filled with hydrogen and the mixture was intensively stirred for 5 h. The catalyst was filtered off and the filtrate was evaporated to dryness under reduced pressure. Column chromatography (silica gel, eluent EtOAc:cyclohexane/1:4) afforded 0.24 g (56%) of 1,3-palmitoylglycerol **38**.

HR-ESI-MS calculated for $C_{35}H_{68}O_5Na$ $[M + Na]^+$ *m/z* 591.4964, found *m/z* 591.4959.

^{1}H NMR (401 MHz, Chloroform-*d*) δ 4.33 – 4.01 (m, 5H), 2.37 (t, *J* = 7.6 Hz, 4H), 1.86 – 1.50 (m, 6H), 1.28 (s, 52H), 0.90 (m, 3H).

^{13}C NMR (101 MHz, Chloroform-*d*) δ 173.9, 68.4, 65.1, 34.1, 31.9, 29.7, 29.7, 29.7, 29.6, 29.5, 29.4, 29.3, 29.1, 24.9, 22.7, 14.1.

rac-1,3-Dihexadecanoyl-2-{18-{[(9Z)-octadec-9-enoyl]oxy}octadecanoyl}glycerol (TG-EST **8**)

N,*N*'-Dicyclohexylcarbodiimide (DCC, 0.06 g; 0.29 mmol) was added to a stirred solution of acid **36** (0.15 g; 0.26 mmol), 1,3-dipalmitoylglycerol **38** (0.16 g; 0.29 mmol) and DMAP (0.004 g; 0.029 mmol) in DCM (5 mL). After stirring at room temperature for 16 h the reaction mixture was evaporated. The residual oil was purified by column chromatography (silica gel, eluent EtOAc:cyclohexane/1:10) and subsequently it was subjected to the second column chromatography (eluent toluene) to give 0.10 g (34%) of desired product TG-EST **8**.

^{1}H NMR (400 MHz, Chloroform-*d*) δ 5.45 – 5.34 (m, 2H), 5.31 – 5.22 (m, 1H), 4.32 (dd, *J* = 11.9, 4.3 Hz, 2H), 4.17 (dd, *J* = 11.9, 6.0 Hz, 2H), 4.07 (t, *J* = 6.7 Hz, 2H), 2.47 – 2.20 (m, 8H), 2.08 – 1.95 (m, 4H), 1.80 – 1.56 (m, 6H), 1.49 – 1.20 (m, 98H), 1.05 – 0.85 (m, 3H).

^{13}C NMR (100 MHz, Chloroform-*d*) δ 173.9, 173.3, 172.9, 129.9, 129.7, 68.9, 64.4, 62.1, 34.4, 34.2, 34.1, 31.94, 31.92, 29.78, 29.74, 29.72, 29.70, 29.68, 29.64, 29.57, 29.54, 29.50, 29.38, 29.33, 29.29, 29.19, 29.15, 29.13, 29.11, 28.7, 27.23, 27.18, 25.9, 25.0, 24.92, 24.88, 22.7, 14.1.

1.9 Copies of ¹H-NMR and ¹³C-NMR spectra

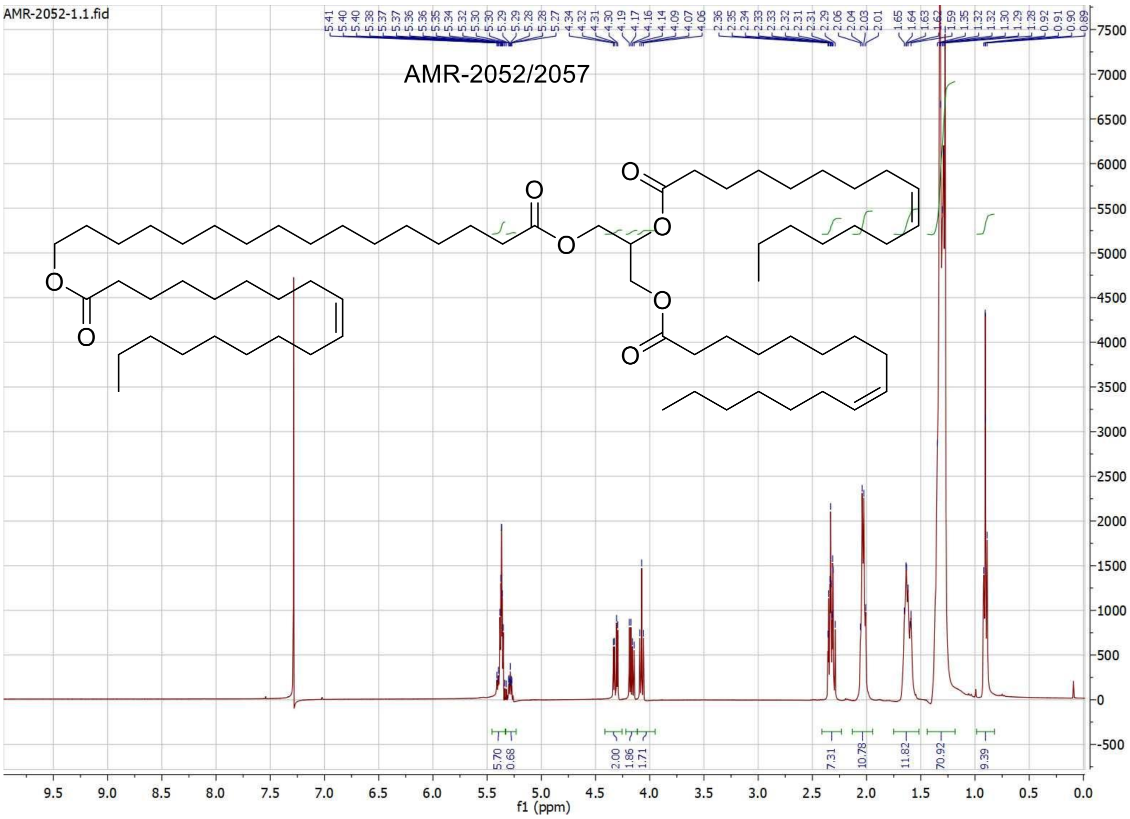

Figure S1. ¹H NMR spectrum of compound TG-EST **1**.

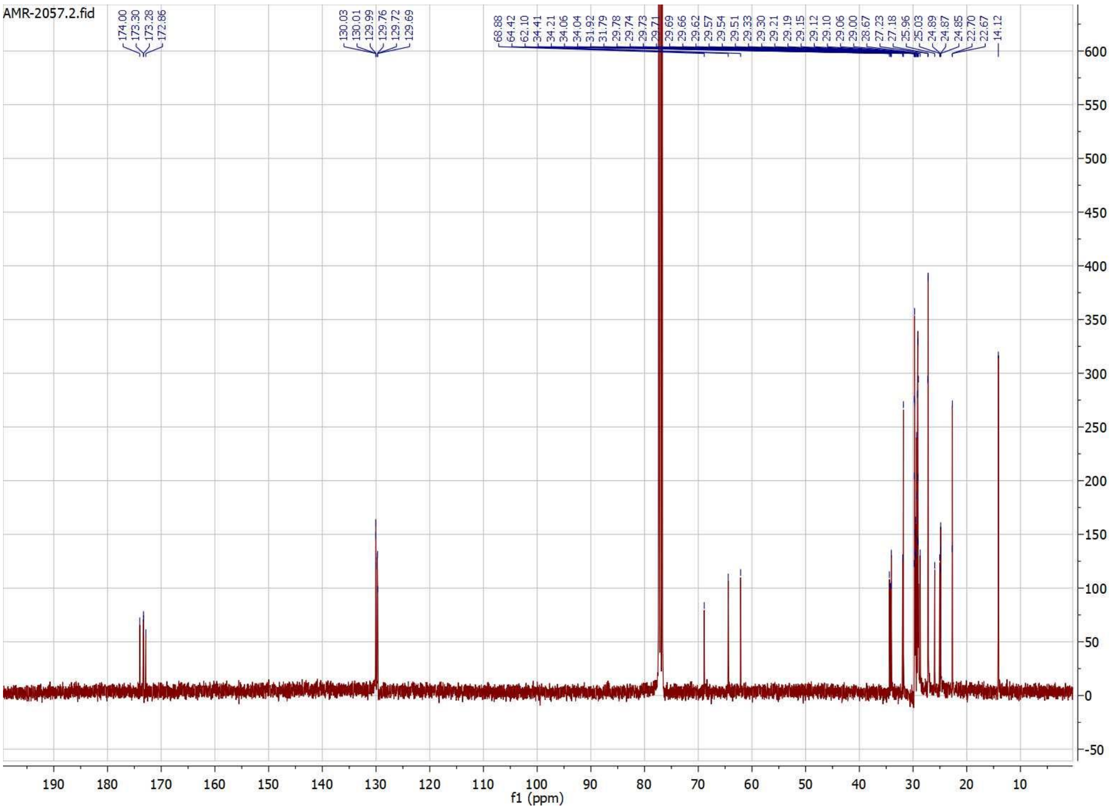

Figure S2. ¹³C NMR spectrum of TG-EST **1**.

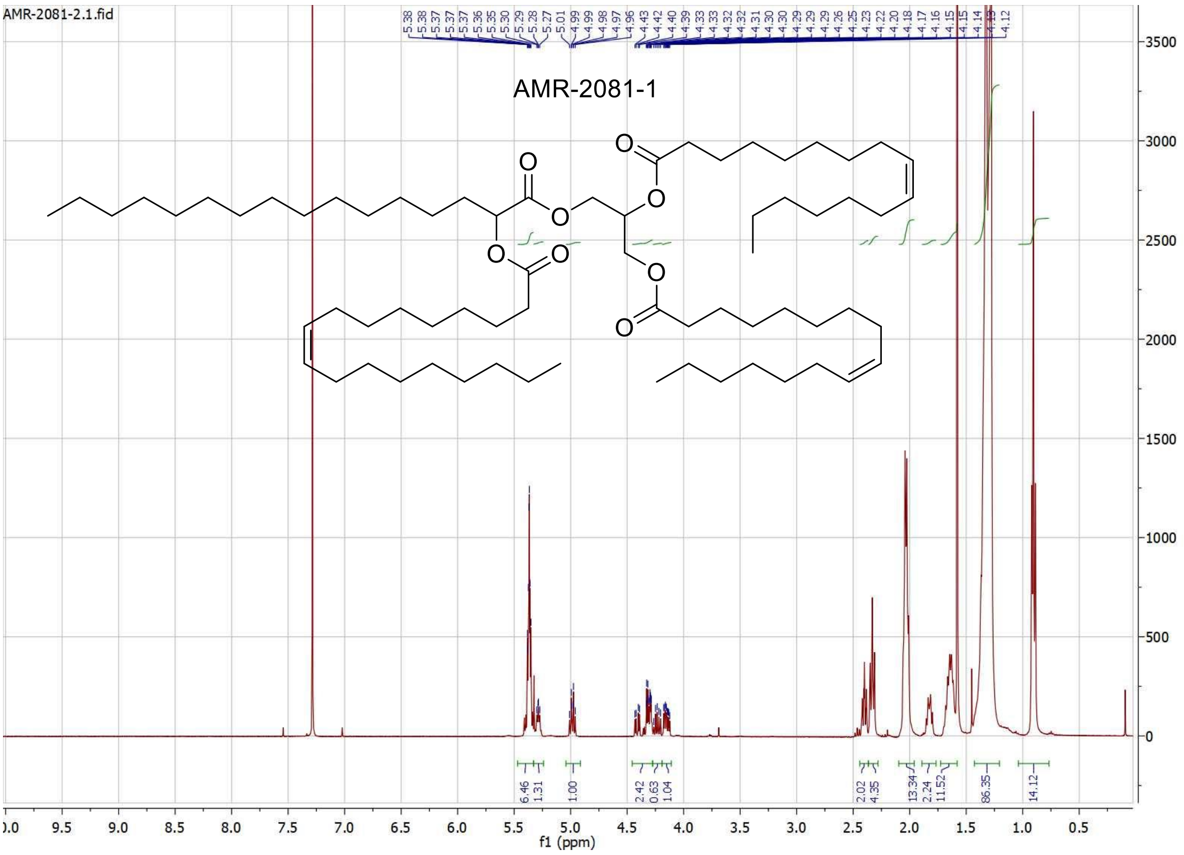

Figure S3. ^{1}H NMR spectrum of TG-EST **2**.

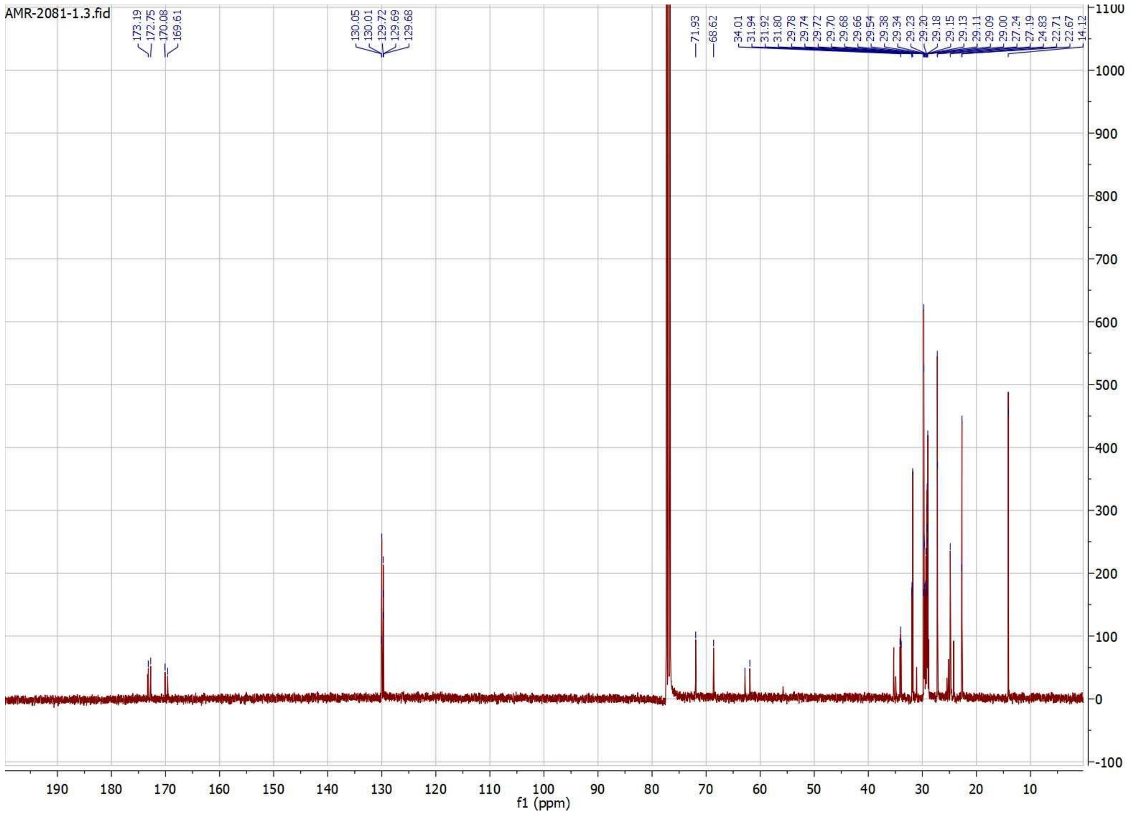

Figure S4. ^{13}C NMR spectrum of TG-EST **2**.

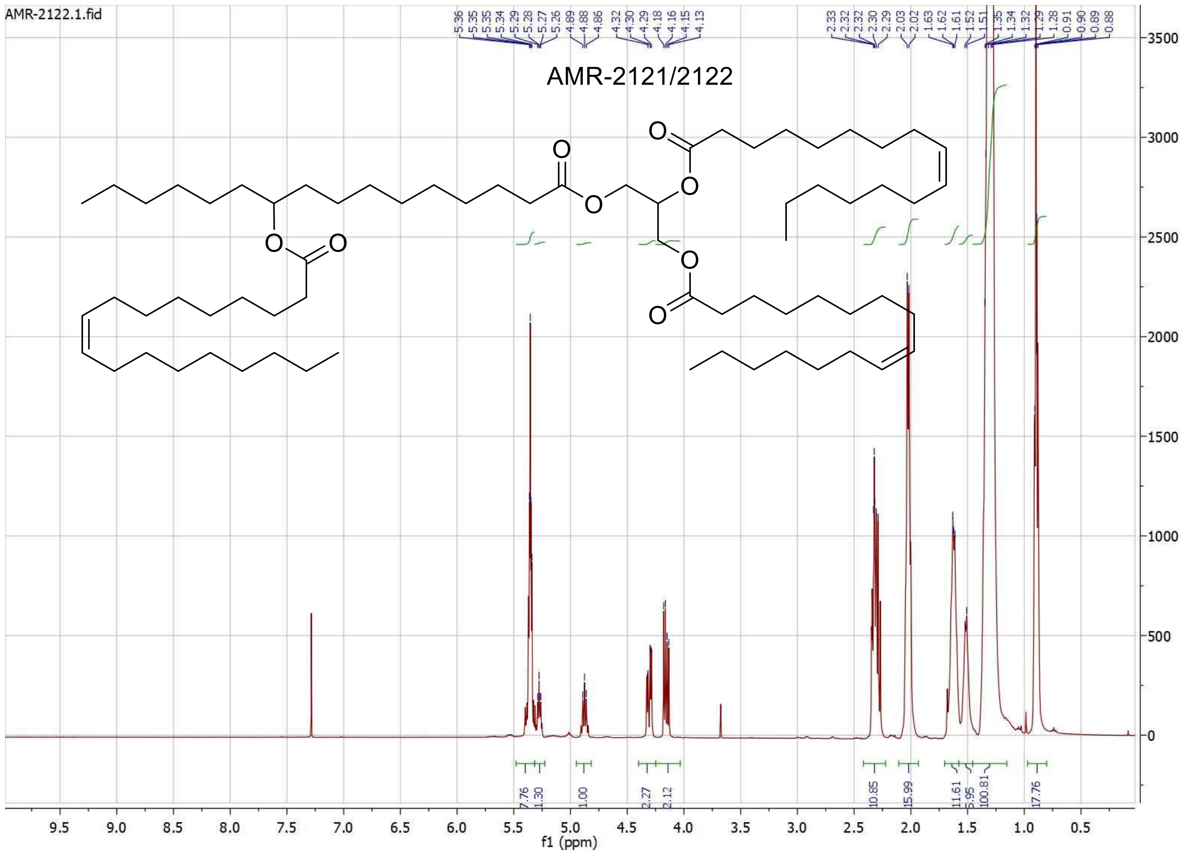

Figure S5. ^{1}H NMR spectrum of TG-EST **3**.

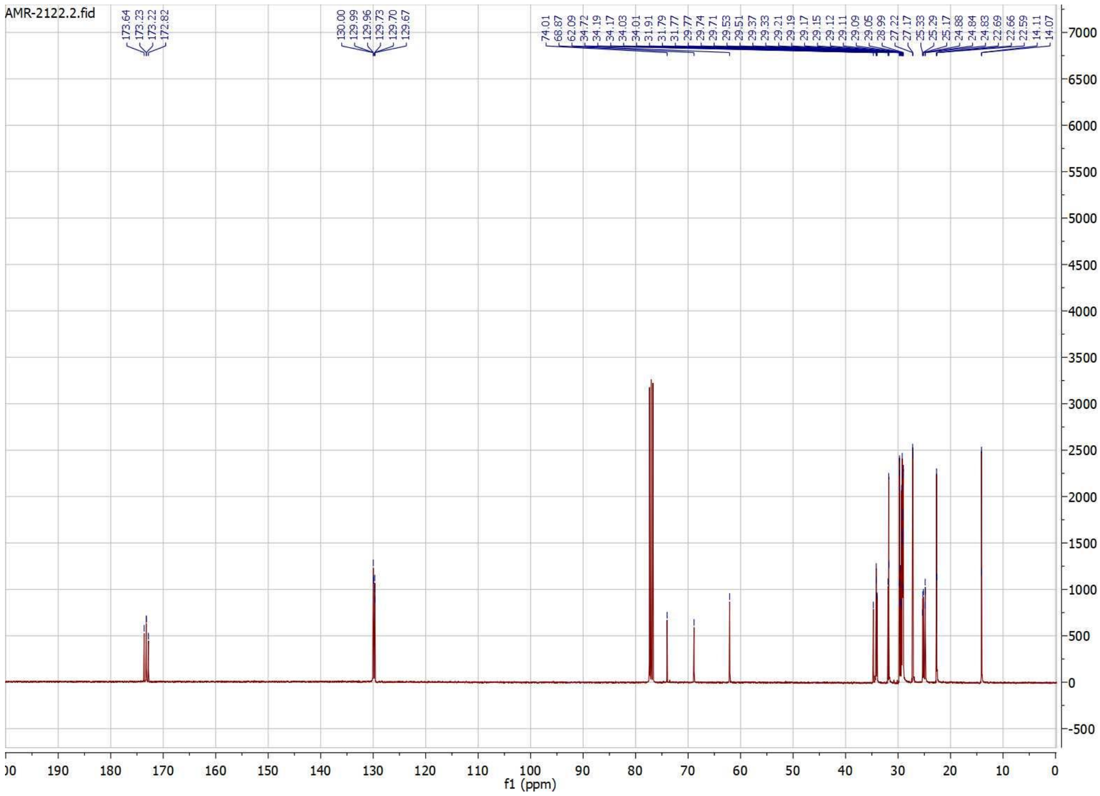

Figure S6. ^{13}C NMR spectrum of TG-EST **3**.

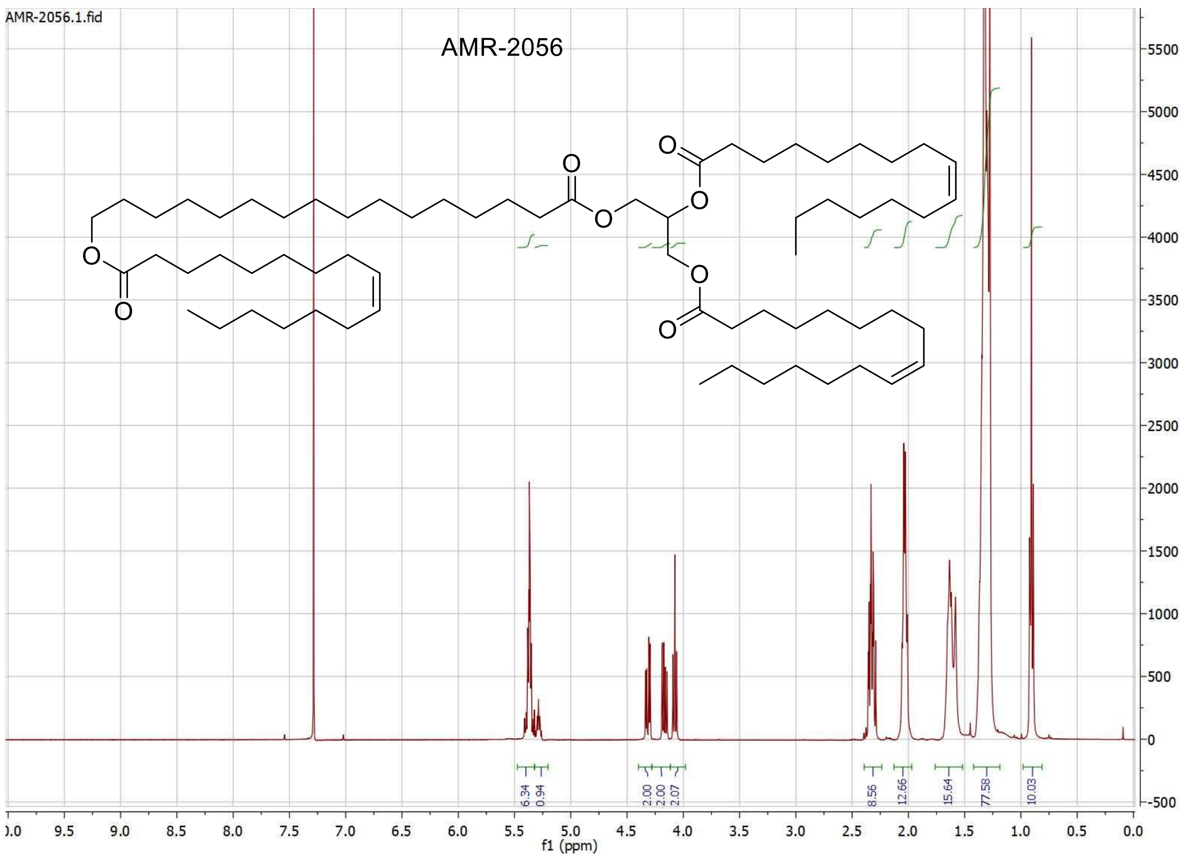

Figure S7. ^{1}H NMR spectrum of TG-EST **4**.

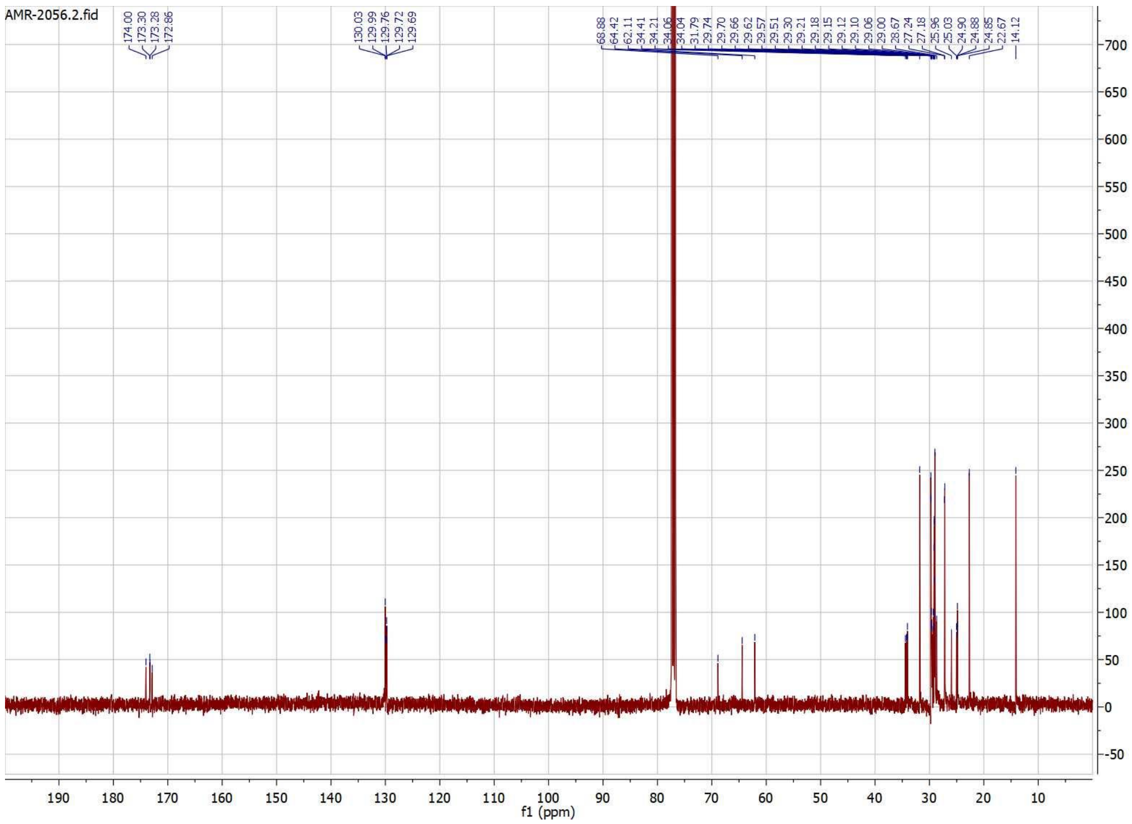

Figure S8. ^{13}C NMR spectrum of TG-EST **4**.

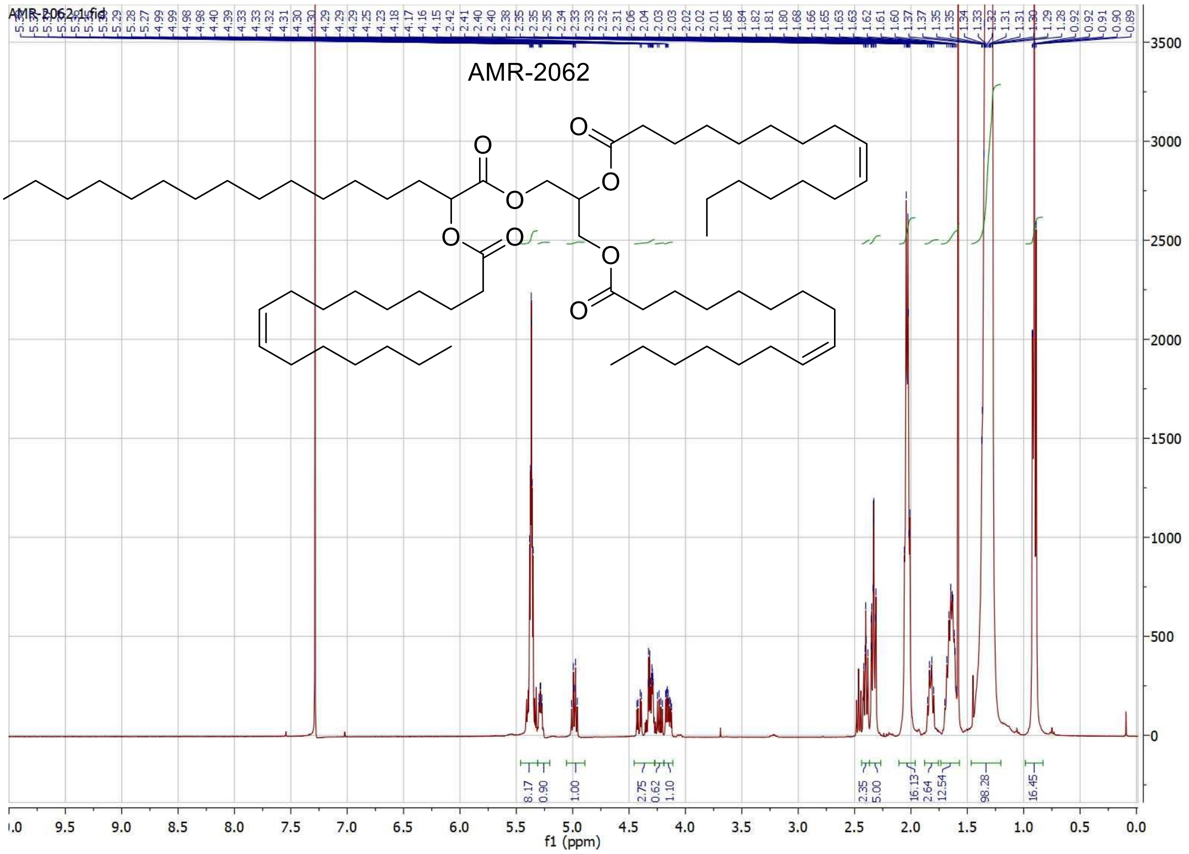

Figure S9. ^{1}H NMR spectrum of TG-EST **5**.

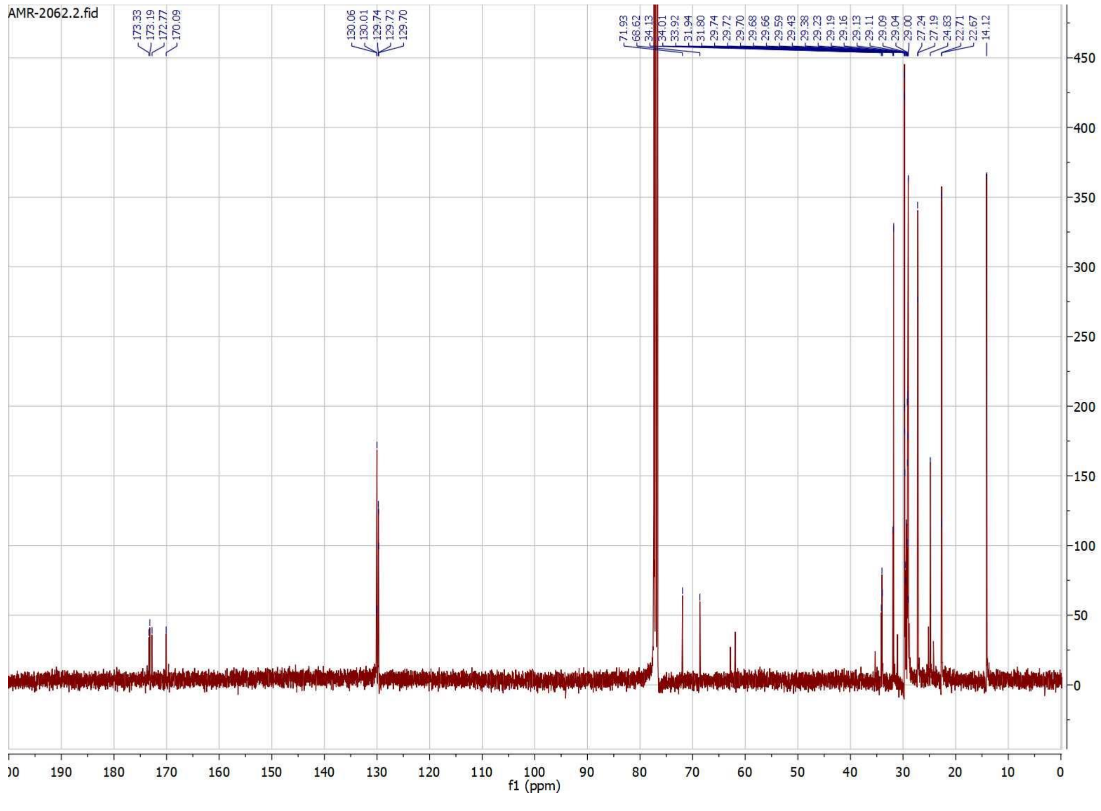

Figure S10. ^{13}C NMR spectrum of TG-EST **5**.

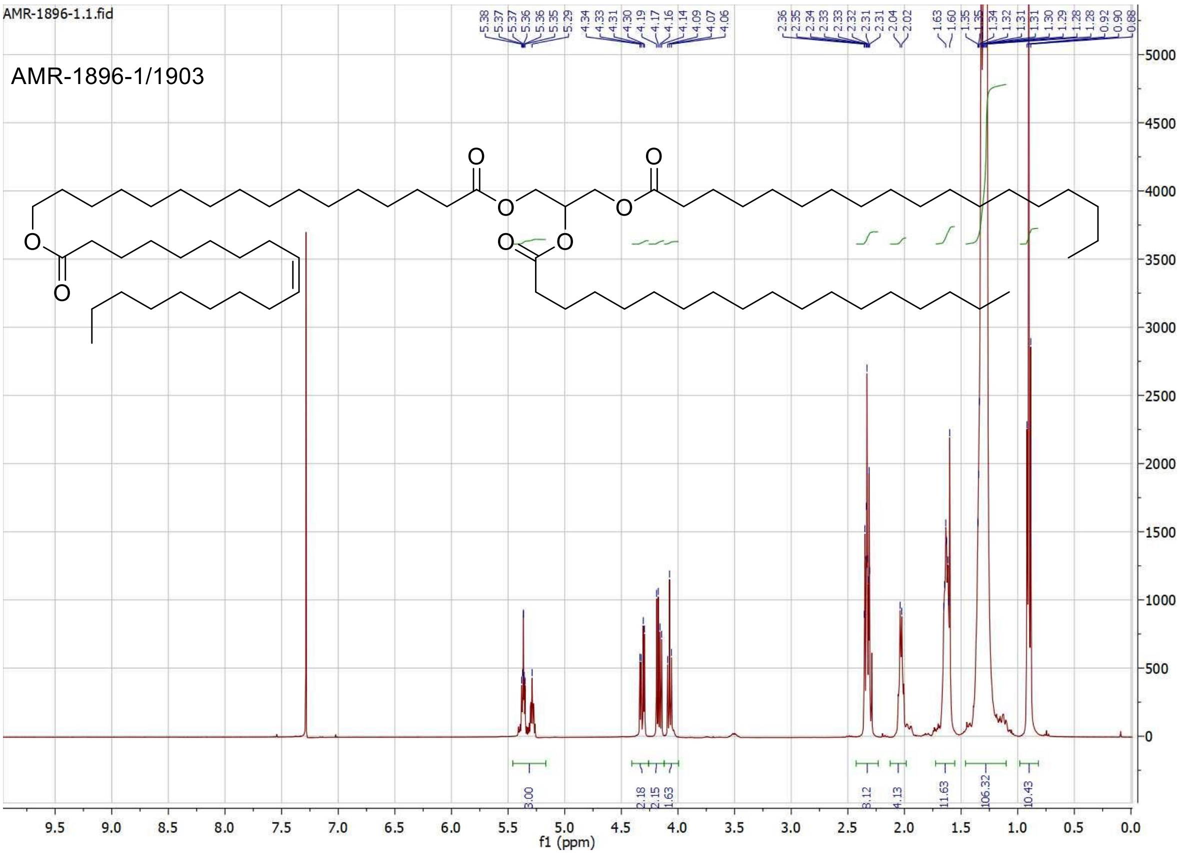

Figure S11. ^{1}H NMR spectrum of TG-EST **6**.

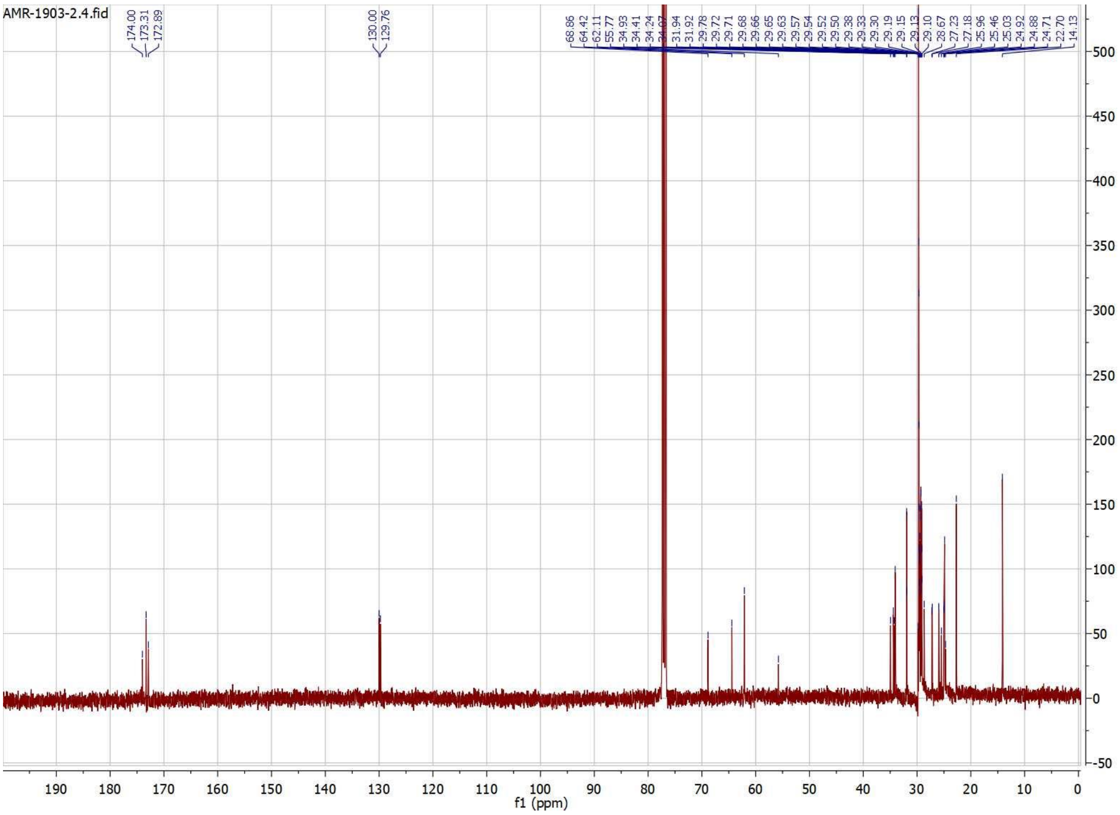

Figure S12. ^{13}C NMR spectrum of TG-EST **6**.

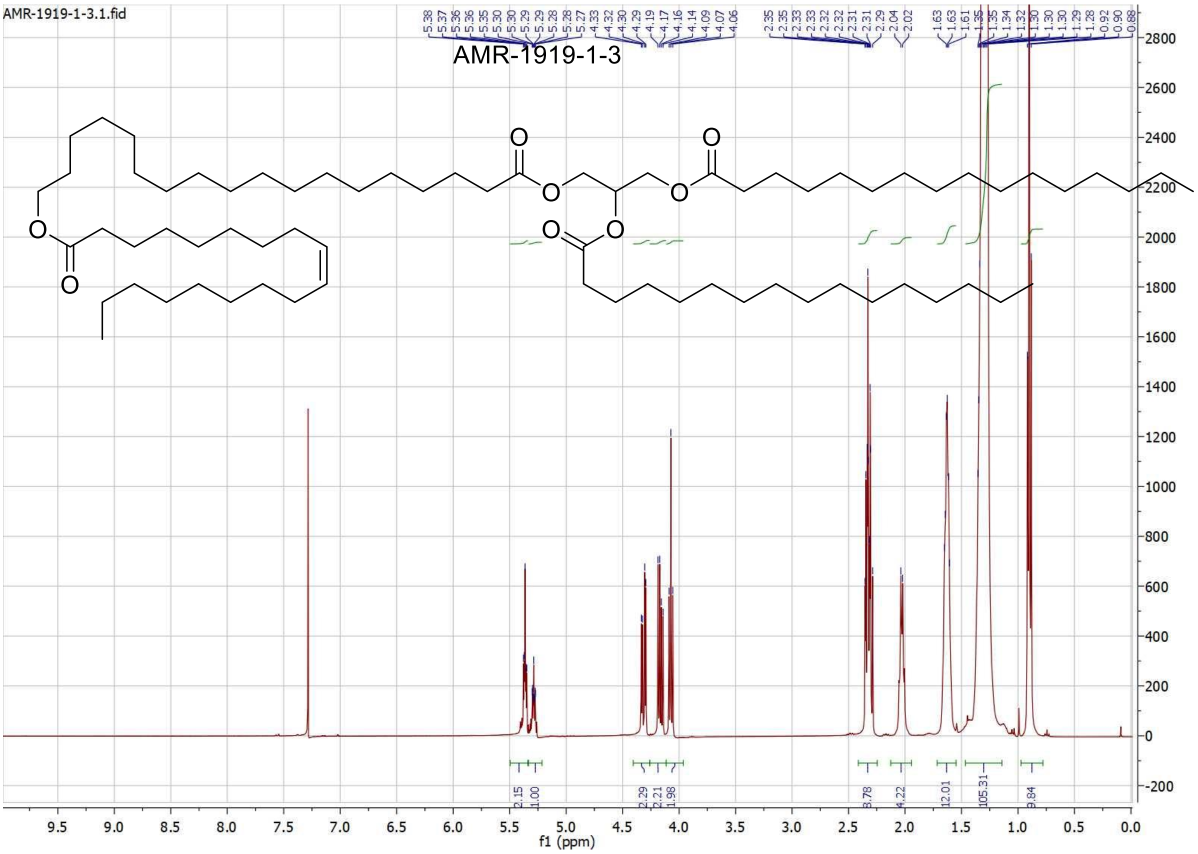

Figure S13. ^{1}H NMR spectrum of TG-EST **7**.

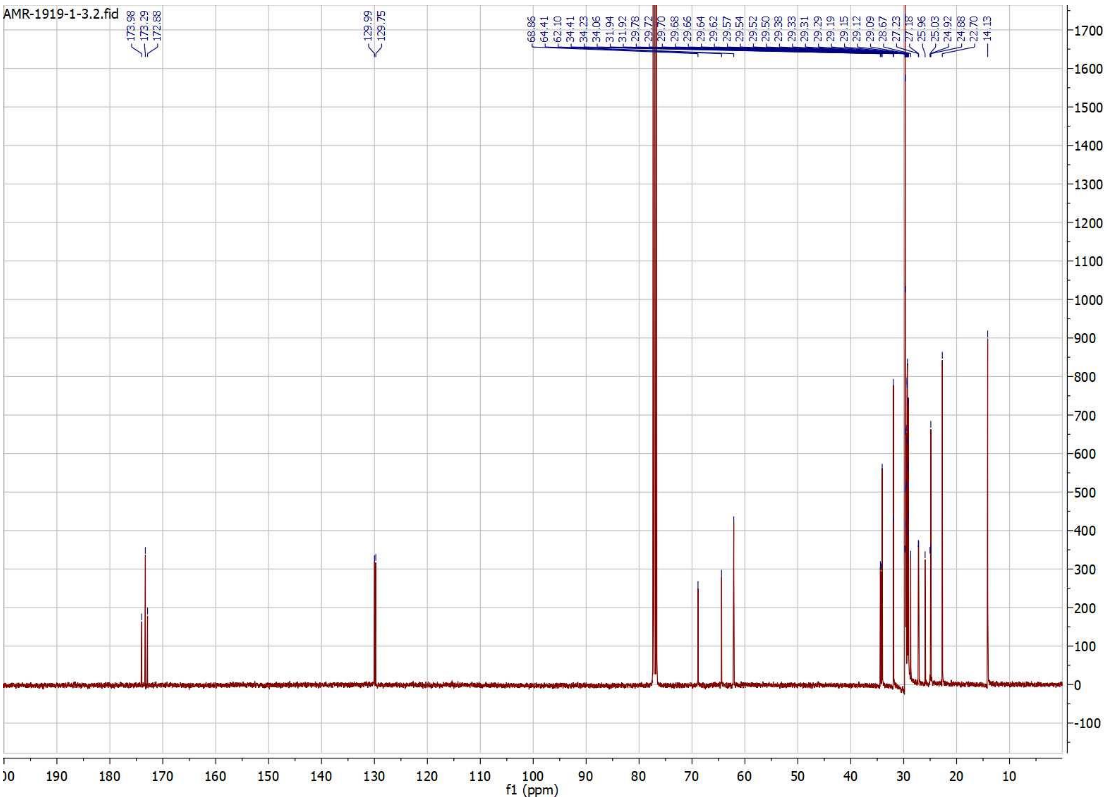

Figure S14. ^{13}C NMR spectrum of TG-EST **7**.

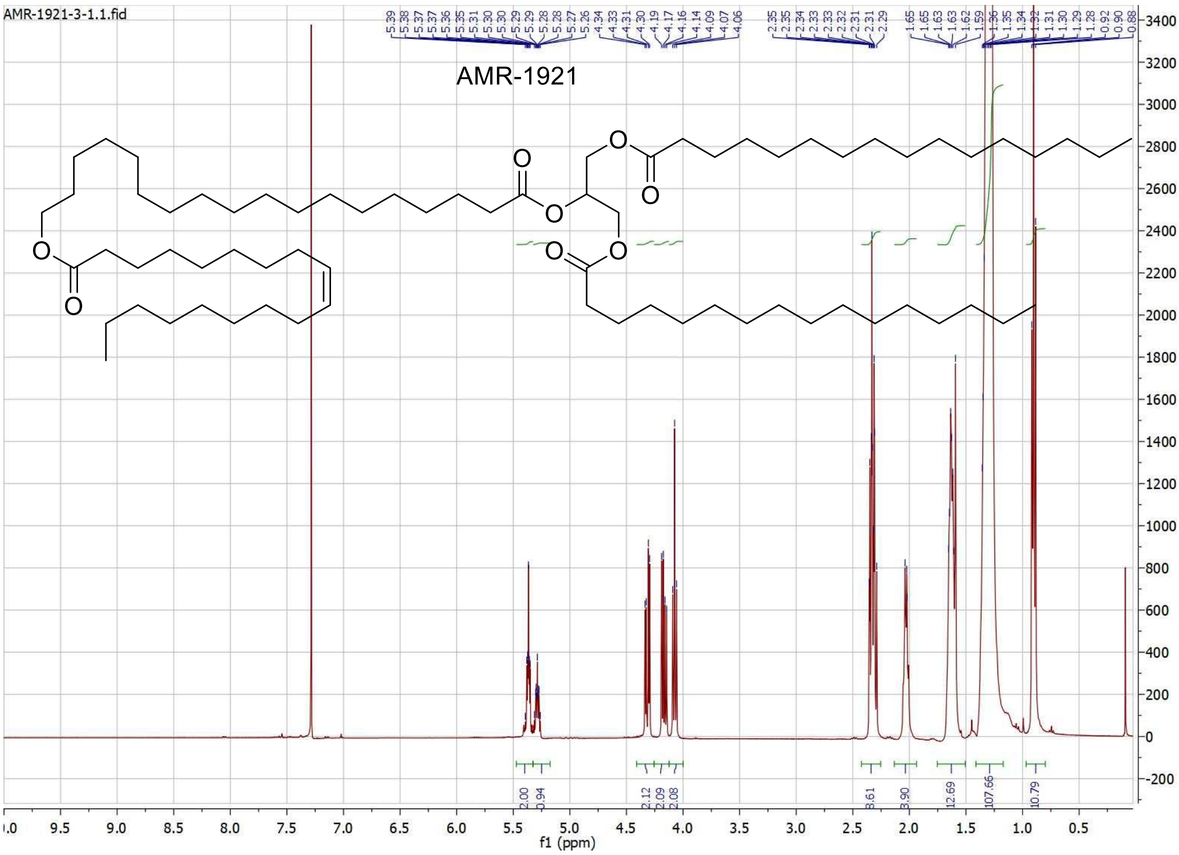

Figure S15. ^{1}H NMR spectrum of TG-EST **8**.

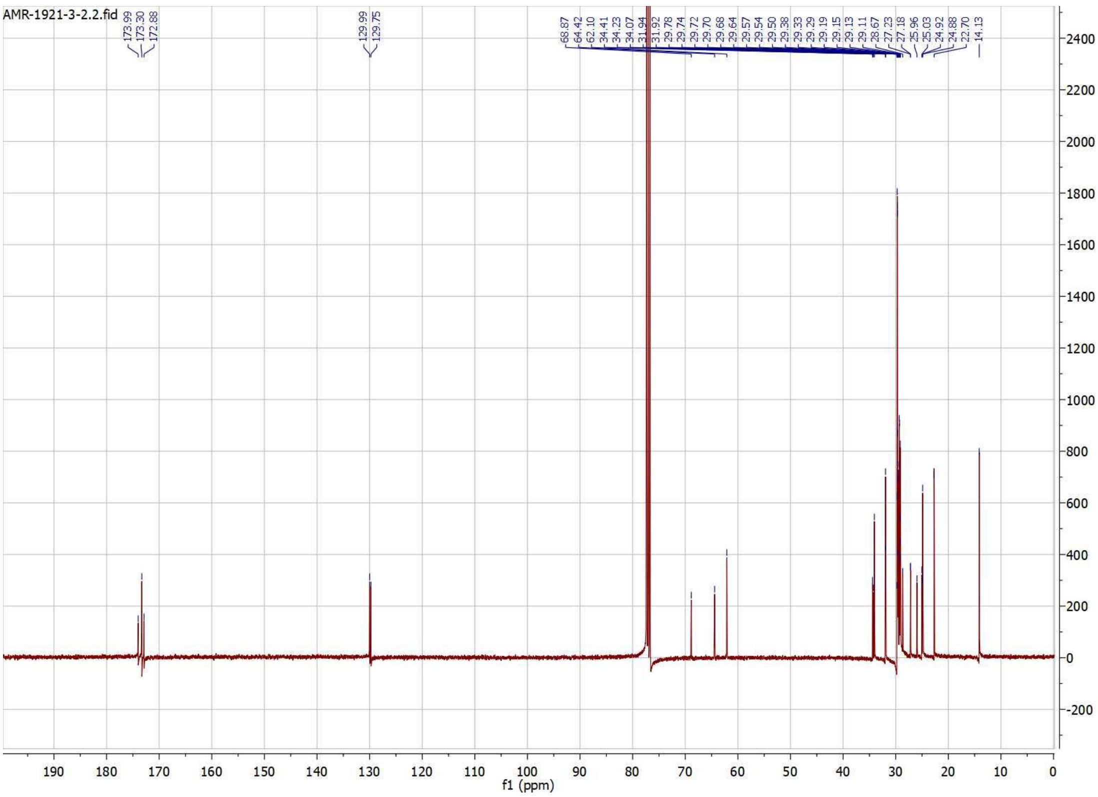

Figure S16. ^{13}C NMR spectrum of TG-EST **8**.

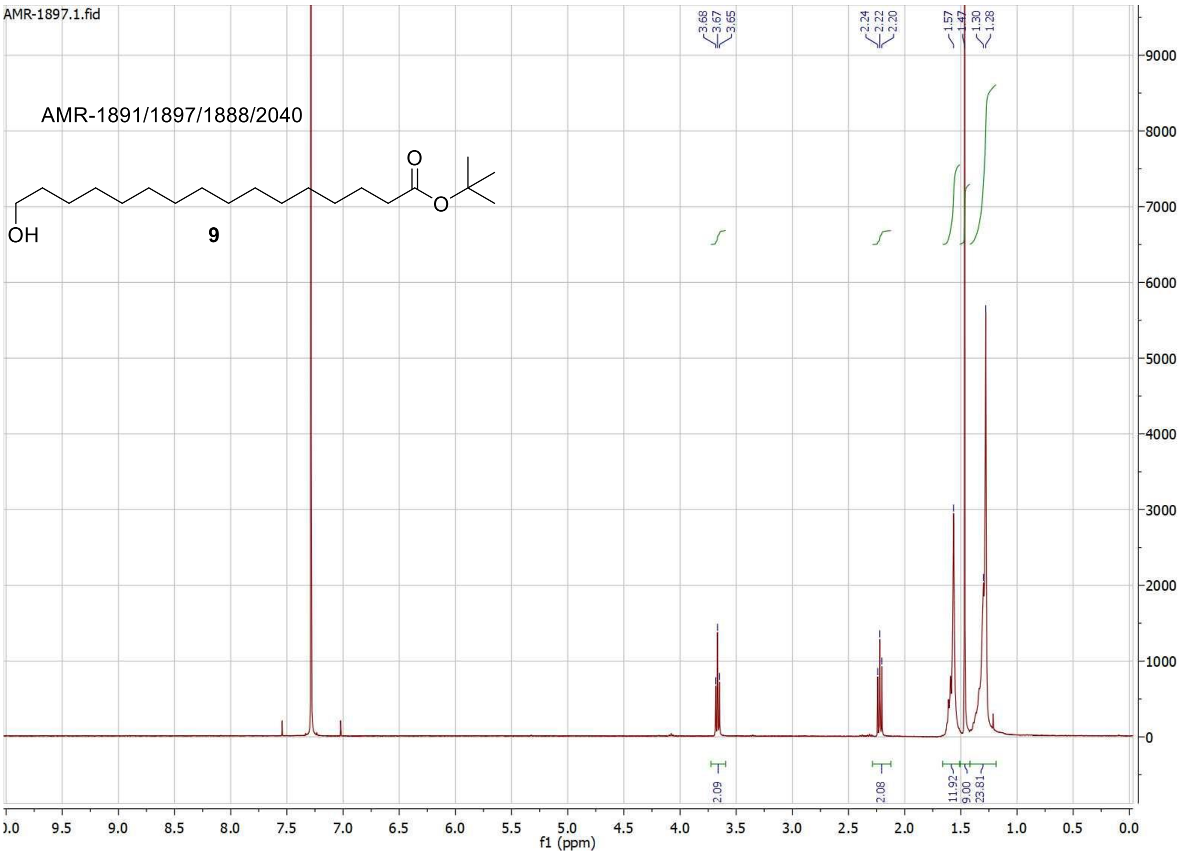

Figure S17. ^{1}H NMR spectrum of compound **9**.

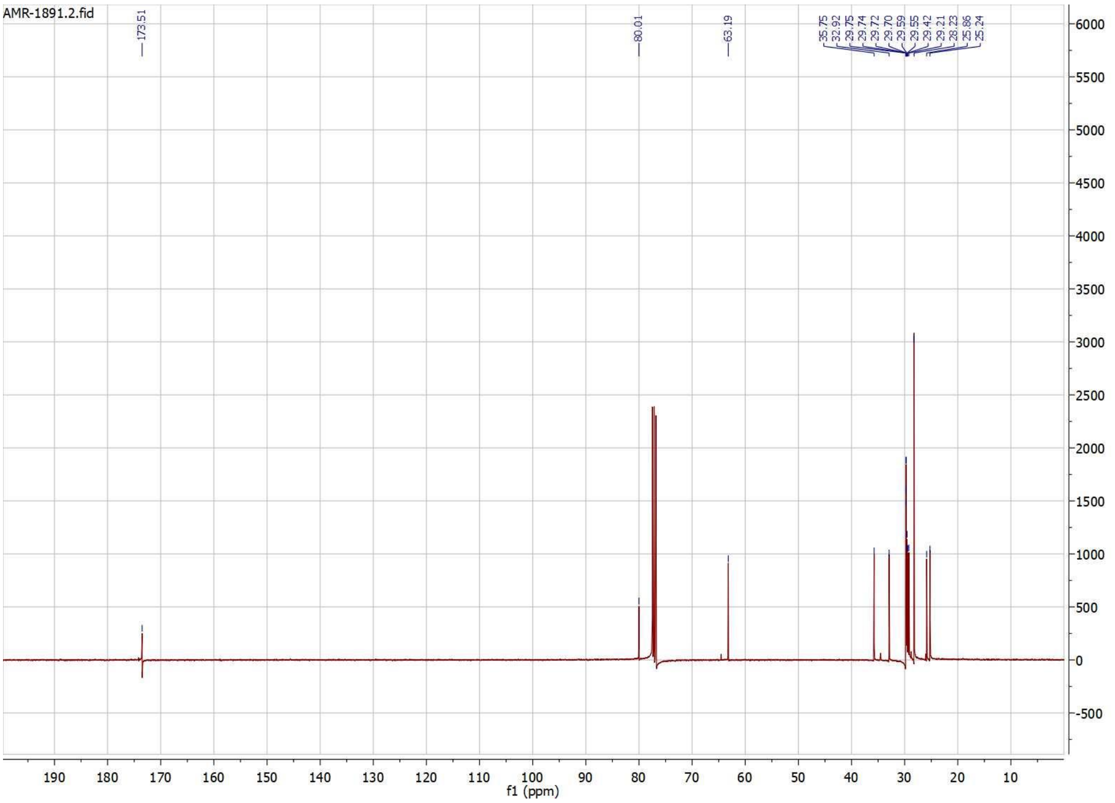

Figure S18. ^{13}C NMR spectrum of compound **9**.

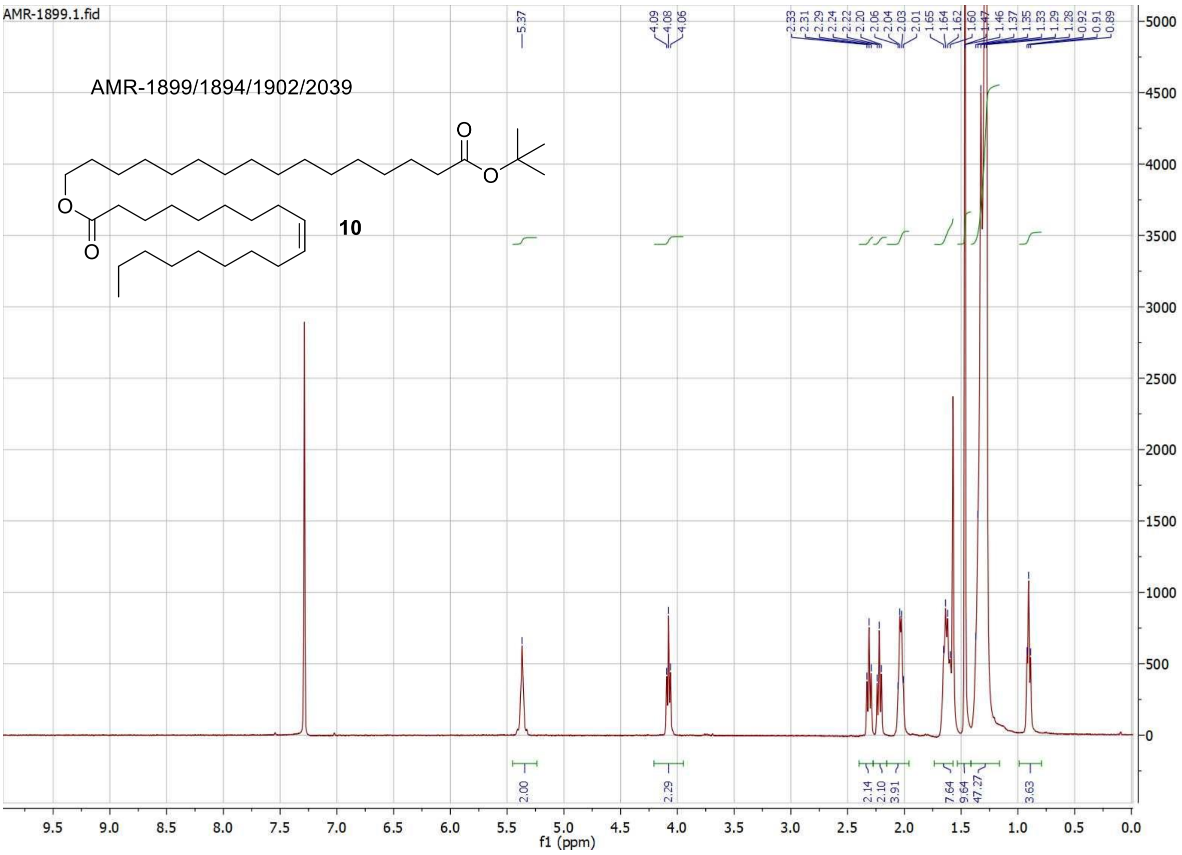

Figure S19. ^{1}H NMR spectrum of compound **10**.

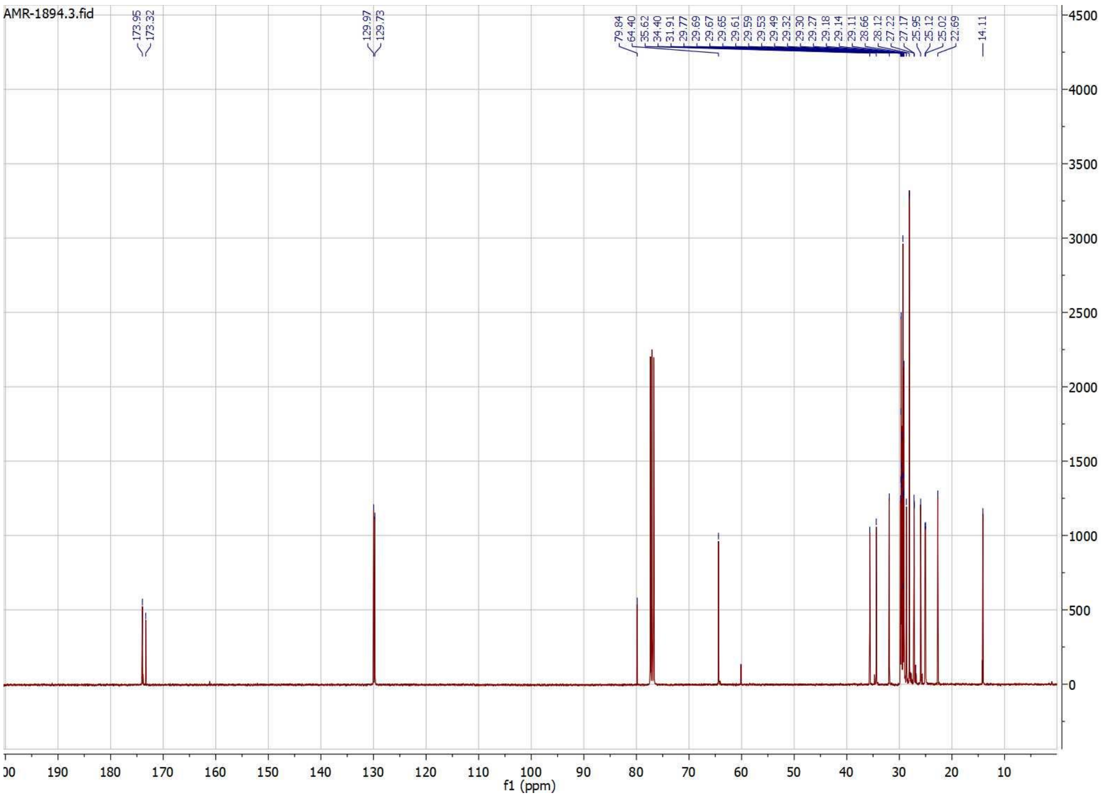

Figure S20. ^{13}C NMR spectrum of compound **10**.

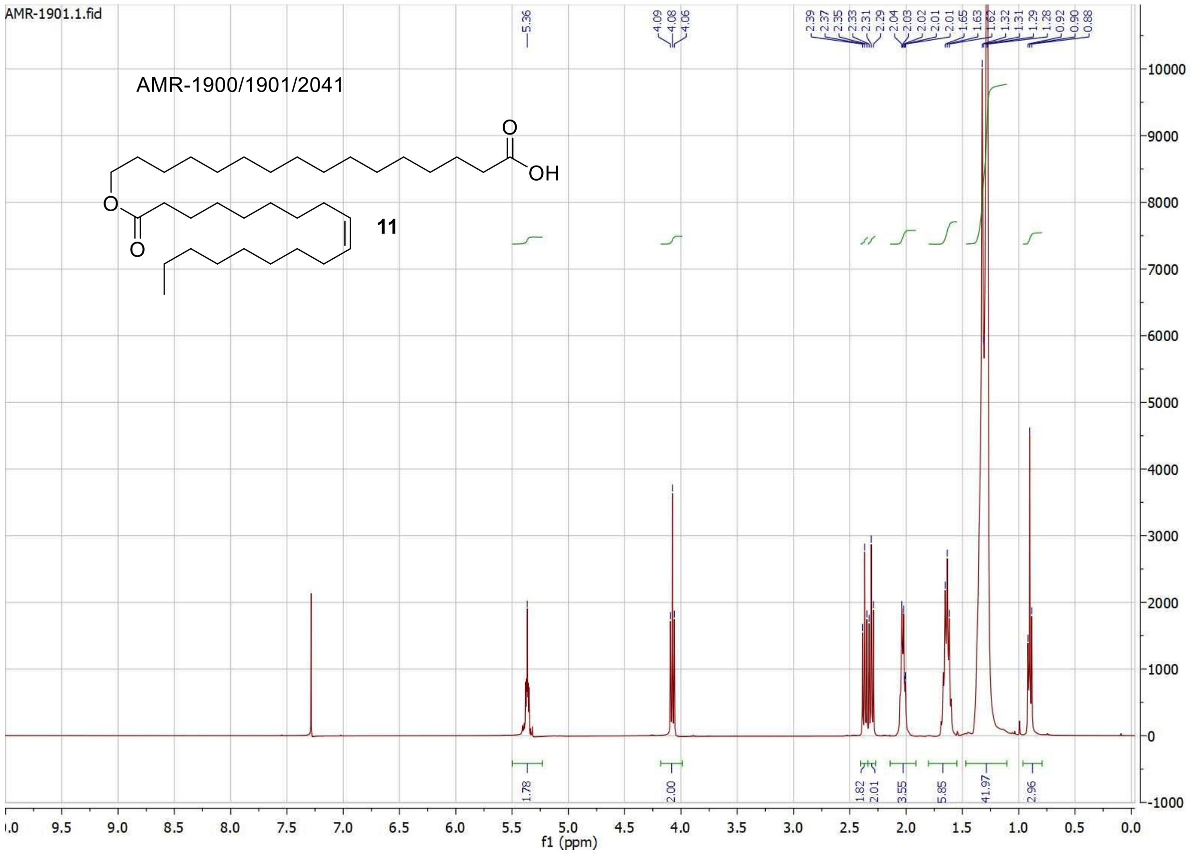

Figure S21. ^{1}H NMR spectrum of compound **11**.

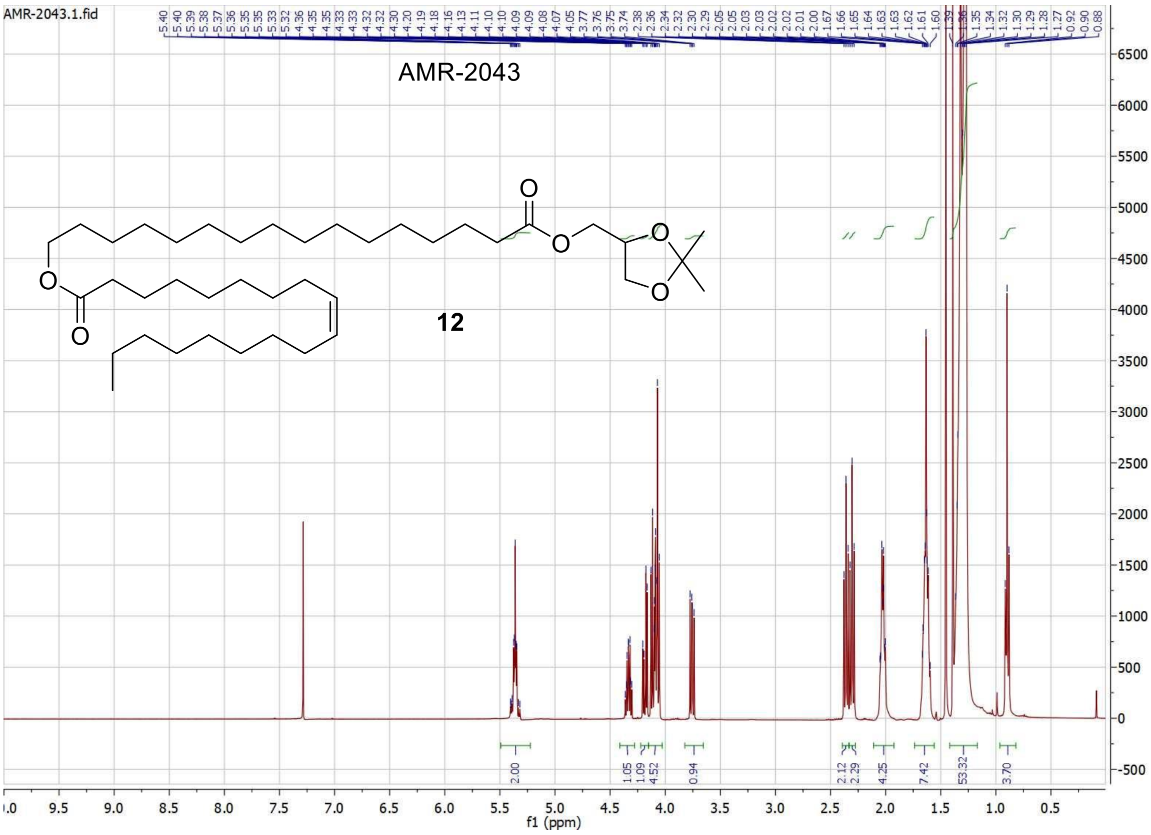

Figure S22. ^{1}H NMR spectrum of compound **12**.

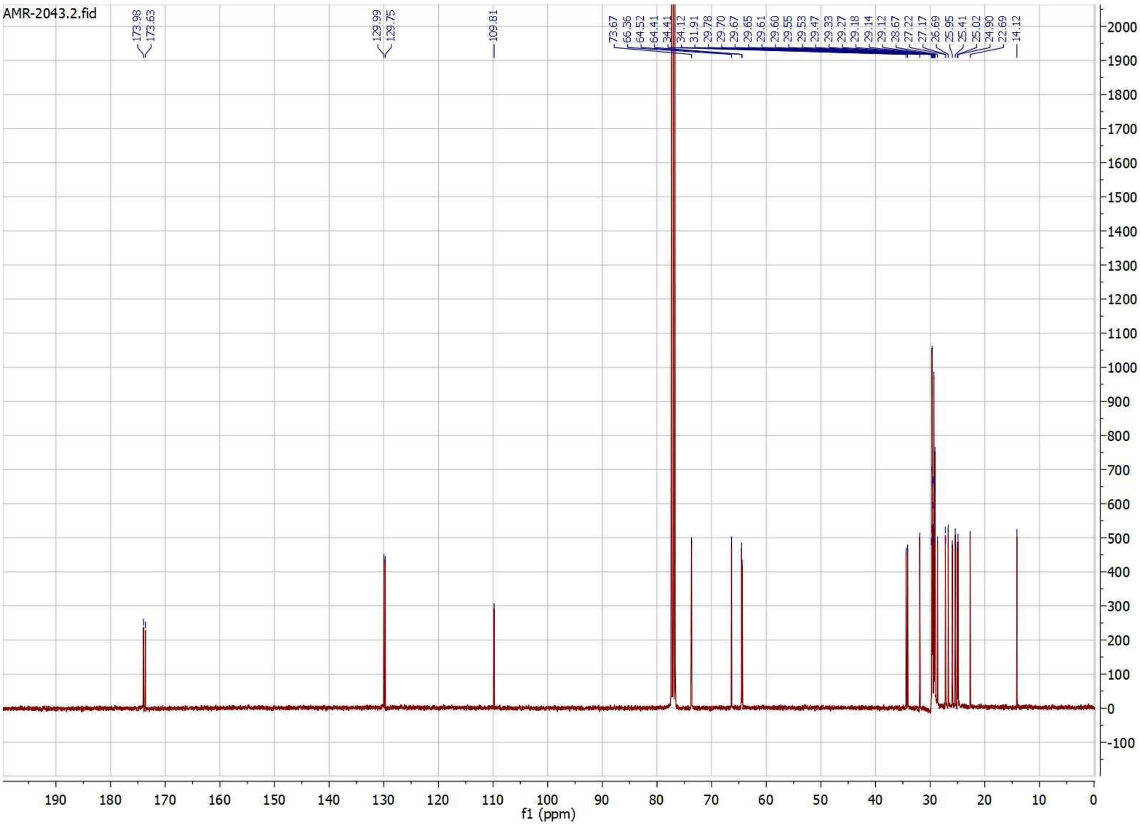

Figure S23. ^{13}C NMR spectrum of compound **12**.

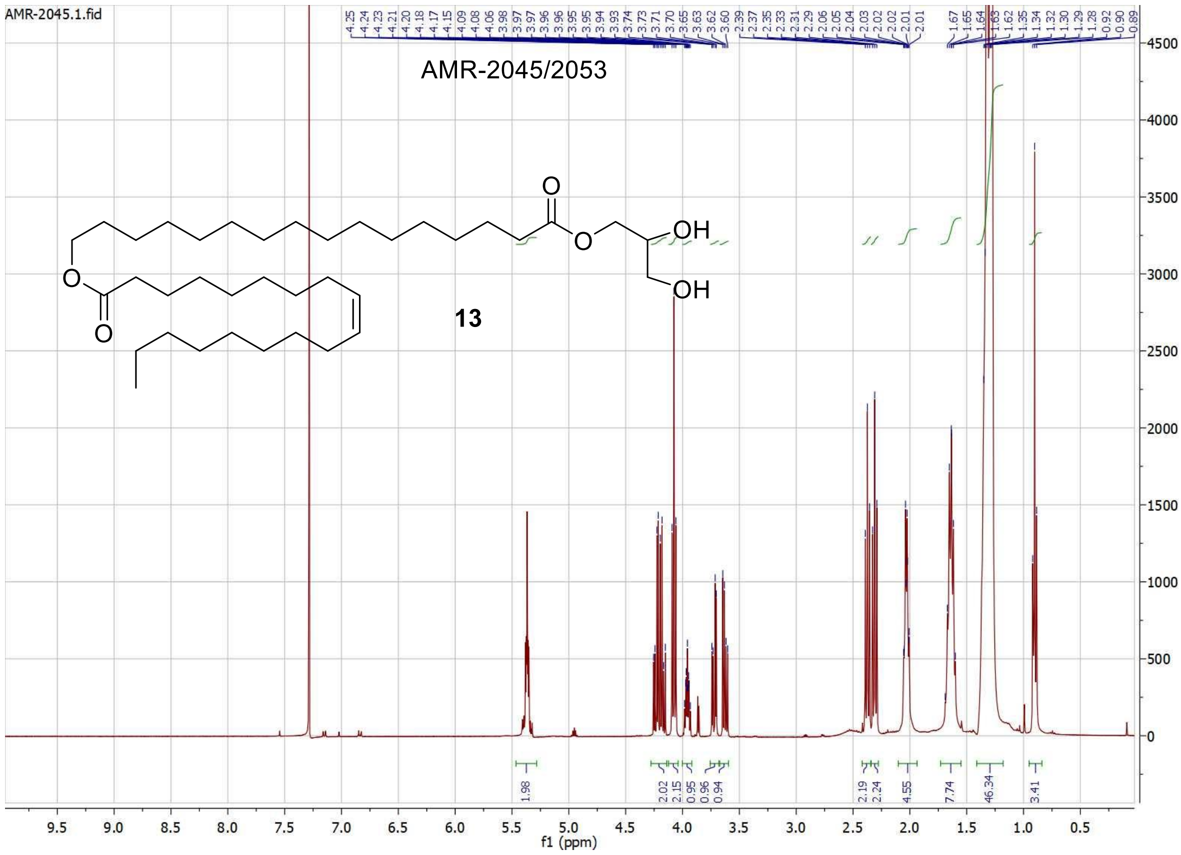

Figure S24. ^{1}H NMR spectrum of compound **13**.

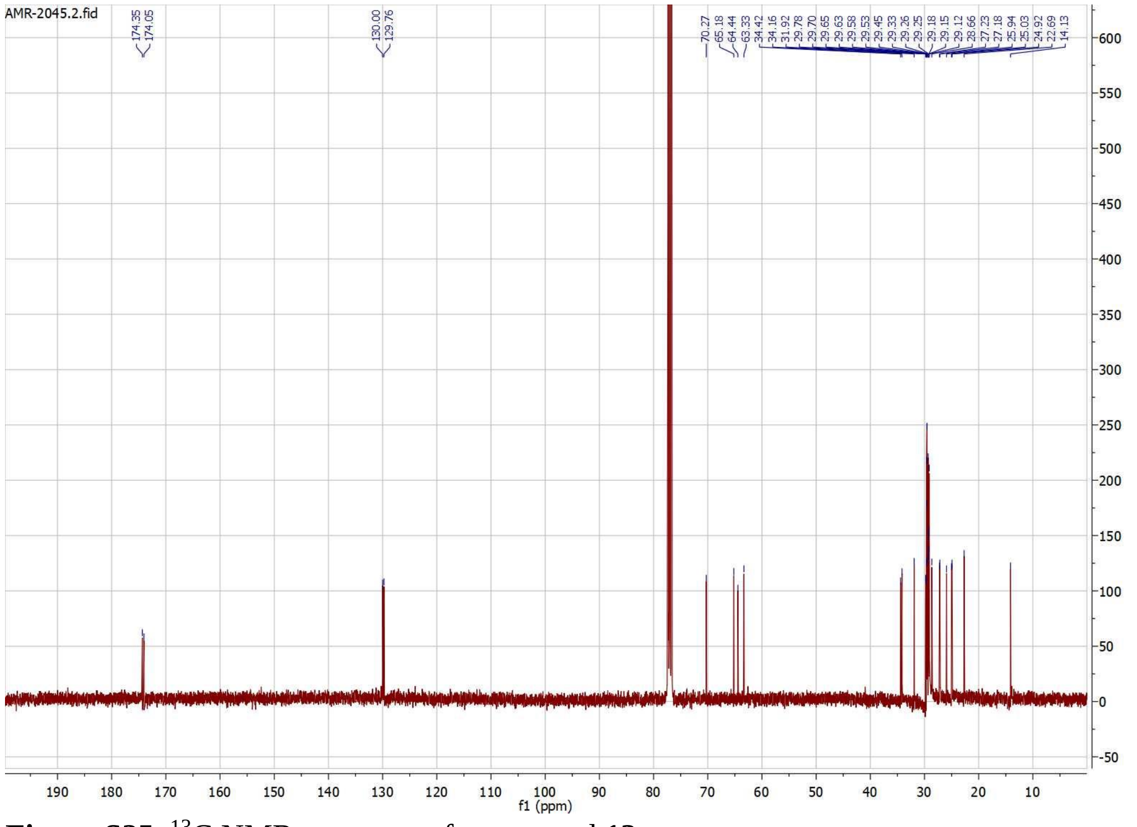

Figure S25. ^{13}C NMR spectrum of compound **13**.

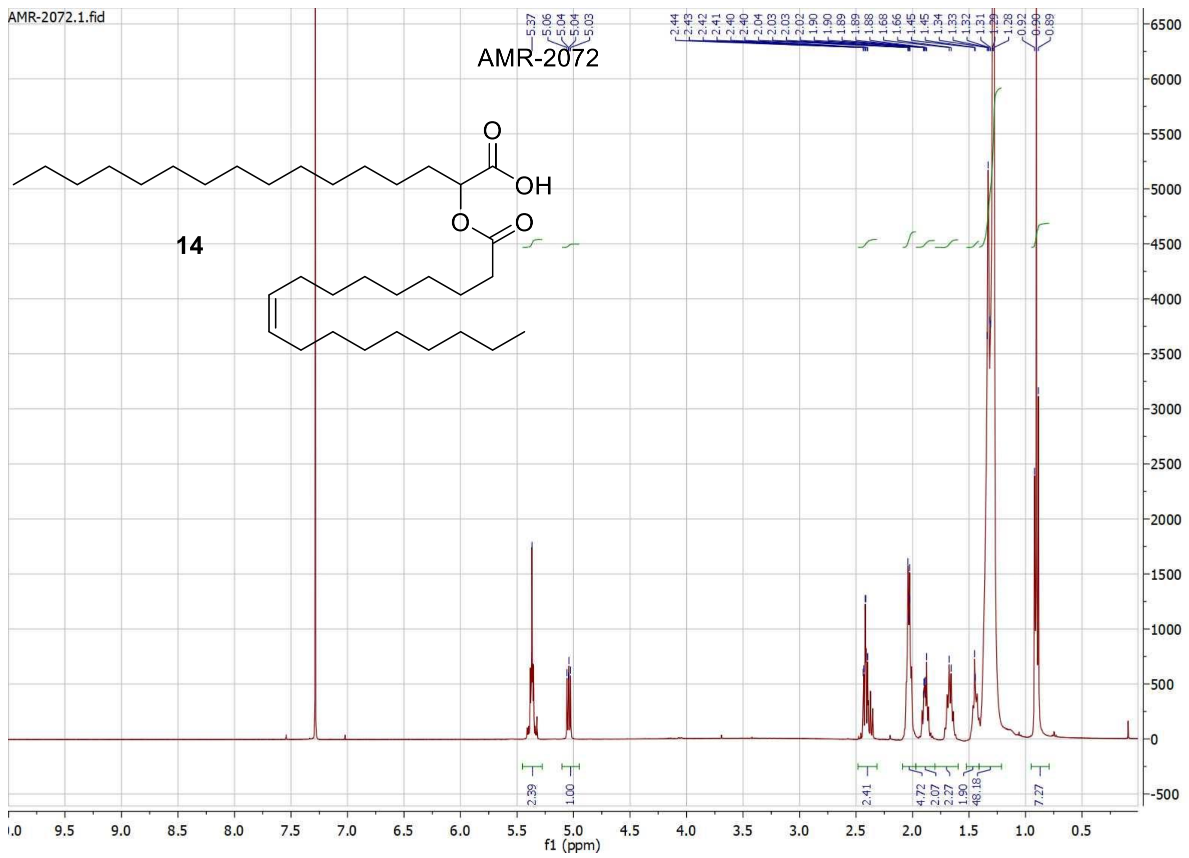

Figure S26. ^{1}H NMR spectrum of compound 1**4**.

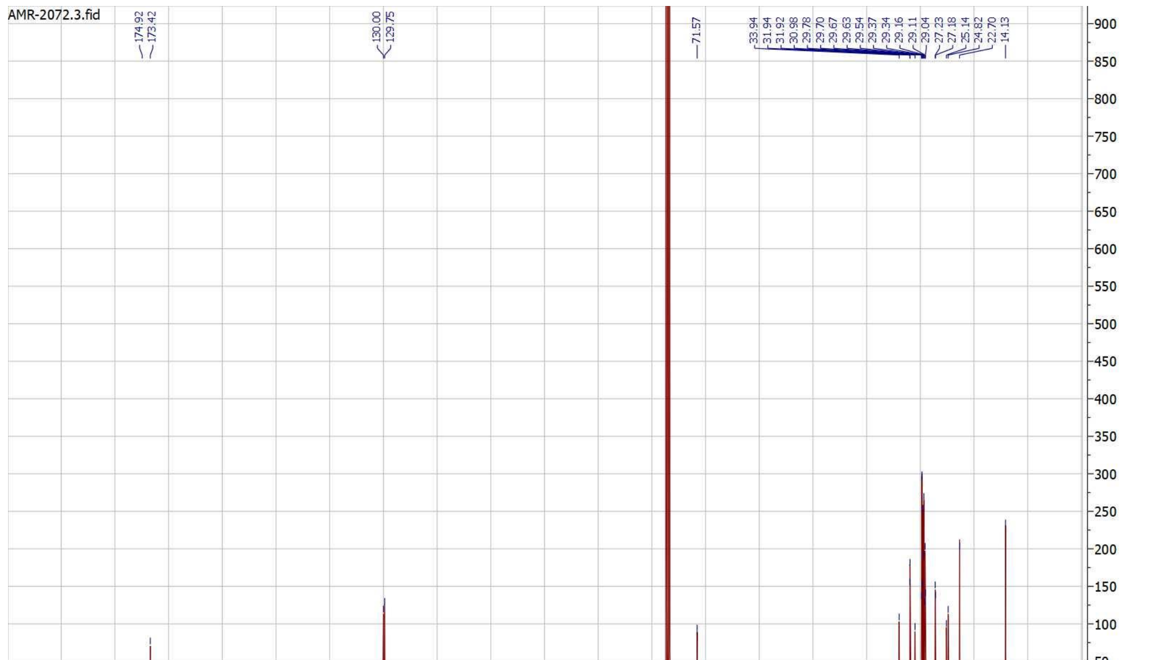

Figure S27. ^{13}C NMR spectrum of compound 1**4**.

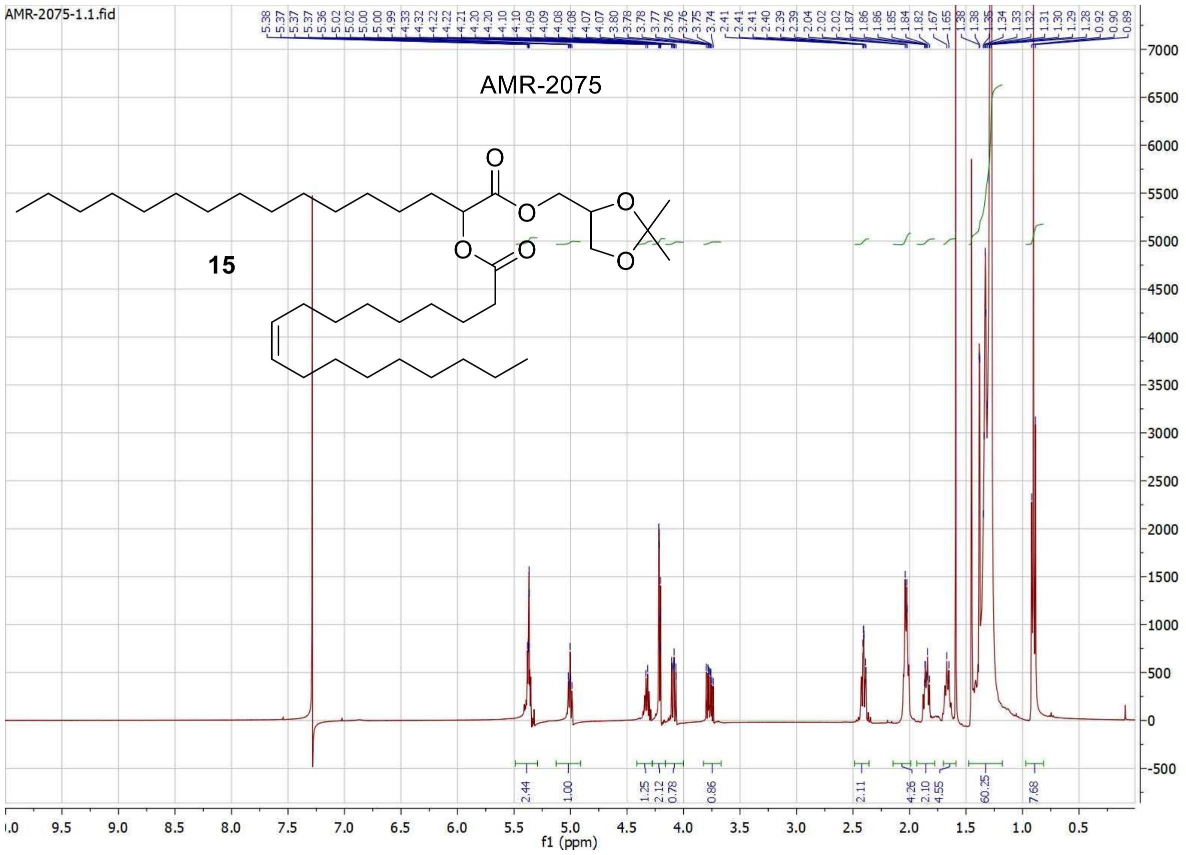

Figure S28. ^{1}H NMR spectrum of compound **15**.

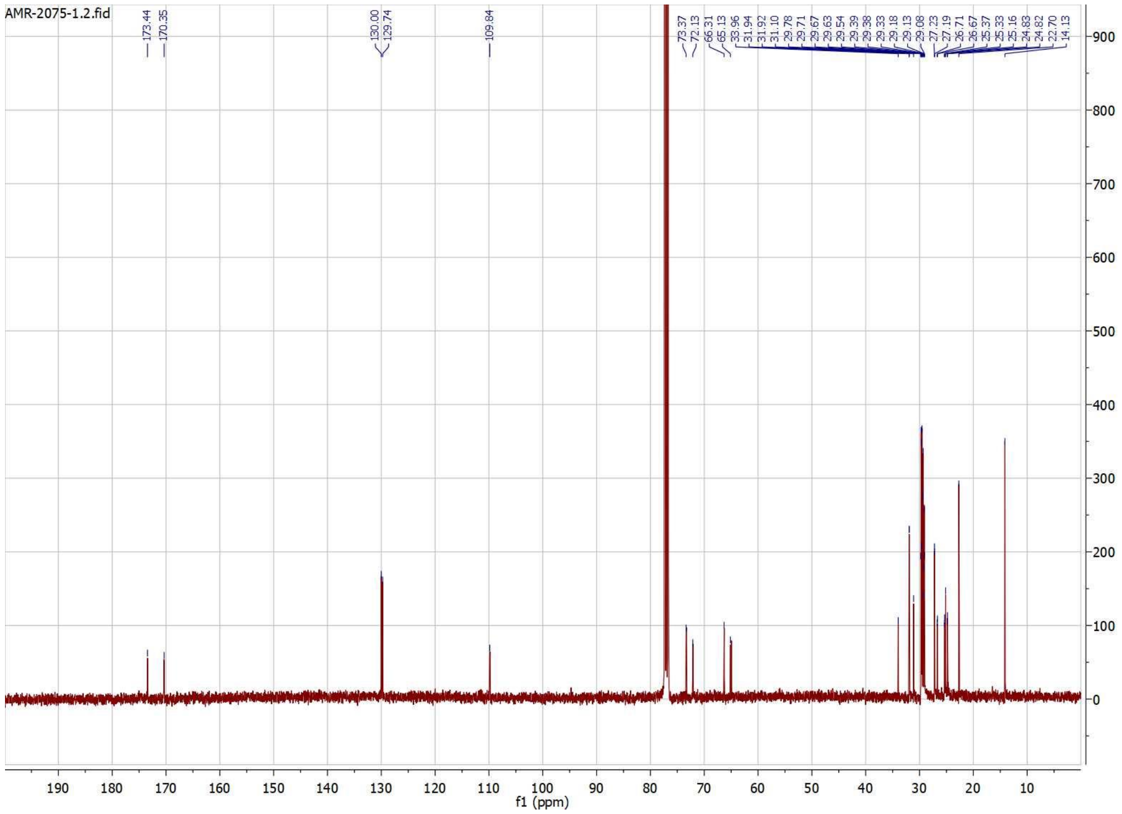

Figure S29. ^{13}C NMR spectrum of compound 1**5**.

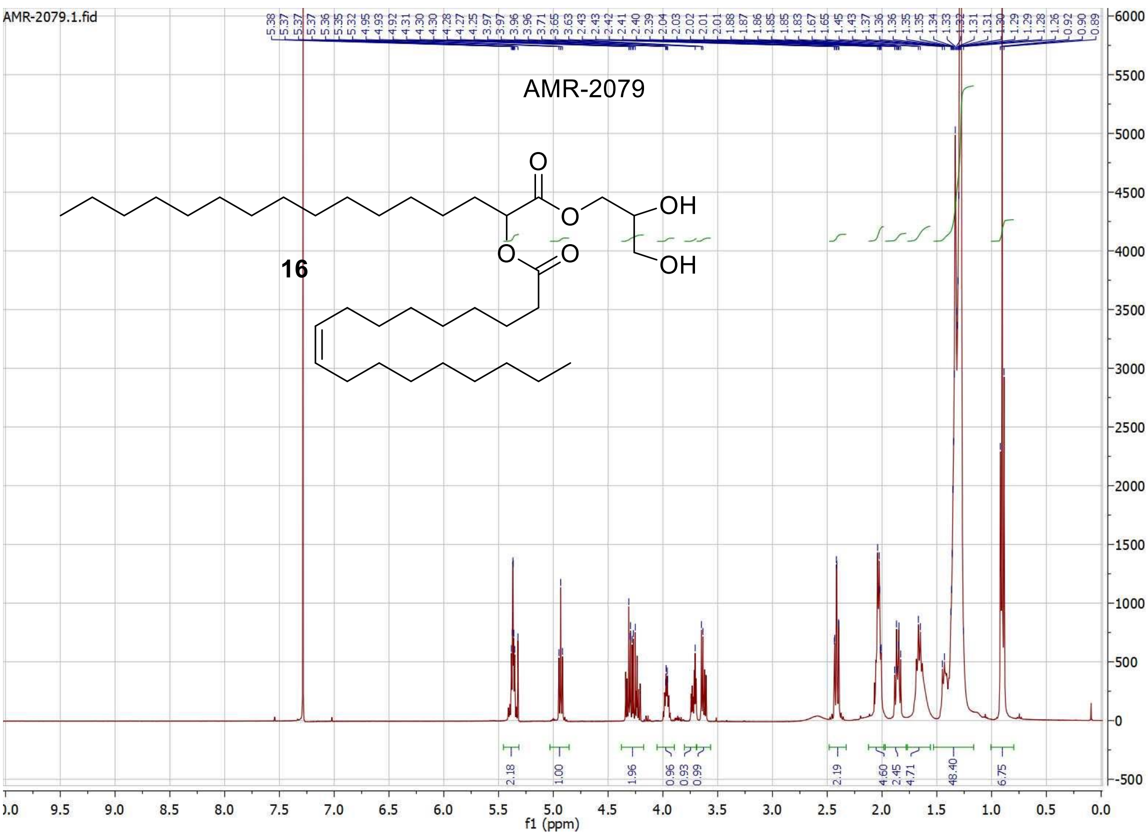

Figure S30. ^{1}H NMR spectrum of compound **16**.

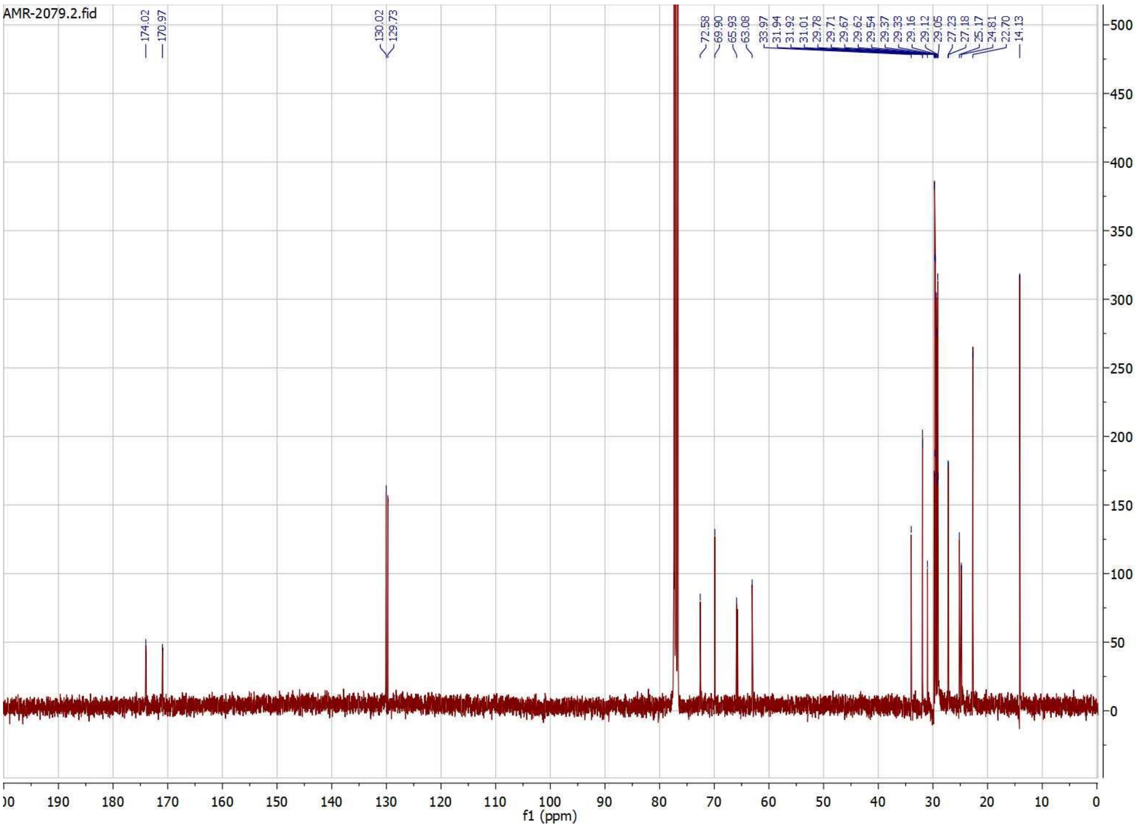

Figure S31. ^{13}C NMR spectrum of compound **16**.

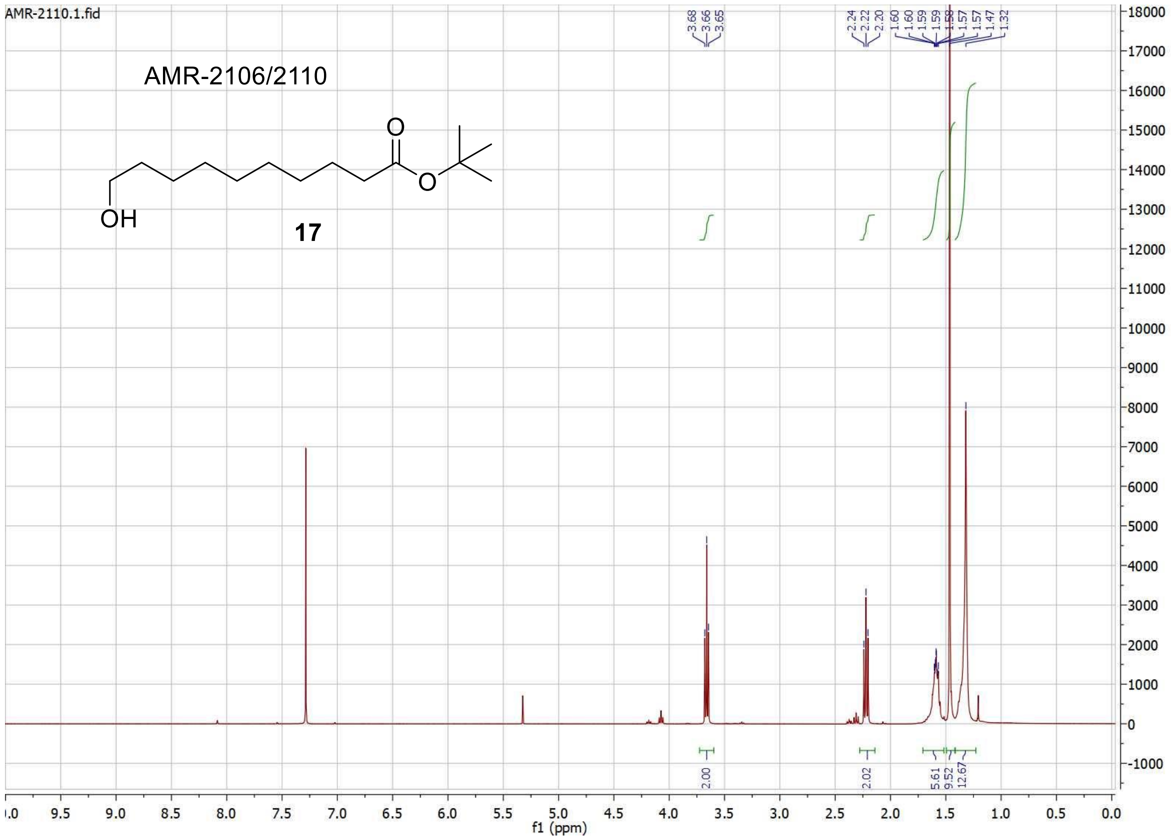

Figure S32. ^{1}H NMR spectrum of compound **17**.

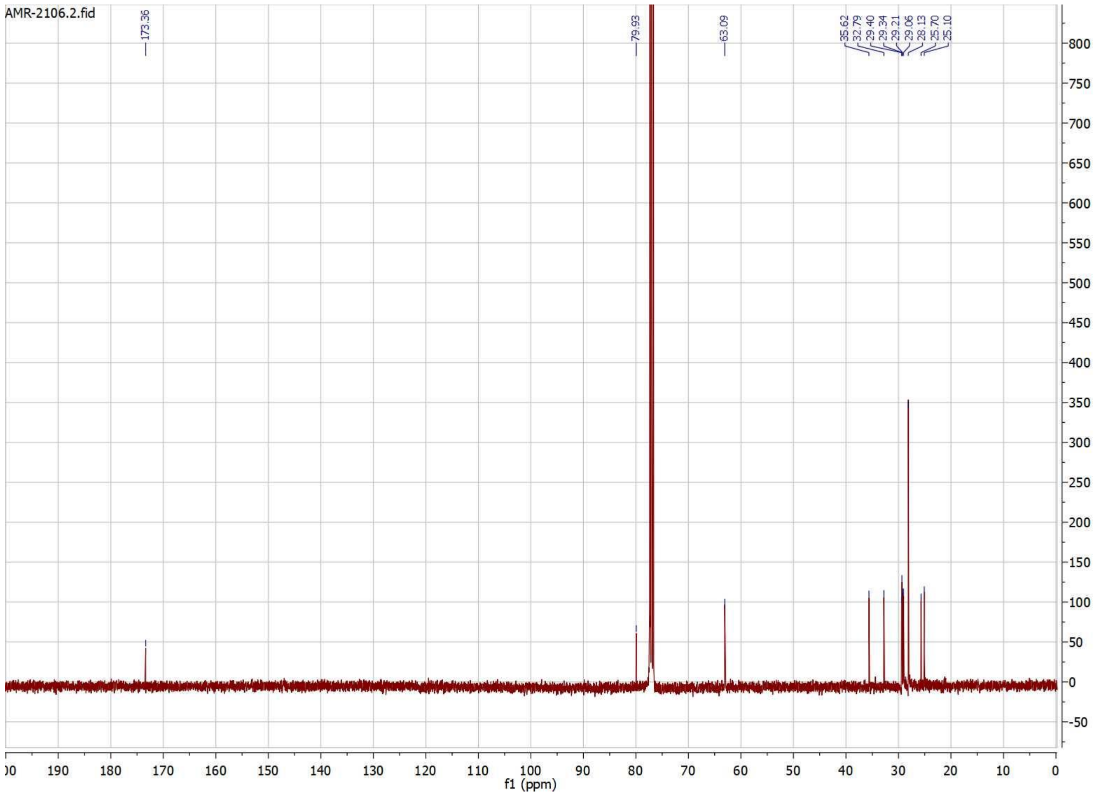

Figure S33. ^{13}C NMR spectrum of compound **17**.

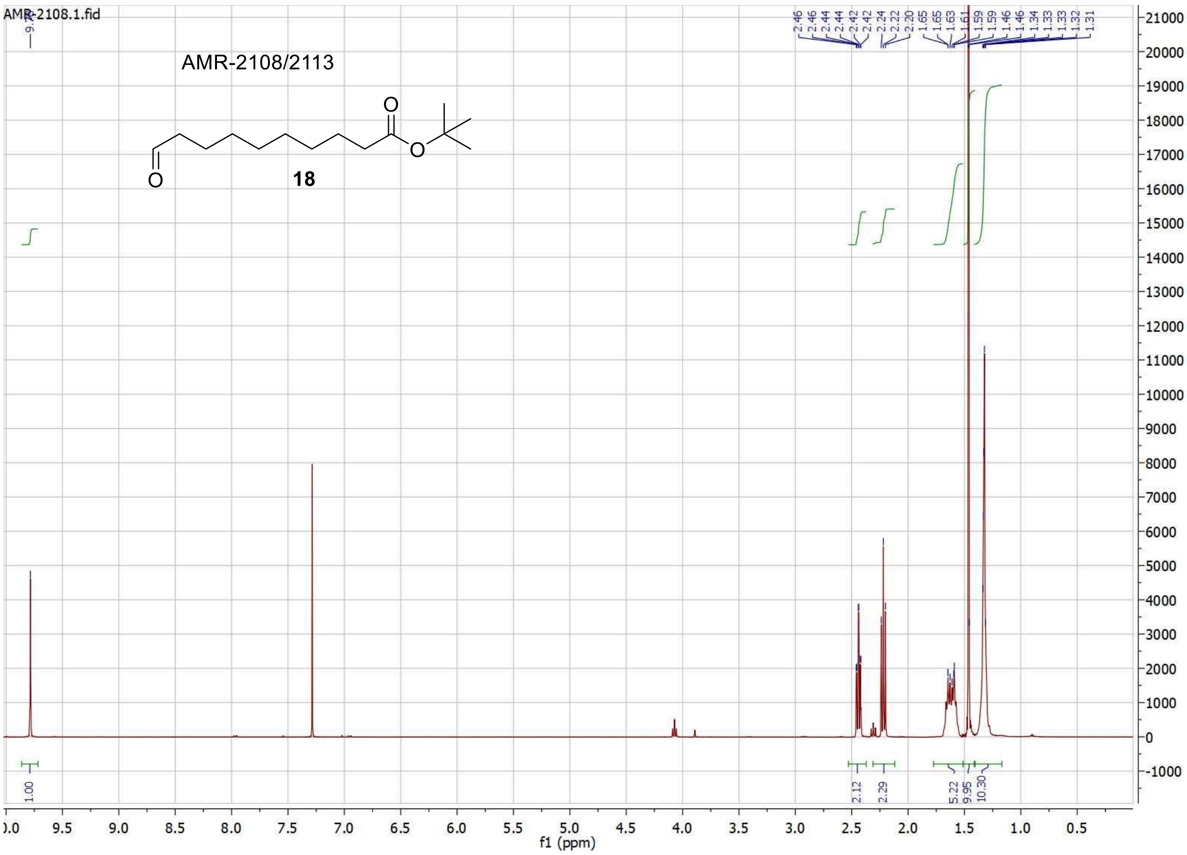

Figure S34. ^{1}H NMR spectrum of compound **18**.

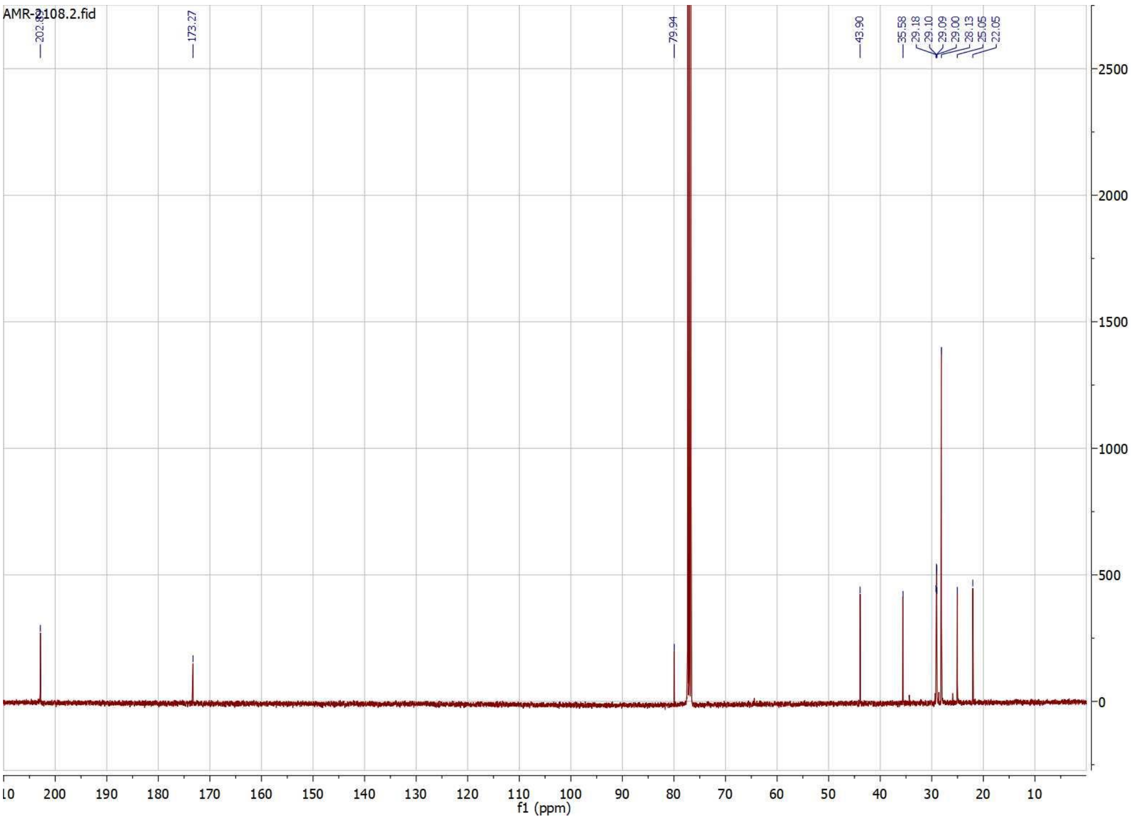

Figure S35. ^{13}C NMR spectrum of compound **18**.

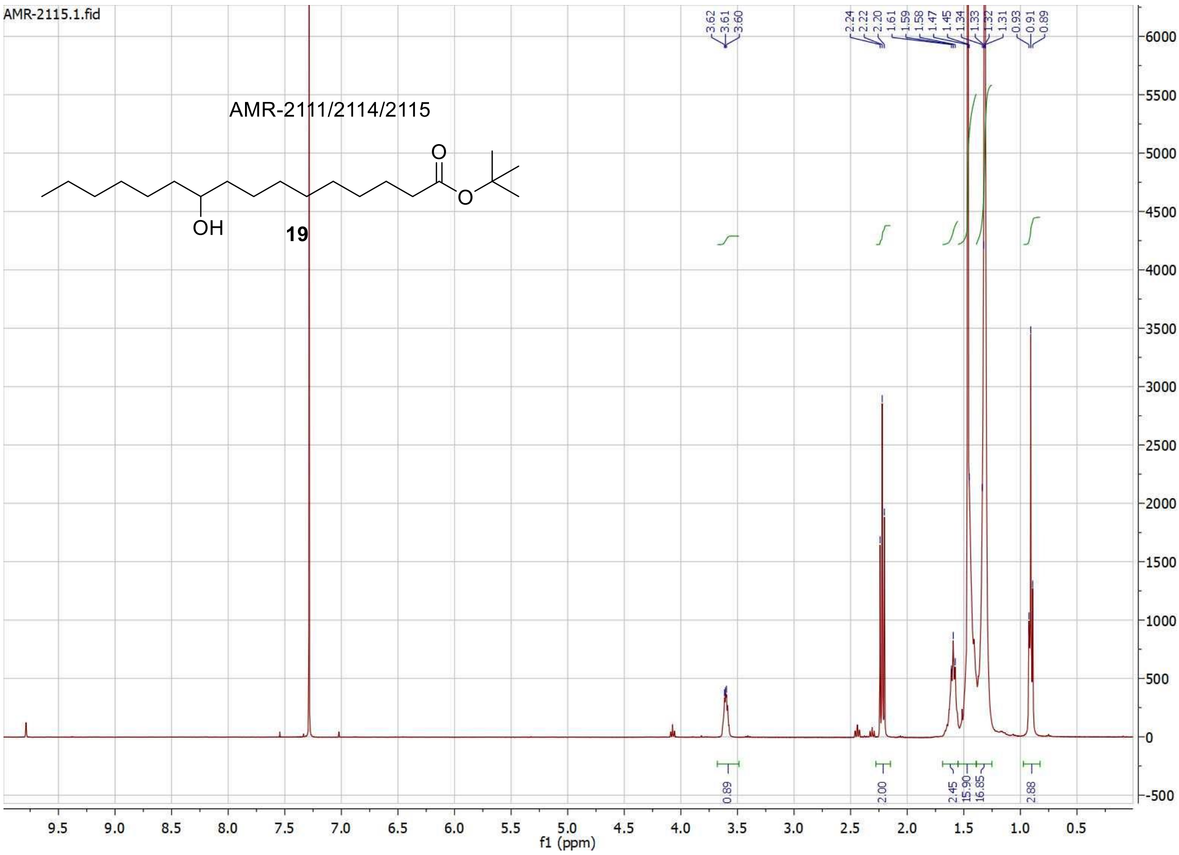

Figure S36. ^{1}H NMR spectrum of compound **19**.

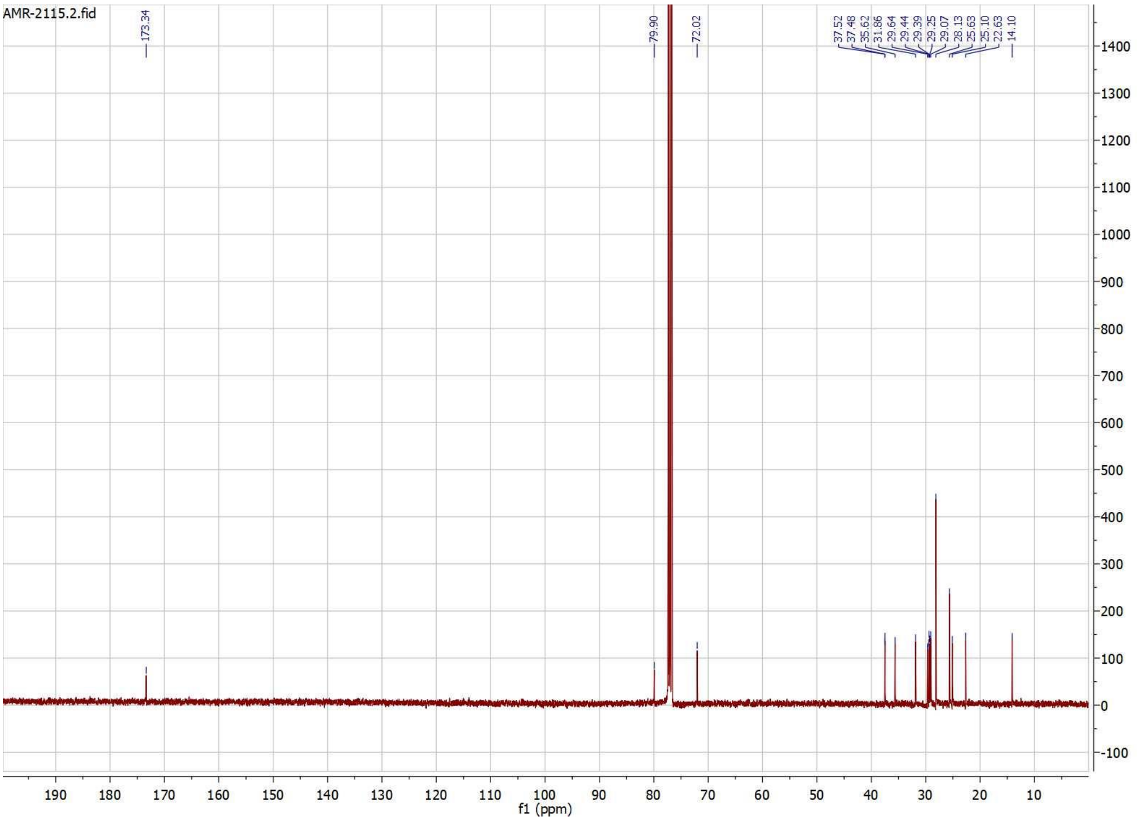

Figure S37. ^{13}C NMR spectrum of compound **19**.

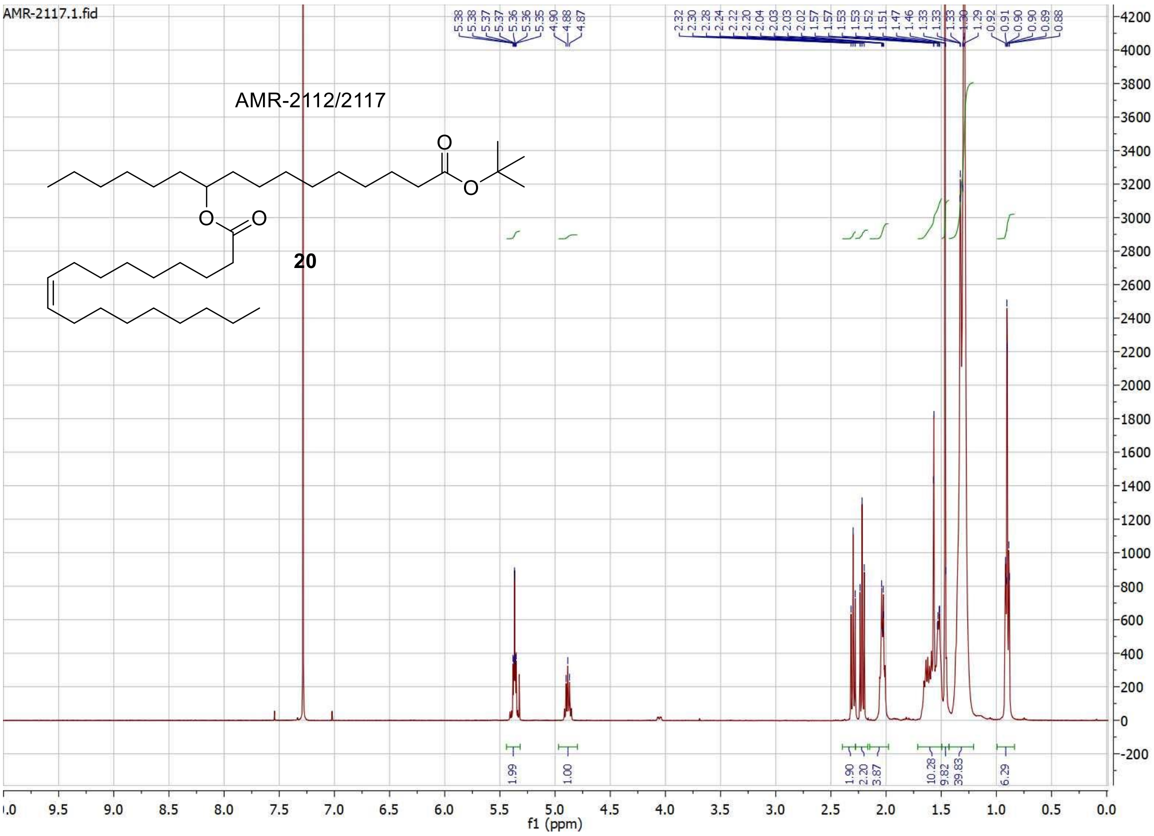

Figure S38. ^{1}H NMR spectrum of compound **20**.

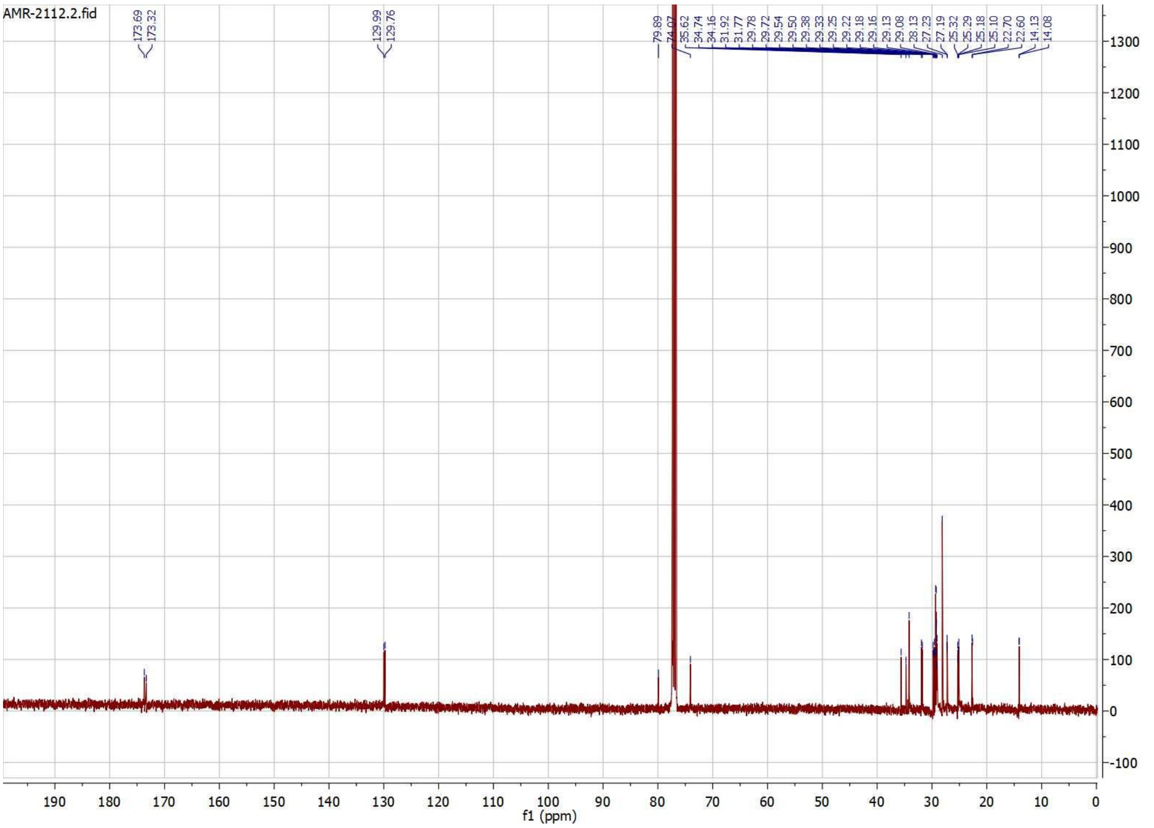

Figure S39. ^{13}C NMR spectrum of compound **20**.

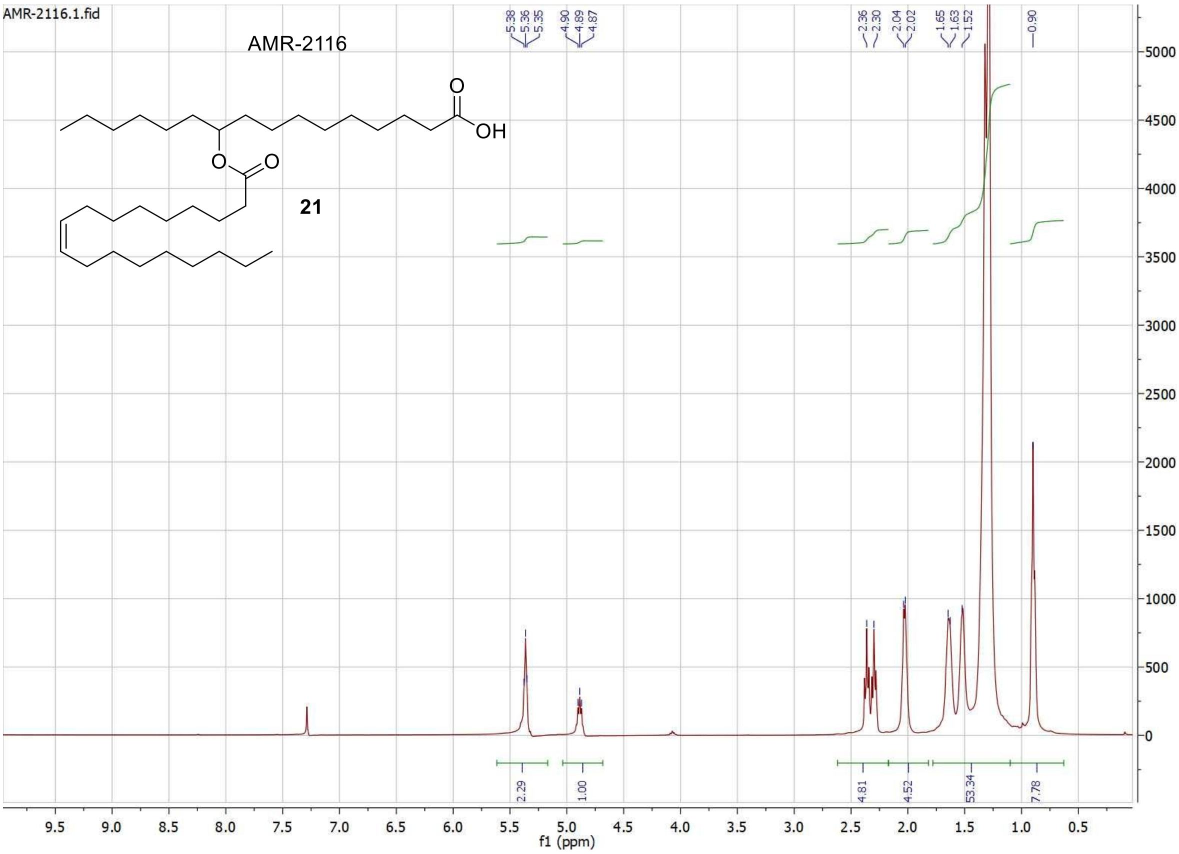

Figure S40. ^{1}H NMR spectrum of compound **21**.

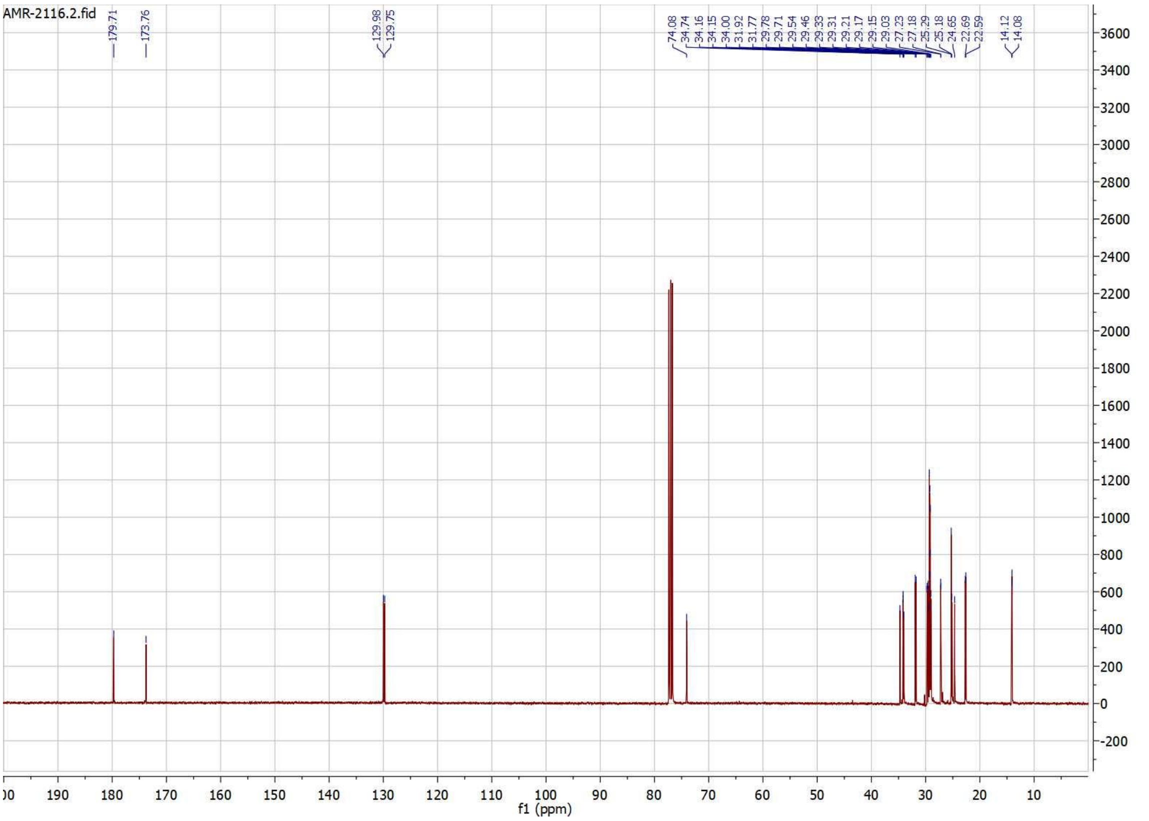

Figure S41. ^{13}C NMR spectrum of compound **21**.

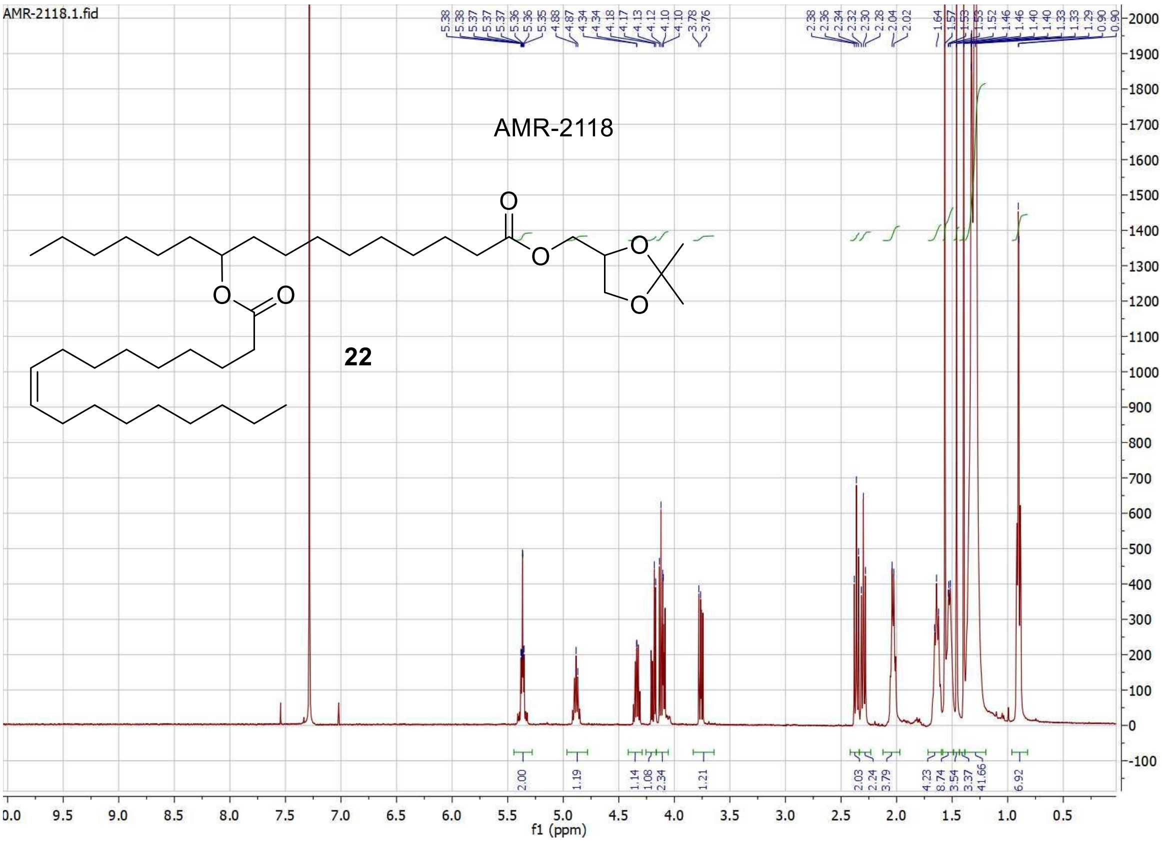

Figure S42. ^{1}H NMR spectrum of compound **22**.

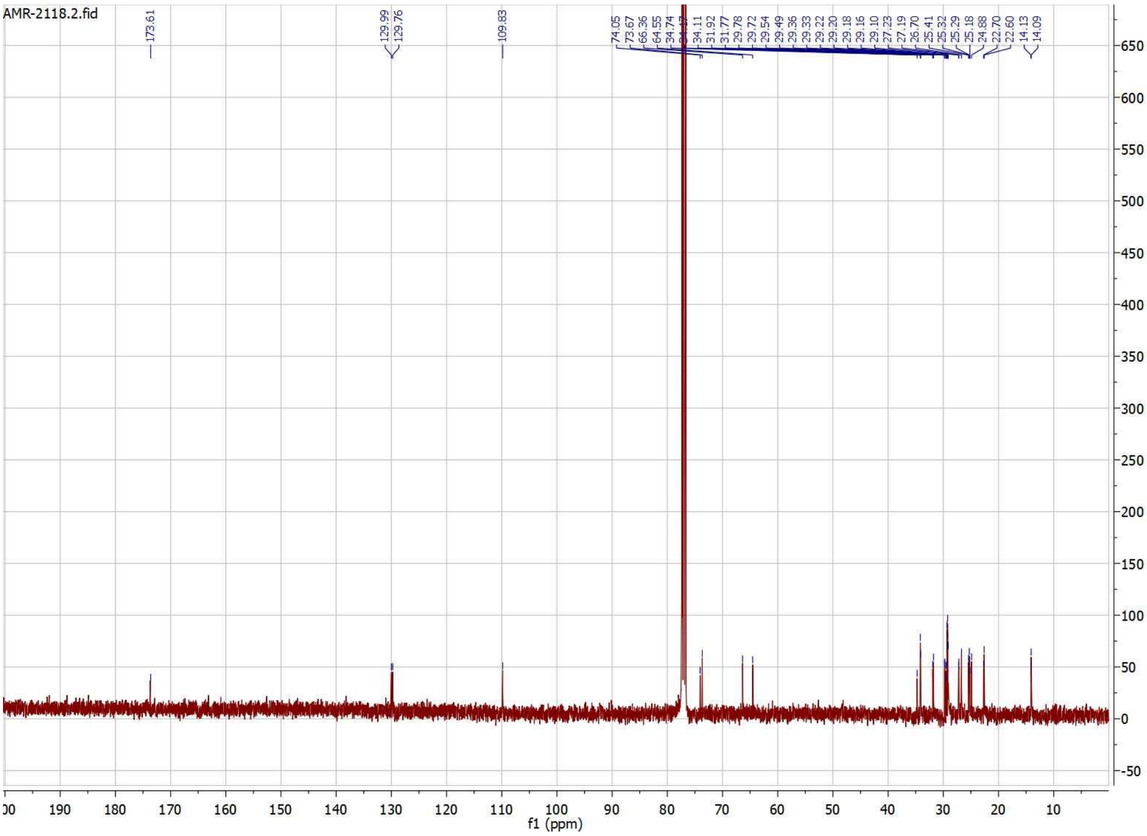

Figure S43. ^{13}C NMR spectrum of compound **22**.

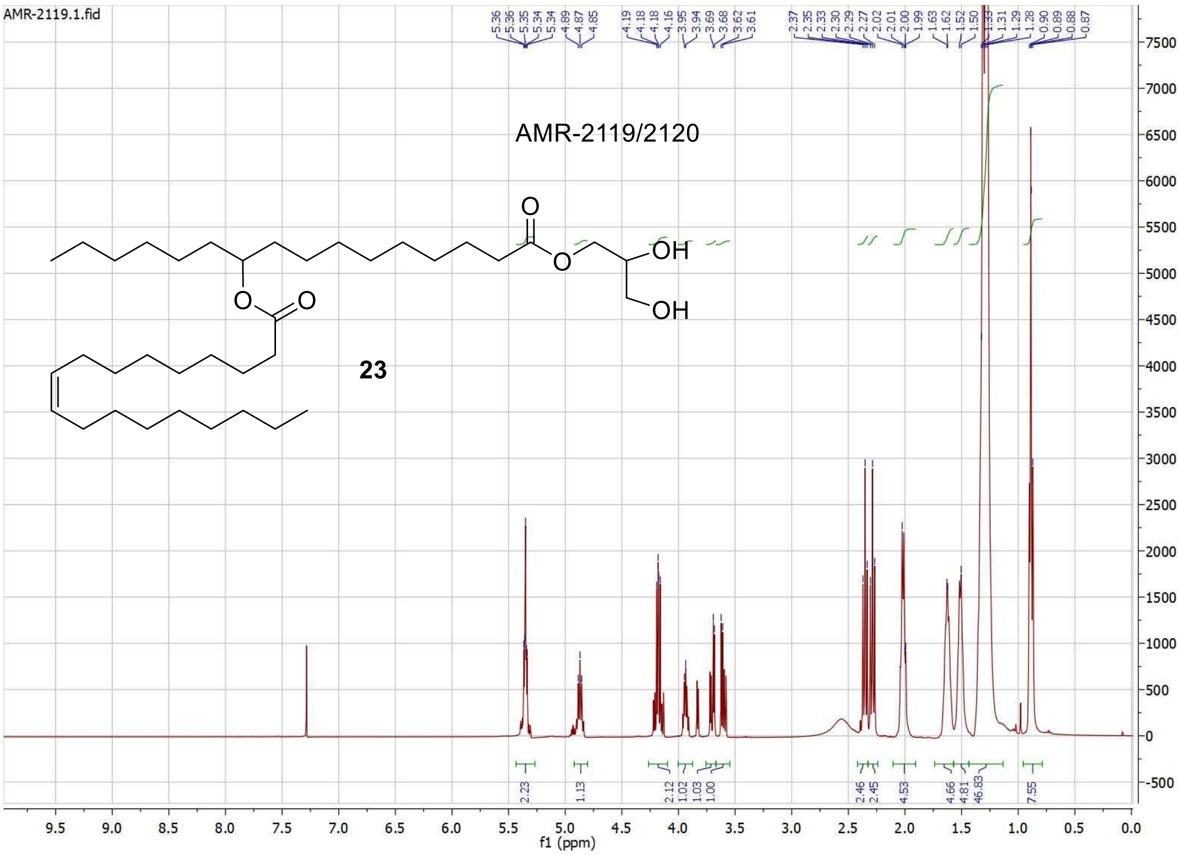

Figure S44. ^{1}H NMR spectrum of compound **23**.

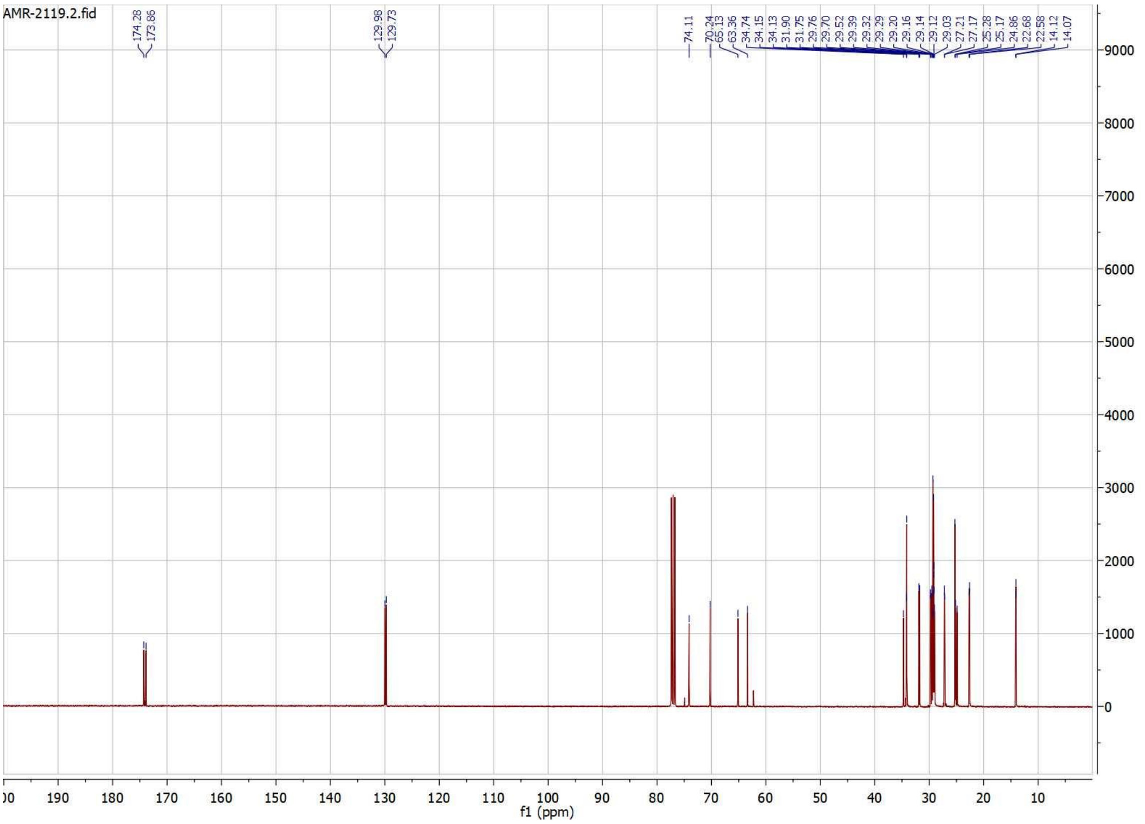

Figure S45. ^{13}C NMR spectrum of compound **23**.

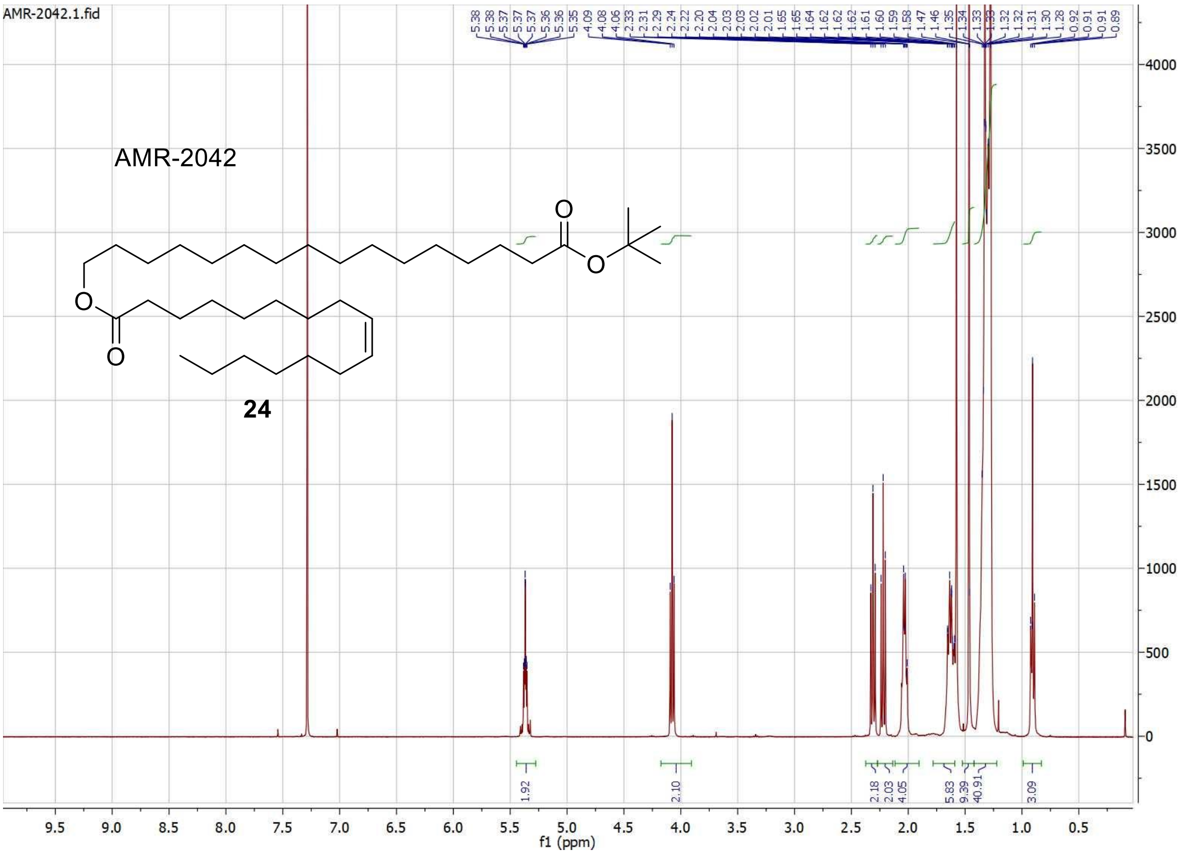

Figure S46. ^{1}H NMR spectrum of compound **24**.

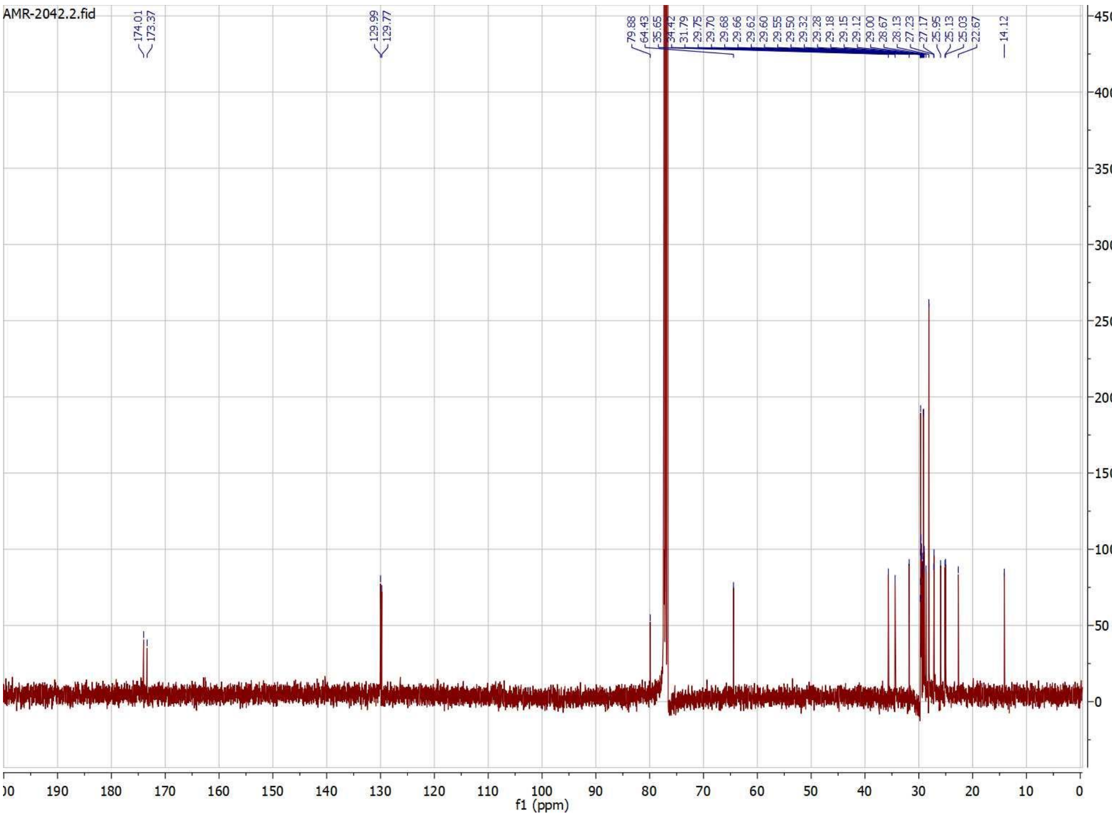

Figure S47. ^{13}C NMR spectrum of compound **24**.

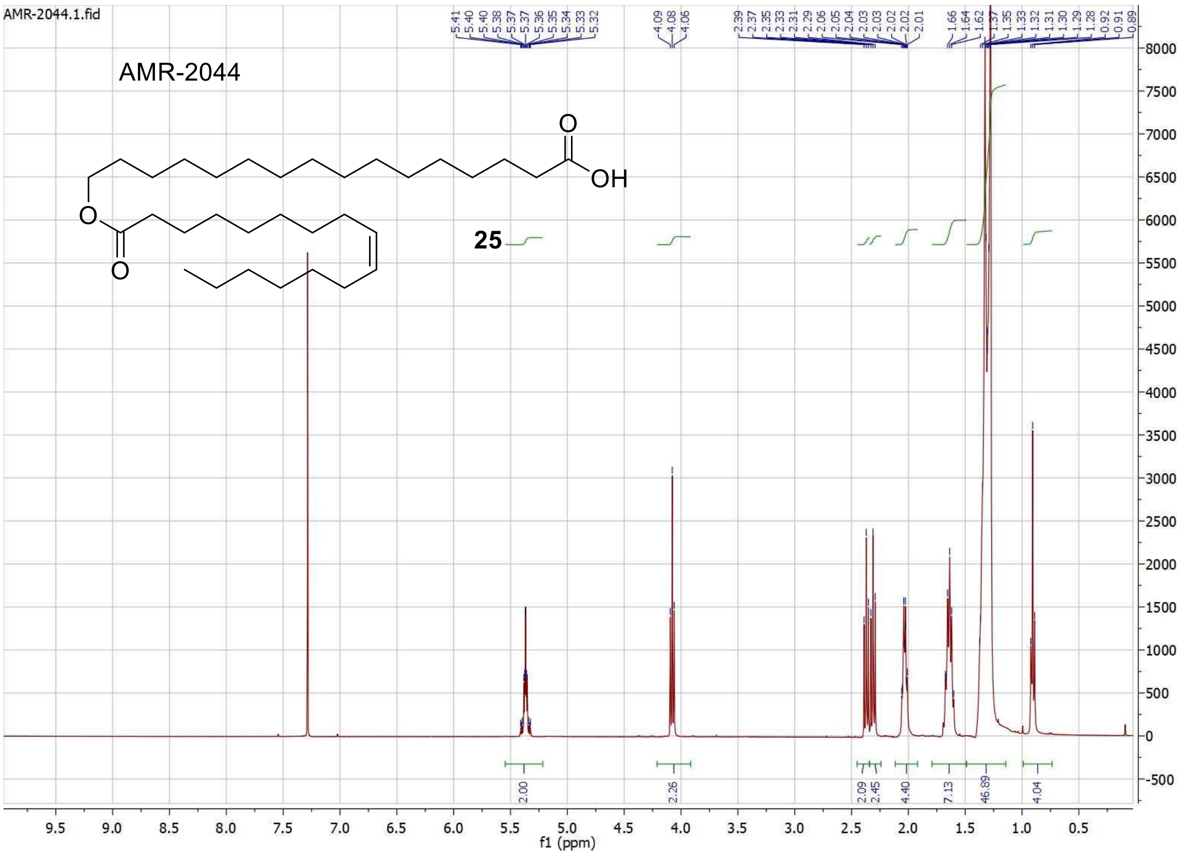

Figure S48. ^{1}H NMR spectrum of compound **25**.

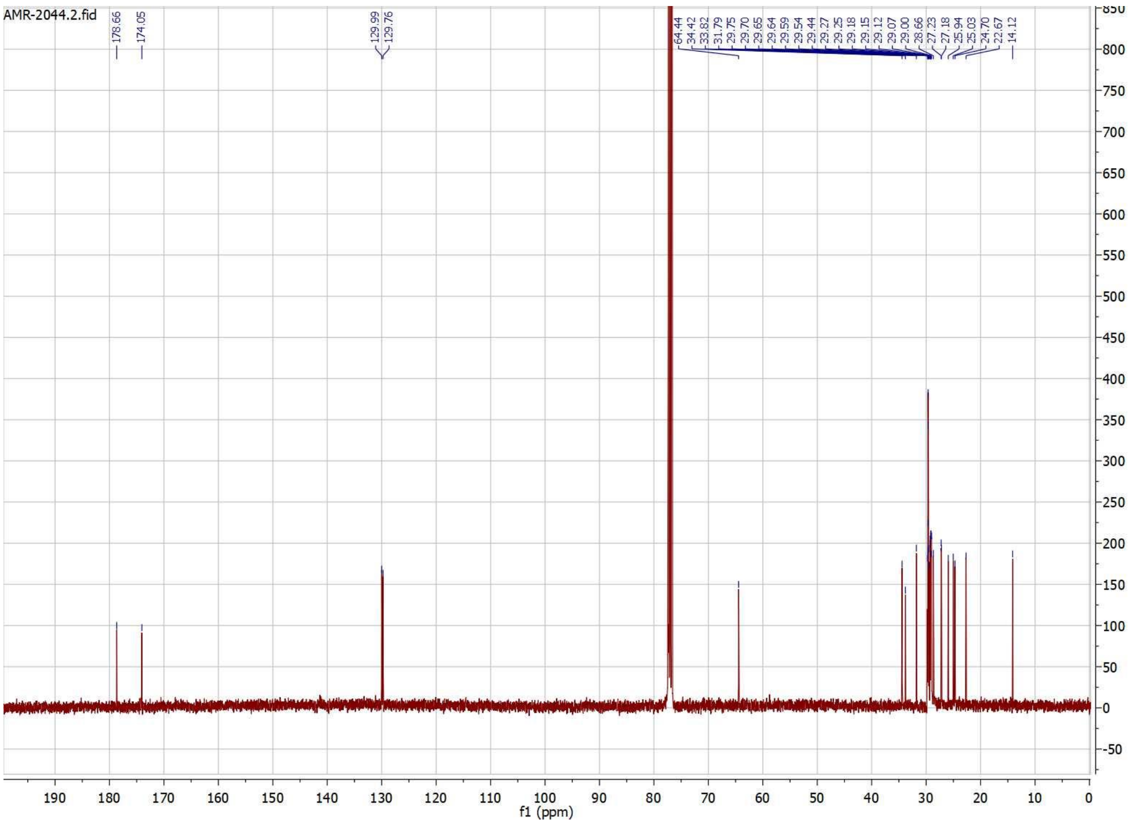

Figure S49. ^{13}C NMR spectrum of compound **25**.

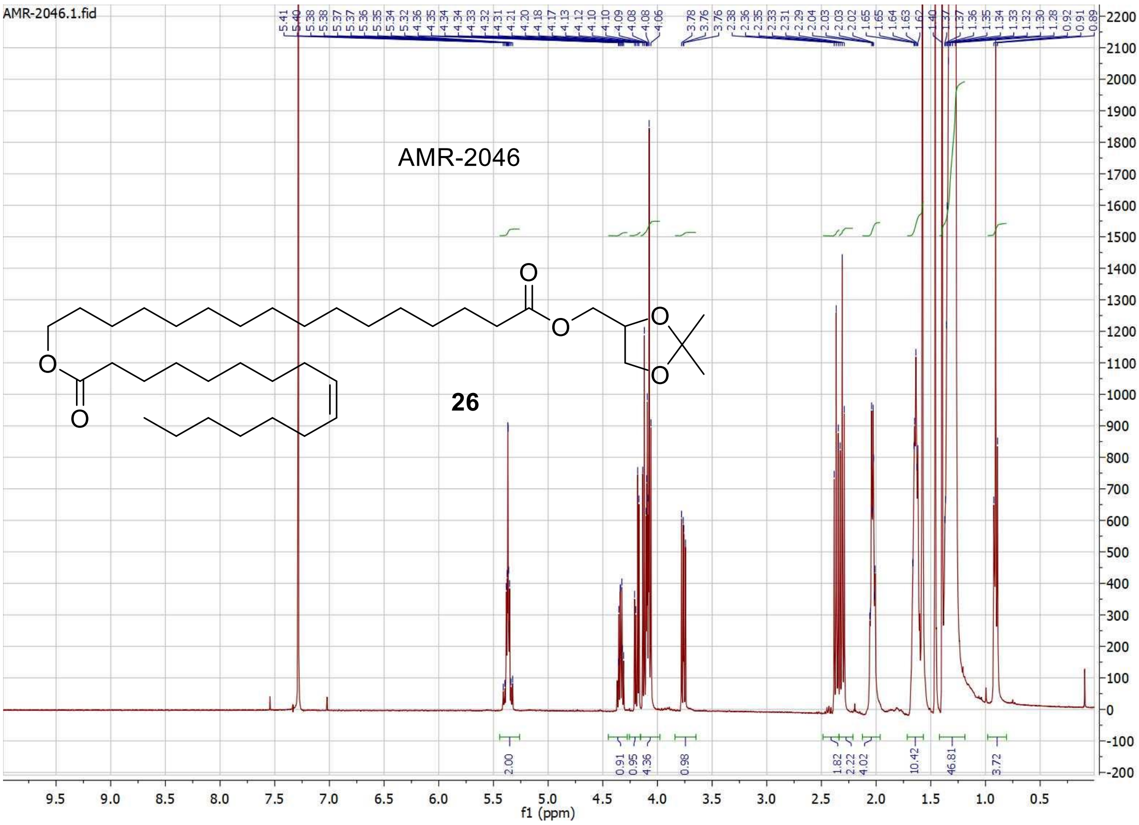

Figure S50. ^{1}H NMR spectrum of compound **26**.

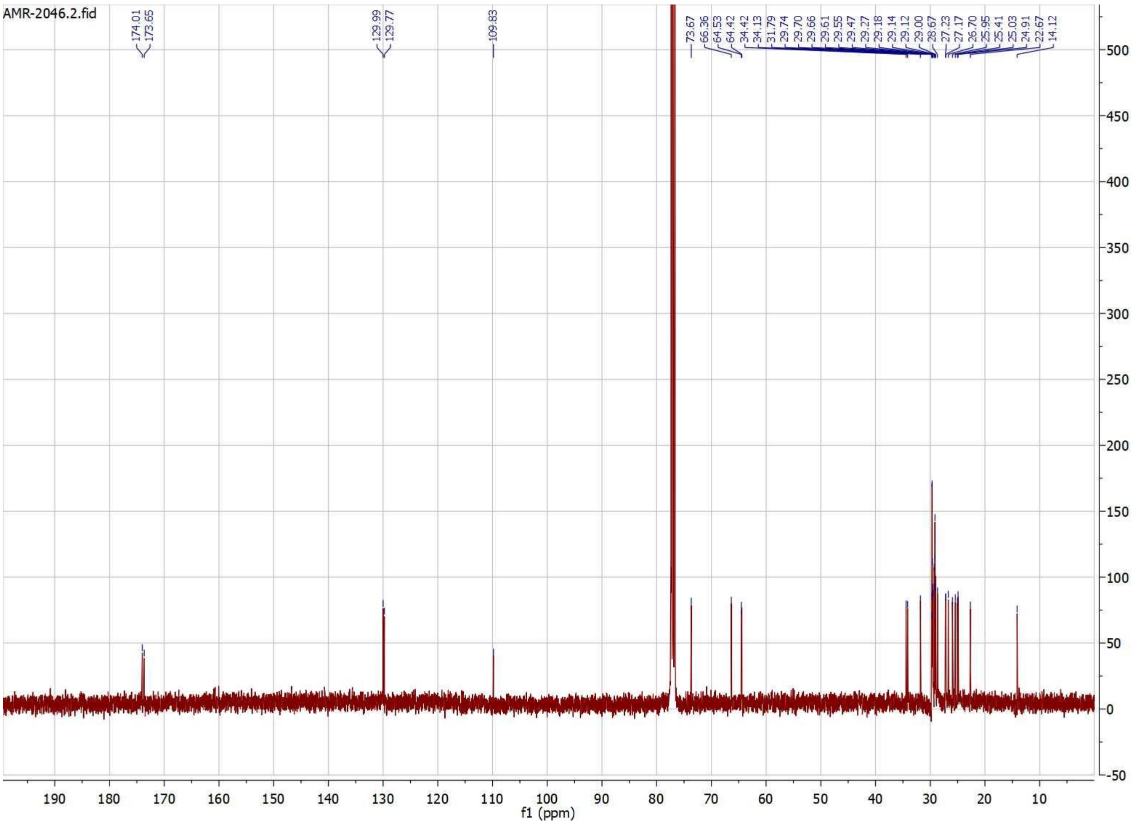

Figure S51. ^{13}C NMR spectrum of compound **26**.

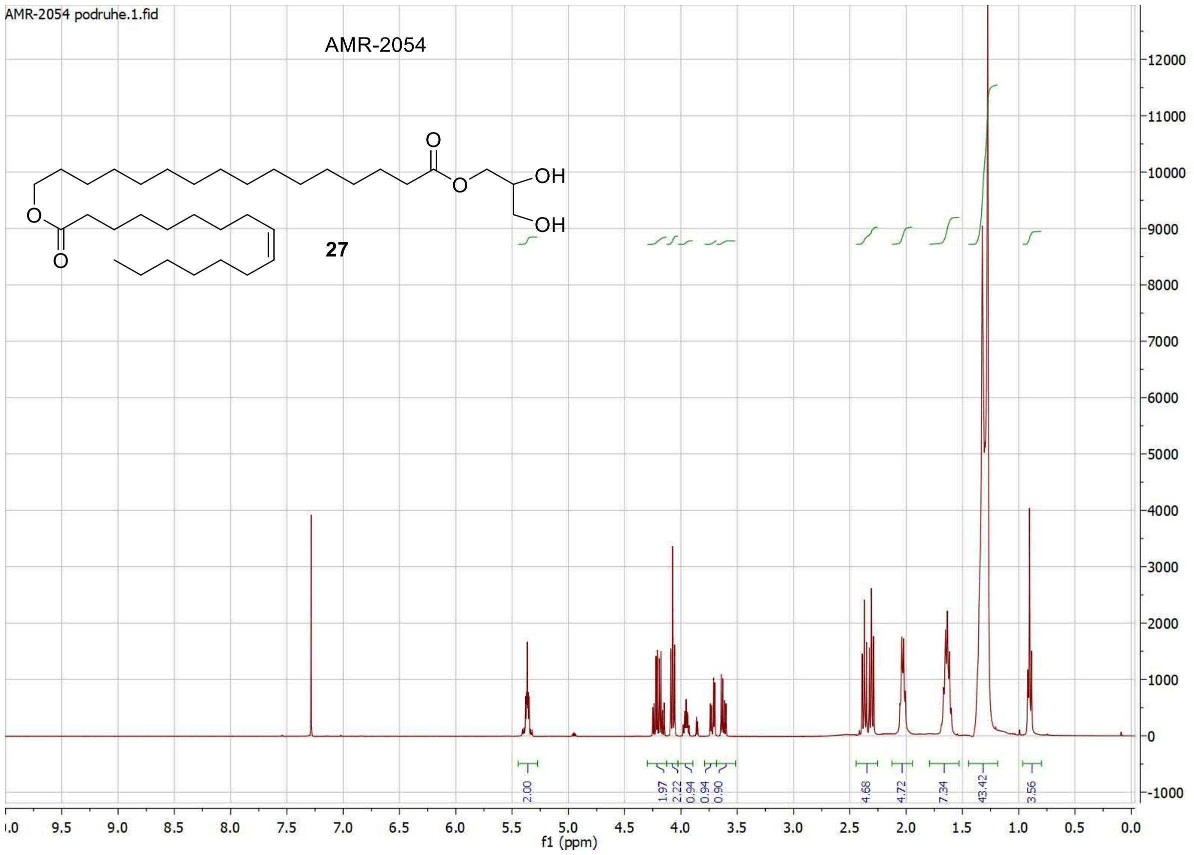

Figure S52. ^{1}H NMR spectrum of compound **27**.

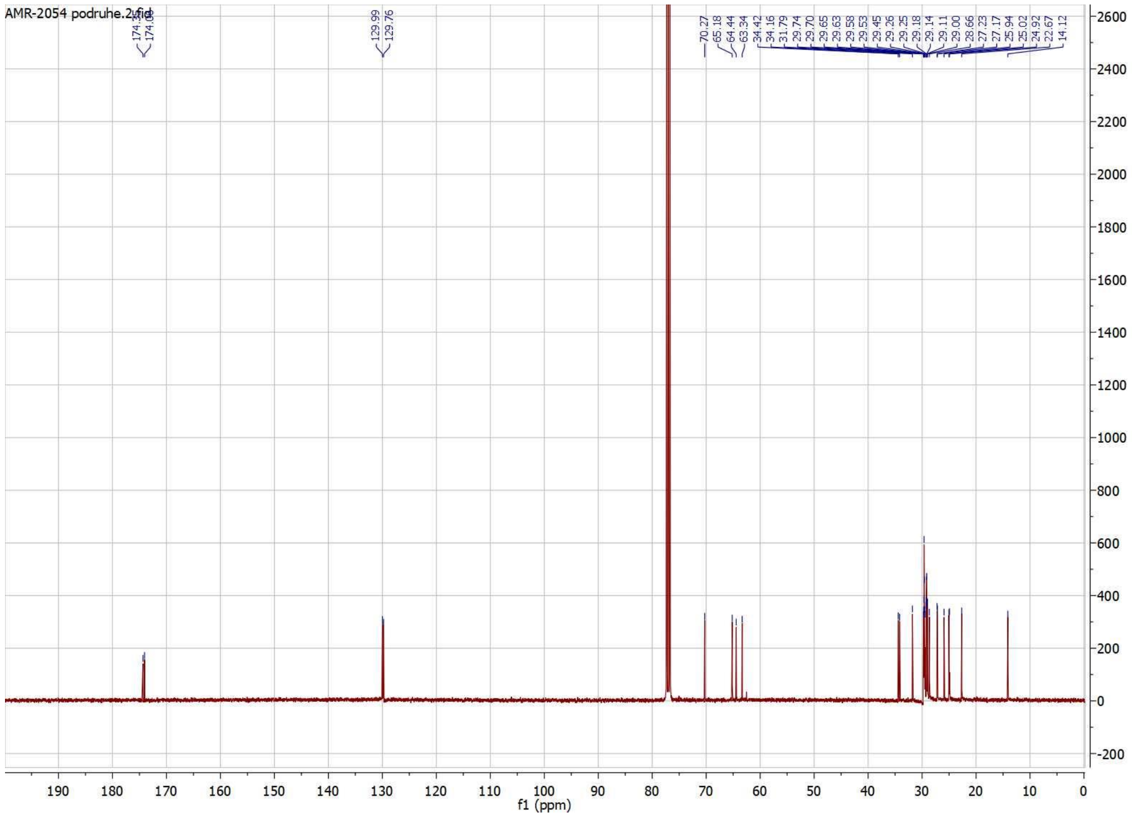

Figure S53. ^{13}C NMR spectrum of compound **27**.

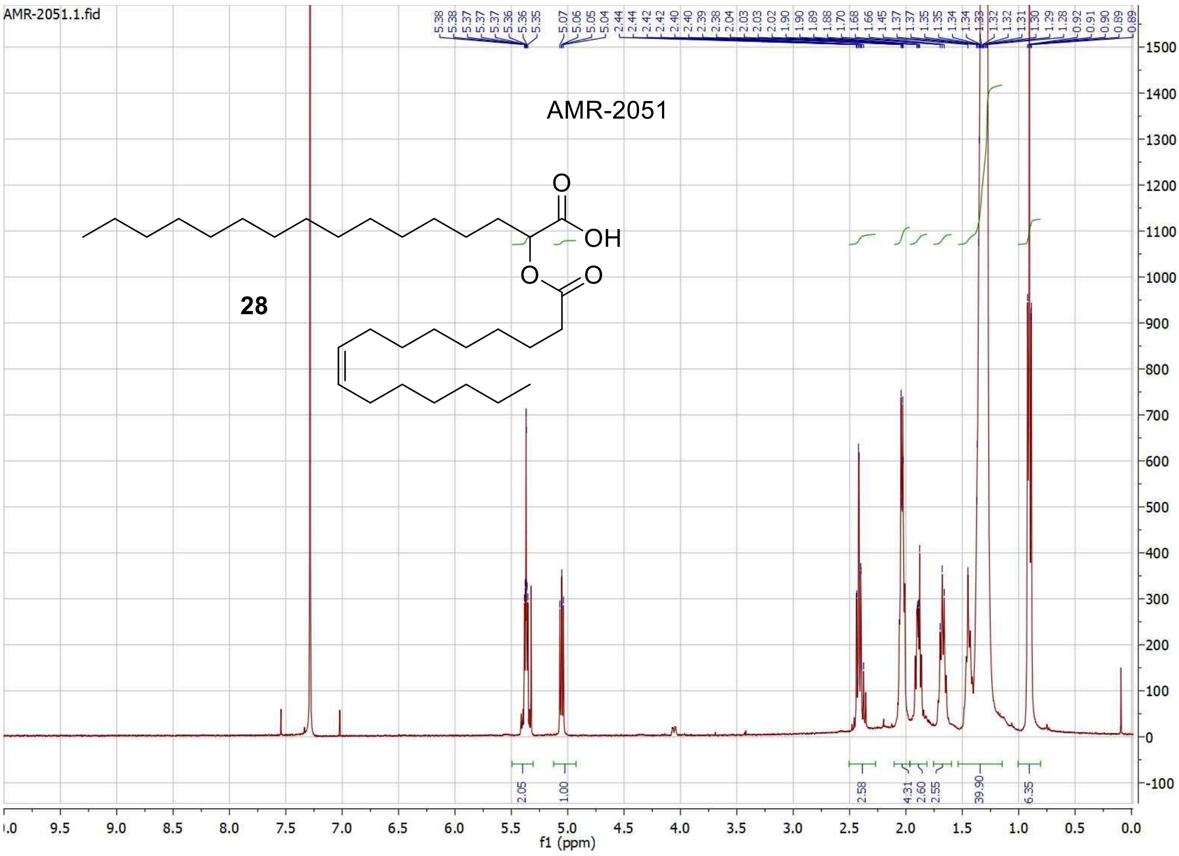

Figure S54. ^{1}H NMR spectrum of compound **28**.

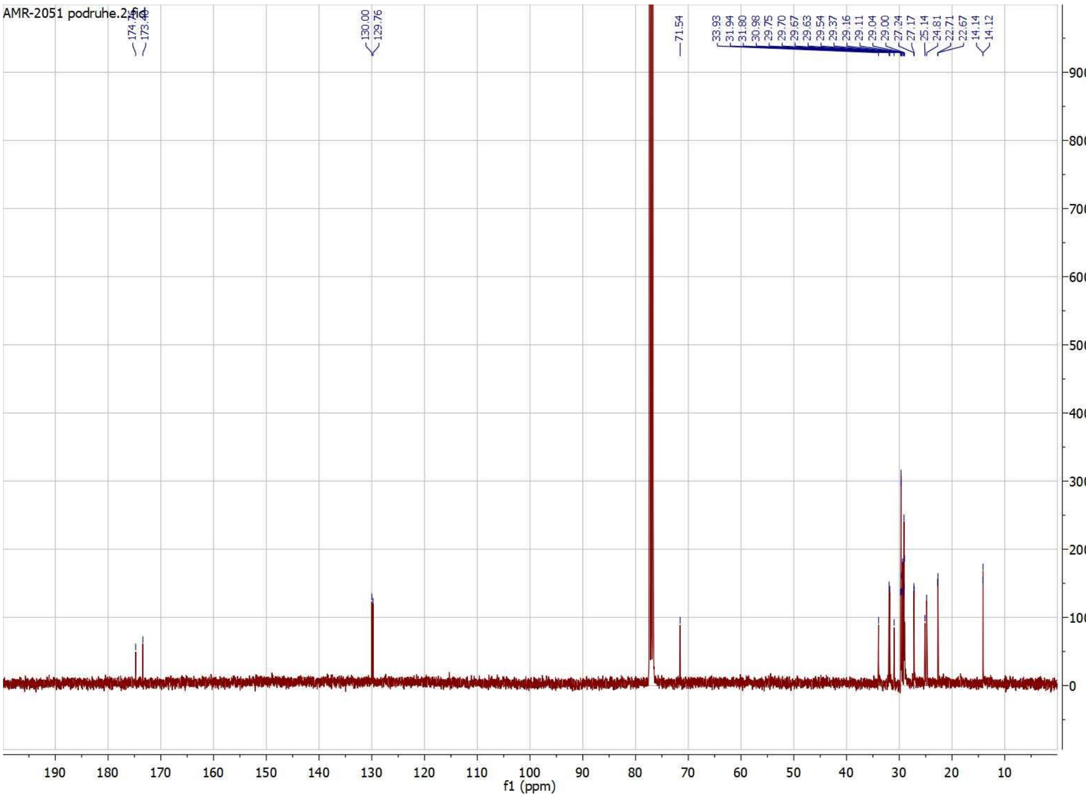

Figure S55. ^{13}C NMR spectrum of compound **28**.

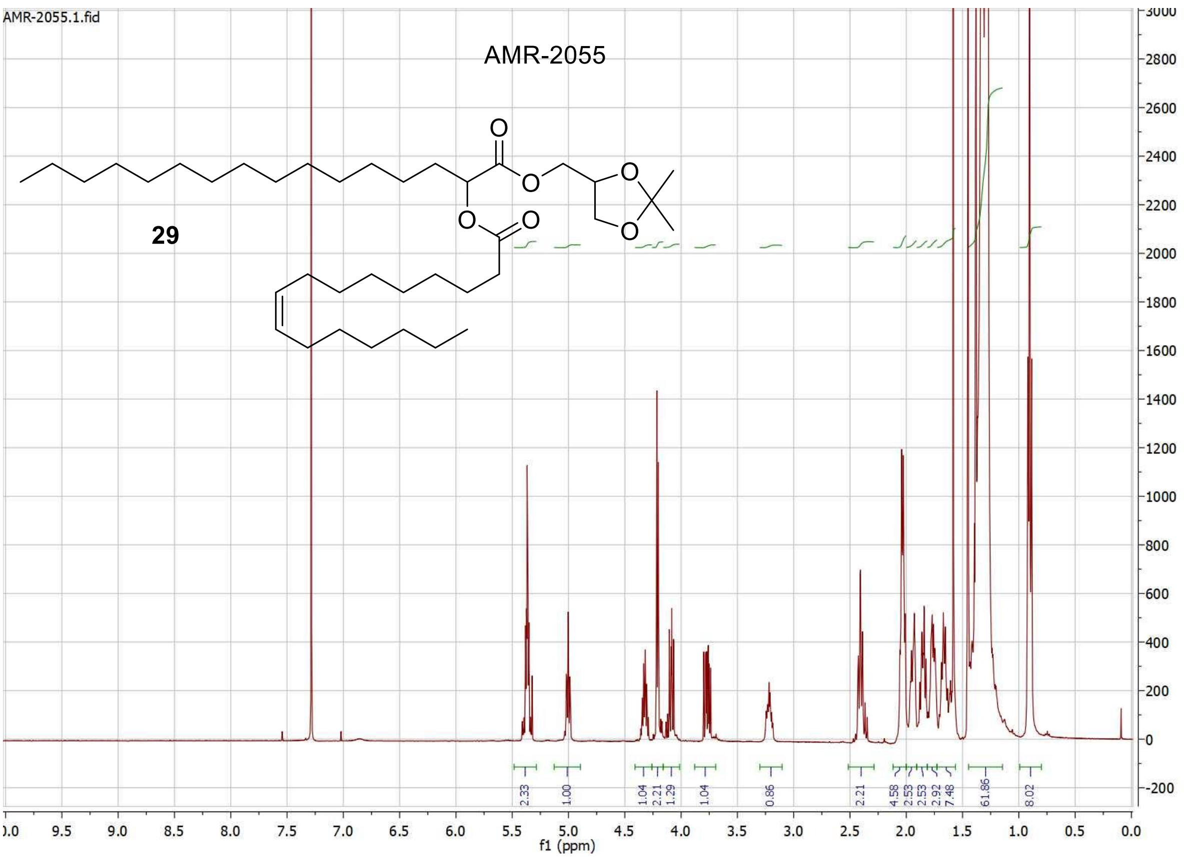

Figure S56. ^{1}H NMR spectrum of compound **29**.

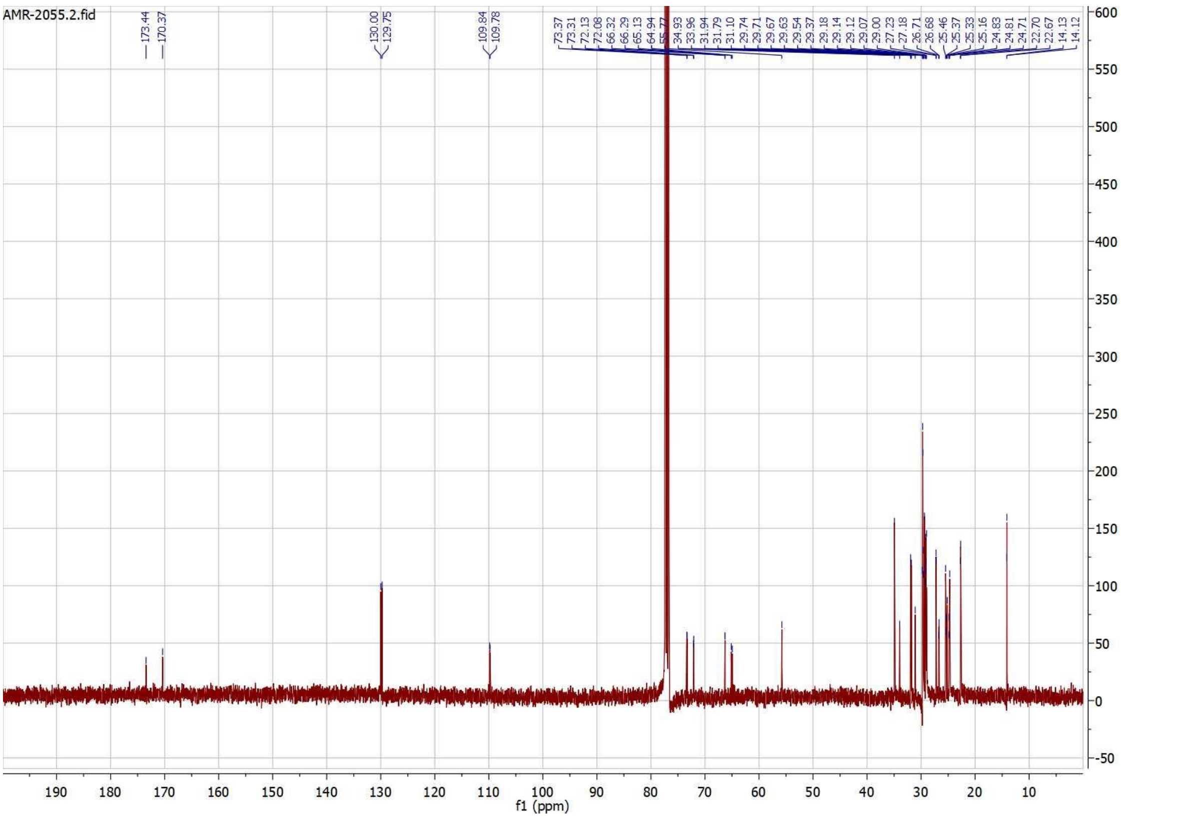

Figure S57. ^{13}C NMR spectrum of compound **29**.

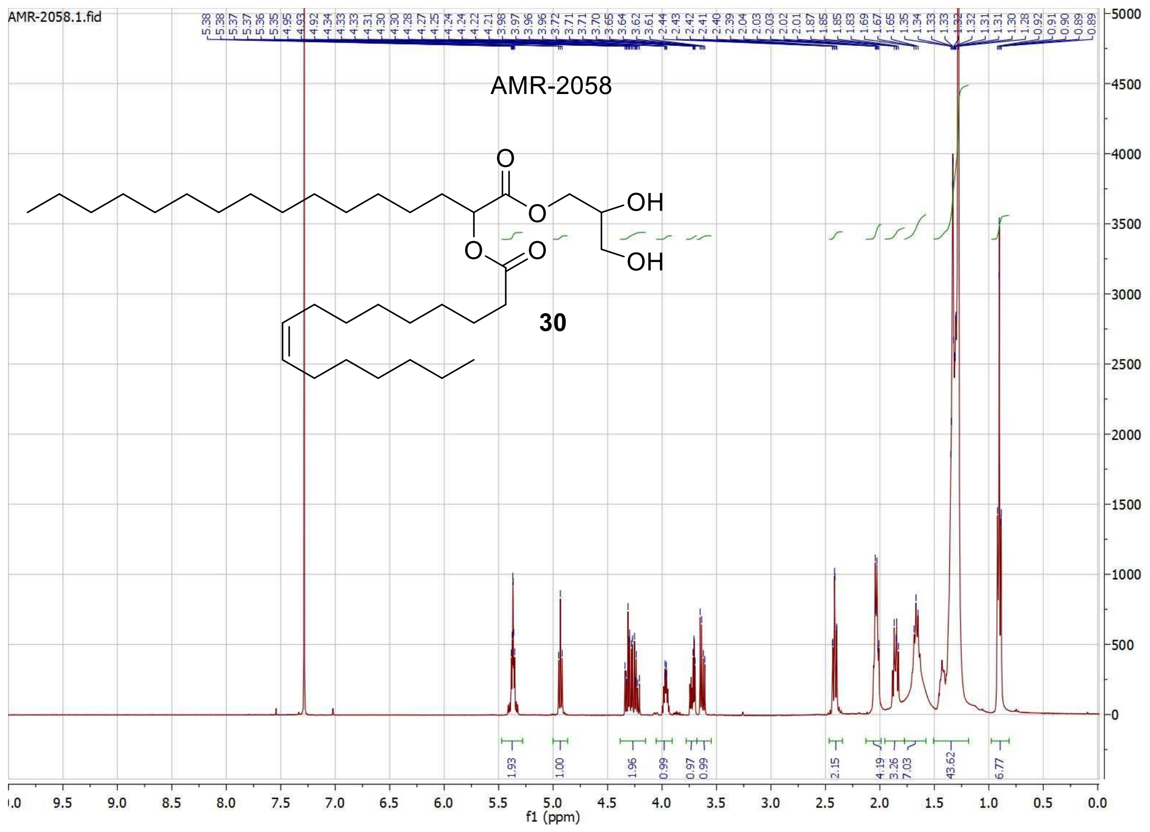

Figure S58. ^{1}H NMR spectrum of compound **30**.

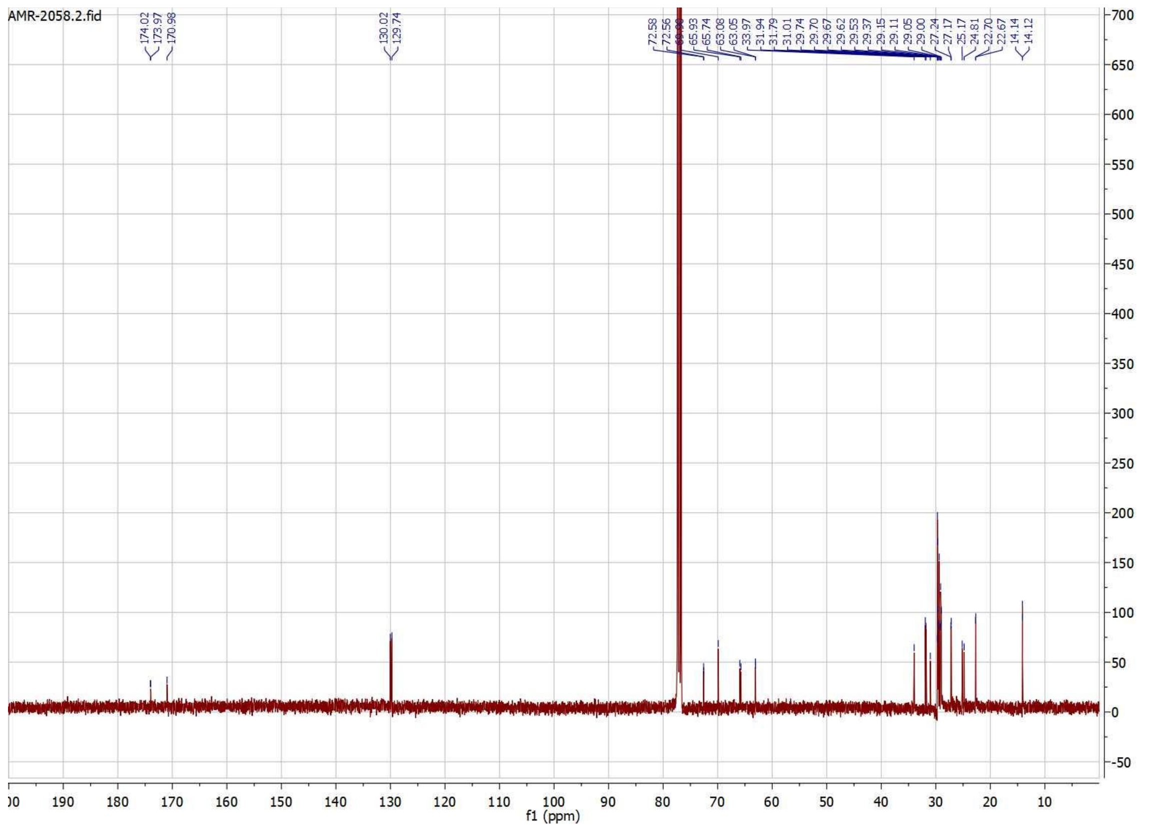

Figure S59. ^{13}C NMR spectrum of compound **30**.

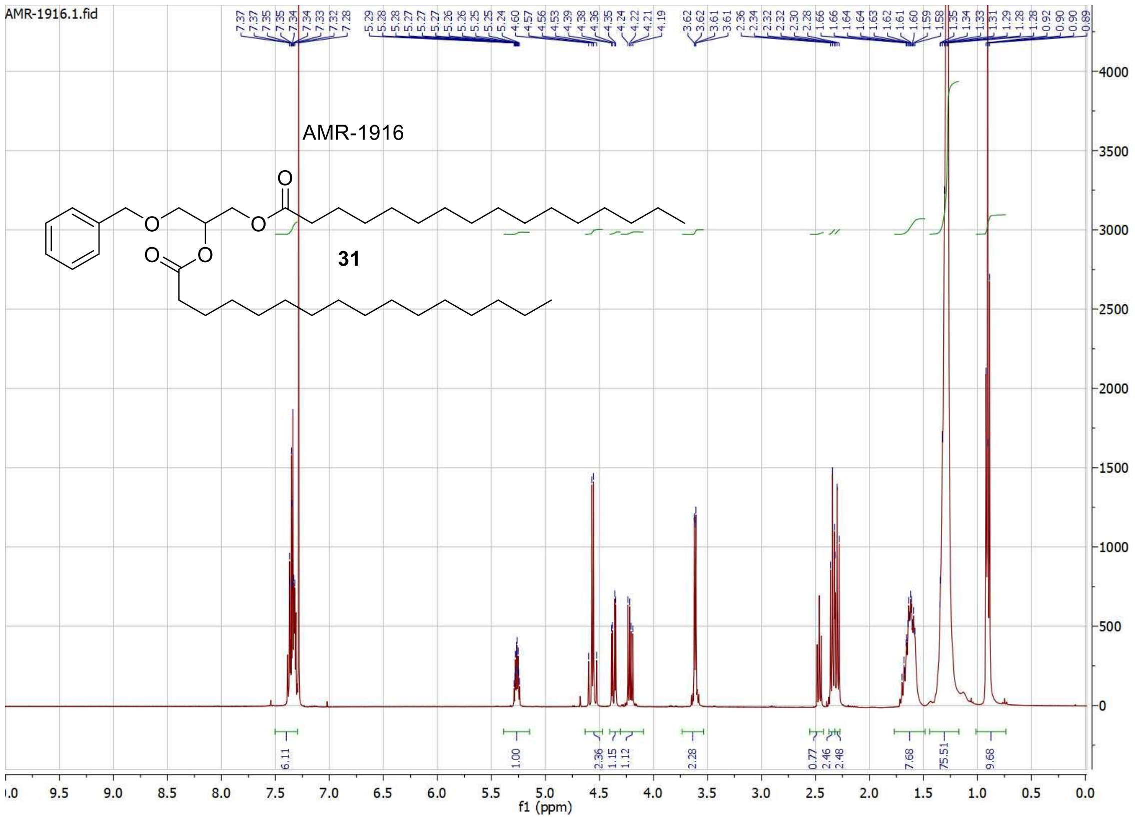

Figure S60. ^{1}H NMR spectrum of compound **31**.

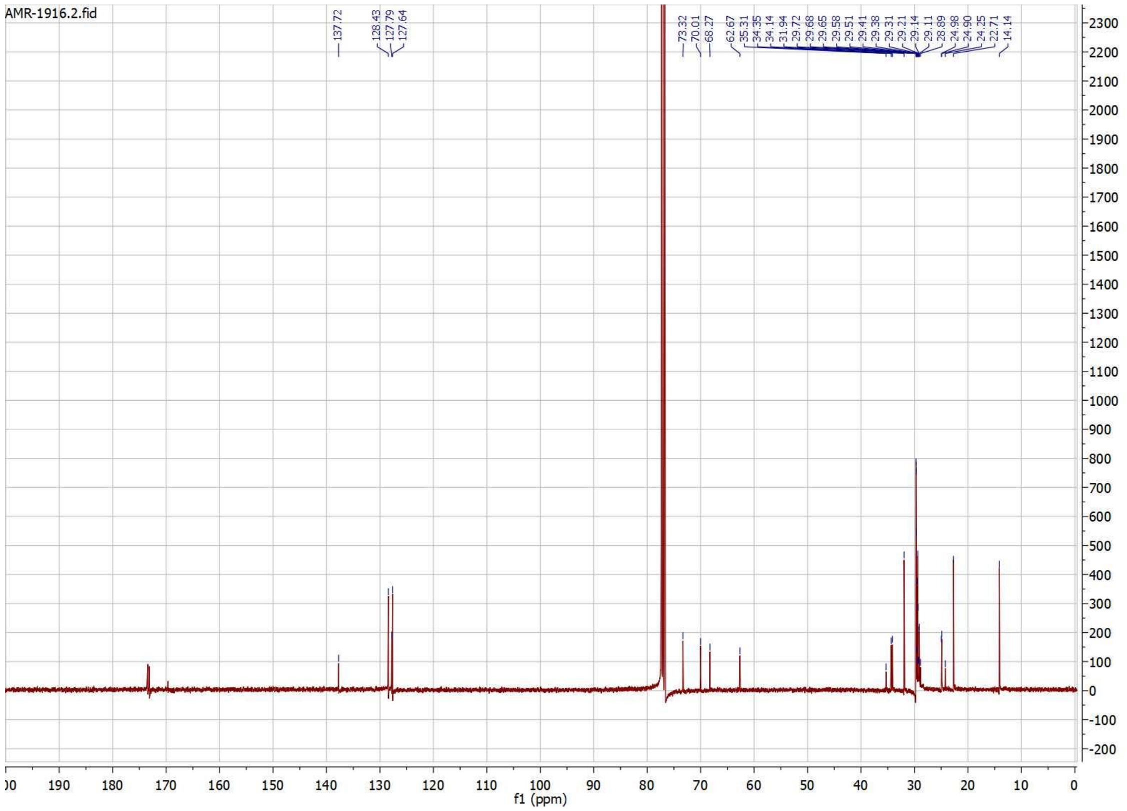

Figure S61. ^{13}C NMR spectrum of compound **31**.

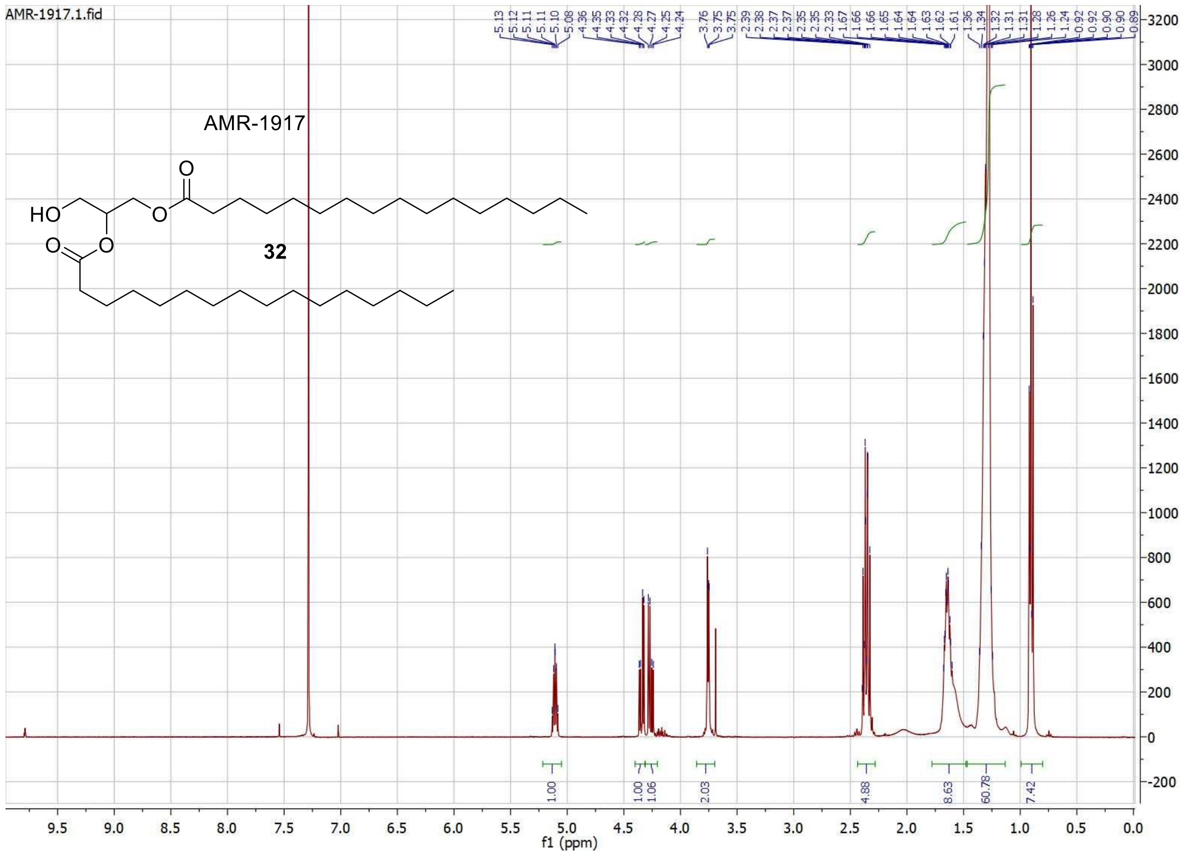

Figure S62. ^{1}H NMR spectrum of compound **32**.

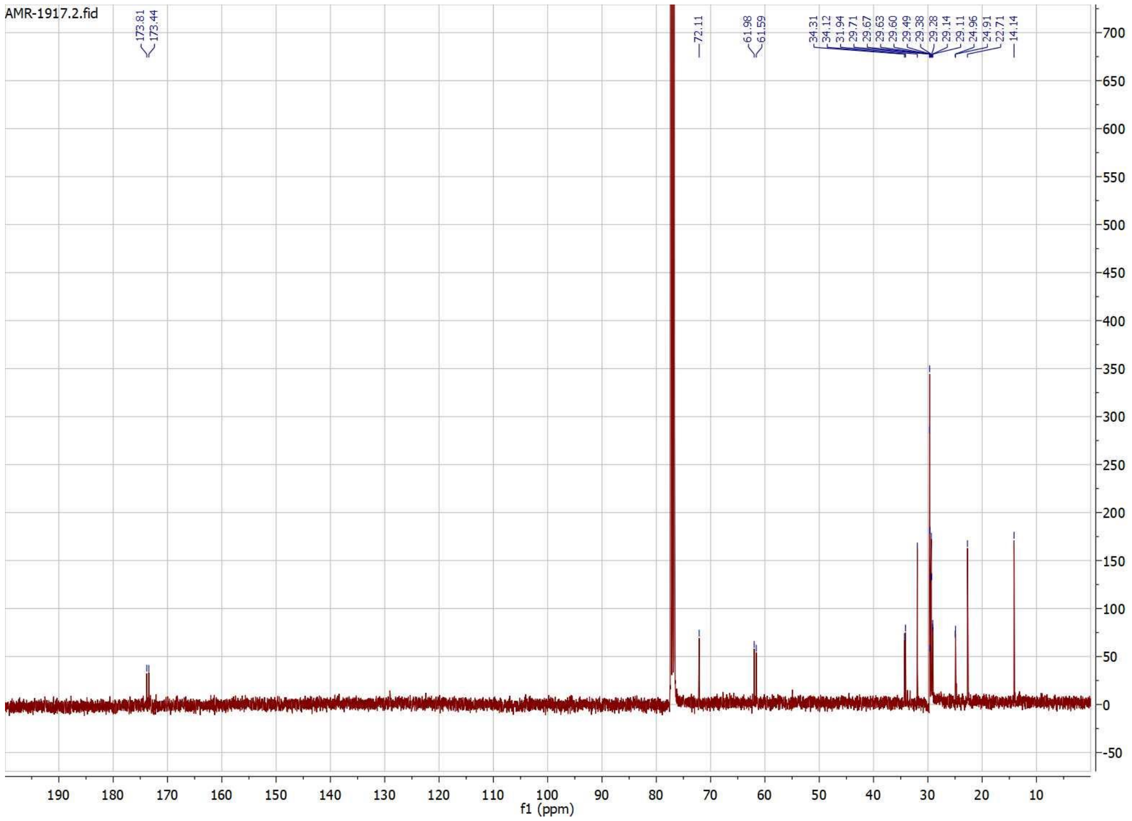

Figure S63. ^{13}C NMR spectrum of compound **32**.

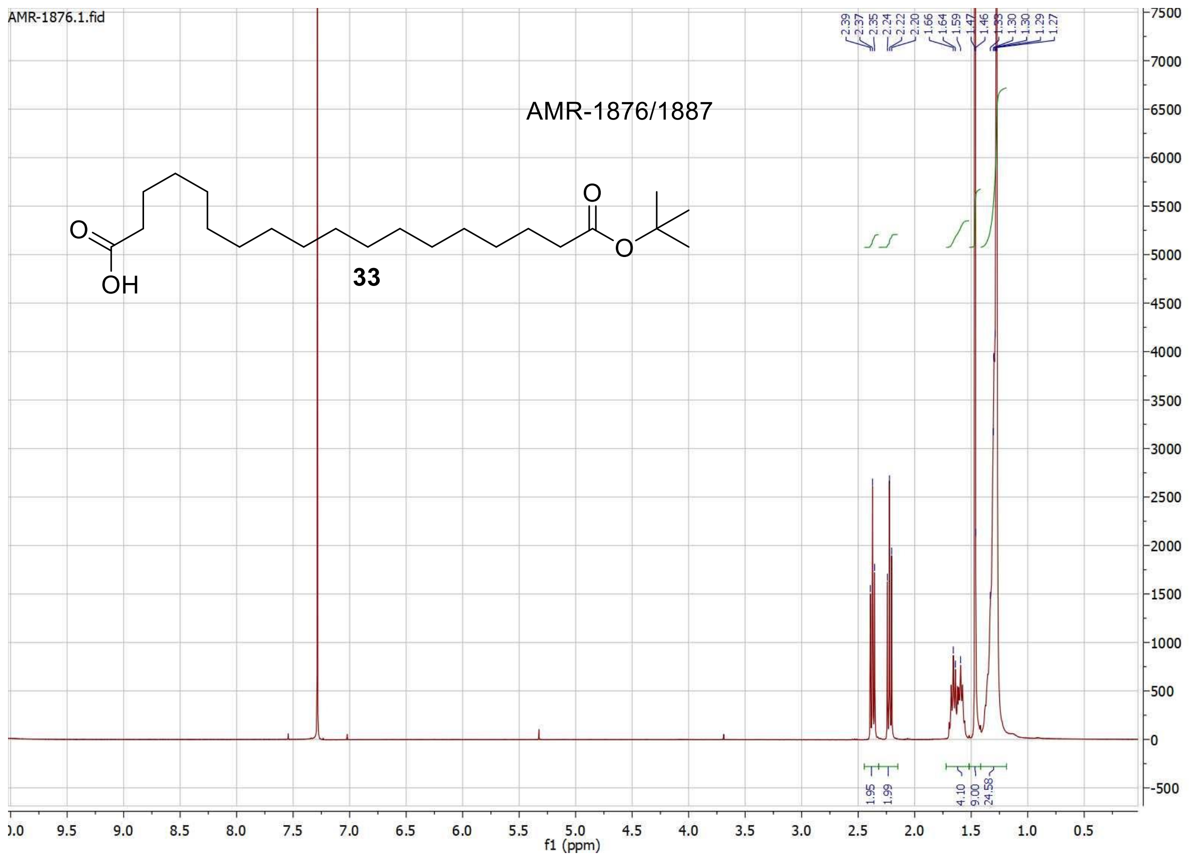

Figure S64. ^{1}H NMR spectrum of compound **33**.

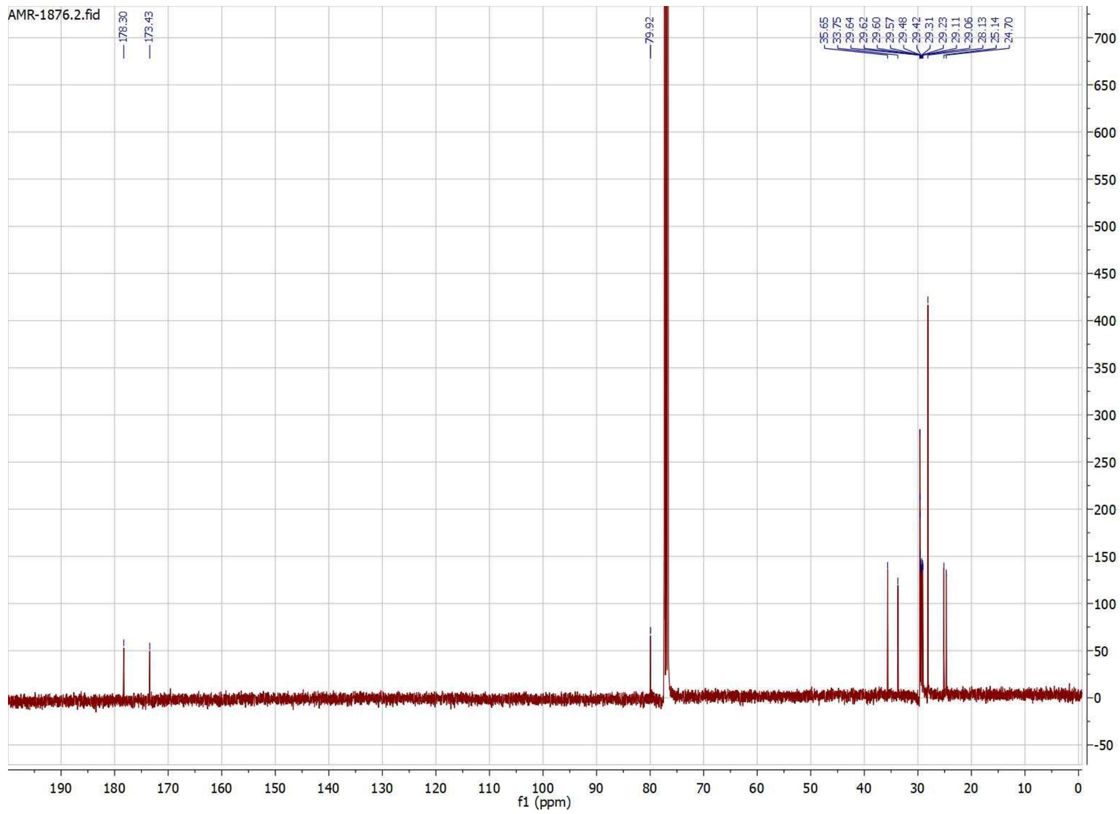

Figure S65. ^{13}C NMR spectrum of compound **33**.

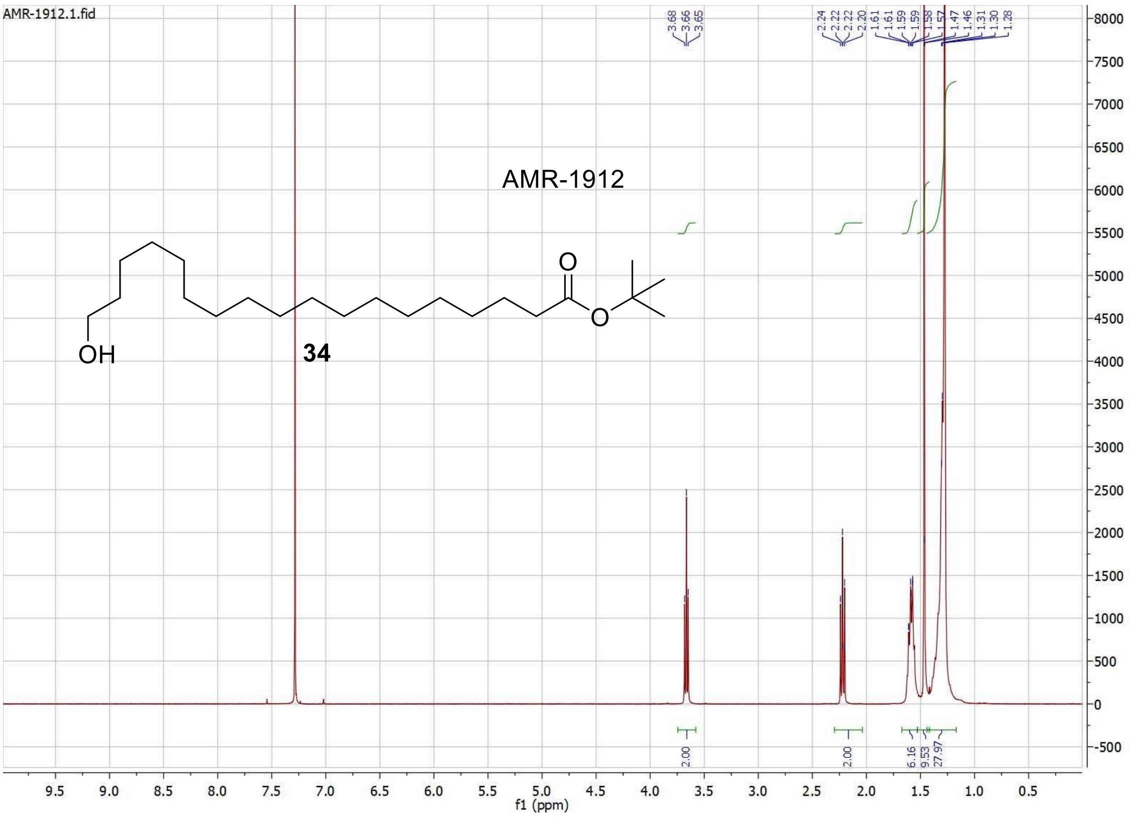

Figure S66. ^{1}H NMR spectrum of compound **34**.

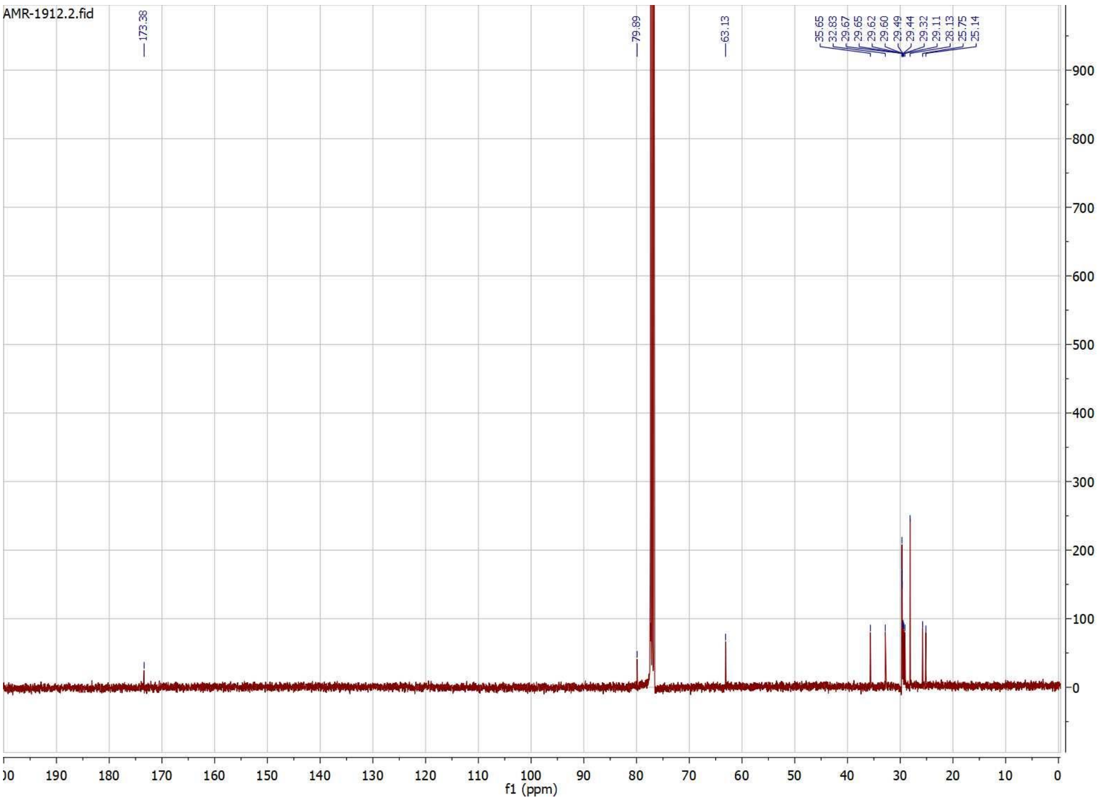

Figure S67. ^{13}C NMR spectrum of compound **34**.

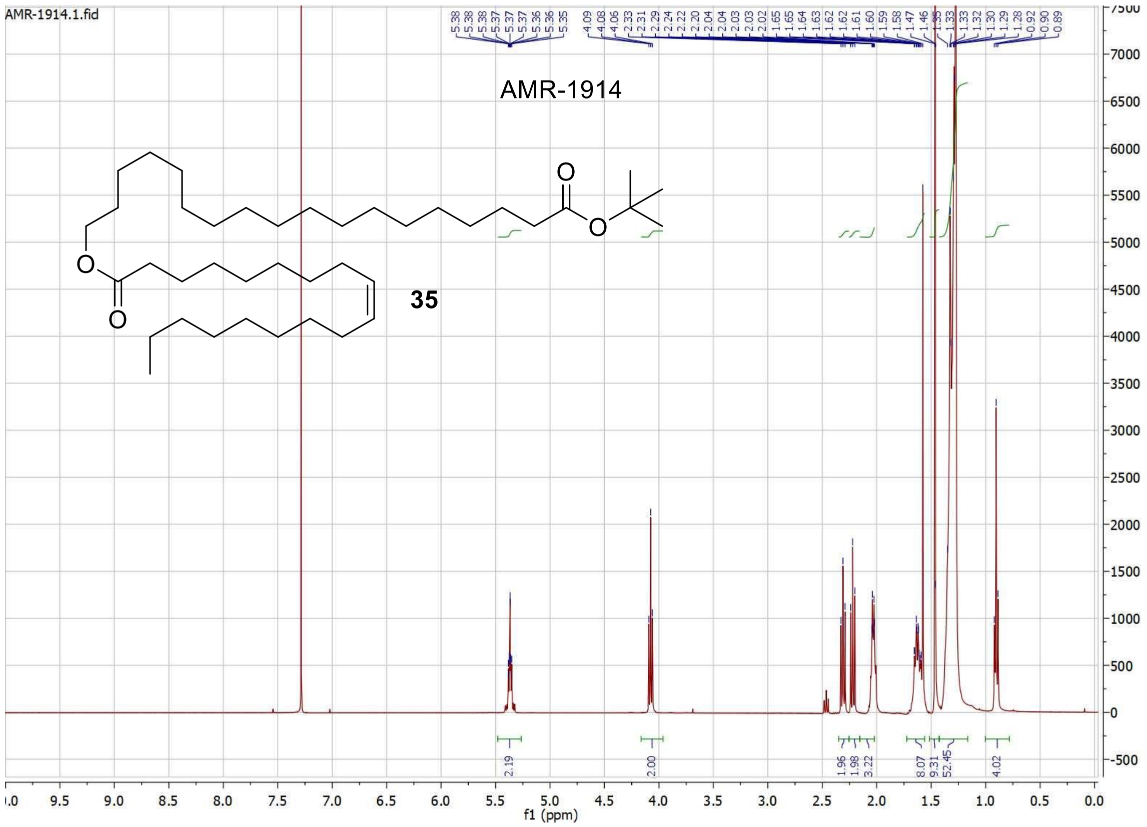

Figure S68. ^{1}H NMR spectrum of compound **35**.

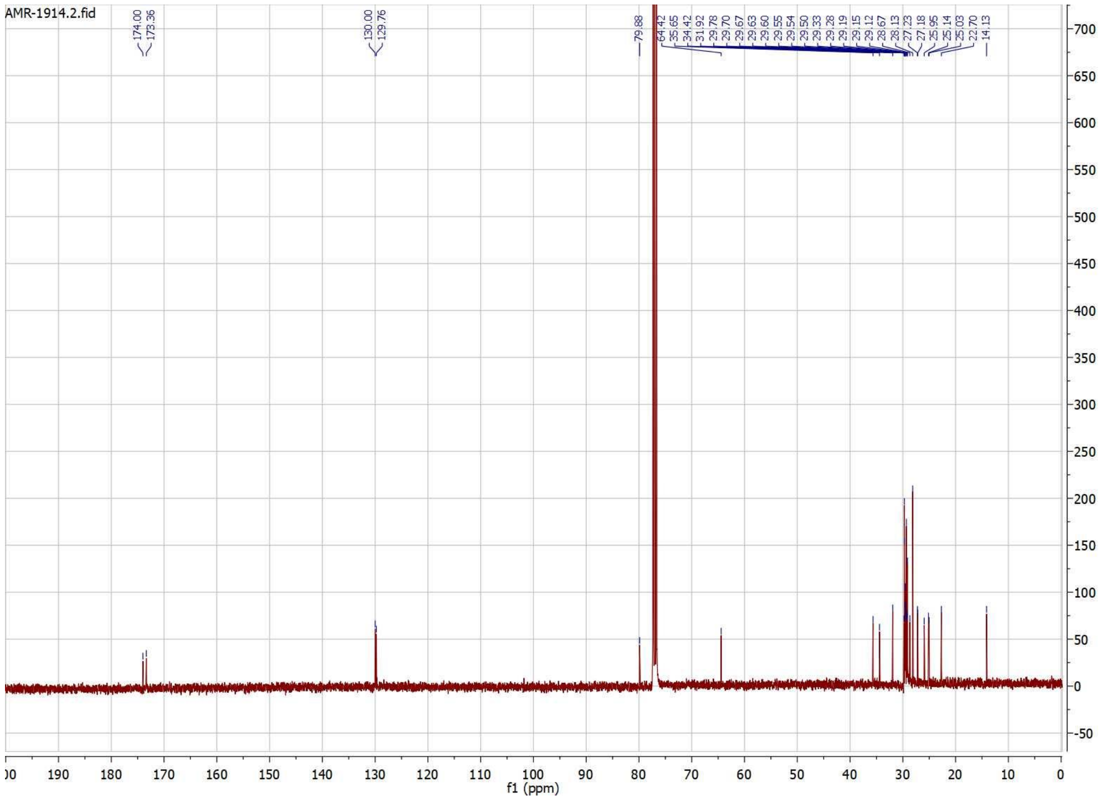

Figure S69. ^{13}C NMR spectrum of compound **35**.

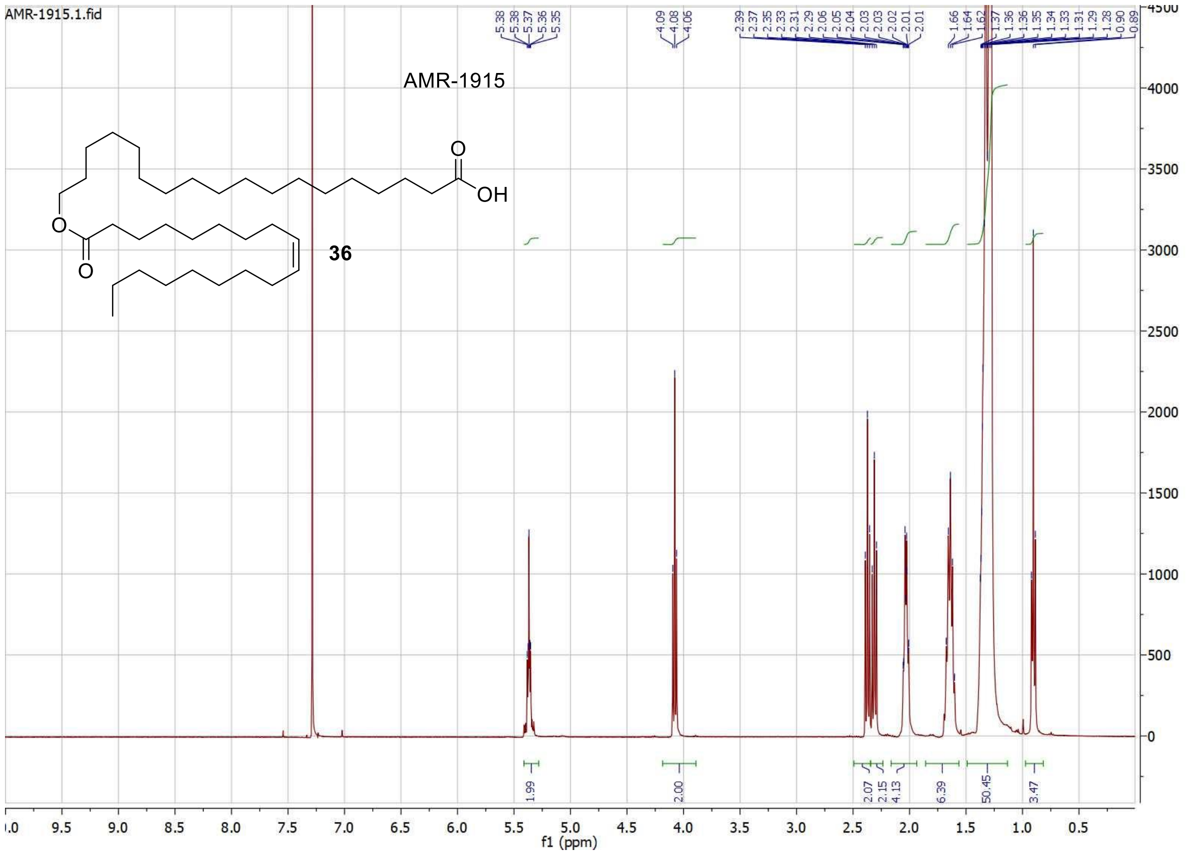

Figure S70. ^{1}H NMR spectrum of compound **36**.

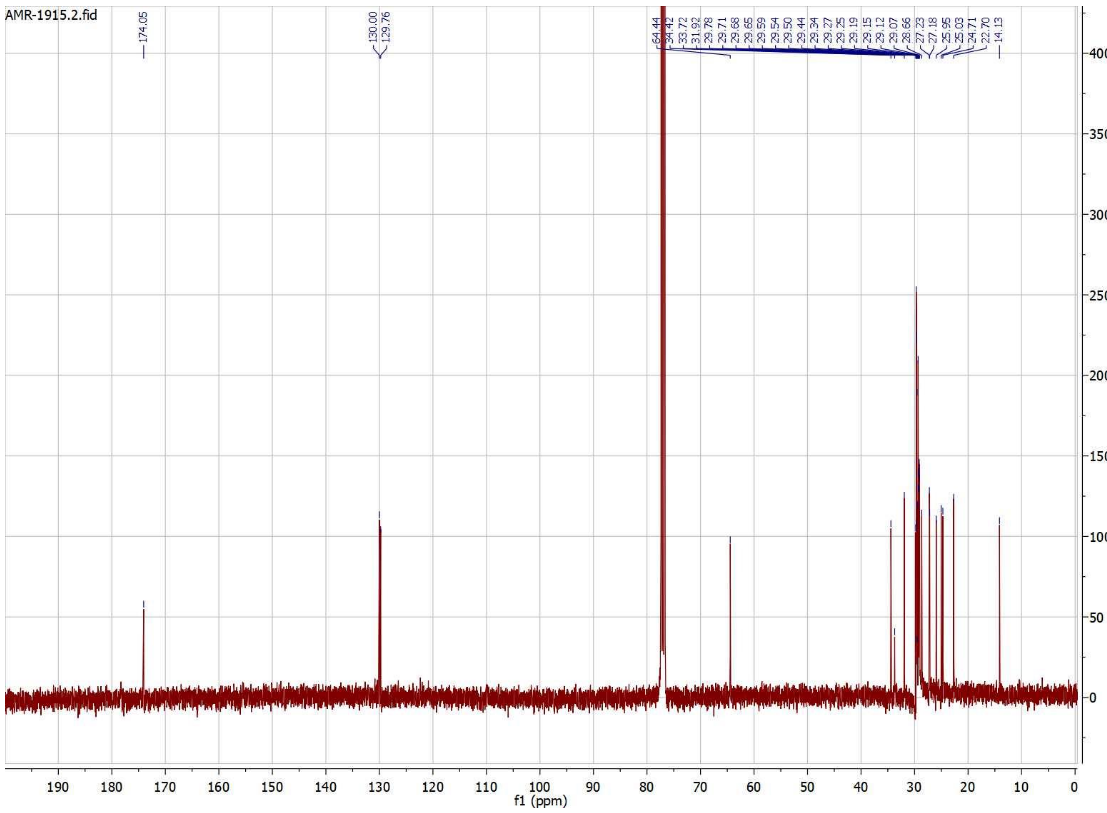

Figure S71. ^{13}C NMR spectrum of compound **36**.

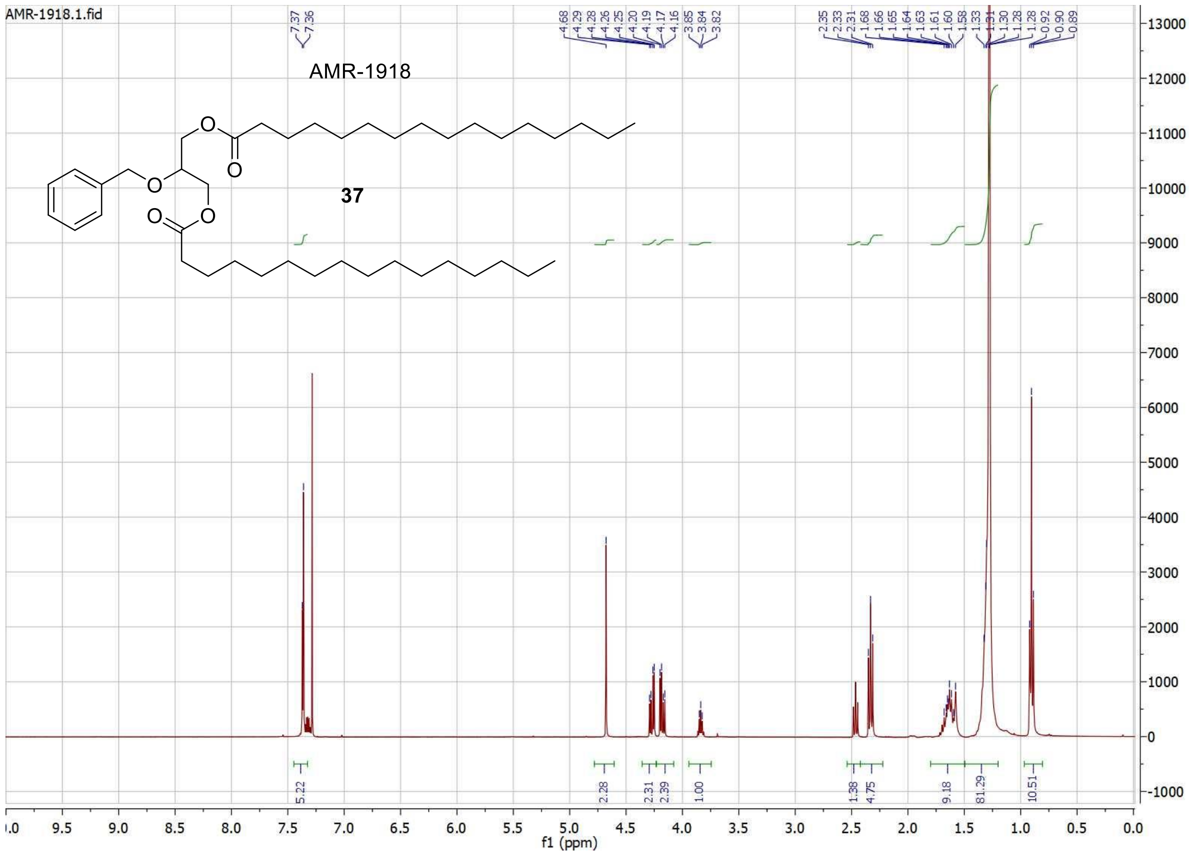

Figure S72. ^{1}H NMR spectrum of compound **37**.

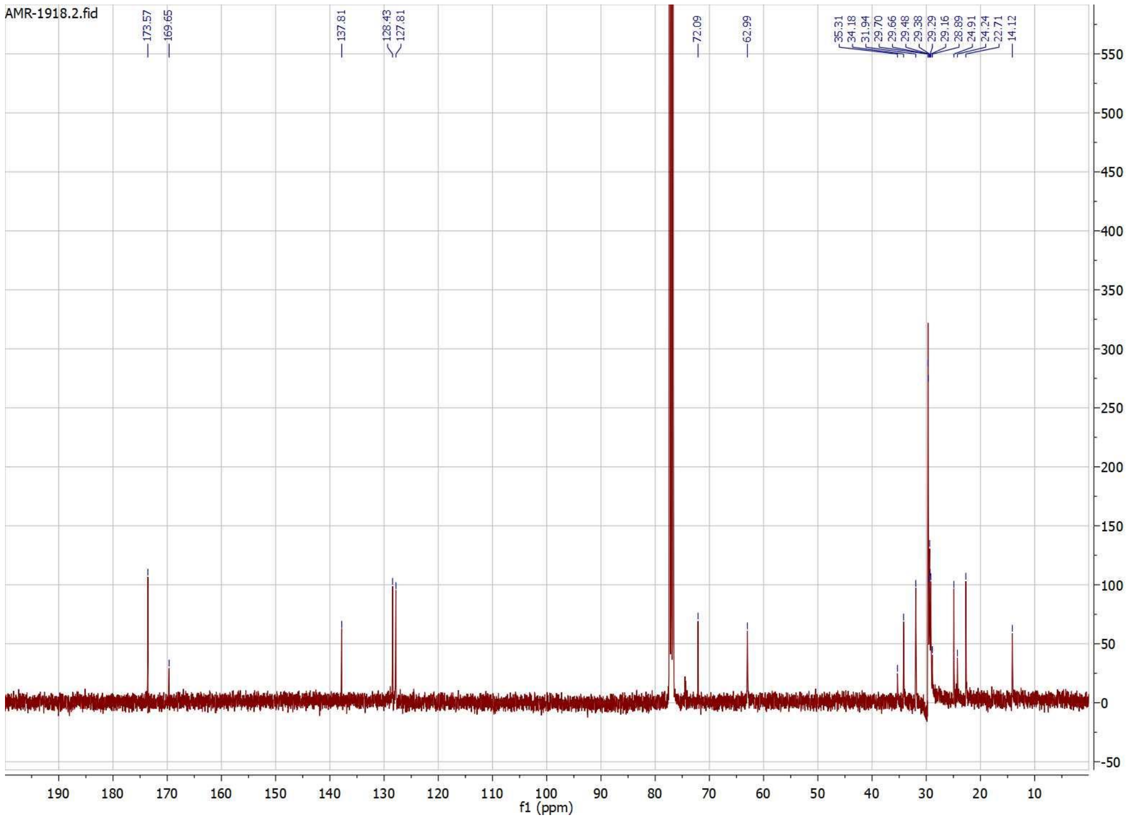

Figure S73. ^{13}C NMR spectrum of compound **37**.

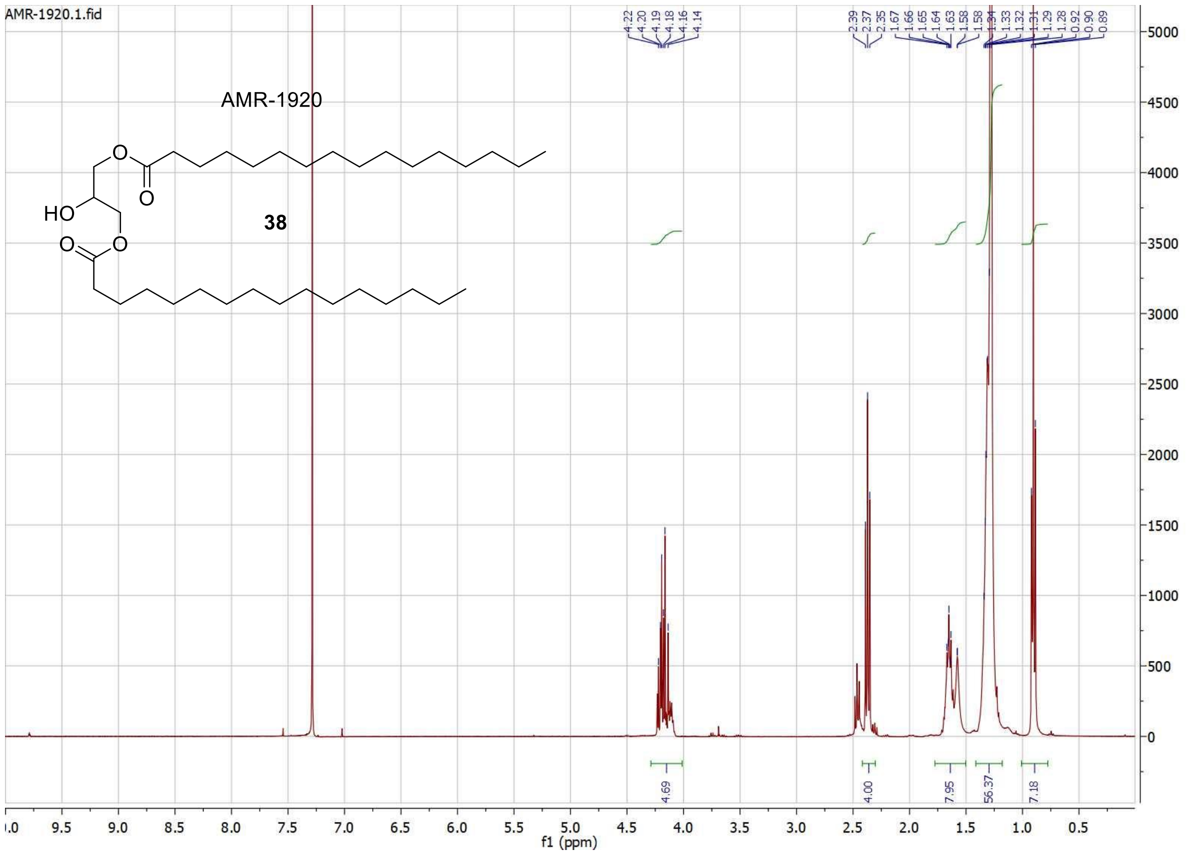

Figure S74. ^{1}H NMR spectrum of compound **38**.

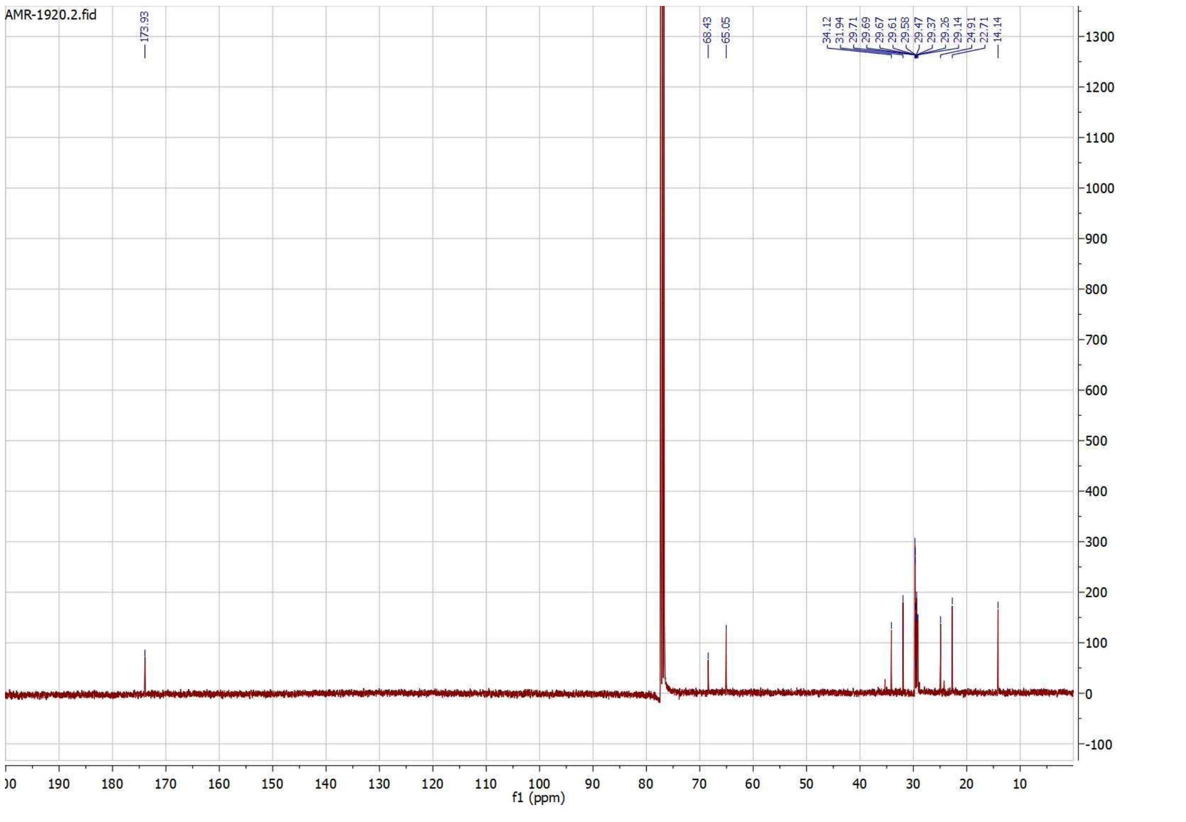

Figure S75. ^{13}C NMR spectrum of compound **38**.

2 Energy-resolved dissociation curves for TG-EST 1–8

2.1 Ammonium adducts

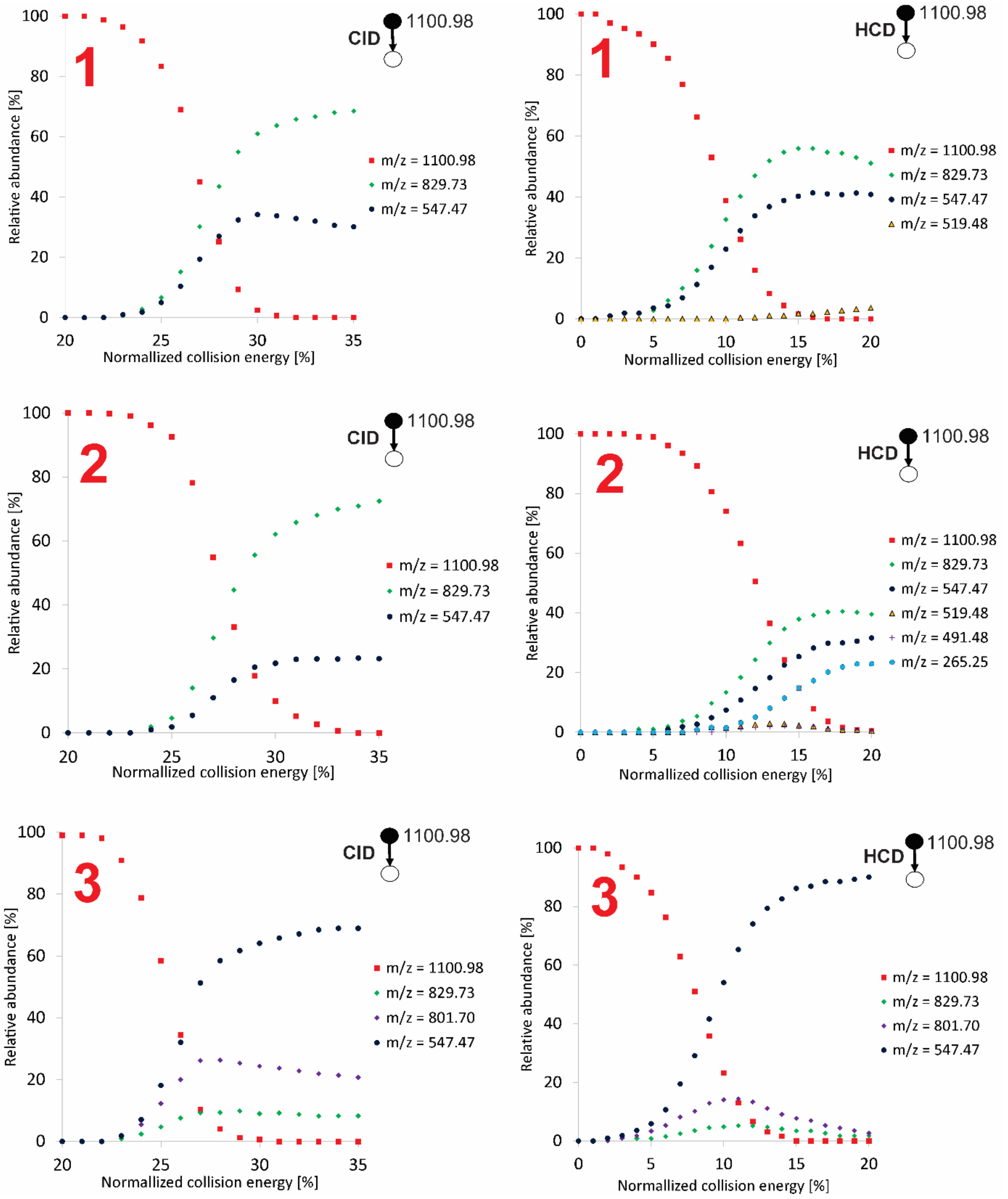

Figure S76. Energy-resolved dissociation curves for $[M + NH_4]^+$ of TG-EST **1–3** obtained by CID (left column) and HCD (right column).

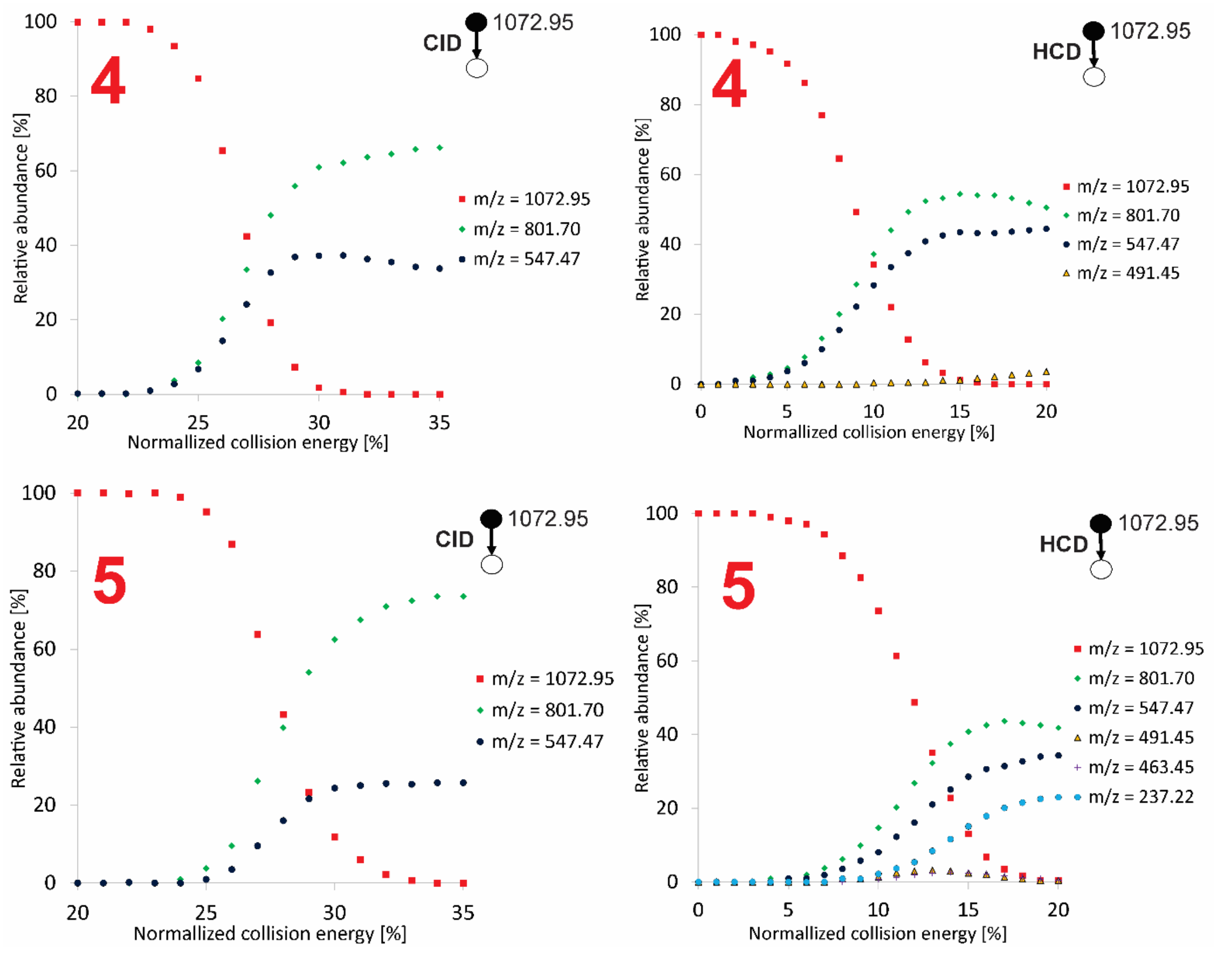

Figure S77. Energy-resolved dissociation curves for $[M + NH_4]^+$ of TG-EST **4** and TG-EST **5** obtained by CID (left column) and HCD (right column).

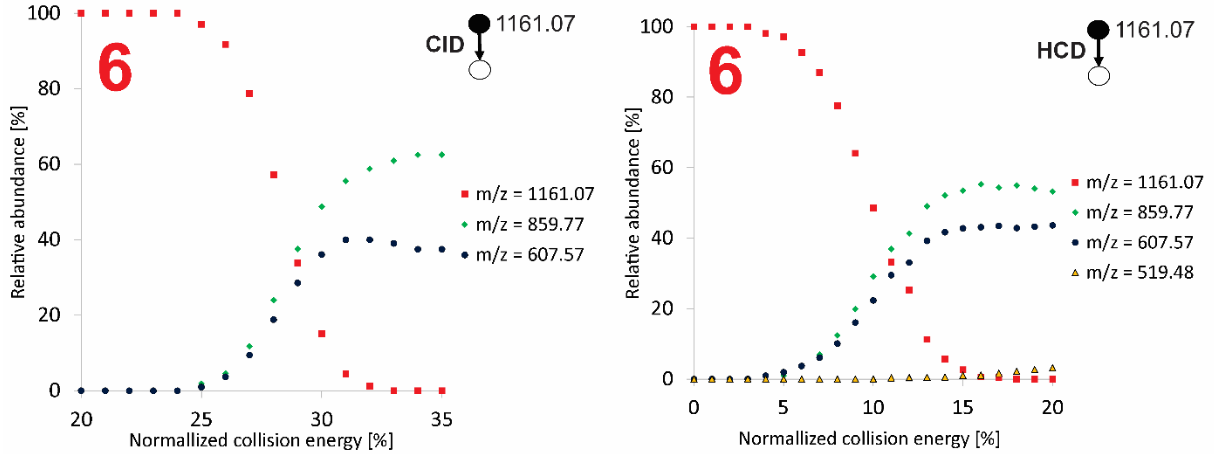

Figure S78. Energy-resolved dissociation curves for $[M + NH_4]^+$ of TG-EST **6** obtained by CID (left column) and HCD (right column).

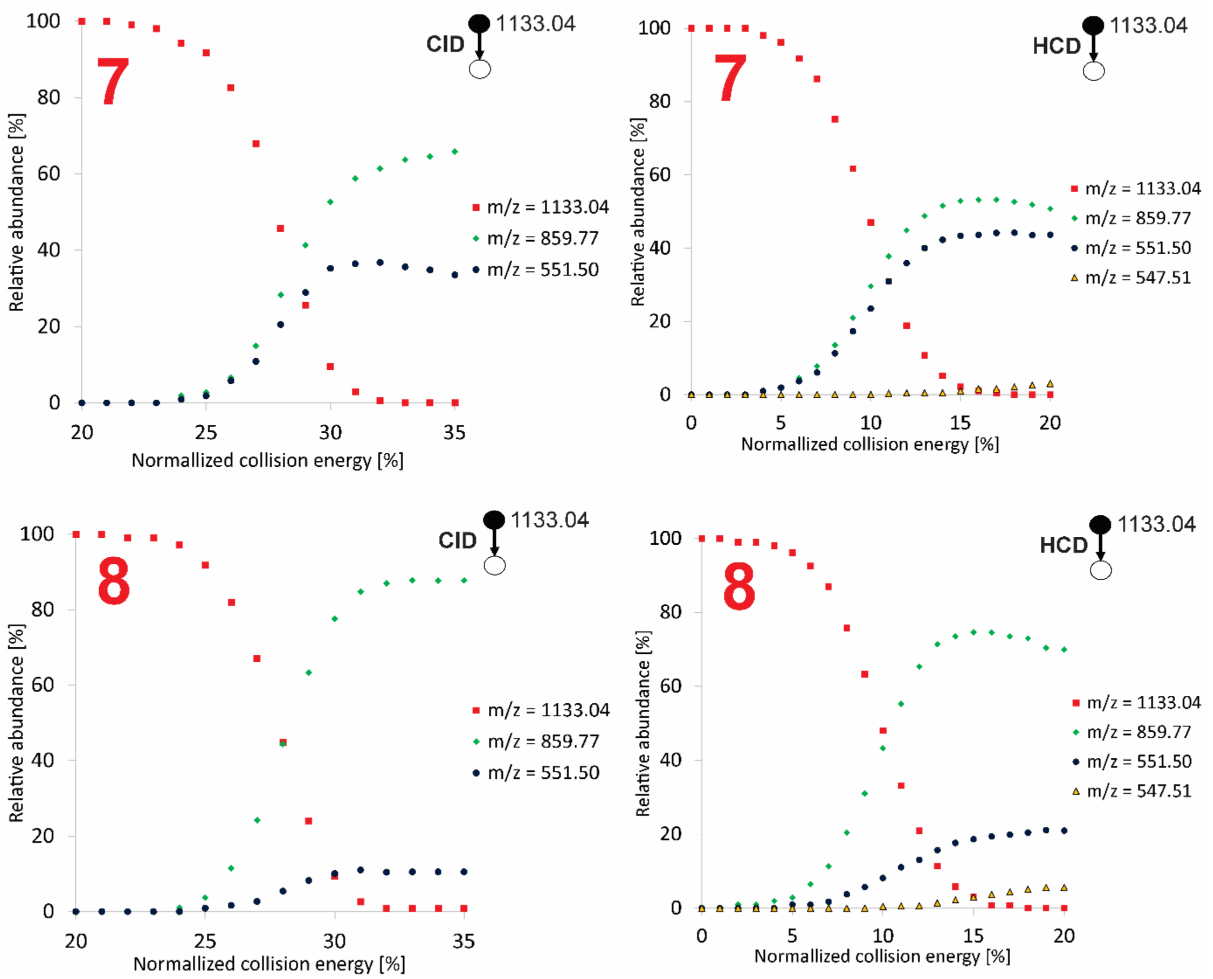

Figure S79. Energy-resolved dissociation curves for $[M + NH_4]^+$ of TG-EST **7** and TG-EST **8** obtained by CID (left column) and HCD (right column).

..

2.2 Lithium adducts

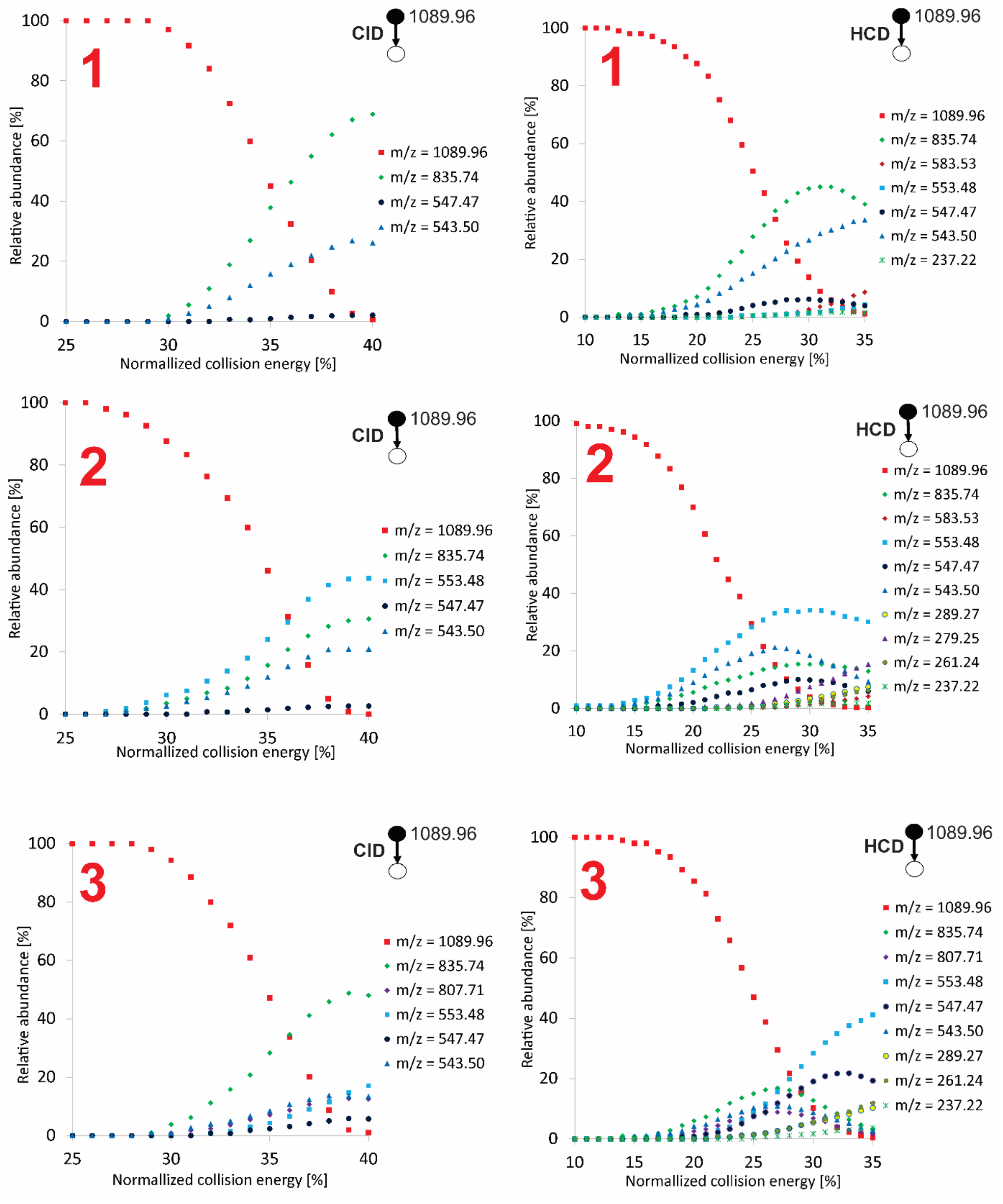

Figure S80. Energy-resolved dissociation curves for [M + Li]$^+$ of TG-EST **1–3** obtained by CID (left column) and HCD (right column).

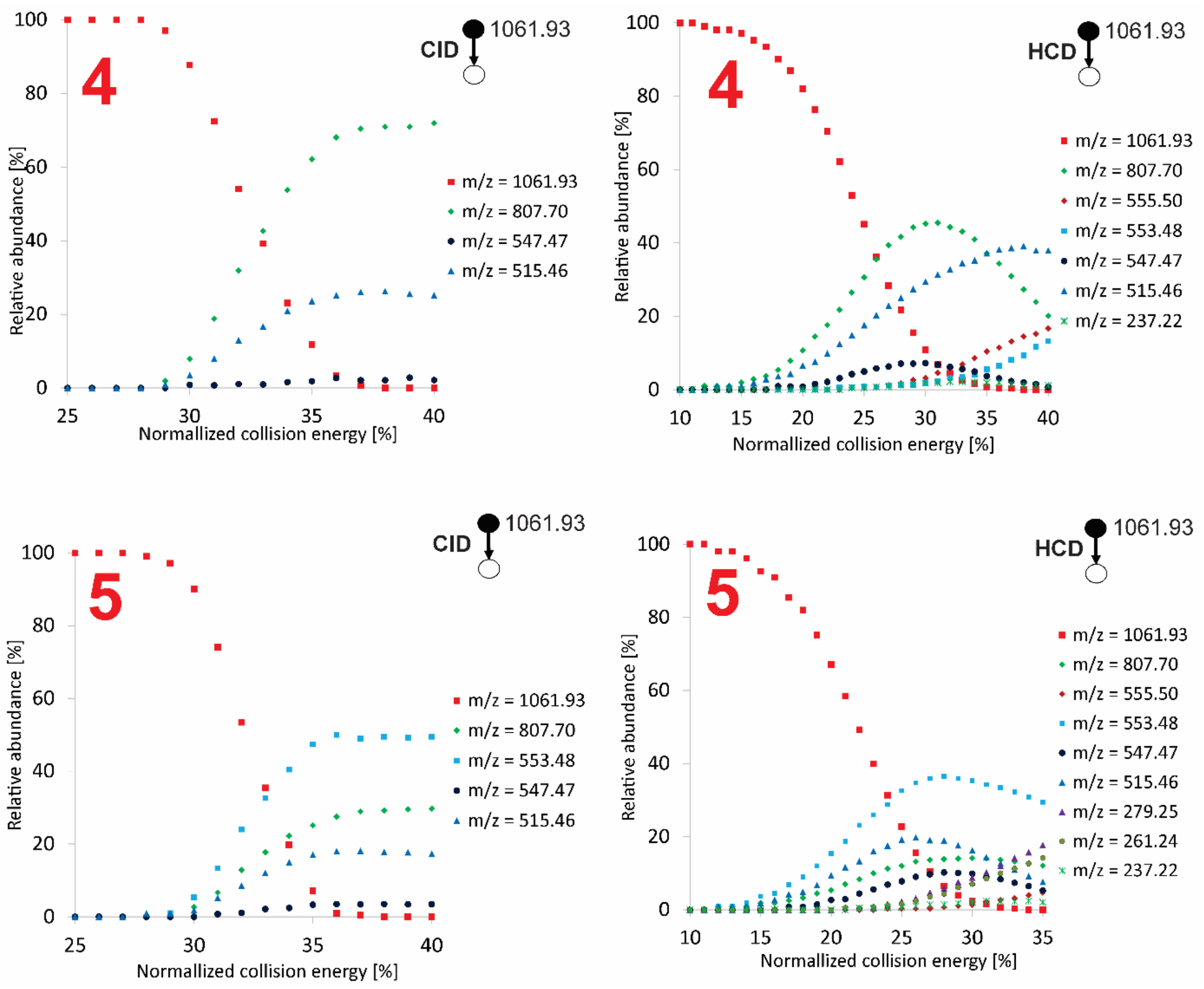

Figure S81. Energy-resolved dissociation curves for $[M + Li]^+$ of TG-EST **4** and TG-EST **5** obtained by CID (left column) and HCD (right column).

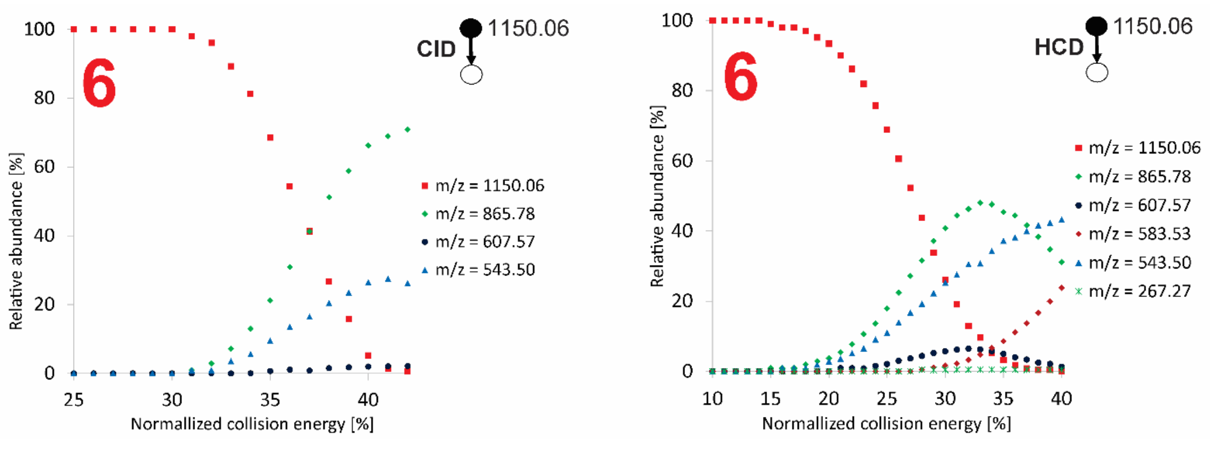

Figure S82. Energy-resolved dissociation curves for $[M + Li]^+$ of TG-EST **6** obtained by CID (left column) and HCD (right column).

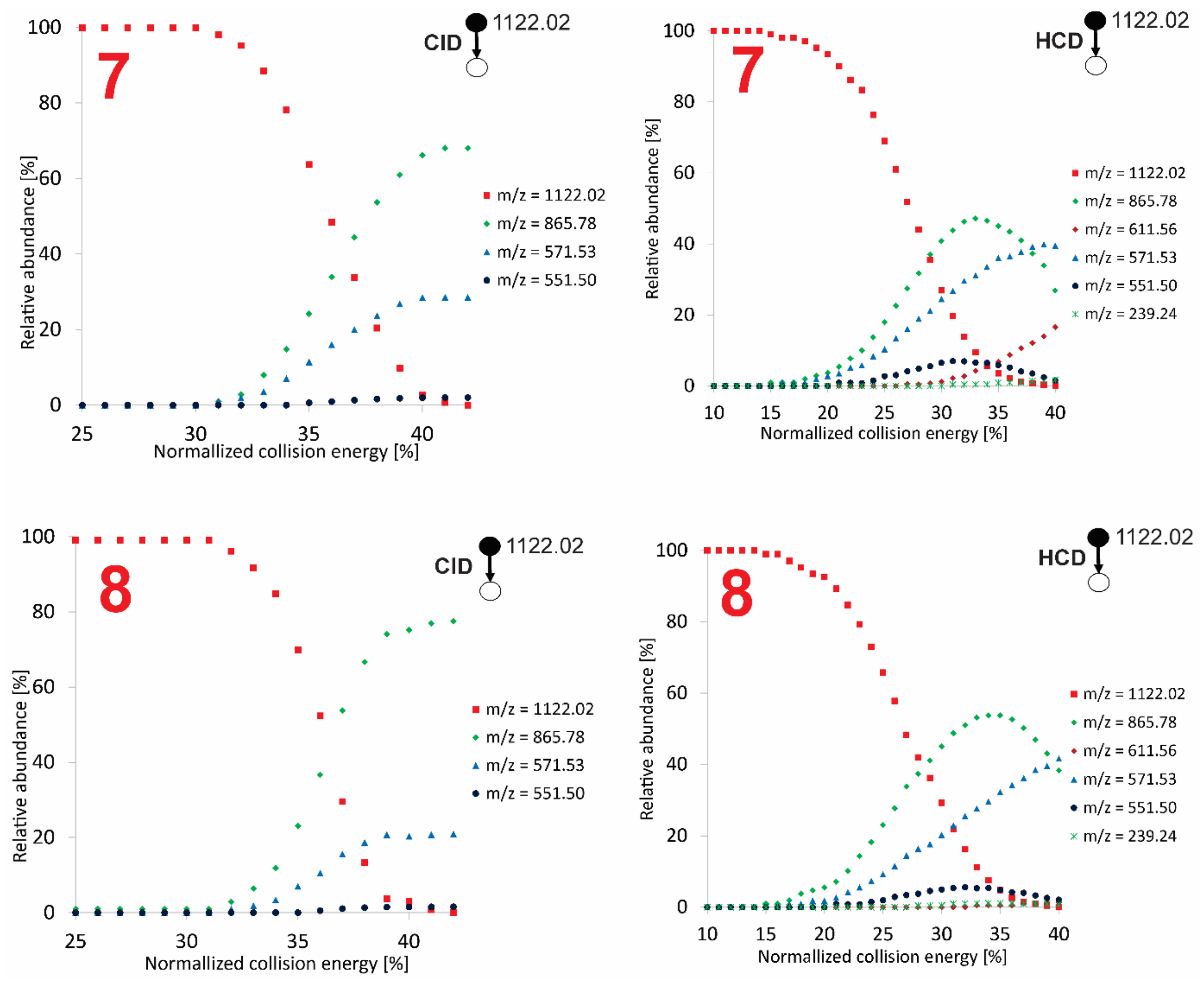

Figure S83. Energy-resolved dissociation curves for $[M + Li]^+$ of TG-EST **7** and TG-EST **8** obtained by CID (left column) and HCD (right column).

2.3 Sodium adducts

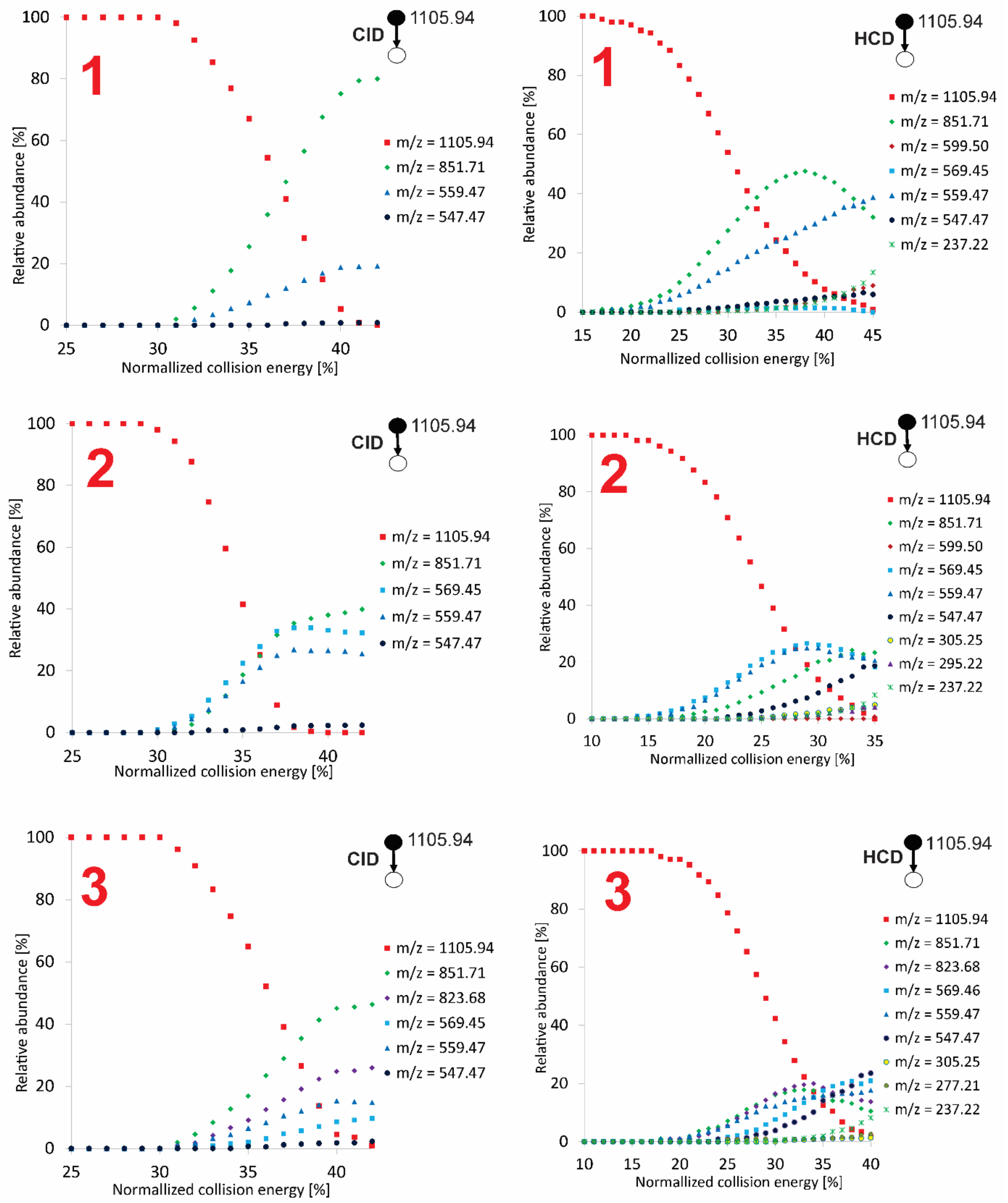

Figure S84. Energy-resolved dissociation curves for $[M + Na]^+$ of TG-EST **1–3** obtained by CID (left column) and HCD (right column).

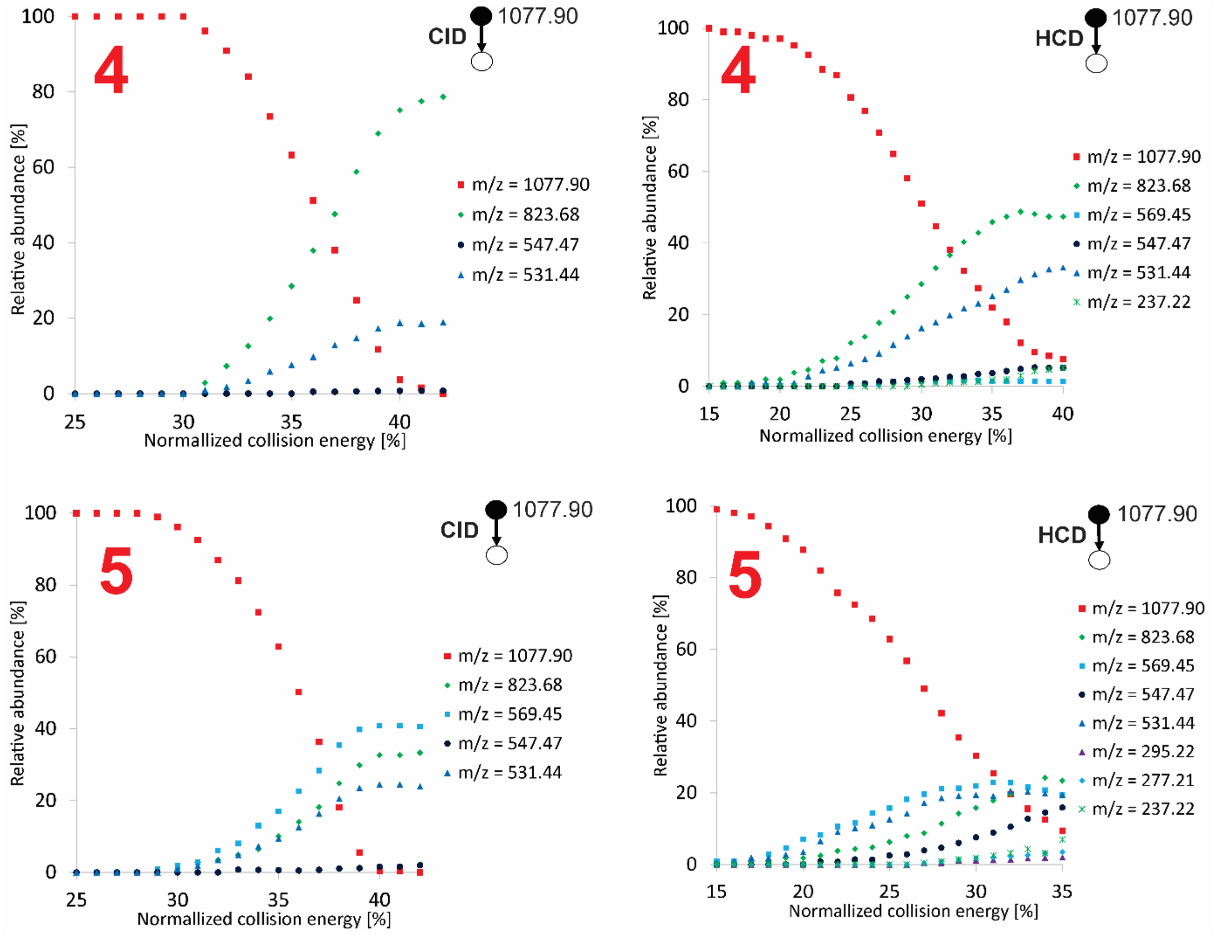

Figure S85. Energy-resolved dissociation curves for $[M + Na]^+$ of TG-EST **4** and TG-EST **5** obtained by CID (left column) and HCD (right column).

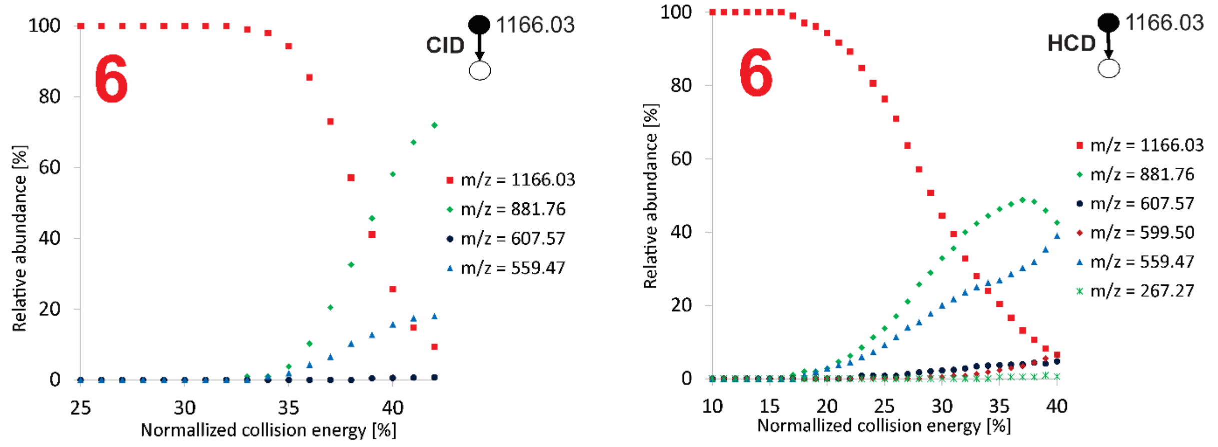

Figure S86. Energy-resolved dissociation curves for $[M + Na]^+$ of TG-EST **6** obtained by CID (left column) and HCD (right column).

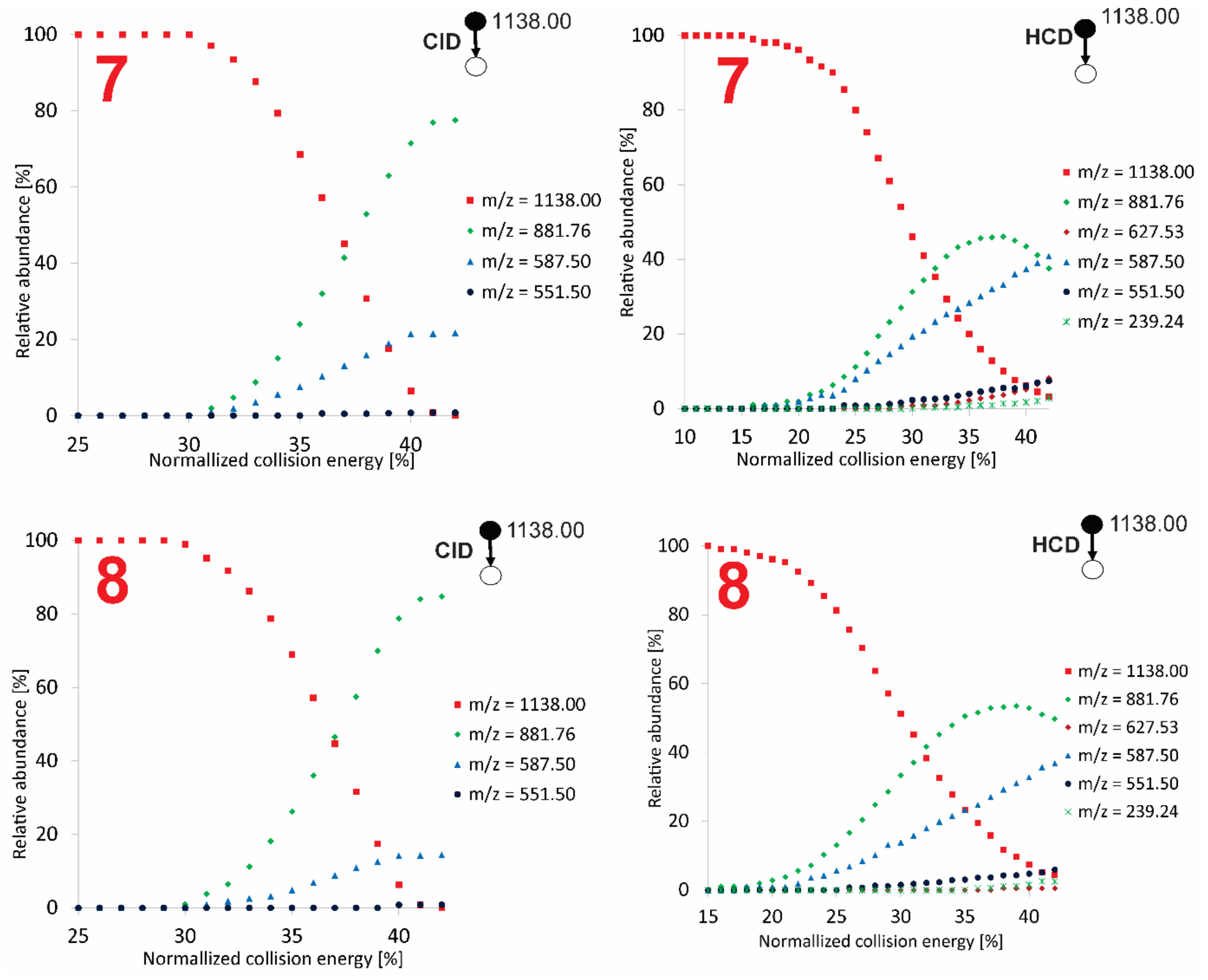

Figure S87. Energy-resolved dissociation curves for $[M + Na]^+$ of TG-EST **7** and TG-EST **8** obtained by CID (left column) and HCD (right column).

3 Fragmentation of ammonium adducts

3.1 MS² HCD spectra of TG-EST 4–8

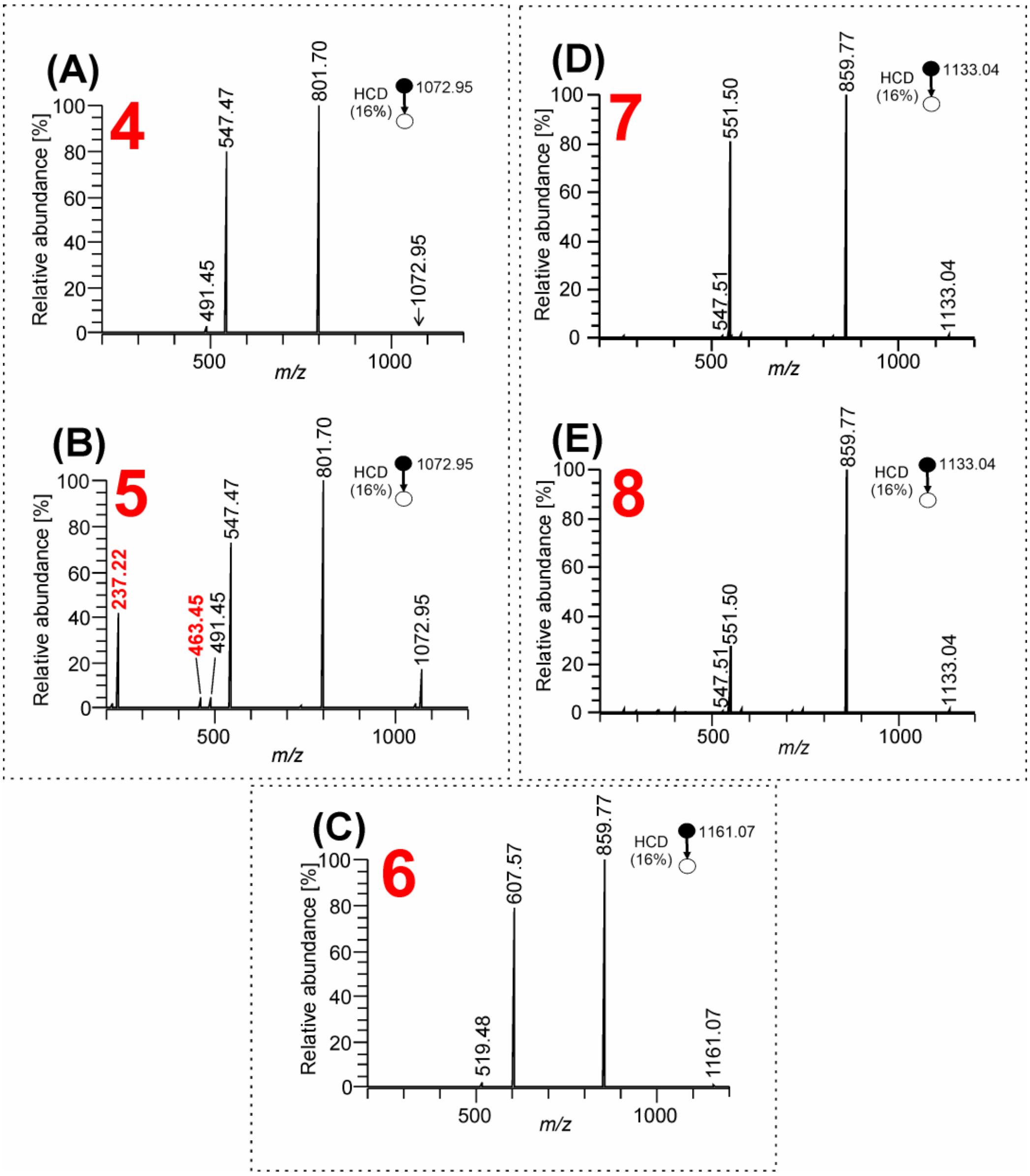

Figure S88. MS^2 HCD spectra of TG-EST $[M + NH_4]^+$ (NCE 16%): **(A)** TG-EST **4** – ω-isomer; **(B)** TG-EST **5** – α-isomer; **(C)** TG-EST **6** – ω-isomer; **(D)** TG-EST **7** – *sn*-1/3-isomer; and **(E)** TG-EST **8** – *sn*-2-isomer. Diagnostic fragment ions for the various positional isomers are highlighted in red. Spectra bounded by a dashed line correspond to isomeric TG-EST with the same mass.

3.2 MS^2 CID spectra of TG-EST 1–6

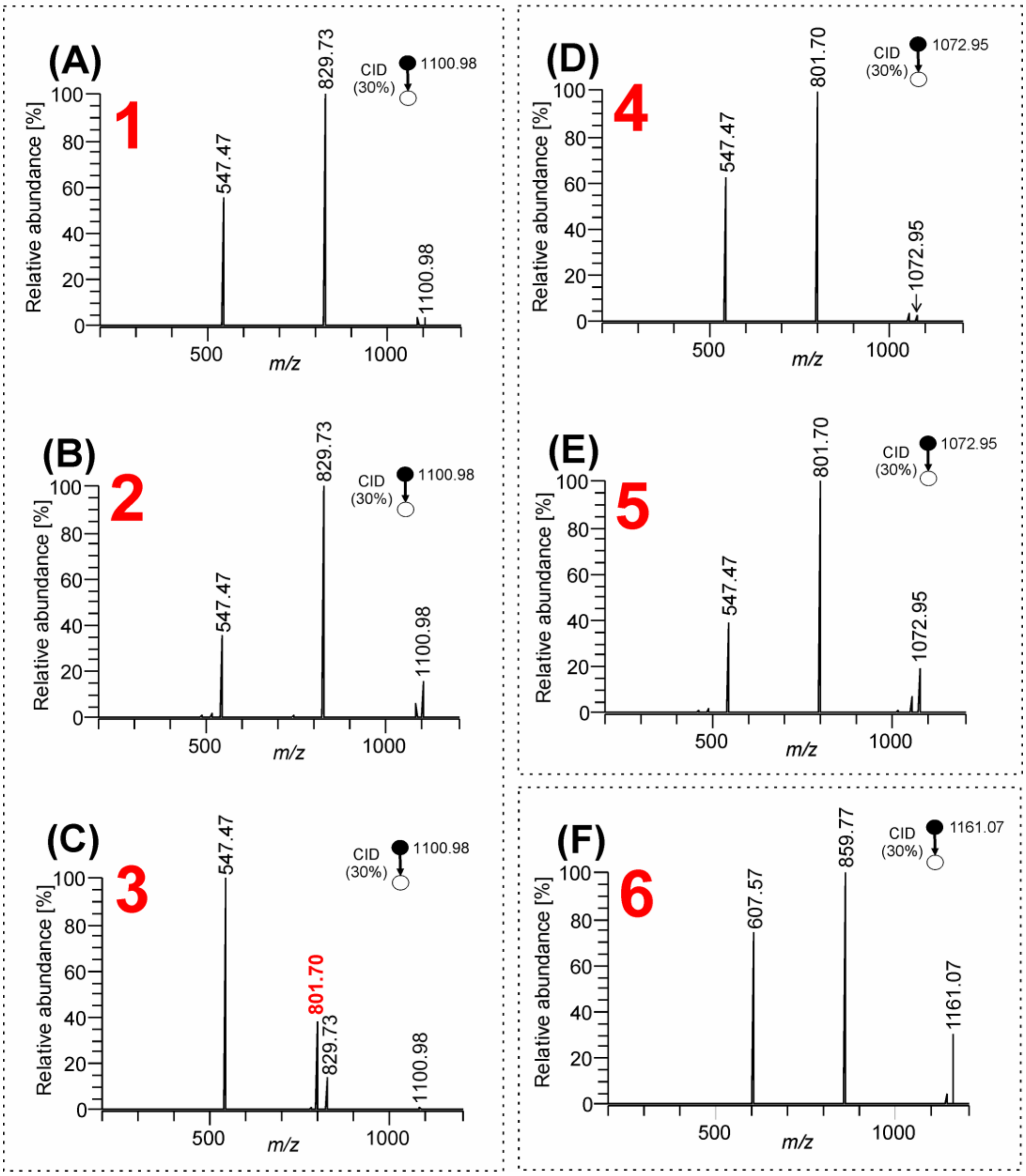

Figure S89. MS^2 CID spectra of TG-EST $[M + NH_4]^+$ (NCE 30%): **(A)** TG-EST **1** – ω-isomer; **(B)** TG-EST **2** – α-isomer; **(C)** TG-EST **3** – 10-isomer; **(D)** TG-EST **4** – ω-isomer, **(E)** TG-EST **5** – α-isomer; and **(F)** TG-EST **6** – ω-isomer. Diagnostic fragment ions for the various positional isomers are highlighted in red. Spectra bounded by a dashed line correspond to isomeric TG-EST with the same mass.

3.3 MS^3 CID/CID spectra of TG-EST 1–3

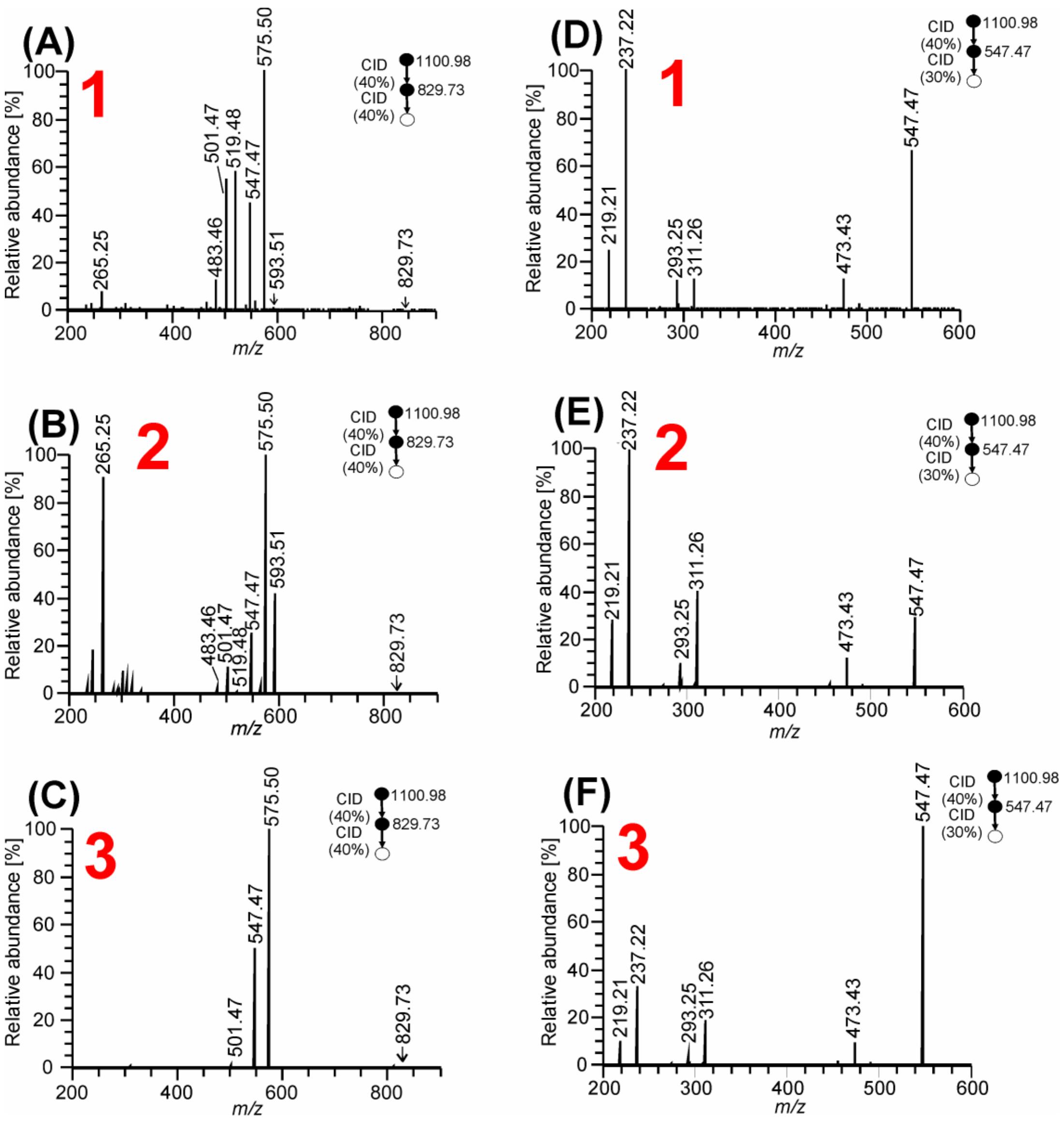

Figure S90. MS^3 CID/CID spectra of TG-EST $[M + NH_4]^+$. The product ion at *m/z* 829.73 arising from $[M + NH_4]^+$ was further fragmented: **(A)** TG-EST **1** – ω-isomer; **(B)** TG-EST **2** – α-isomer; **(C)** TG-EST **3** – 10-isomer. The product ion at *m/z* 547.47 arising from $[M + NH_4]^+$ was further fragmented: **(D)** TG-EST **1** – ω-isomer, **(E)** TG-EST **2** – α-isomer; and **(F)** TG-EST **3** – 10-isomer.

4 Fragmentation of lithium adducts

4.1 Rationalization of *m/z* 583.53 (MS^2 HCD)

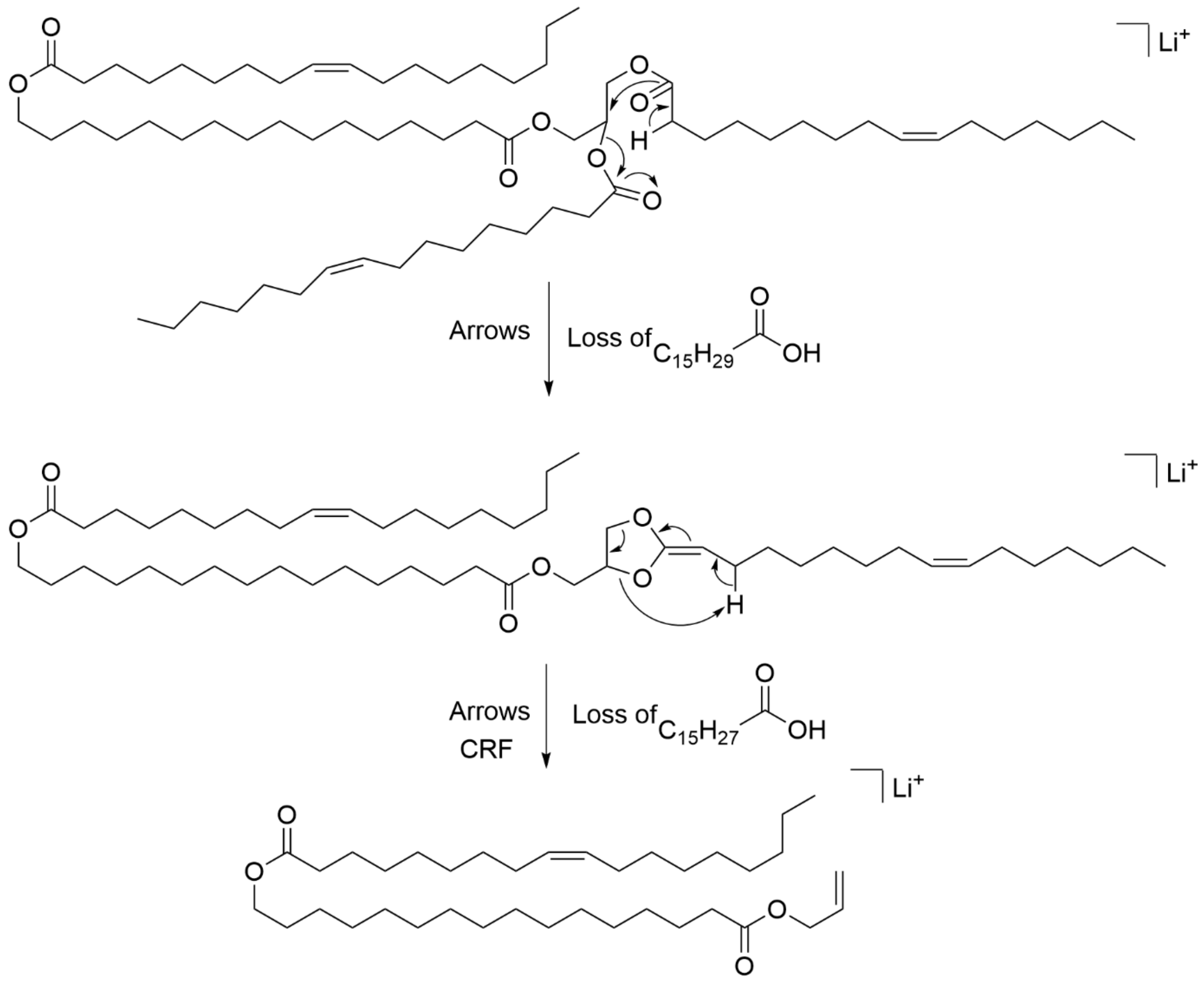

Figure S91. Reaction mechanism proposed for fragmentation of lithiated TG-EST **1** resulting in *m/z* 583.53. From manuscript: Hsu, F.F.; Turk, J. Structural characterization of triacylglycerols as lithiated adducts by electrospray ionization mass spectrometry using low-energy collisionally activated dissociation on a triple stage quadrupole instrument. *Journal of American Society for Mass Spectrometry* **1999**, *10*, 587–599.

4.2 MS^2 HCD spectra of TG-EST 4–8

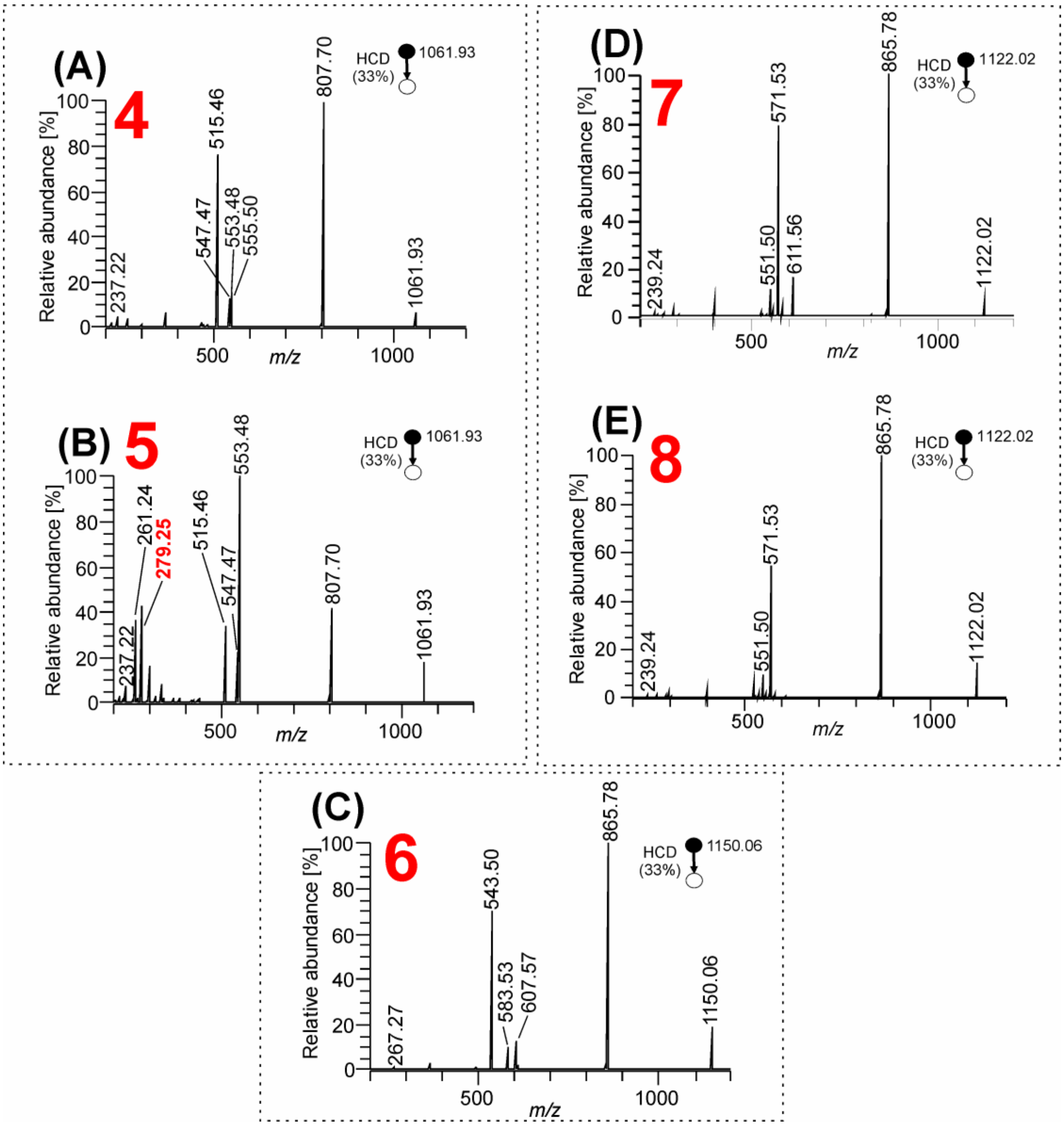

Figure S92. MS^2 HCD spectra of TG-EST $[M + Li]^+$ (NCE 33%): **(A)** TG-EST **4** – ω-isomer; **(B)** TG-EST **5** – α-isomer; **(C)** TG-EST **6** – ω-isomer; **(D)** TG-EST **7** – *sn*-1/3-isomer; and **(E)** TG-EST **8** – *sn*-2-isomer. Diagnostic fragment ions for the various positional isomers are highlighted in red. Spectra bounded by a dashed line correspond to isomeric TG-EST with the same mass.

4.3 MS² CID spectra of TG-EST 1–6

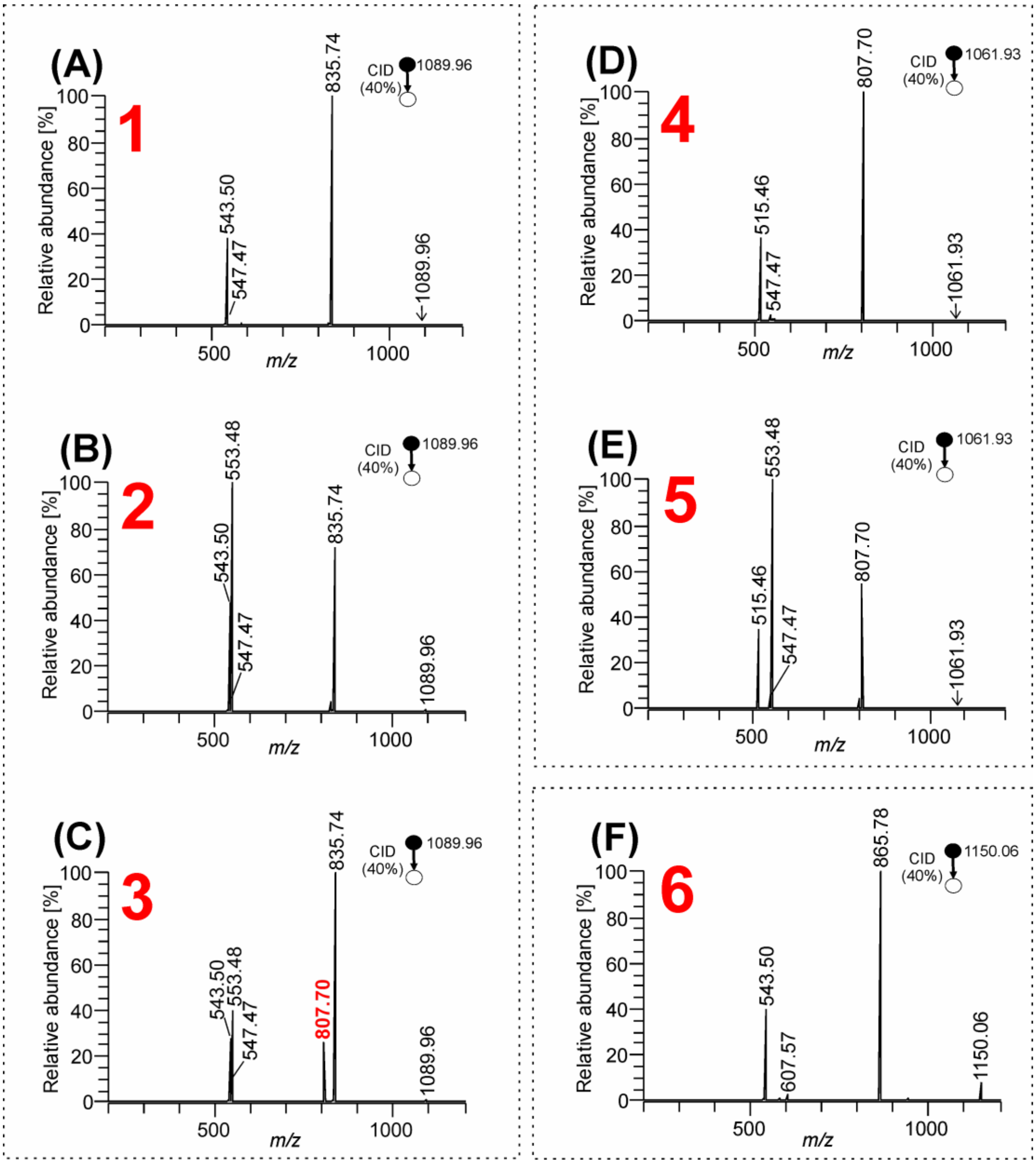

Figure S93. MS^2 CID spectra of TG-EST $[M + Li]^+$ (NCE 40%): **(A)** TG-EST **1** – ω-isomer; **(B)** TG-EST **2** – α-isomer; **(C)** TG-EST **3** – 10-isomer; **(D)** TG-EST **4** – ω-isomer; **(E)** TG-EST **5** – α-isomer; and **(F)** TG-EST **6** – ω-isomer. Diagnostic fragment ions for the various positional isomers are highlighted in red. Spectra bounded by a dashed line correspond to isomeric TG-EST with the same mass.

4.4 MS^4 CID/CID/OzID spectra of TG-EST 1–3

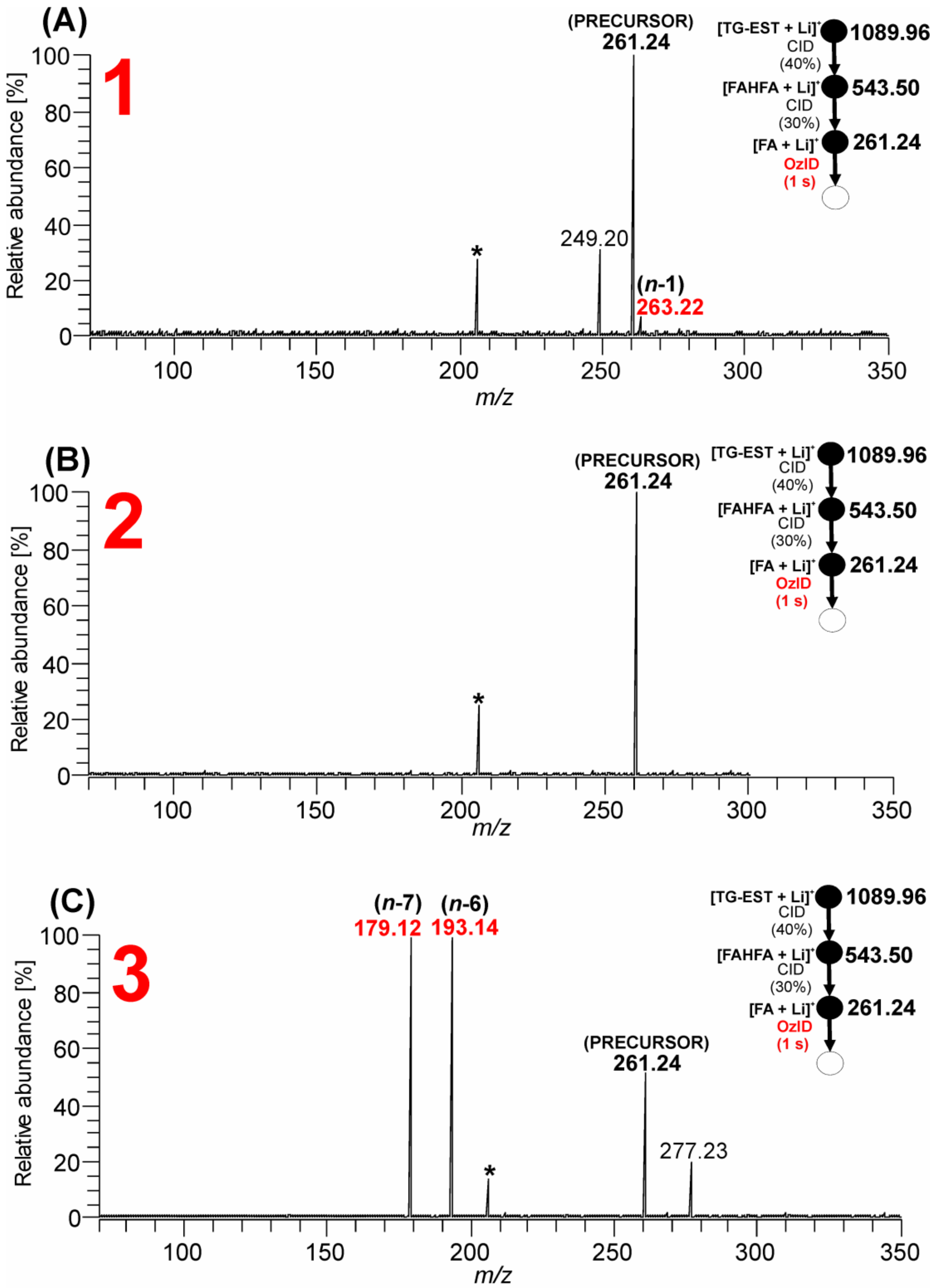

*Fourier transform artifact signal (*m/z* = 206.36)

Figure S94. MS^4 CID/CID/OzID spectra of TG-EST $[M + Li]^+$. The (MS^3) CID product ion at *m/z* 261.24 corresponding to hexadecenoic acid was re-isolated and allowed to react with ozone: **(A)** TG-EST **1** – ω-isomer; **(B)** TG-EST **2** – α-isomer; **(C)** TG-EST **3** – 10-isomer. Diagnostic product ions for the various positional isomers are highlighted in red.

5 Fragmentation of sodium adducts

5.1 MS² HCD spectra of TG-EST 4–8

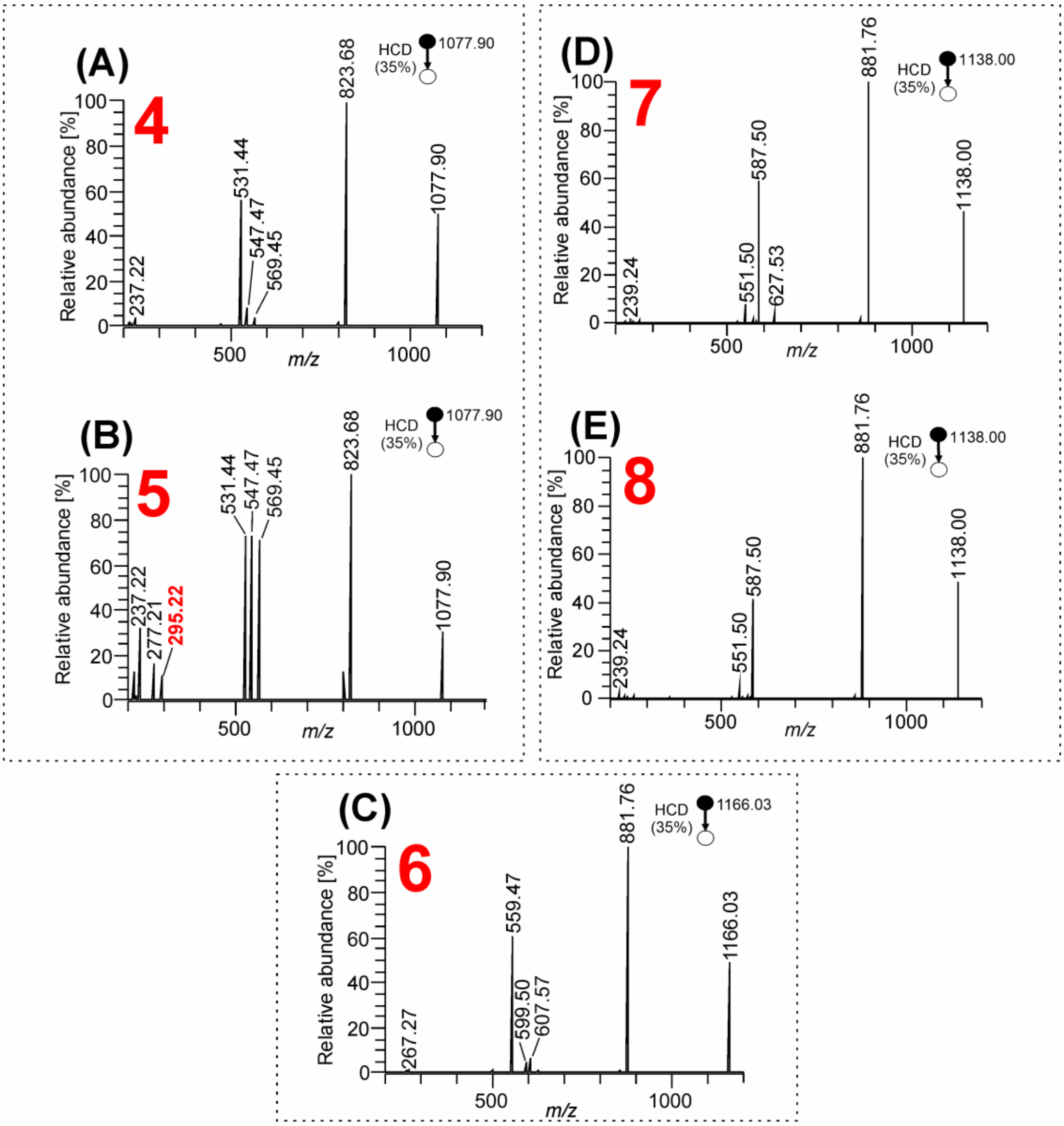

Figure S95. MS² HCD spectra of TG-EST [M + Na]⁺ (NCE 35%): **(A)** TG-EST **4** – ω-isomer; **(B)** TG-EST **5** – α-isomer; **(C)** TG-EST **6** – ω-isomer; **(D)** TG-EST **7** – *sn*-1/3-isomer; and **(E)** TG-EST **8** – *sn*-2-isomer. Diagnostic fragment ions for the various positional isomers are highlighted in red. Spectra bounded by a dashed line correspond to isomeric TG-EST with the same mass.

5.2 MS^2 CID spectra of TG-EST 1–6

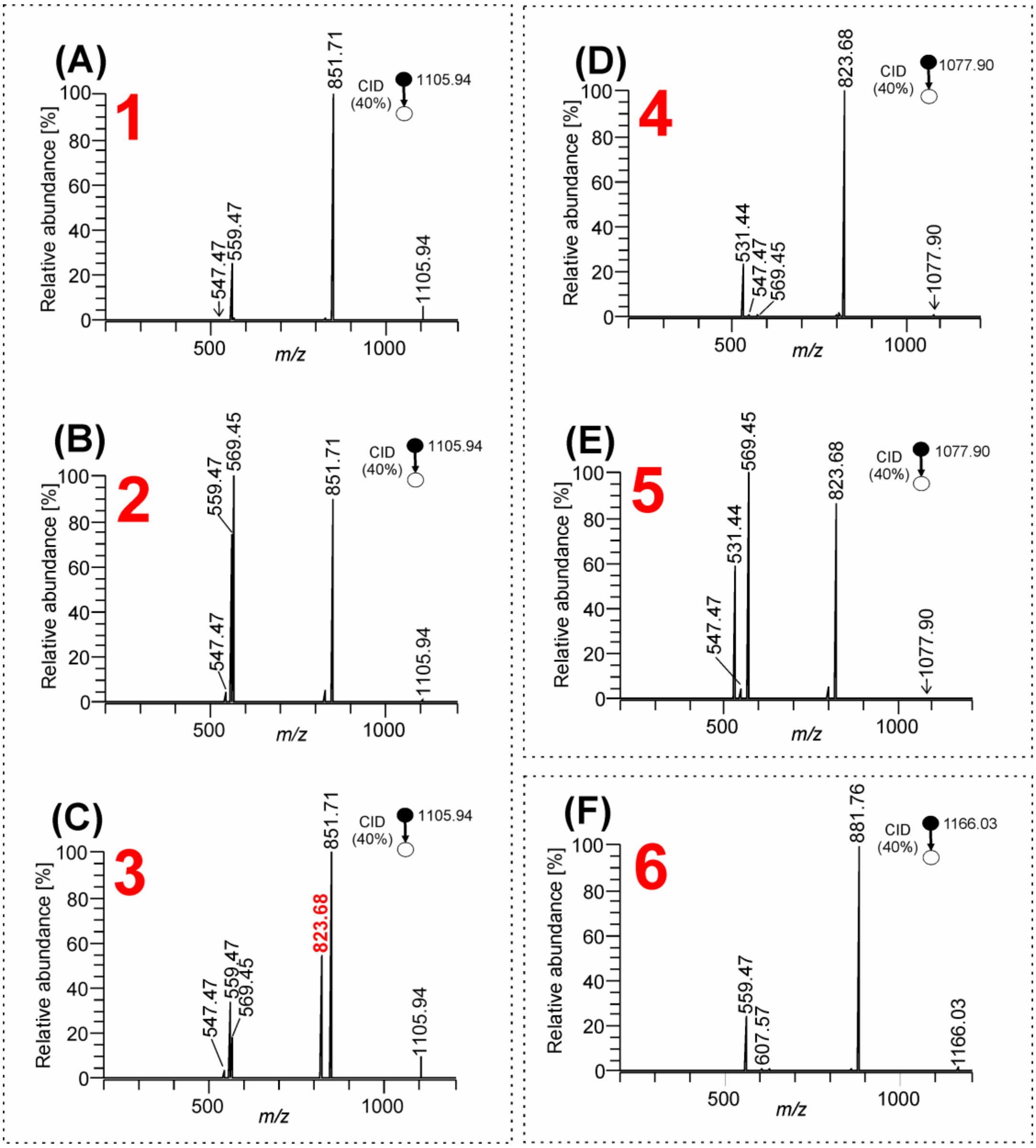

Figure S96. MS^2 CID spectra of TG-EST $[M + Na]^+$ (NCE 40%): **(A)** TG-EST **1** – ω-isomer; **(B)** TG-EST **2** – α-isomer; **(C)** TG-EST **3** – 10-isomer; **(D)** TG-EST **4** – ω-isomer, **(E)** TG-EST **5** – α-isomer; and **(F)** TG-EST **6** – ω-isomer. Diagnostic fragment ions for the various positional isomers are highlighted in red. Spectra bounded by a dashed line correspond to isomeric TG-EST with the same mass.

6 Mass spectra of TG-EST 2 at lower concentrations

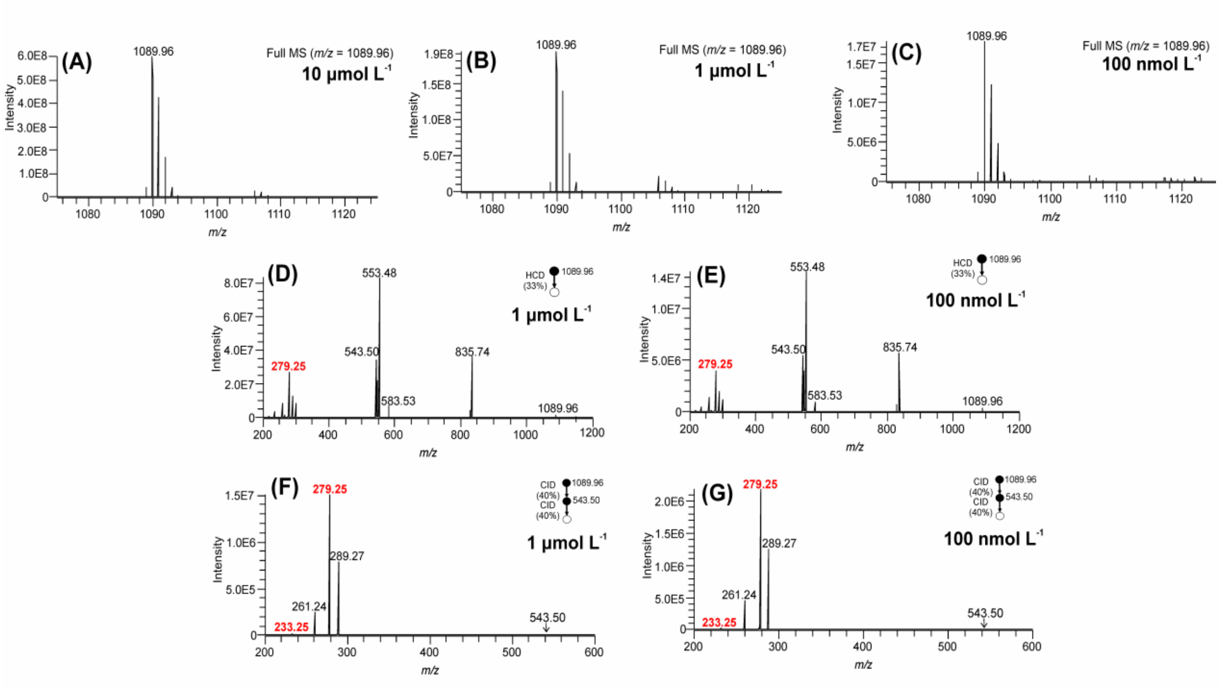

Figure S97. Mass spectra of TG-EST **2**, cationization with lithium ions. Spectra full MS, 10 µmol L^{-1} (**A**); 1 µmol L^{-1} (**B**); 100 nmol L^{-1} (**C**). Spectra MS^2 (HCD), 1 µmol L^{-1} (**D**); 100 nmol L^{-1} (**E**). Spectra MS^3 (CID/CID), 1 µmol L^{-1} (**F**); 100 nmol L^{-1} (**G**).
